# HSF1 Pathway Inhibitor Clinical Candidate (CCT361814/NXP800) Developed from a Phenotypic Screen as a Potential Treatment for Refractory Ovarian Cancer and Other Malignancies

**DOI:** 10.1021/acs.jmedchem.3c00156

**Published:** 2023-04-05

**Authors:** A. Elisa Pasqua, Swee Y. Sharp, Nicola E. A. Chessum, Angela Hayes, Loredana Pellegrino, Michael J. Tucker, Asadh Miah, Birgit Wilding, Lindsay E. Evans, Carl S. Rye, N. Yi Mok, Manjuan Liu, Alan T. Henley, Sharon Gowan, Emmanuel De Billy, Robert te Poele, Marissa Powers, Suzanne A. Eccles, Paul A. Clarke, Florence I. Raynaud, Paul Workman, Keith Jones, Matthew D. Cheeseman

**Affiliations:** Centre for Cancer Drug Discovery and Division of Cancer Therapeutics at The Institute of Cancer Research, London SW7 3RP, United Kingdom

## Abstract

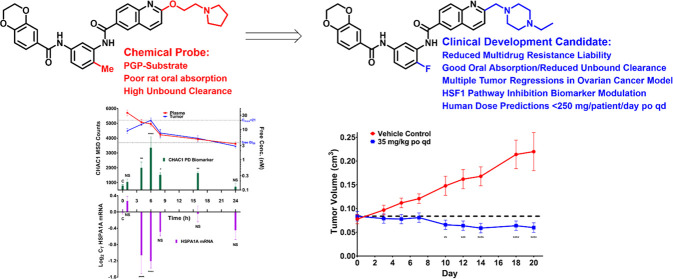

CCT251236 **1**, a potent chemical probe, was previously developed from a cell-based phenotypic high-throughput screen (HTS) to discover inhibitors of transcription mediated by HSF1, a transcription factor that supports malignancy. Owing to its activity against models of refractory human ovarian cancer, **1** was progressed into lead optimization. The reduction of P-glycoprotein efflux became a focus of early compound optimization; central ring halogen substitution was demonstrated by matched molecular pair analysis to be an effective strategy to mitigate this liability. Further multiparameter optimization led to the design of the clinical candidate, CCT361814/NXP800 **22**, a potent and orally bioavailable fluorobisamide, which caused tumor regression in a human ovarian adenocarcinoma xenograft model with on-pathway biomarker modulation and a clean in vitro safety profile. Following its favorable dose prediction to human, **22** has now progressed to phase 1 clinical trial as a potential future treatment for refractory ovarian cancer and other malignancies.

## Introduction

Ovarian cancer is the most lethal and the second most common gynecological malignancy in the developed world, and only a modest decrease in mortality has been achieved over the past three decades.^1^ Approximately 80% of patients are diagnosed at an advanced stage, leading to poor prognosis with little prospect of cure.^1^ The combination of surgical cytoreduction and the administration of platinum complexes and taxanes remains the standard of care for advanced ovarian cancer.^2^ Although initial treatment is effective in ∼70% of patients, and the introduction of the anti-VEGF receptor monoclonal antibody bevacizumab and PARP inhibitors provides a welcome addition to initial therapy, the majority develop drug resistance and relapse, resulting in a 5 year survival rate of only ∼30%.^3^ Clearly, there is a high unmet medical need in the treatment of ovarian cancer.

Multidrug resistance (MDR) in relapsed ovarian cancer is observed in 50–75% of patients following first-line chemotherapy.^4^ MDR is often the result of the overexpression of ABC-transporter proteins at the cancer cell surface, which efflux compounds and reduce their intracellular free concentrations.^5^ Various ABC-transporter proteins have been linked to MDR with oncology drugs,^6^ but the most commonly encountered is the overexpression of multidrug resistance protein 1 (MDR1), also known as the P-glycoprotein (P-gp) efflux pump.^7^ Consequently, the reduction of P-gp-mediated efflux in lead optimization is important for the successful development of novel and effective ovarian anticancer therapies.^7^

Heat shock transcription factor 1 (HSF1) is the master regulator of the canonical heat shock stress response.^8^ In cancer, HSF1 is important for tumorigenesis and progression and is activated by various elements of the cancer state.^9^ HSF1 reprograms the transcriptome in a manner overlapping with, but distinct from, the classical heat shock response.^10^ Also, HSF1 is amplified, overexpressed, or activated in multiple human cancers; these features, combined with the oncogenic HSF1 gene signature, predict adverse clinical outcomes, including in ovarian cancer.^11,12^ Moreover, in ovarian cancer cells, the shRNA knockdown of HSF1 leads to decreased proliferation and increased apoptosis.^12^ In contrast, the knockout of HSF1 is tolerated in flies and mice.^12^ Together with a range of other data, these results support the inhibition of HSF1 as a “nononcogene addiction” approach to exploit tumor stress with the potential to antagonize multiple hallmark cancer traits.^12^ Unfortunately, HSF1 is a ligandless transcription factor and is predicted to be very difficult-to-drug directly. Therefore, we sought HSF1 pathway inhibitors that could indirectly inhibit HSF1-mediated transcription.

**Figure 1:**
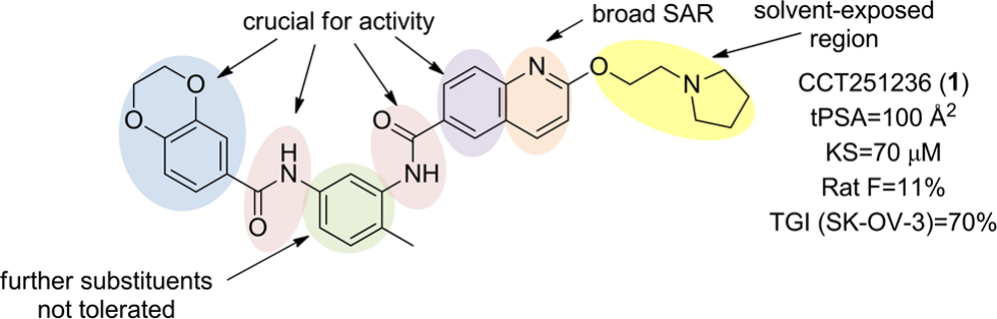
Complex cellular structure–activity relationships (SARs) of the bisamide chemotype and poor rat-free exposure from an oral dose of the lead compound **1**.

We previously reported the discovery of a new chemical probe, CCT251236 **1** (Figure 1), which was developed from a low solubility hit identified using a proximal but mechanism-agnostic phenotypic screen to detect inhibitors of the HSF1 stress pathway.^13^ Bisamide **1** displayed potent cellular activity in the human ovarian cancer cell line SK-OV-3, blocking HSP72 induction by an HSP90 inhibitor, which was used as a surrogate biomarker of HSF1 pathway inhibition (IC_50_ = 19 nM). Also, consistent with the sensitivity to HSF1 RNAi knockdown both in vitro and in vivo,^12^**1** displayed excellent antiproliferative activity against cancer cells (GI_50_ = 2.2 nM) as a single agent. Furthermore, we demonstrated that bisamide **1** is a potent ligand for the putative transcription factor regulator pirin (*K*_D_ = 44 nM), with clear in vitro antimigratory activity, the phenotype previously associated with pirin binding, in the melanoma cell line WM266.4, at low free concentrations (<100 nM).^13^ Subsequently, intracellular target engagement with pirin by **1** in intact cancer cells was confirmed via a CRBN-mediated PROTAC probe.^14^ However, the molecular mechanism of action for the wide-ranging antiproliferative activity of this chemotype still remains to be confirmed. Finally, in the in vivo SK-OV-3 human ovarian cancer solid tumor xenograft model in nude mice, bisamide **1** was shown to be orally bioavailable and displayed clear efficacy (tumor growth inhibition (TGI) = 70%), driven by a low free drug exposure (free *C*_av_^0–24h^ = 1.2 nM)^15^ achieved using a well-tolerated intermittent 20 mg/kg po dosing regimen.^13^

We now report the development of the probe HSF1 pathway inhibitor **1** into a clinical candidate, which shows future potential for the treatment of relapsed ovarian cancer and other malignancies. We developed the compound using only cell-based structure–activity relationships (SARs) and focused on improving the oral absorption while reducing in vivo unbound clearance and the P-gp-efflux-mediated multidrug resistance risk.

## Results and Discussion

### Targeting Ovarian Cancer

It has been proposed that HSF1 pathway inhibition could be an effective treatment in relapsed ovarian cancer,^12^ as well as other malignancies;^16^ therefore, to assess the development potential of probe bisamide **1**, the compound was screened against a panel of genetically diverse human ovarian cancer cell lines (Table S1). Compared to the standard-of-care drug carboplatin (pGI_50_ < 6, *N* = 4 cell lines), bisamide **1** displayed significantly more potent antiproliferative activity against this panel (8.7 > pGI_50_ > 7.3, *N* = 9 cell lines). Given the challenges in treating relapsed ovarian cancer and the clear treatment potential of this chemotype, bisamide **1** was progressed into lead optimization.

### Rodent Pharmacokinetic (PK) Optimization

A pharmacokinetic (PK) study in Sprague-Dawley (SD) rats (Table S13) revealed that bisamide **1** possessed poor oral bioavailability (*F* = 11%) from moderate total blood clearance (CL_tb_ = 20 mL/min/kg, extraction ratio = 29%, *F*_max_ = 71%) with moderate in vivo unbound clearance (CL_u_ = 1100 mL/min/kg, *f*_ub_ = 0.019). Unbound clearance, when acting as a suitable estimate for unbound intrinsic clearance, is an important target parameter owing to its relationship with free drug exposure and unbound average concentration.^17,18^ In vitro, low-to-moderate passive permeability was observed in the Caco-2 assay, which is commonly used to predict absorption^19^ (*A*–*B* = 2.4 × 10^–6^ cm/s, efflux ratio = 16; Table S8). The high efflux ratio indicated that **1** is likely to suffer from P-gp-mediated efflux, which can present a challenge for clinical development. Therefore, we began a medicinal chemistry campaign to improve the preclinical PK profile of this highly potent and effective chemotype to increase oral absorption, reduce unbound clearance, and mitigate the risk of P-gp-mediated efflux.

**Scheme 1:**
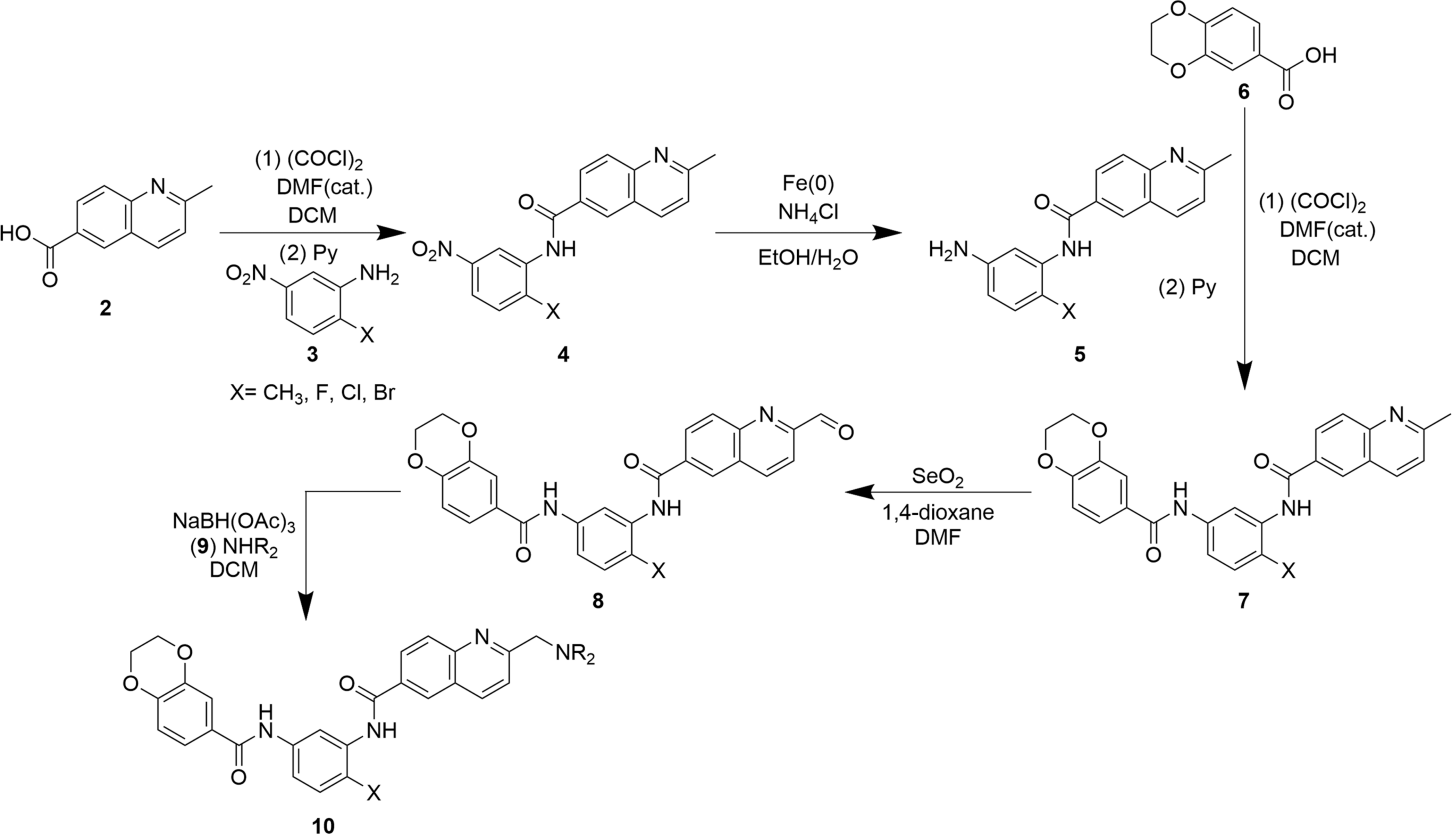
Generic Synthesis of Bisamide Lead Optimization Analogues

Bisamide **1** represents a challenging start-point for lead optimization, as the cellular SAR of this chemotype is complex, with steep activity cliffs from small structural changes and few clear patterns to drive compound development.^13^ The incorporation of a solubilizing group had been crucial for the favorable mouse PK profile of bisamide **1**, so to carry out multiparameter optimization on the chemotype, we focused on structural changes to this region. The solubilizing group of each analogue was inferred to be solvent-exposed due to its tolerance to a broad range of structural changes in the cell-based assays.^13^ To expedite analogue evaluation, a new synthetic route was developed incorporating a late-stage selenium dioxide-mediated benzylic oxidation (Scheme 1).^14,20^

2-Methylquinoline-6-carboxylic acid **2** was converted to the acid chloride using oxalyl chloride and catalytic *N*,*N*-dimethylformamide (DMF), before reacting with 1,3-nitroaniline **3** to give nitroamide **4**. Iron(0)-mediated reduction of the nitro group gave **5**, which was then subjected to a second amide bond formation reaction following the in situ generation of the acid chloride of the dihydrobenzodioxine-carboxylic acid **6** to give bisamide **7**. Subsequent selenium dioxide-mediated oxidation of the quinolinic methyl group of **7** gave aldehyde **8** in low to moderate yields, as both the optimal reaction temperature and ratio of 1,4-dioxane/DMF cosolvents were dependent on the benzylic substituent, X. This optimization was also critical owing to the poor solubility of bisamide **7** in 1,4-dioxane and the tendency of the aldehyde to overoxidize to the carboxylic acid under the reaction conditions. Aldehyde **8** then underwent reductive amination with various amine bases **9** under standard conditions and in moderate yields to give analogues **10**. Variations on this general route were also carried out to synthesize specific analogues, and details are available in the Experimental Section and Supporting Information.

Our first target for lead optimization was to improve oral absorption in a manner that would be tolerated as part of the multiparameter optimization and would maintain the potent antiproliferative activity of this chemotype. Therefore, we replaced the linker to the solubilizing group with a shorter chain and removed the oxygen in bisamide **1**, reducing both topological polar surface area (tPSA) and basicity without significantly increasing lipophilicity (Table 1).

**Table 1:**
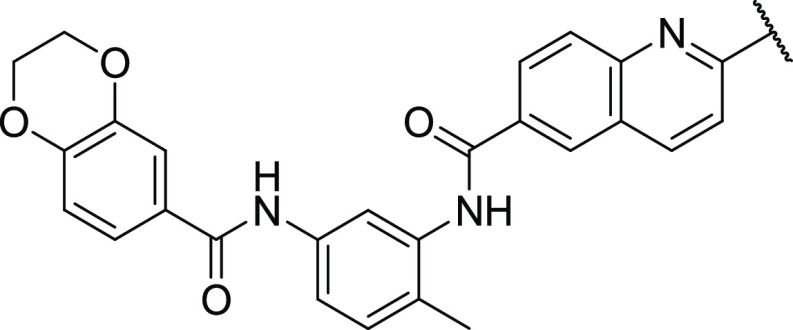

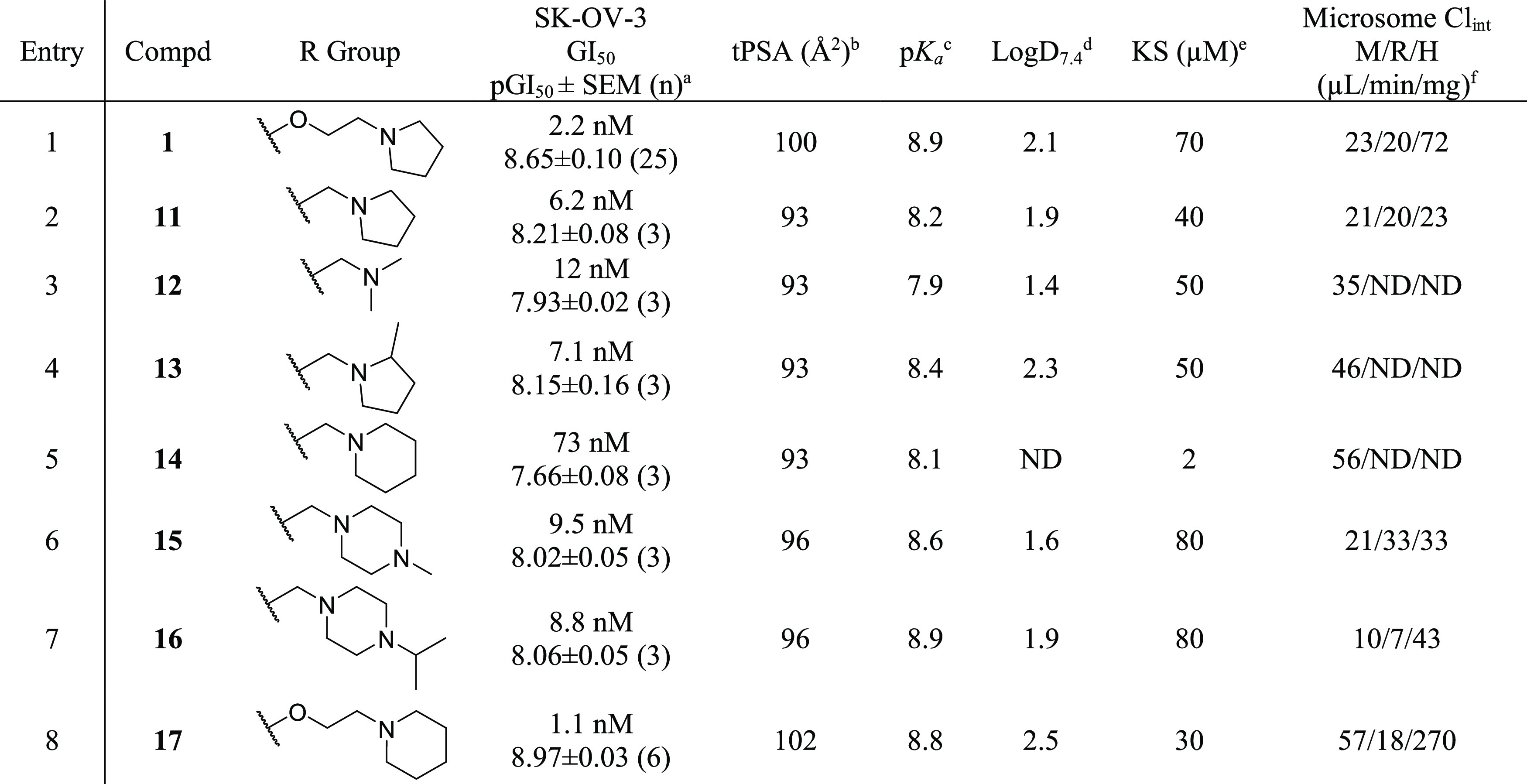
Solubilizing Group In Vitro Optimization

a All data were reprocessed using GraphPad Prism 7.01. Growth inhibition was measured after 96 h of treatment and compared to the vehicle control using the CellTiter-Blue assay. GI_50_ values were estimated by fitting a log[Inhibitor] vs response—variable slope (four-parameter) model. The number of repeats (*n*) is given in parentheses. All results are quoted as the geometric mean ± standard error of the mean (SEM). pGI_50_ = −log GI_50_ (M).

b Calculated using ChemDraw 16.0.1.4 and quoted to 0 dp.

c Calculated using MoKa version 2.5.2; all values quoted to 2 SF.

d Measured using a previously described high-performance liquid chromatography (HPLC) method, *n* = 1; all values quoted to 2 SF.^13^

e Kinetic solubility (KS) measured via an HPLC method from phosphate buffer at pH 7.4; all values quoted to 1 SF; the dynamic range of the assay is 1–100 μM, *n* = 1.

f Mouse (M), rat (R), and human (H) liver microsome (MLM, RLM, and HLM) assays were carried out at Cyprotex, *n* = 1. In vitro Cl_int_ values are calculated from the half-lives using standard procedures. Assumes the fraction unbound in the assay is 1.

g ND, not determined.

The one-carbon linker analogue, methylene **11** (Table 1, entry 2), displayed a 2.8-fold decrease in antiproliferative activity but only a 1.7-fold decrease in kinetic solubility (KS, used as a crude estimate of thermodynamic solubility), despite the predicted decrease in p*K*_a_ compared to **1** (8.2–8.9, respectively). Both analogues displayed similar lipophilicity (Log *D*_7.4_), but **11** exhibited a 3.1-fold reduction in human liver microsome (HLM) intrinsic clearance (CL_int_) (23 vs 72 μL/min/mg) while maintaining comparable microsomal stability to **1** in both rodent species (RLM CL_int_ = 20 μL/min/mg).^21^

The reduced tPSA (93 Å^2^), combined with the improved metabolic stability profile, led us to investigate this structural change further with a series of solubilizing group analogues (Table 1, entries 3–7), with compound design focusing on maintaining antiproliferative activity while improving metabolic stability. The potential for progressing compounds to in vivo mouse PK evaluation was assessed through changes in physicochemical properties and microsomal clearance data.

Comparing subsequent analogues to **11**: the acyclic dimethylamino analogue **12** (Table 1, entry 3) displayed a 1.3-fold increase in KS (50 μM), but unfortunately, also a 1.6-fold increase in MLM CL_int_, presumably due to CYP450-mediated *N-*demethylation. Therefore, all subsequent analogues were limited to cyclic structures that should display increased resistance to oxidation.

The more lipophilic analogues, methylpyrrolidine **13** and piperidine **14** (Table 1, entries 4 and 5), suffered a decrease in metabolic stability and KS (2.0–50 μM), so to balance their physicochemical properties, an additional nitrogen was introduced to the six-membered ring of the solubilizing group. The *N-*methylpiperazine derivative **15** (Table 1, entry 6) displayed a favorable 2.0-fold increase in KS (80 μM), combined with metabolic stability comparable to **11**. Hypothesizing that the *N-*methyl moiety of **15** remained an oxidative metabolic liability, the *N-*isopropylpiperazine analogue **16** was synthesized (Table 1, entry 7) and, consistent with our design strategy, the metabolic stability was compared favorably to that of lead bisamide **1** across all species (RLM CL_int_ = 7.0 μL/min/mg, HLM CL_int_ = 43 μL/min/mg) while maintaining improved KS (80 μM). Compound **16** was therefore selected for further evaluation in mouse PK studies (Table 2, entry 1).

**Table 2:** Selected In Vivo Mouse Blood PK Parameters of Lead Compounds^a^

Entry	Compd	Dose po/iv (mg/kg)^b^	*T*_max_ (h)	po AUC^0–6h^ (h*nM)	iv Cl_tb_ (mL/min/kg)^c^	*t*_1/2_ (h)^d^	*F* (%)^e^	*f*_ub_^f^	AUC_u_^0–6h^ (h*nM)^g^	Free *C*_av_^0–24h^ (nM)^h^	CL_u_ (mL/min/kg)^i^
1	**16**	5/5	1.7	430 (890–210)	34 (36–32)	1.2	11	0.032	14	0.66	1100
2	**17**	5/5	2.3	830 (910–750)	40 (48–32)	1.7	24	0.015	12	0.83	2700
3	**18**	5/1	1.7	2400 (2800–2000)	33 (35–31)	2.0	63	0.011	26	1.6	3000
4	**21**	5/5	1 7	3900 (4900–3000)	2.8 (33–25)	2.3	96	0.0053	20	1.3	5300

a All graphs were plotted using GraphPad Prism 7.01. PK parameters were derived from the blood concentration/time using noncompartmental analysis (Model 200 and 201) (Phoenix, version 6.1). All results are quoted to two significant figures as the geometric mean of *n* = 3 individual BALB/c mice. The 90% confidence intervals are in parentheses.

b The po and iv dosing vehicles are described in the Supporting Information.

c CL_tb_ = total blood clearance.

d Terminal half-life calculated from the iv dose.

e Assumes linear PK.

f *f*_ub_ = fraction unbound in blood, *f*_ub_ = *f*_up_/B:P, *f*_up_ = fraction unbound in plasma using equilibrium dialysis, B:P = blood-to-plasma ratio and quoted as the geometric mean from *n* = 3 statistical repeats from pooled samples; see the Supporting Information for details.

g AUC_u_ = AUC*f_ub_.

h Free *C*_av_^0–24h^ = AUC^inf^/24*f_ub_.

i CL_u_ = CL_tb_/*f*_ub_.

### P-gp-Mediated Efflux

*N-*Isopropylpiperazine **16** was dosed in BALB/c mice, both as an oral solution and iv bolus, and blood concentrations were measured over a 24 h period. Unfortunately, the in vivo PK profile of **16** was disappointing, with low oral bioavailability (11%) from moderate total blood clearance (CL_tb_ = 34 mL/min/kg, extraction ratio = 38%, *F*_max_ = 62%),^22^ corresponding to an unbound clearance of 1100 mL/min/kg (Table S4). The predicted CL_u_ from the MLM assay was only 39 mL/min/kg,^23^ 28-fold lower than the experimental result (Table 3, entry 1 and Table S5). We hypothesized that this disconnection between predicted and experimentally determined CL_u_ was due to P-gp-mediated efflux increasing biliary excretion in vivo, which could not be predicted by the MLM assay.^24^

**Table 3:**
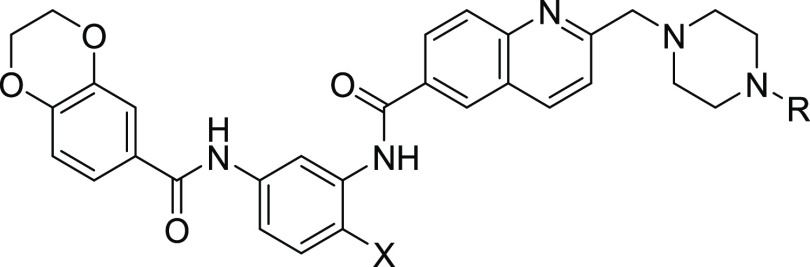
Multiparameter Optimization of the Piperazine Subseries^a^

Entry	Compd	R group	X	SK-OV-3 GI_50_ pGI_50_ ± SEM (*n*)^b^	CH1^doxR^/CH1^WT^^c^	Log *D*_7.4_^d^	KS (μM)^e^	MLM (μL/min/mg)^f^	Pred. in vivo CL_u_ from MLM (mL/min/kg)^j^	Mouse Heps (μL/min/10^6^)^g^	Pred. in vivo CL_u_ from Heps (mL/min/kg)^j^
1	**16**	*^i^*Pr	Me	8.8 nM 8.06 ± 0.05 (3)	8.4	1.9	80	10	39	20	460^h^
2	**18**	*^i^*Pr	Cl	15 nM 7.83 ± 0.08 (8)	1.7	2.3	70	17	65	28	930^i^
3	**19**	H	Cl	12 nM 7.94 ± 0.01 (3)	5.2	ND	ND	12	ND	ND	ND
4	**20**	Me	Cl	19 nM 7.72 ± 0.16 (3)	1.2	2.1	30	110	ND	ND	ND
5	**21**	Et	Cl	11 nM 7.96 ± 0.09 (10)	1.4	2.2	50	26	100	71	1400^i^
6	**22**	Et	F	8.5 nM 8.07 ± 0.02 (45)	1.8	1.7	50	15	58	53	900^h^

a All data were reprocessed using GraphPad Prism Version 7.01. ND = not determined. All results are quoted to two significant figures unless otherwise stated.

b The number of repeats (*n*) are described in parentheses; growth inhibition was measured after 96 h of treatment and compared to the vehicle control; all results are quoted as the geometric mean ± SEM, pGI_50_ = −log GI_50_ (M).

c The fold resistance is determined by the ratio of geometric mean GI_50_ values in CH1^doxR^ cells and CH1^WT^ cells in the CellTiter-Blue growth inhibition assay.

d Measured via an HPLC method, *n* = 1.

e Measured via an HPLC method from phosphate buffer at pH 7.4; all values quoted to 1 SF; the dynamic range of the assay is 1–100 μM, *n* = 1.

f Mouse liver microsome (MLM) assay was carried out at Cyprotex, *n* = 1; in vitro CL_int_ (μL/min/mg of protein) is calculated from the half-life using standard procedures and assumes that the fraction unbound in the assay is 1.

g Mouse hepatocyte assay was carried out at Cyprotex, *n* = 1; in vitro CL_int_ (μL/min/10^6^ cells) is calculated from the half-life using standard procedures.

h Assumes that the fraction unbound in the assay is 0.4.

i Assumes that the fraction unbound in the assay is 0.2.

j Calculated from the in vitro CL_int_ using scaling factors and applying the well-stirred model; see the Supporting Information for details.

To assess the effect of P-gp-mediated efflux on in vivo clearance, **16** was submitted for comparative studies in wild-type (CF1^WT^) and P-gp-knockout (CF1^PGP-KO^) mice (Figure S8).^25^ The PK data revealed that the CL_tb_ in CF1^WT^ (35 mL/min/kg) was significantly higher than in the CF1^PGP-KO^ (24 mL/min/kg, *p* = 0.024, Student’s *t*-test), indicating that P-gp-efflux did contribute to the unfavorable mouse PK profile.^26^ Interestingly, the volume of distribution (*V*_ss_) remained unchanged.^27^

Once efflux was highlighted as a concern for further compound optimization, both for preclinical PK optimization and for future clinical success against refractory ovarian cancer, we required a medium-throughput assay to rapidly determine efflux-mediated SAR. Cellular P-gp-mediated efflux acquired MDR to the cytotoxic anthracycline, doxorubicin is well established and can be used as both a P-gp efflux model and MDR-resistance model.^28^ We therefore proposed that doxorubicin resistance could be exploited to establish a surrogate assay for P-gp-mediated efflux with appropriate throughput in matched pair ovarian cancer cell lines. An acquired doxorubicin-resistant human cancer cell line, CH1^doxR^, was previously obtained in-house through exposure of the wild-type cell line CH1^WT^ to doxorubicin.^29^ After several passages, the CH1^doxR^ cell line demonstrated >100-fold resistance. The P-gp-dependent MDR properties of CH1^doxR^ were confirmed by the rescue of the antiproliferative activity of doxorubicin by cotreatment with the P-gp-inhibitor verapamil,^30^ resulting in a shift in GI_50_ in CH1^doxR^ from 310 to 1.9 nM, now within 5.0-fold of the antiproliferative activity observed in CH1^WT^ (Figure S4).

After demonstrating that the antiproliferative activity of the bisamide chemical probe **1** in CH1^doxR^ cells could also be increased by cotreatment with the P-gp inhibitor (Figure S4), we aimed to validate the use of CH1^WT^ and CH1^doxR^ cells as a viable approach to establish useful SAR by carrying out a screen of selected bisamide analogues (*N* = 43; Table S3). The comparative antiproliferative activity of each analogue against the CH1^WT^ and CH1^doxR^ cells was measured, and the fold-differences between their respective geometric mean GI_50_ values were used as a surrogate for their respective CH1^doxR^/CH1^WT^ P-gp-mediated efflux ratios.^31^ The statistical significance of the ratio was determined using Student’s *t*-test from the respective pGI_50_ values, and only ratios of analogues where *p* < 0.05 were considered to be P-gp-substrates. Using this approach, compound **16** gave a moderately high CH1^doxR^/CH1^WT^ ratio (8.4), consistent with its poor in vivo mouse PK profile. In contrast, the oxygen-linked piperidine analogue **17** displayed no significant difference in the comparison of their respective GI_50_ values (CH1^doxR^/CH1^WT^ = 1.7), indicating that this compound is likely only a weak P-gp substrate. Owing to its acceptable in vitro and wild-type Balb/c mouse in vivo profile (Table 2, entry 2), **17** was selected for in vivo study in P-gp-knockout mice (Figure S8). The PK data revealed that there was no significant difference in the CL_tb_ of **17** between P-gp-knockout and wild-type mice, consistent with our in vitro prediction from the CH1 cell-based assay.

Unfortunately, the in vitro cell-based MDR assay revealed no clear SAR or patterns relating to the physicochemical properties that are typically used to remove P-gp-mediated efflux.^32^ Critical structural features likely to be important for passive permeability, such as the two amide moieties, could not be replaced in a manner consistent with the cellular SAR to retain activity and there was no clear correlation with compound lipophilicity.^13^ To carry out the necessary optimization to improve compound PK profiles across multiple species, a general method to eliminate P-gp-efflux was needed. Levatic et al. have reported the empirical observation that molecular density is an important feature in P-gp-drug recognition and compounds with high specific volumes are less likely to suffer from P-gp-mediated efflux.^33^ We hypothesized that as halogens possess high van der Waals volumes,^34^ they could be used to reduce the molecular density of the bisamide chemotype and mitigate this liability (Table 3).

**Figure 2:**
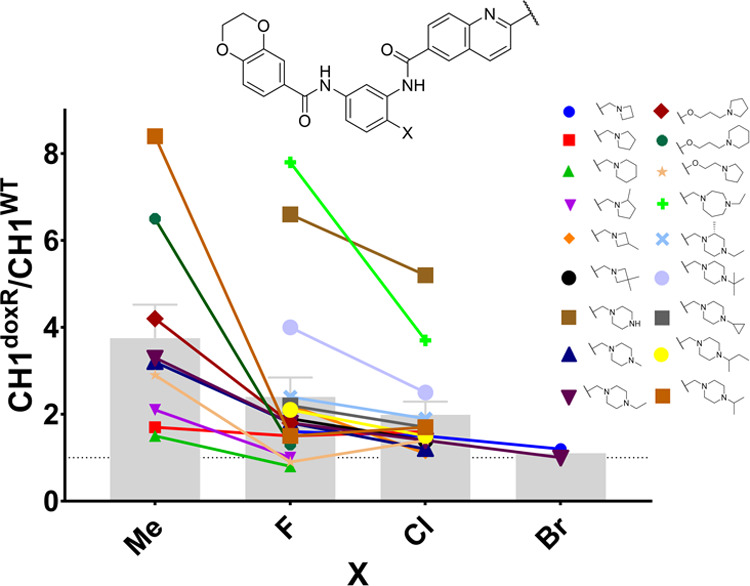
Matched molecular pair analysis on the effect of halogen substitution on multidrug resistance. The efflux ratio is calculated from the ratio of geometric mean GI_50_ values in the CH1^WT^ and CH1^doxR^ cell lines; each GI_50_ is calculated from at least *n* = 3 biological repeats. Each colored line represents an MMP varying only at the central ring substituent. The gray bars represent the arithmetic mean ± SEM of the grouped central ring substituents. The gray dotted line is the efflux ratio = 1. See Table S3 for details.

It was important that we introduced the halogen on the bisamide chemotype distal from the solubilizing group to allow for further orthogonal PK optimization. We hypothesized that the benzylic methyl on the central ring of the bisamide, which was critical to cellular activity of this chemotype, was suitable for substitution, as halogens have been shown to act as good bioisosteric replacements for small lipophilic groups.^35^ A matched molecular pair (MMP)^36^ of piperazine **16** was synthesized, replacing the methyl with chlorine to give **18** (Table 3, entry 2). Chlorobisamide **18** maintained excellent antiproliferative activity, and importantly, the efflux-mediated CH1^doxR^/CH1^WT^ ratio was reduced from 8.4-fold to 1.7-fold, with respect to **16**. To assess whether this effect was general to this chemotype, we synthesized halogenated MMPs of the compounds that exhibited significant efflux in the CH1^doxR^/CH1^WT^ assay (Table S3). In all cases, halogen substitution strongly reduced efflux compared to their respective methyl MMPs (Figure 2, colored lines) and by comparing the average effect of each halogen (Figure 2, gray bars). In particular, the larger halogens had an apparently greater impact (Br > Cl > F), consistent with their effect on molecular density or possibly through more efficient steric shielding of the proximal amide moiety, although the exact mechanism for the change in P-gp recognition with this chemotype remains unclear (Figure 2).^34,38^

### In Vitro/In Vivo Correlation Disconnection

Following our discovery of a general strategy to mitigate the P-gp-mediated efflux liability of the bisamide chemotype, we then sought to complete the multiparameter optimization necessary to deliver a clinical candidate. Further in vitro analysis of chlorobisamide **18** revealed that the compound possessed low MLM CL_int_ and good KS (Table 3, entry 2) and so was selected for an in vivo PK study in BALB/c mice (Table 2, entry 2). Chlorobisamide **18** displayed an impressive improvement in oral bioavailability (*F* = 63%, CL_tb_ = 33 mL/min/kg, extraction ratio = 37%, *F*_max_ = 63%), resulting from high absorption consistent with its in vitro property profile. However, **18** still displayed disappointingly high in vivo CL_u_ (3000 mL/min/kg, *f*_ub_ = 0.011), despite the predicted CL_u_ from the MLM being 51-fold lower. No significant degradation was observed following the incubation of bisamide analogues in mouse plasma, so plasma instability was considered unlikely to be contributing to the discrepancy (Table S7). From these data, it was clear that there was still another component contributing to the in vivo clearance that needed to be addressed.

### Hepatocyte Clearance

Microsomes can underpredict in vivo CL_u_ from either the loss of metabolic enzyme integrity during tissue handling. the effect of uptake transporters on intracellular free concentrations or under-representation of cytosolic enzymes and cofactors.^24^ Therefore, chlorobisamide **18** was screened in vitro using mouse hepatocytes (MHeps), which revealed a predicted in vivo CL_u_ now within 3.5-fold of the measured value.^21^ The improved correlation could be due to better representation of amide hydrolysis degradation pathways in hepatocytes compared to that in microsomes. The MHeps assay, combined with our CH1^doxR^/CH1^WT^ ratio assay, finally gave us an appropriate in vitro triage of compounds for in vivo PK assessment.

Although introducing the chlorine substituent had mitigated the P-gp-mediated efflux risk, it had also significantly increased the lipophilicity of **18** relative to its MMP, **16**, potentially leading to the increased in vivo CL_u_. To improve the metabolic stability of **18**, we aimed to decrease the lipophilicity of the chloro-series closer to the value obtained with methyl analogue **16**, which showed lower in vivo CL_u_. Removal of the *N-*alkyl moiety to afford the free piperazine **19** was not tolerated, resulting in a large CH1^WT^/CH1^doxR^ ratio for predicted P-gp-mediated efflux (Table 3, entry 3), while *N-*methylpiperazine **20** displayed high MLM CL_int_ (110 μL/min/mg) and so was not investigated further. However, *N-*ethylpiperazine **21** displayed a good balance of physicochemical properties (Table 3, entry 5), which translated into excellent mouse oral bioavailability (Table 2, entry 4) from moderate total blood clearance (CL_tb_ = 28 mL/min/kg, extraction ratio = 31%, *F*_max_ = 69%). Unfortunately, the high CL_u_ persisted and no other changes to the solubilizing group of the chloro-series were able to significantly improve the metabolic stability predicted from in vitro analysis (see Table S2 for details).

To further reduce lipophilicity, we hypothesized that we could replace the benzylic chlorine substituent with fluorine, but we were concerned that this decrease could have a detrimental effect on passive permeability. However, analysis of the Cambridge Structural Database of small molecules^37^ revealed that *ortho*-fluorobenzamides tend to adopt more planar conformations than their methyl counterparts (Figure S3). The amide–NH bond can eclipse the fluorine–carbon bond, forming a dipole–dipole interaction and masking the hydrogen bond donor, hence mitigating concerns of decreased lipophilicity on passive permeability.^38^ The fluorine MMP, CCT361814/NXP800 **22**, pleasingly displayed the desired reduction in lipophilicity (Table 3, entry 6), which correlated with reduced in vitro MLM (15 μL/min/mg) and mouse hepatocyte CL_int_; while maintaining excellent antiproliferative activity (free GI_50_ = 3.7 nM, *f*_ua_ = 0.43; Table S4)^39^ and acceptable KS (50 μM). Fluorobisamide **22** was therefore submitted for an in vivo mouse PK study (Table 4, entry 1).

**Table 4:** In Vivo Blood PK Profiles of **22** in Rodent and Nonrodent Species^a^

Entry	Species^b^	Dose po/iv (mg/kg)^e^	*T*_max_ (h)	po AUC^0–t^ (h*nM)	iv Cl_tb_ (mL/min/kg)^h^	*t*_1/2_ (h)^i^	*F* (%)^j^	*f*_ub_^k^	AUC_u_^0–t^ (h*nM)^l^	Free *C*_av_^0–24h^ (nm)^m^	iv Cl_u_ (mL/min/kg)^n^
1	mouse	5/5	2.0	6000 (7800–4600)^f^	10 (10–9.7)	4.0	42	0.012	72	3.3	830
2	rat^c^	5/1	6.0	2600^f^	24	3.1	45	0.033	86	3.7	730
3	dog^d^	2.5/0.5	2.0	250^g^	21	1.4	9.1^h^	0.14	35	1.9	150

a All values are quoted to two SFs and are the geometric mean of *n* = 3 individual animals unless otherwise stated. PK parameters are calculated from the blood concentration/time curve using noncompartmental analysis model 201 and 202 Phoenix version.6.1. The 90% confidence intervals are in parentheses.

b Immunocompetent BALB/c mice, SD rats, and beagle dogs.

c The rat blood PK was determined as a composite profile of six animals.

d Dog live phase was carried out at Charles River Laboratories; data are derived from the geometric mean of *n* = 4 individual dogs (two males and two females); PK parameters in the dog study were calculated from the plasma concentration/time curve and were converted to blood PK parameters using the blood-to-plasma ratio determined in vitro at Cyprotex.

e The po and iv dosing vehicles are described in the Supporting Information.

f *t* = 24 h.

g *t* = 6 h.

h CL_tb_ = total blood clearance.

i Terminal half-life calculated from the iv dose.

j Assumes linear PK.

k *f*_ub_ = fraction unbound in blood, *f*_ub_ = *f*_up_/B:P, *f*_up_ = fraction unbound in plasma, B:P = blood-to-plasma ratio, measured in vitro using dialysis and quoted as the geometric mean from *n* = 3 statistical repeats from pooled samples; see the Supporting Information for details.

l AUC_u_ = AUC*f_ub_.

m Free C_av_^0–24h^ = AUC^inf^/24*f_ub_.

n CL_u_ = CL_tb_/*f*_ub_.

The mouse in vivo CL_u_ for compound **22** was consistent with the predicted value from the MHeps assay and comparable to methyl analogue **16** (Table 2, entry 1). Despite the decreased lipophilicity, the CH1^doxR^/CH1^WT^-predicted P-gp-mediated efflux ratio was low and fluorobisamide **22** displayed good mouse oral bioavailability (42%) from moderate total blood clearance (CL_tb_ = 10 mL/min/kg, extraction ratio = 11%, *F*_max_ = 89%).^22^ Owing to these favorable data, fluorobisamide **22** was selected for evaluation of its in vivo efficacy against established SK-OV-3 human ovarian cancer solid tumor xenografts in athymic immunodeficient mice (Table 5).

**Table 5:**
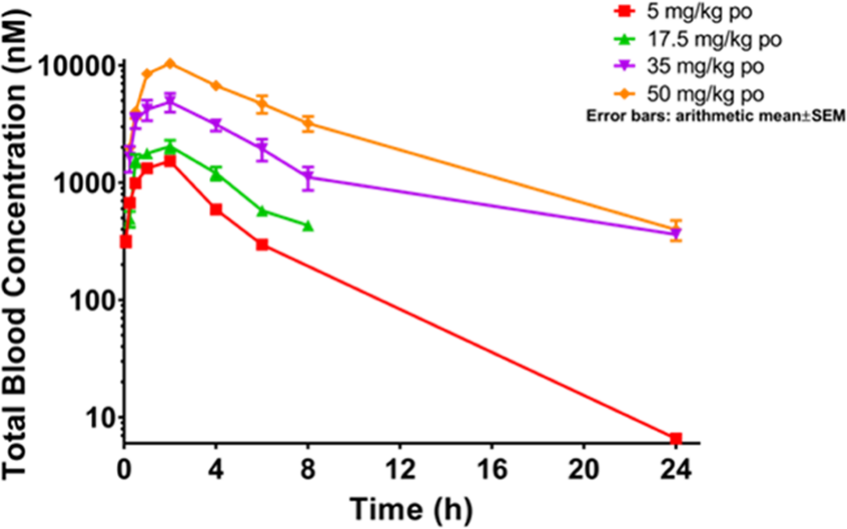
Blood Pharmacokinetic Profiles of Fluorobisamide **22** in Athymic Mice with Increasing Oral Dose^a^

Entry	Dose (mg/kg)^b^	*T*_max_ (h)	AUC^0–t^ (h*nM)	iv Cl_tb_ (mL/min/kg)^f^	*t*_1/2_ (h)^g^	*F* (%)^h^	*f*_ub_^i^	AUC_u_^0–t^ (h*nM)^j^	Free *C*_av_^0–24h^ (nM)^k^	iv CL_u_ (mL/min/kg)^l^
1	5 iv	NA	6700 (7800–5700)^c^	21 (24–18)	1.5	NA	0.0070	47	2.1	3000
2	5 po	1.7	1300 (1800–990)^c^			20		9.1	0.59	
3	17.5 po	1.7	9000 (12 000–7000)^d^			39		63	3.1	
4	35 po	2	34 000 (57 000–21 000)^d^			72		240	10	
5	50 po	1.7	78 000 (100 000–60 000)^e^			120		550	24	

a All graphs were plotted using GraphPad Prism Version 7.01. NA = not applicable. Each point on the PK curve is the arithmetic mean ± SEM of *n* = 3 individual animals. All values are quoted to two SFs and are the geometric mean of *n* = 3 individual mice. PK parameters are calculated from the blood concentration/time curve using noncompartmental analysis Phoenix version 6.1. The 90% confidence intervals are in parentheses.

b The po and iv dosing vehicles are described in the Supporting Information.

c *t* = 6 h.

d *t* = 8 h.

e *t* = 24 h.

f CL_tb_ = total blood clearance from the 5 mg/kg iv dose.

g Terminal half-life calculated from the iv dose.

h Assumes linear PK.

i *f*_ub_ = fraction unbound in blood, *f*_ub_ = *f*_up_/B:P, *f*_up_ = fraction unbound in plasma, B:P = blood-to-plasma ratio, measured in vitro using dialysis and quoted as the geometric mean from *n* = 3 statistical repeats from pooled samples; see the Supporting Information for details.

j AUC_u_ = AUC*f_ub_.

k Free *C*_av_^0–24h^ = AUC^inf^/24*f_ub_.

l CL_u_ = CL_tb_/*f*_ub_.

### Efficacy and PD

**Figure 3:**
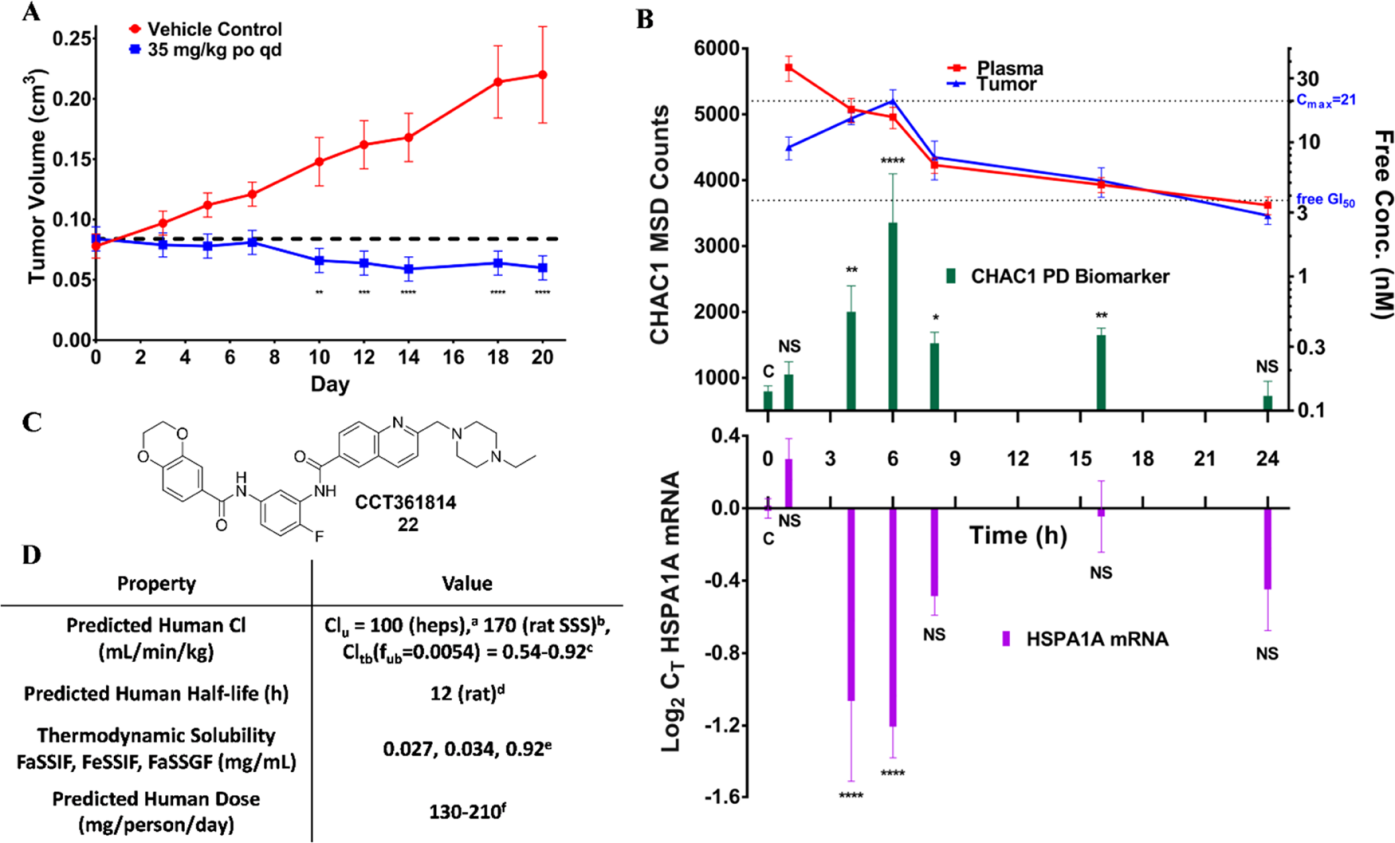
Antitumor, PK, and pharmacodynamic (PD) activity of fluorobisamide **22** in immunodeficient athymic mice. All data were analyzed and plotted using GraphPad Prism 7.01. NS *p* > 0.05, * *p* ≤ 0.05, ** *p* ≤ 0.01, *** *p* ≤ 0.001, **** *p* ≤ 0.0001. A: Antitumor activity of fluorobisamide **22** following a 35 mg/kg po qd dose against established SK-OV-3 human ovarian cancer xenografts in immunodeficient athymic mice. Each point represents the arithmetic mean ± SEM of *n* = 10 mice. Analysis by standard two-way ANOVA: interaction p<0.001, time p=0.0032, treatment p<0.0001. Comparison of vehicle vs treated arms at each time point using Sidak’s multiple comparison test. See the Supporting Information for details. B: PK/PD analysis following a single 50 mg/kg po dose in athymic mice with established SK-OV-3 xenografts. The CHAC1 and *HSPA1A* biomarker responses in the tumor were quantified using an MSD and quantitative polymerase chain reaction (qPCR) assays, respectively, and were correlated to the relative free concentration of **22** in both the plasma (*n* = 4) and the tumor (*n* = 4 except 4 and 16 h when *n* = 3). The free concentration in the plasma was estimated by *C*_u_ = *C*_T_*f_up_; *f*_up_ was measured in vitro using standard dialysis methods in pooled athymic mouse plasma. Tumor-free concentrations were estimated by calculating the free fraction in the tumor post distribution equilibrium (>8 h). The significance of all CHAC1 and *HSPA1A* biomarker responses in treated samples (CHAC1: *n* = 4 at each time point except 6 h where *n* = 3, *HSPA1A*: *n* = 4 at each time point except 4 and 6 h where *n* = 3) is described relative to the vehicle control (*n* = 23, 0–24 h) and was analyzed using a one-way ANOVA (CHAC1: *p* < 0.0001, *HSPA1A*: *p* < 0.0001) and Dunnett’s multiple comparison test of the log-transformed data. C: Structure of the clinical candidate fluorobisamide **22**. D: Clinical development candidate profile of fluorobisamide **22** with predicted human parameters; ^a^heps = human hepatocytes, CL_u_ calculated from in vitro CL_int_ using scaling factors and the well-stirred model; ^b^rat SSS = rat single-species scaling (SSS) CL_u_^human^ = CL_u_^rat^ (body weight^human^/body weight^rat^) ^∧^ 0.75; ^c^CL_tb_ = CL_u_*f_ub_, *f*_ub_ = *f*_up_/B:P, *f*_up_ and B:P were determined in vitro by dialysis of pooled plasma and blood samples; ^d^determined from the rat *t*_1/2_ using an empirical approach log *t*_1/2_(human) = 0.906*log *t*_1/2_(rat) + 0.723; ^e^measured at Pharmidex, the dynamic range of the assay is 0–1 mg/mL, FaSSIIF and FeSSIF pH6.5, FaSSGF pH1.6; ^f^estimated from *C*_av(free)_ = 10 nM in the mouse model, dose = (*C*_eff(free)_*CL_u_*τ)/*F*, where τ = dose frequency (24 h) and *F* is assumed to be 0.4.

The assessment of the blood PK profiles of fluorobisamide **22** in immunodeficient athymic mice revealed a much higher CL_u_ (3000 mL/min/kg) following an iv dose than had been observed in immunocompetent BALB/c mice, consistent with our previous observations with chemical probe **1**,^13^ and overproportional exposure with increasing po dose. Owing to the nonlinear PK, high CL_u_ in this mouse strain, and following a multidose tolerability study (Figure S9), a 35 mg/kg po qd dose was selected for the efficacy study, which should give a 2.7-fold coverage (AUC_u_^0–24h^ = 240 h*nM, free C_av_^0–24h^ = 10 nM) of the in vitro free GI_50_ (Figure 3A).

The mice maintained acceptable condition and body weight (Figure S10; <10% loss) when dosed at 35 mg/kg po continuously with a solution of fluorobisamide **22** for 20 days without the need for dose breaks. Excellent efficacy was observed against established SK-OV-3 human ovarian cancer solid tumor xenografts grown subcutaneously, with tumor growth inhibition (TGI) of 120% relative to control^40^ and 8 out of 10 tumors displaying regression. *T*/*C* values, based on the final mean tumor weights, also showed a significant reduction to 37% of control (*p* = 0.0008, Student’s *t*-test; Figure S11).

Discovering pharmacodynamic (PD) biomarkers using compounds developed from phenotypic screens of transcription inhibitors can be challenging, as to observe an effect the biological pathways of interest must commonly be potentiated by an external stimulus.^41a^ In vitro, we utilized as a PD biomarker the blocked induction of the protein product HSP72, which is encoded by the canonical HSF1-regulated gene *HSPA1A* in human cancer cell lines, following activation with the HSP90 inhibitor 17-AAG, to confirm that fluorobisamide **22** antagonized the pathway (SK-OV-3, HSP72 cell-based ELISA, pIC_50_ = 7.03 ± 0.07, IC_50_ = 94 nM, *n* = 40).^42^ However, for in vivo studies, we required protein PD biomarkers that would not need an HSP90 inhibitor to activate HSF1. Also, modulation at the mRNA rather than protein level would provide more proximal biomarkers for the inhibition of HSF1-mediated transcription.^43^ Therefore, we carried out gene expression profiling of cancer cell lines and tumor xenograft tissues following treatment with fluorobisamide **22**; this demonstrated the inhibition of heat shock response gene signature and activation of the related integrated stress response signature, which we then exploited for PD biomarker development.^44−46^

qPCR analysis of the end-of-study tumor samples from the treated and untreated arms of the efficacy study revealed a clear increased expression of the mRNA of *CHAC1* (Figure S12), a gene involved in the integrated stress response and activation of which would be consistent with the inhibition of the HSF1 stress pathway.^45^*CHAC1* is known to be downstream of the HSF1-regulated gene, *ATF3.*(10,44) At the protein level, the induction of CHAC1 was confirmed by immunoblot, from both in vivo tumor samples and in vitro data in SK-OV-3 cells treated with fluorobisamide **22** (19 nM, 5 × free GI_50_; Figure S13).^47^

To investigate the relationship between the free exposure of **22** and HSF1 pathway inhibition PD biomarker modulation, a single dose (50 mg/kg), single agent PK/PD study was designed and carried out on athymic mice bearing established SK-OV-3 human ovarian cancer solid subcutaneous tumor xenografts, as were used in the efficacy study (Figure 3B). The PK/PD data revealed a tumor *T*_max_ at 6 h, 4 h later than the dose escalation study blood *T*_max_ in nontumor-bearing mice.^48^ Distribution equilibrium was achieved after 8 h, and by comparison with free plasma concentrations at these later time points, we estimated the free tumor concentrations^49^ to achieve a tumor-free *C*_max_ = 21 nM and free *C*_min_ = 2.8 nM after 24 h. At the dose of fluorobisamide **22** used, free tumor concentrations were greater than the in vitro free GI_50_ for 21 h. CHAC1 protein expression was confirmed using immunoblotting (Figure S14) and was quantified by an MSD assay we developed in-house^50^ (Figure S15). In vivo, CHAC1 induction by fluorobisamide **22** correlated well with the free concentration in the tumor, with CHAC1 induction *T*_max_ (PD) also occurring at the fluorobisamide **22** tumor *T*_max_ (PK) and at a free concentration comparable to those for the in vitro induction (Figure 3B).

To assess a more direct biomarker for the antagonism of HSF1-mediated transcription, we turned to the inhibition of expression of the canonical heat shock *HSPA1A* gene, which encodes the HSP72 protein. As expected, given its long degradation half-life,^43^ no clear changes to basal HSP72 protein levels were observed over the 24 h of the PK/PD study. However, analysis of the short half-life *HSPA1A* mRNA tumor levels using qPCR revealed significant depletion by fluorobisamide **22** that also correlated well with the tumor-free concentrations (Figure 3B).

### Clinical Candidate

The optimized fluorobisamide **22** clearly displayed improved mouse PK and efficacy in the SK-OV-3 human ovarian xenograft model compared to our earlier chemical probe **1**. Screening **22** against our panel of genetically diverse human ovarian cancer cell lines demonstrated that the compound retained excellent antiproliferative activity (Table S1). To further assess the potential of **22** as a clinical candidate, the compound was assayed in the Cerep in vitro safety screen of 87 potentially high-risk off-target proteins (Table S9).^51^ From this screen, only adenosine A2A receptor antagonism (IC_50_ = 2.0 μM; Figure S4) was confirmed by a functional assay but at a value ∼100-fold higher than the efficacious free concentrations.^52^ Fluorobisamide **22** also displayed no hERG (IC_50_ > 30 μM)^53^ or CYP (IC_50_ > 10 μM) inhibition (Table S10 and Figure S7) liability and was clean in kinase screening panels (data not shown), consistent with our previous analysis.^13^ Analysis of rat PK data for **22** (Table 4, entry 2) revealed a clear improvement with respect to the lead compound **1**, with reduced CL_u_, consistent with the rat hepatocyte prediction of 410 mL/min/kg, good oral bioavailability (45%) from a moderate total blood clearance (CL_tb_ = 24 mL/min/kg, extraction ratio = 34%, *F*_max_ = 66%),^17,57^ and acceptable half-life (*t*_1/2_) (Table 4, entry 2, and Table S12).

To select a higher species for further PK study, **22** was submitted to minipig and dog hepatocyte assays (Table S6). The minipig hepatocyte CL_int_ (860 μL/min/10^6^ cells) was very high, consistent with a previous study, which showed that the bisaryl amide motif found in the bisamide chemotype is particularly susceptible to hydrolysis by minipig liver amidases.^54^ Given the confidence we had gained in the predictive value of in vitro hepatocyte metabolism for in vivo CL_u_ prediction, minipig was not considered further. The dog hepatocytes gave CL_int_ = 31 μL/min/10^6^ cells, which predicted an in vivo CL_u_ = 96 mL/min/kg. However, in contrast to the very low blood-free fraction (*f*_ub_; Table S4) of **22** in mice, rats, and humans (0.0053–0.033), the dog blood-free fraction was surprisingly high (*f*_ub_ = 0.14). Despite this contrast, the dog was selected as our higher species for further study (Table 4, entry 3).^55^ The dog PK data revealed a lower CL_u_ compared to the rodent species and were consistent with the hepatocyte prediction, but oral bioavailability (9.1%) was also low, from a moderate total blood clearance (CL_tb_ = 21 mL/min/kg, extraction ratio = 52%, *F*_max_ = 48%).^56,57^ The low absorption in the dog compared to that in the rat for **22** could be due to solubility-limited absorption of this basic compound in the relatively high pH of the dog’s stomach.^58^ Nevertheless, the free exposure from the 2.5 mg/kg po dose in the dog was still comparable to the efficacy study free exposure due to the much lower CL_u_ (AUC_u_^0–6h^ = 40 h*nM, free *C*_av_^0–6h^ = 6.6 nM).

Furthermore, **22** displayed improved in vitro permeability in the Caco-2 assay (*A*–*B* = 7.7 × 10^–6^ cm/s, efflux ratio = 2.8; Table S8), despite low compound recovery (∼50%, consistent with intracellular compound retention), which can limit passive permeability.^59^ Thermodynamic solubility assays in simulated human biorelevant simulated fluids showed that compound **22** (Figure 3D) was highly soluble in simulated gastric fluid, possibly due to the low pH, and modestly soluble in intestinal fluid (Table S11).^60^ From our studies and the excellent efficacy in the SK-OV-3 human ovarian xenograft model driven by a low free concentration, the calculated dose predictions for **22** to human based on the efficacious AUC_u_^0–24h^ were favorable, using both single-species scaling (SSS)^61^ and scaling from human hepatocytes, at less than 210 mg/person/day.^62^ These data led to the nomination of fluorobisamide **22** as our clinical candidate (Figure 3).^63^

## Conclusions

We carried out multiparameter lead optimization of the bisamide chemical probe **1**, discovered from an HSF1 stress pathway inhibitor phenotypic screen, using cell-based SAR to maintain the excellent antiproliferative activity. During our PK optimization, minimizing P-gp-mediated efflux became an early focus. We developed a medium-throughput cell-based antiproliferation sensitivity assay as a surrogate to assess P-gp-mediated efflux and demonstrated that incorporating halogens into our analogue design reduced this liability in all examples across a wide range of substrates. This led to an empirical but effective strategy to mitigate P-gp-mediated efflux that could potentially be applicable to other chemotypes. A further multiparameter optimization gave us our clinical candidate, fluorobisamide **22**. This compound displayed a good PK profile across different species and excellent therapeutic efficacy, including tumor regression, from a low free exposure in an in vivo human ovarian cancer xenograft mouse model, and demonstrated biomarker modulation in tumor tissue consistent with the HSF1 pathway inhibition—representing overall a strong pharmacological audit trail.^41^

We have carried out numerous studies to determine the molecular mechanism of action of fluorobisamde **22**, including transcriptional profiling and use of multiple orthogonal chemoproteomic technologies. Demonstration of the increased expression of *CHAC1* mRNA and the reduced expression of *HSPA1A* mRNA, which represent useful PD markers, is consistent with the activation of the integrated stress response and inhibition of HSF1-mediated transcription. Further mechanistic follow-up studies are underway.

Following successful preclinical development, CCT361814/NXP800 **22** entered phase 1 clinical trial (NCT05226507) in cancer patients in 2022 as a potential future treatment for refractory ovarian cancer and other malignancies.^64^

## Experimental Section

All experiments using animals were performed in accordance with the local Animal Welfare and Ethical Review Board, the U.K. Home Office Animals Scientific Procedures Act 1986, and the U.K. National Cancer Research Institute Guidelines for the Welfare of Animals in Cancer Research.^65^ The ICR does not undertake research in nonrodent species and requires internal ethical review when such studies are sponsored by organizations with whom we collaborate. Collaborator-sponsored nonrodent pharmacology studies of compound **22** necessary for the prediction of therapeutic window and application to the clinic were approved by the ICR Animal Welfare and Ethics Review Board and were conducted in full compliance with national regulations at AAALAC accredited R&D sites.

### General Procedures (Chemistry)

All final compounds were screened through our in-house computational PAINS filter and gave no structural alerts as potential assay interference compounds.^66^ Unless otherwise stated, reactions were conducted in oven-dried glassware under an atmosphere of nitrogen or argon using anhydrous solvents. All commercially obtained reagents and solvents were used as received. Thin-layer chromatography (TLC) was performed on precoated aluminum sheets of silica (60 F254 nm, Merck) and visualized using short-wave UV light. Flash column chromatography was carried out on Merck silica gel 60 (particle size 40–65 μm). Column chromatography was also performed on Biotage SP1 or Isolera 4 purification systems using Biotage Flash silica cartridges (SNAP KP-Sil). Ion-exchange chromatography was performed using acidic Biotage Isolute Flash SCX-2 columns. All compounds are >95% pure by HPLC analysis. HPLC traces of the clinical candidate **22** and all in vivo compounds are included in the Supporting Information.

#### Semipreparative HPLC

500 μL standard injections (with needle wash) of the sample were made on a Phenomenex Gemini C18 column (5 μ, 250 mm × 21.2 mm, Phenomenex, Torrence). Chromatographic separation at room temperature was carried out using a 1200 Series Preparative HPLC (Agilent, USA) over a 15 min gradient elution from 90:10 to 0:100 water:methanol (both modified with 0.1% formic acid) at a flow rate of 20 mL/min. UV–vis spectra were acquired at 254 nm on a 1200 Series Prep Scale diode array detector (Agilent). Post-UV and pre-MS splitting were achieved using an Active Split (Agilent) before being infused into a 6120 Series Quad mass spectrometer fitted with an ESI/APCI Multimode ionization source (Agilent). Collection was triggered by UV signal and collected on a 1200 Series Fraction Collector (Agilent). ^1^H NMR spectra were recorded on Bruker Avance 500 (500 MHz) spectrometers using an internal deuterium lock. Chemical shifts are quoted in parts per million (ppm) using the following internal references: CDCl_3_ (δH 7.26), MeOD (δH 3.31), and DMSO-*d*_6_ (δH 2.50). Signal multiplicities are recorded as singlet (s), doublet (d), triplet (t), quartet (q), quintet (qn), and multiplet (m), doublet of doublets (dd), doublet of doublet of doublets (ddd), broad (br), obscured (obs) or apparent (app). Coupling constants, *J*, are measured to the nearest 0.1 Hz. ^13^C NMR spectra were recorded on Bruker Avance 500 spectrometers at 126 MHz using an internal deuterium lock. Chemical shifts are quoted to 0.01 ppm, unless greater accuracy was required, using the following internal references: CDCl_3_ (δC 77.0), MeOD (δC 49.0), and DMSO-*d*_6_ (δC 39.5). High-resolution mass spectra were recorded on an Agilent 1200 series HPLC and a diode array detector coupled to a 6210 time-of-flight mass spectrometer with a dual multimode APCI/ESI source (methods I–IV) or on a Waters Acquity UPLC and a diode array detector coupled to a Waters G2 QToF mass spectrometer fitted with a multimode ESI/APCI source (methods V–VI). Analytical separation was carried out according to the methods listed below. The mobile phase was a mixture of methanol (solvent A) and water (solvent B), both containing formic acid at 0.1%; UV detection was at 254 nm.

Method I: analytical separation was carried out at 30 °C on a Merck Purospher STAR column (RP-18e, 30 mm × 4 mm) using a flow rate of 1.5 mL/min in a 4 min gradient elution. Gradient elution was as follows: 10:90 (*A*/*B*) to 90:10 (*A*/*B*) over 2.5 min, 90:10 (*A*/*B*) for 1 min, and then reversion back to 10:90 (*A*/*B*) over 0.3 min, finally 10:90 (*A*/*B*) for 0.2 min. Method II: analytical separation was carried out at 30 °C on a Merck chromolith flash column (RP-18e, 25 mm × 2 mm) using a flow rate of 0.75 mL/min in a 4 min gradient elution. Gradient elution was as follows: 5:95 (*A*/*B*) to 100:0 (*A*/*B*) over 2.5 min, 100:0 (*A*/*B*) for 1 min, and then reversion back to 5:95 (*A*/*B*) over 0.1 min, finally 5:95 (*A*/*B*) for 0.4 min. Method III: analytical separation was carried out at 40 °C on a Merck Purospher STAR column (RP-18e, 30 mm × 4 mm) using a flow rate of 3 mL/min in a 2 min gradient elution. Gradient elution was as follows: 10:90 (*A*/*B*) to 90:10 (*A*/*B*) over 1.25 min, 90:10 (*A*/*B*) for 0.5 min, and then reversion back to 10:90 (*A*/*B*) over 0.15 min, finally 10:90 (*A*/*B*) for 0.1 min. Method IV: analytical separation was carried out at 40 °C on a Merck Purospher STAR column (RP-18e, 30 mm × 4 mm) using a flow rate of 1.5 mL/min in a 2 min gradient elution. Gradient elution was as follows: 5:95 (*A*/*B*) to 100:0 (*A*/*B*) over 1.25 min, 100:0 (*A*/*B*) for 0.5 min, and then reversion back to 5:95 (*A*/*B*) over 0.05 min, finally 5:95 (*A*/*B*) for 0.2 min. Method V: Waters Acquity UPLC, Phenomenex Kinetex XB-C18 column (30 mm × 2.1 mm, 1.7 μ, 100 Å) at 30 °C using a flow rate of 0.3 mL/min in a 4 min gradient elution. Gradient elution was as follows: 10:90 (*A*/*B*) to 90:10 (*A*/*B*) over 3 min, 90:10 (*A*/*B*) for 0.5 min, and then reversion back to 10:90 (*A*/*B*) over 0.3 min, finally 10:90 (*A*/*B*) for 0.2 min. Method VI: Waters Acquity UPLC, Phenomenex Kinetex C18 column (30 mm × 2.1 mm, 2.6 μ, 100 Å), flow rate and gradient elution according to Method V. The following reference masses were used for HRMS analysis: Agilent 1200 series: caffeine [M + H]^+^ 195.087652; hexakis(1*H*,1*H*,3*H*-tetrafluoropentoxy)phosphazene [M + H]^+^ 922.009798 and hexakis(2,2-difluoroethoxy)phosphazene [M + H]^+^ 622.02896 or reserpine [M + H]^+^ 609.280657; Waters Acquity UPLC: Leucine Enkephalin fragment ion [M + H]^+^ 397.1876. All compounds were >95% purity by liquid chromatography-mass spectrometry (LCMS) analysis unless otherwise stated.

#### Synthetic Route I

##### *N*-(3-Amino-4-methylphenyl)-2,3-dihydrobenzo[*b*][1,4]dioxine-6-carboxamide

Oxalyl chloride (1.40 mL, 16.6 mmol) was added dropwise to a solution of 1,4-benzodioxane-6-carboxylic acid (2.49 g, 13.8 mmol) and DMF (0.027 mL, 0.340 mmol) in anhydrous dichloromethane (DCM) (34 mL). The reaction mixture was stirred at room temperature for 3.5 h and then concentrated. The residue was dissolved in DCM and concentrated again. This residue was dissolved in anhydrous DCM (12 mL) and added dropwise to a solution of 4-methyl-3-nitroaniline (2.10 g, 13.8 mmol) and pyridine (2.23 mL, 27.6 mmol) in anhydrous DCM (25 mL). The reaction mixture was stirred at room temperature for 2 h and then concentrated. The resulting solid was suspended in MeOH, diluted with water, and then isolated by filtration and washed with water to afford the title compound (4.24 g, 98%) as a pale tan-colored amorphous solid. ^1^H NMR (500 MHz, DMSO*-d*_6_) δ 10.39 (s, 1H), 8.54 (d, *J* = 2.2 Hz, 1H), 7.99 (dd, *J* = 8.4, 2.3 Hz, 1H), 7.55 (d, *J* = 2.1 Hz, 1H), 7.52 (dd, *J* = 8.4, 2.2 Hz, 1H), 7.47 (dd, *J* = 8.4, 0.8 Hz, 1H), 7.01 (d, *J* = 8.4 Hz, 1H), 4.34–4.29 (m, 4H), 2.49 (s, 3H). HRMS (ESI^+^): calcd for C_16_H_15_N_2_O_5_ (M + H)^+^, 315.0976; found 315.0982.

Palladium (10% on activated carbon, 0.567 g, 5.33 mmol) was added to a suspension of *N-*(4-methyl-3-nitrophenyl)-2,3-dihydrobenzo[*b*][1,4]dioxine-6-carboxamide (4.24 g, 13.5 mmol) in ethanol (90 mL) and ethyl acetate (90 mL). The reaction mixture was stirred under hydrogen (1 atm) at 28 °C overnight, filtered through celite with EtOAc, and concentrated to afford the title compound (3.80 g, 99%) as a pale yellow amorphous solid. ^1^H NMR (500 MHz, DMSO*-d*_6_) δ 9.70 (s, 1H), 7.49 (d, *J* = 2.2 Hz, 1H), 7.46 (dd, *J* = 8.3, 2.2 Hz, 1H), 7.10 (d, *J* = 2.0 Hz, 1H), 6.95 (d, *J* = 8.4 Hz, 1H), 6.83 (d, *J* = 8.1 Hz, 1H), 6.79 (dd, *J* = 8.1, 2.0 Hz, 1H), 4.81 (s, 2H), 4.32–4.26 (m, 4H), 2.01 (s, 3H). HRMS (ESI^+^): calcd for C_16_H_17_N_2_O_3_ (M + H)^+^, 285.1234; found 285.1233.

##### Methyl 2-Formylquinoline-6-carboxylate

To a solution of 2-methylquinoline-6-carboxylic acid (3.00 g, 16.0 mmol) in anhydrous MeOH (40 mL) under argon at room temperature, 4 M HCl in 1,4-dioxane (16.0 mL, 64.1 mmol) was added dropwise and the resulting mixture was heated at 85 °C for 4 h. Then, the reaction mixture was allowed to cool to room temperature, concentrated under reduced pressure, diluted with EtOAc (40 mL), and washed with 1 M NaOH (2 × 40 mL), water (1 × 40 mL), and brine (1 × 40 mL). The organic layer was dried over MgSO_4_, filtered, and concentrated under reduced pressure to afford a light tan-colored solid as a crude product, which was carried onto the next step without purification (2.36 g, 73%). ^1^H NMR (500 MHz, CDCl_3_) δ 8.54 (d, *J* = 1.9 Hz, 1H), 8.27 (dd, *J* = 8.8, 1.9 Hz, 1H), 8.16–8.12 (m, 1H), 8.04 (dt, *J* = 8.8, 0.7 Hz, 1H), 7.35 (d, *J* = 8.4 Hz, 1H), 3.98 (s, 3H), 2.77 (s, 3H). HRMS (ESI^+^): calcd for C_12_H_12_NO_2_ (M + H)^+^, 202.0868; found 202.0863.

To a suspension of selenium dioxide (0.873 g, 7.87 mmol) in anhydrous 1,4-dioxane (11 mL) under argon at room temperature, methyl 2-methylquinoline-6-carboxylate (1.44 g, 7.16 mmol) was added in one portion and the resulting suspension was allowed to stir at 80 °C for 18 h. The reaction was allowed to cool to room temperature, filtered through celite, and concentrated under vacuo to afford an orange solid as a crude product, which was purified by column chromatography on silica gel using a gradient of 10–20% EtOAc in petroleum ether to afford the clean product as a pale yellow amorphous solid (1.28 g, 83%). ^1^H NMR (500 MHz, CDCl_3_) δ 10.26 (d, *J* = 0.6 Hz, 1H), 8.68 (d, *J* = 1.6 Hz, 1H), 8.45 (d, *J* = 8.7 Hz, 1H), 8.42 (dd, *J* = 8.7, 1.6 Hz, 1H), 8.32 (d, *J* = 8.7 Hz, 1H), 8.11 (d, *J* = 8.2 Hz, 1H), 4.04 (s, 3H). HRMS (ESI^+^): calcd for C_12_H_10_NO_3_ (M + H)^+^, 216.0660; found 216.0658.

##### *N*-(5-(2,3-Dihydrobenzo[*b*][1,4]dioxine-6-carboxamido)-2-methylphenyl)-2-(pyrrolidin-1-ylmethyl)quinoline-6-carboxamide (**11**)

Pyrrolidine (0.144 mL, 1.74 mmol) was added to a suspension of methyl 2-formylquinoline-6-carboxylate (0.250 g, 1.16 mmol) in anhydrous DCM (5 mL). The reaction mixture was allowed to stir at room temperature for 6 h. Then, sodium triacetoxyborohydride (0.369 g, 1.74 mmol) was added in one portion and the reaction mixture was stirred overnight at room temperature. The reaction mixture was diluted with DCM (5 mL) and washed with NaHCO_3_ saturated aqueous solution (1 × 10 mL). The two layers were separated, and the aqueous phase was extracted with DCM (1 × 10 mL). The organic layer was dried over MgSO_4_, filtered, and concentrated under reduced pressure. The residue was purified by column chromatography using a gradient of 2–5% MeOH in DCM to afford the title compound as a light brown amorphous solid (225 mg, 71%). ^1^H NMR (500 MHz, CDCl_3_) δ 8.58 (d, *J* = 1.9 Hz, 1H), 8.32–8.24 (m, 2H), 8.10 (d, *J* = 8.8 Hz, 1H), 7.85 (br s, 1H), 4.14 (br s, 2H), 4.00 (s, 3H), 2.83 (br s, 4H), 1.94 (br s, 4H). HRMS (ESI^+^): calcd for C_16_H_19_N_2_O_2_ (M + H)^+^, 271.1441; found 271.1444.

Aqueous NaOH solution (1.02 M) (2.29 mL, 2.33 mmol) was added to a solution of methyl 2-(pyrrolidin-1-ylmethyl)quinoline-6-carboxylate (0.210 g, 0.777 mmol) in tetrahydrofuran (THF, 3 mL), followed by MeOH (1 mL) to ensure a homogeneous solution. The reaction mixture was stirred at room temperature overnight. Then, the reaction mixture was heated to 35 °C and a further 1.25 mL of NaOH aqueous solution (1.02 M) was added and the reaction mixture was allowed to stir overnight. The reaction mixture was concentrated to remove THF and MeOH. The remaining aqueous layer was washed with EtOAc (1 × 5 mL) and acidified to pH 3 with 2 M aqueous HCl. A precipitate was formed and filtered off. The filtrate was then concentrated to dryness to afford the title compound as a brown solid, which was carried onto the next step without purification (630 mg, contains NaCl, quantitative yield assumed for the next synthetic step). LCMS (ESI^+^): *t*_R_ = 1.42 min, *m*/*z* = 257 (M + H)^+^.

2-(7-Aza-1*H*-benzotriazole-1-yl)-1,1,3,3-tetramethyluronium hexafluorophosphate (HATU) (0.323 g, 0.850 mmol) was added to a solution of 2-(pyrrolidin-1-ylmethyl)quinoline-6-carboxylic acid (0.199 g, 0.680 mmol) and *N,N-*diisopropylethylamine (0.594 mL, 3.40 mmol) in anhydrous DMF (4 mL). The reaction mixture was stirred for 5 min before *N-*(3-amino-4-methylphenyl)-2,3-dihydrobenzo[*b*][1,4]dioxine-6-carboxamide (0.193 g, 0.680 mmol) was added. The reaction mixture was allowed to stir at room temperature overnight. Then, a further portion of *N,N-*diisopropylethylamine (263 μL) and HATU (258 mg) was added and the resulting mixture was allowed to stir for 6 h. The reaction mixture was diluted with water (8 mL), and the resulting precipitate was isolated by filtration and washed with water. The residue was purified by column chromatography using a gradient of 5–12% MeOH in DCM to afford 65 mg of a semicrude product as an orange-brown solid. Repurification by semipreparative HPLC afforded the title compound as a pale yellow amorphous solid (27 mg, 6.7% over two steps). ^1^H NMR (500 MHz, DMSO-*d*_6_) δ 10.15 (s, 1H), 10.08 (s, 1H), 8.63 (d, *J* = 2.0 Hz, 1H), 8.48 (d, *J* = 8.5 Hz, 1H), 8.26 (dd, *J* = 8.8, 2.1 Hz, 1H), 8.16 (s, 1H), 8.08 (d, *J* = 8.8 Hz, 1H), 7.88 (d, *J* = 2.2 Hz, 1H), 7.72 (d, *J* = 8.5 Hz, 1H), 7.59 (dd, *J* = 8.2, 2.2 Hz, 1H), 7.54 (d, *J* = 2.1 Hz, 1H), 7.51 (dd, *J* = 8.5, 2.2 Hz, 1H), 7.25 (d, *J* = 8.4 Hz, 1H), 6.98 (d, *J* = 8.4 Hz, 1H), 4.34–4.27 (m, 4H), 3.94 (s, 2H), 2.61–2.52 (m, 4H), 2.25 (s, 3H), 1.77–1.73 (m, 4H) (formic acid salt). ^13^C NMR (126 MHz, DMSO-*d*_6_) δ 165.39, 164.82, 163.84, 162.24, 148.59, 146.81, 143.39, 137.96, 137.78, 136.69, 132.39, 130.60, 129.22, 129.16, 128.64, 128.45, 128.14, 126.67, 122.22, 121.66, 119.06, 118.65, 117.30, 117.12, 65.37, 64.86, 64.48, 62.29, 54.29, 23.74, 17.94. HRMS (ESI^+^): calcd for C_31_H_31_N_4_O_4_ (M + H)^+^, 523.2340; found 523.2342.

##### Racemic-*N*-(5-(2,3-Dihydrobenzo[*b*][1,4]dioxine-6-carboxamido)-2-methylphenyl)-2-((2-methylpyrrolidin-1-yl)methyl)quinoline-6-carboxamide (**13**)

To a solution of methyl 2-formylquinoline-6-carboxylate (150 mg, 0.697 mmol) in anhydrous DCM (7 mL), 2-methylpyrrolidine (0.213 mL, 2.09 mmol) was added dropwise at room temperature and the resulting mixture was stirred for 2.5 h. Then, sodium triacetoxyborohydride (443 mg, 2.09 mmol) was added in one portion and the resulting mixture was stirred overnight at room temperature. The reaction mixture was diluted with DCM (10 mL) and washed with a NaHCO_3_ saturated aqueous solution (20 mL). The aqueous phase was extracted with DCM (3 × 10 mL), and the combined organic layers were dried over Na_2_SO_4_, filtered, and concentrated under reduced pressure to afford a yellow oil as a crude product, which was carried onto the next step without purification (194 mg). ^1^H NMR (500 MHz, CDCl_3_) δ 8.51 (d, *J* = 2.0 Hz, 1H), 8.22 (dd, *J* = 8.8, 2.0 Hz, 1H), 8.19–8.12 (m, 1H), 8.05 (dt, *J* = 8.9, 0.8 Hz, 1H), 7.67 (d, *J* = 8.5 Hz, 1H), 4.23 (d, *J* = 14.1 Hz, 1H), 3.94 (s, 3H), 3.57 (d, *J* = 14.1 Hz, 1H), 2.99–2.91 (m, 1H), 2.61–2.50 (m, 1H), 2.27 (q, *J* = 8.9 Hz, 1H), 2.07–1.86 (m, 1H), 1.79–1.57 (m, 2H), 1.45 (dddd, *J* = 12.5, 10.7, 8.5, 6.1 Hz, 1H), 1.13 (d, *J* = 6.0 Hz, 3H). LCMS (ESI^+^): *t*_R_ = 0.88 min, *m*/*z* = 285, (M + H)^+^.

To a solution of methyl 2-((2-methylpyrrolidin-1-yl)methyl)quinoline-6-carboxylate (194 mg, 0.682 mmol) in anhydrous THF (3.2 mL), 2 M aqueous NaOH solution (1.70 mL, 3.41 mmol) was added dropwise and MeOH (1.3 mL) was added to increase the miscibility of the two layers. The resulting red/brown solution was allowed to stir at 20 °C for 3 h. The reaction mixture was concentrated under reduced pressure, and the remaining aqueous layer was acidified to pH 3 with 1 M aqueous HCl and then washed with EtOAc (1 × 5 mL). The organic phase was discarded, and the aqueous phase was concentrated under reduced pressure to afford a beige amorphous solid as a crude product, which was carried onto the next step without purification. HRMS (ESI^+^): calcd for C_16_H_19_N_2_O_2_ (M + H)^+^, 272.1472; found 272.1468.

To a solution of 2-(7-aza-1*H*-benzotriazole-1-yl)-1,1,3,3-tetramethyluronium hexafluorophosphate (HATU) (176 mg, 0.462 mmol), 2-((2-methylpyrrolidin-1-yl)methyl)quinoline-6-carboxylic acid hydrochloride (100 mg, 0.326 mmol) in anhydrous DMF (2.5 mL) with *N,N-*diisopropylethylamine (0.322 mL, 1.85 mmol), *N-*(3-amino-4-methylphenyl)-2,3-dihydrobenzo[*b*][1,4]dioxine-6-carboxamide (105 mg, 0.370 mmol) was added in one portion and the resulting mixture was allowed to stir at 20 °C under an inert argon atmosphere for 18 h. The reaction mixture was poured onto water to afford a light brown precipitate, which was washed with water. The crude product was purified by column chromatography using a gradient of 0–20% EtOAc in DCM. A second purification by column chromatography using a gradient of 0–10% MeOH in DCM + 1% NH_3_ in MeOH afforded the title compound as a light brown amorphous solid (24 mg, ∼12%). ^1^H NMR (500 MHz, MeOD) δ 8.69–8.55 (m, 1H), 8.49 (d, *J* = 8.5 Hz, 1H), 8.31 (dd, *J* = 8.8, 1.8 Hz, 1H), 8.17 (d, *J* = 8.8 Hz, 1H), 7.87–7.70 (m, 2H), 7.59–7.42 (m, 3H), 7.31 (d, *J* = 8.4 Hz, 1H), 6.95 (d, *J* = 8.4 Hz, 1H), 4.43 (d, *J* = 15.1 Hz, 1H), 4.34–4.24 (m, 4H), 3.75 (s, 1H), 3.11 (s, 1H), 2.77 (s, 1H), 2.51 (s, 1H), 2.34 (s, 3H), 2.12 (s, 1H), 1.83 (s, 2H), 1.64–1.49 (m, 1H), 1.27 (d, *J* = 5.9 Hz, 3H). ^13^C NMR (126 MHz, DMSO-*d*_6_) δ 165.39, 164.83, 148.55, 146.81, 143.39, 137.88, 137.79, 136.69, 132.43, 130.61, 129.22, 129.15, 128.65, 128.50, 128.14, 126.68, 122.41, 121.67, 119.07, 118.66, 117.31, 117.14, 64.86, 64.49, 60.40, 60.30, 60.08, 54.50, 32.72, 21.94, 19.24, 17.95. HRMS (ESI^+^): calcd for C_32_H_32_N_4_NaO_4_ (M + Na)^+^, 559.2316; found 559.2308.

##### *N*-(5-(2,3-Dihydrobenzo[*b*][1,4]dioxine-6-carboxamido)-2-methylphenyl)-2-((4-ethylpiperazin-1-yl)methyl)quinoline-6-carboxamide

To a solution of methyl 2-formylquinoline-6-carboxylate (150 mg, 0.697 mmol) in anhydrous DCM, 1-ethylpiperazine (0.266 mL, 2.09 mmol) was added dropwise at room temperature and the resulting mixture was allowed to stir under an inert argon atmosphere for 2.5 h. Then, sodium triacetoxyborohydride (443 mg, 2.09 mmol) was added in one portion and the resulting mixture was allowed to stir overnight at room temperature. The reaction mixture was diluted with DCM (20 mL) and quenched with a NaHCO_3_ saturated aqueous solution (20 mL). The aqueous phase was extracted with DCM (3 × 10 mL), and the combined organic layers were dried over Na_2_SO_4_ and concentrated under reduced pressure to afford a yellow amorphous solid as a crude product, which was carried onto the next step without purification (197 mg). ^1^H NMR (500 MHz, CDCl_3_) δ 8.55 (d, *J* = 1.9 Hz, 1H), 8.31–8.15 (m, 2H), 8.08 (dt, *J* = 8.9, 0.7 Hz, 1H), 7.69 (d, *J* = 8.5 Hz, 1H), 3.97 (s, 3H), 3.85 (s, 2H), 2.57 (d, *J* = 47.4 Hz, 8H), 2.42 (q, *J* = 7.2 Hz, 2H), 1.08 (t, *J* = 7.2 Hz, 3H). ^13^C NMR (126 MHz, CDCl_3_) δ 166.67, 162.22, 149.53, 137.47, 130.66, 129.33, 128.90, 127.62, 126.49, 121.86, 65.10, 53.37, 52.76, 52.36, 52.29, 11.94.

To a solution of methyl 2-((4-ethylpiperazin-1-yl)methyl)quinoline-6-carboxylate (197 mg, 0.629 mmol) in THF (3.0 mL), 2 M aqueous NaOH (1.57 mL, 3.14 mmol) was added dropwise at 20 °C and MeOH (1.2 mL) was added to increase the miscibility of the two layers. The resulting red/brown solution was allowed to stir at 20 °C for 2 h. The reaction mixture was concentrated under reduced pressure to remove THF and MeOH; then, the aqueous layer was acidified to pH 3 with 1 M aqueous HCl and washed with EtOAc (3 × 5 mL). The aqueous layer was concentrated under vacuo to afford a salmon solid as a crude product, which was carried onto the next step without purification. ^1^H NMR (500 MHz, DMSO-*d*_6_) δ 13.37 (br s, 1H), 8.80–8.62 (m, 2H), 8.27 (dd, *J* = 8.9, 2.0 Hz, 1H), 8.16 (d, *J* = 8.8 Hz, 1H), 7.84 (d, *J* = 8.4 Hz, 1H), 4.56 (s, 2H), 3.62 (br s, 8H), 3.18 (br s, 2H), 1.26 (t, *J* = 7.3 Hz, 3H). HRMS (ESI^+^): calcd for C_17_H_22_N_3_O_2_ (M + H)^+^, 302.1764; found 302.1762.

To a solution of 2-(7-aza-1*H*-benzotriazole-1-yl)-1,1,3,3-tetramethyluronium hexafluorophosphate (HATU) (159 mg, 0.418 mmol) and 2-((4-ethylpiperazin-1-yl)methyl)quinoline-6-carboxylic acid hydrochloride salt (100 mg, 0.298 mmol) in anhydrous DMF (2.3 mL) with *N,N-*diisopropylethylamine (0.291 mL, 1.67 mmol), *N-*(3-amino-4-methylphenyl)-2,3-dihydrobenzo[*b*][1,4]dioxine-6-carboxamide (95.0 mg, 0.334 mmol) was added in one portion and the resulting mixture was allowed to stir at 20 °C for 18 h. The reaction mixture was poured onto water (3 mL) to afford a pale yellow precipitate, which was washed with water (3 × 5 mL). Then, the solid was purified by flash column chromatography eluting with 20% EtOAc in DCM and then a gradient of 0–10% MeOH in DCM + 1% 7 N NH_3_ in MeOH to afford the title compound as a pale yellow amorphous solid (48 mg, 25%). ^1^H NMR (500 MHz, MeOD) δ 8.58 (d, *J* = 2.0 Hz, 1H), 8.45 (d, *J* = 8.5 Hz, 1H), 8.29 (dd, *J* = 8.9, 2.1 Hz, 1H), 8.13 (d, *J* = 8.8 Hz, 1H), 7.85–7.73 (m, 2H), 7.59–7.39 (m, 3H), 7.29 (dd, *J* = 8.3, 0.9 Hz, 1H), 6.93 (d, *J* = 8.4 Hz, 1H), 4.40–4.17 (m, 4H), 3.89 (s, 2H), 2.93–2.37 (m, 10H), 2.33 (s, 3H), 1.11 (t, *J* = 7.2 Hz, 3H). ^13^C NMR (126 MHz, MeOD) δ 166.87, 166.54, 161.28, 148.33, 147.01, 143.45, 138.07, 136.96, 135.72, 132.24, 130.35, 130.19, 128.13, 128.01, 127.92, 127.59, 126.83, 122.13, 120.71, 119.45, 119.21, 116.76, 116.61, 64.52, 64.13, 64.01, 52.60, 52.20, 51.87, 48.44, 16.39, 10.37. HRMS (ESI^+^): calcd for C_33_H_36_N_5_O_4_ (M + H)^+^, 567.2793; found 567.2789.

##### *N*-(3-Amino-4-fluorophenyl)-2,3-dihydrobenzo[*b*][1,4]dioxine-6-carboxamide

Oxalyl chloride (18.5 mL, 211 mmol) was added to a stirred solution of 1,4-benzodioxane-6-carboxylic acid (34.6 g, 192 mmol) and pyridine (31.1 mL, 384 mmol) in anhydrous DCM (400 mL) at 0 °C. After 1 h, the reaction mixture was concentrated in vacuo. The remaining residue was redissolved in anhydrous DCM (200 mL) and concentrated in vacuo. The remaining residue was redissolved in DCM (40 mL) and added to a stirred solution of 4-fluoro-3-nitroaniline (30 g, 192 mmol) and pyridine (31.1 mL, 384 mmol) in DCM (400 mL) at 0 °C. After stirring for 16 h, the reaction mixture was concentrated in vacuo and diluted with MeOH (400 mL) and water (400 mL). A precipitate was formed, which was isolated by filtration and washed with water. The solid was dried under vacuum to afford the desired product as a yellow amorphous solid (52.2 g, 85%). ^1^H NMR (500 MHz, DMSO-*d*_6_) δ 10.47 (s, 1H), 8.69 (dd, *J* = 6.9, 2.8 Hz, 1H), 8.13 (ddd, *J* = 9.1, 4.0, 2.8 Hz, 1H), 7.66–7.44 (m, 3H), 7.01 (d, *J* = 8.4 Hz, 1H), 4.32 (td, *J* = 5.3, 3.6 Hz, 4H). ^13^C NMR (126 MHz, DMSO-*d*_6_) δ 165.30, 150.98 (d, *J* = 252.69 Hz), 147.28, 143.49, 136.69 (d, *J* = 7.65 Hz), 136.57 (d, *J* = 2.77 Hz), 127.95 (d, *J* = 8.74 Hz), 127.19, 121.83, 119.10 (d, *J* = 22.18 Hz), 117.50, 117.21, 117.03 (d, *J* = 2.17 Hz), 64.91, 64.49. HRMS (ESI+): calcd for C_15_H_12_FN_2_O_5_ (M + H)^+^ 319.0725; found 319.0729.

Ammonium chloride (10.3g, 192 mmol) and iron (10.7 g, 192 mmol) were added to a mixture of *N-*(4-fluoro-3-nitrophenyl)-2,3-dihydrobenzo[*b*][1,4]dioxine-6-carboxamide (12.228 g, 38.4 mmol) in ethanol (120 mL) and water (40 mL). The reaction was refluxed at 90 °C for 1 h. The reaction was cooled to room temperature and diluted with DCM (30 mL) and MeOH (30 mL). The resulting mixture was filtered through celite and washed with MeOH. The filtrate was concentrated under reduced pressure. The crude solid was diluted in an aqueous saturated NaHCO_3_ solution (150 mL) to make a slurry, which was filtered. The solid was collected, washed with water, and then diluted with toluene and dried in vacuo to afford the crude product as a beige amorphous solid, used as a crude in the next synthetic step (6.25 g). ^1^H NMR (500 MHz, DMSO-*d*_6_) δ 9.81 (s, 1H), 7.55–7.41 (m, 2H), 7.28 (d, *J* = 7.8 Hz, 1H), 7.02–6.78 (m, 3H), 5.14 (s, 2H), 4.30 (d, *J* = 5.6 Hz, 4H). ^13^C NMR (126 MHz, DMSO-*d*_6_) δ 164.63, 147.57 (d, *J* = 227.57 Hz), 146.62, 143.34, 136.56 (d, *J* = 14.52 Hz), 136.06 (d, *J* = 2.88 Hz), 128.37, 121.59, 117.24, 117.08, 114.86 (d, *J* = 21.65 Hz), 109.02 (d, *J* = 2.54 Hz), 108.65 (d, *J* = 5.94 Hz), 64.84, 64.48. HRMS (ESI+): calcd for C_15_H_14_FN_2_O_3_ (M + H)^+^ 289.0988; found 289.0992.

##### 2-((4-Isopropylpiperazin-1-yl)methyl)quinoline-6-carboxylic Acid

Pyrrolidine (0.399 mL, 2.79 mmol) was added to a suspension of methyl 2-formylquinoline-6-carboxylate (0.200 g, 0.929 mmol) in anhydrous DCM (9 mL). The reaction mixture was stirred at room temperature for 2.5 h. Then, sodium triacetoxyborohydride (0.591 g, 2.79 mmol) was added in one portion and the reaction mixture was stirred overnight at room temperature. The reaction mixture was diluted with DCM (20 mL) and washed with a NaHCO_3_ saturated aqueous solution (1 × 20 mL). The two layers were separated, and the aqueous layer was extracted with DCM (3 × 10 mL). The combined organic layers were dried over MgSO_4_ and concentrated to afford the crude product as an amorphous orange solid (322 mg). ^1^H NMR (500 MHz, CDCl_3_) δ 8.54 (d, *J* = 1.9 Hz, 1H), 8.25 (dd, *J* = 8.8, 1.9 Hz, 1H), 8.18 (dd, *J* = 8.6, 0.7 Hz, 1H), 8.07 (d, *J* = 8.7 Hz, 1H), 7.69 (d, *J* = 8.5 Hz, 1H), 3.96 (s, 3H), 3.84 (s, 2H), 2.67–2.52 (m, 9H), 1.04 (s, 3H), 1.03 (s, 3H). ^13^C NMR (126 MHz, CDCl_3_) δ 166.81, 162.43, 149.68, 137.55, 130.78, 129.46, 129.00, 127.71, 126.60, 122.00, 77.42, 77.16, 76.91, 65.25, 54.56, 53.88, 52.48, 48.78, 18.77. HRMS (ESI+): calcd for C_19_H_26_N_3_O_2_ (M + H)^+^ 328.2020; found 328.2031.

To a solution of methyl 2-((4-isopropylpiperazin-1-yl)methyl)quinoline-6-carboxylate (320 mg, 0.977 mmol) in THF (6.0 mL), 2 M aqueous NaOH (2.44 mL, 4.89 mmol) was added dropwise at 20 °C and MeOH (2.4 mL) was added to increase the miscibility of the two layers. The resulting red/brown solution was allowed to stir at 20 °C for 2 h. The reaction mixture was concentrated under reduced pressure to remove THF and MeOH; then, the aqueous layer was acidified to pH 3 with 1 M aqueous HCl and washed with EtOAc (3 × 5 mL). The aqueous layer was concentrated under vacuum to afford a salmon solid as a crude product, which was carried onto the next step without purification (306 mg, contains NaCl). ^1^H NMR (500 MHz, DMSO-*d*_6_) δ 8.73 (d, *J* = 1.9 Hz, 1H), 8.71 (d, *J* = 8.6 Hz, 1H), 8.27 (dd, *J* = 8.7, 1.9 Hz, 1H), 8.18 (d, *J* = 8.8 Hz, 1H), 7.89 (dd, *J* = 8.5, 1.8 Hz, 1H), 3.74–3.41 (m, 11H), 1.30 (m, 6H). LCMS (ESI^+^): *t*_R_ = 0.70 min, *m*/*z* = 314, (M + H)^+^.

##### *N*-(5-(2,3-Dihydrobenzo[*b*][1,4]dioxine-6-carboxamido)-2-fluorophenyl)-2-((4-isopropylpiperazin-1-yl)methyl)quinoline-6-carboxamide

*N*-(3-Amino-4-fluorophenyl)-2,3-dihydrobenzo[*b*][1,4]dioxine-6-carboxamide (100 mg, 0.347 mmol), 2-((4-isopropylpiperazin-1-yl)methyl)quinoline-6-carboxylic acid hydrochloride (130 mg, 0.372 mmol), and 1-ethyl-3-(3-dimethylaminopropyl)carbodiimide (EDC, 166 mg, 0.867 mmol) were dissolved in anhydrous DMF (2.5 mL); then, pyridine (0.140 mL, 1.73 mmol) was added dropwise and the resulting mixture was allowed to stir at 20 °C for 20 h. A further portion of 2-((4-isopropylpiperazin-1-yl)methyl)quinoline-6-carboxylic acid (130 mg, 0.416 mmol), EDC (166 mg, 0.867 mmol), and pyridine (0.140 mL, 1.73 mmol) was added, and the resulting mixture was allowed to stir for a total of 72 h at 20 °C. The reaction was quenched with water (5 mL) and extracted with DCM/MeOH 9/1 (3 × 5 mL). Purification by column chromatography using a gradient of 0–10% MeOH in DCM + 1% 7 N NH_3_ in MeOH followed by trituration in diethyl ether afforded the title compound as an orange amorphous solid (50 mg, 9.2% over three steps). ^1^H NMR (500 MHz, DMSO-*d*_6_) δ 10.41 (br s, 1H), 10.21 (br s, 1H), 8.65 (s, 1H), 8.49 (d, *J* = 8.78 Hz, 1H), 8.25 (dd, *J* = 8.78, 1.88 Hz, 1H), 8.14 (dd, *J* = 6.90, 2.51 Hz, 1H), 8.08 (d, *J* = 8.78 Hz, 1H), 7.73 (d, *J* = 8.78 Hz, 1H), 7.68–7.63 (m, 1H), 7.55 (d, *J* = 2.51 Hz, 1H), 7.52 (dd, *J* = 8.78, 1.88 Hz, 1H), 7.29 (app t, *J* = 9.28 Hz, 1H), 6.99 (d, *J* = 8.78 Hz, 1H), 4.36–4.27 (m, 4H), 3.79 (br s, 2H), 3.15–2.34 (m, 9H), 0.99 (br s, 6H). ^13^C NMR (126 MHz, MeOD) δ 166.69, 166.62, 161.22, 152.52 (d, *J* = 245.41 Hz), 151.49, 148.39, 147.12, 143.49, 138.19, 134.88 (d, *J* = 3.22 Hz), 131.99, 128.21, 127.99, 127.37, 126.82, 125.27 (d, *J* = 11.96 Hz), 122.18, 120.76, 119.62 (d, *J* = 7.97 Hz), 119.01, 116.80, 116.64, 115.28 (d, *J* = 21.26 Hz), 64.54, 64.15, 63.80, 55.27, 52.30, 29.34, 16.98. HRMS (ESI^+^): calcd for C_33_H_35_FN_5_O_4_ (M + H)^+^, 584.2668; found 584.2636.

#### Synthetic Route II

##### Ethyl 2-((Tosyloxy)methyl)quinoline-6-carboxylate

To a stirred solution of 2-methylquinoline-6-carboxylic acid (2.00 g, 10.7 mmol) in ethanol (50 mL) was added sulfuric acid (0.4 mL, 10.7 mmol). The reaction was heated to 80 °C under argon for 22 h. The solvent was removed in vacuo. The resulting residue was taken up in water (100 mL). The solution was basified (∼pH 10) by the addition of 2 M aqueous NaOH solution. The resulting precipitate was collected by filtration and washed with copious water and then dried under vacuum to afford a pale pink amorphous solid (1.57 g, 68%). ^1^H NMR (500 MHz, CDCl_3_) δ 8.56 (d, *J* = 1.9 Hz, 1H), 8.29 (dd, *J* = 8.8, 1.9 Hz, 1H), 8.19–8.13 (m, 1H), 8.05 (dt, *J* = 8.8, 0.7 Hz, 1H), 7.36 (d, *J* = 8.4 Hz, 1H), 4.46 (q, *J* = 7.1 Hz, 2H), 2.79 (s, 3H), 1.46 (t, *J* = 7.1 Hz, 3H). LCMS (ESI^+^): *t*_R_ = 2.24 min, *m*/*z* 216 (M + H)^+^.

3-Chloroperbenzoic acid (0.695 g, 3.02 mmol) was added to a solution of ethyl 2-methylquinoline-6-carboxylate (0.5 g, 2.32 mmol) in anhydrous DCM (7 mL) at 0 °C. The reaction mixture was then allowed to warm to room temperature and stirred overnight. The orange reaction mixture was washed with 10% aqueous Na_2_SO_3_ solution (1 × 10 mL) and saturated aqueous NaHCO_3_ solution (1 × 10 mL). The two layers were separated, and the aqueous layer was diluted with brine and extracted with DCM (3 × 10 mL). The combined organic phases were dried over MgSO_4_, filtered, and concentrated in vacuo. The crude orange oil was crystallized from EtOAc/PE. The solid was isolated by filtration and washed with PE/EtOAc (3/1 mixture). A second product fraction was isolated after the concentration of the filtrate. This solid was triturated with PE/EtOAc (∼4/1) and isolated by filtration. The title compound was obtained as a pale orange amorphous solid (381 mg, 71%). ^1^H NMR (500 MHz, CDCl_3_) δ 8.82 (d, *J* = 9.1 Hz, 1H), 8.58 (d, *J* = 1.7 Hz, 1H), 8.33 (dd, *J* = 9.1, 1.8 Hz, 1H), 7.74 (d, *J* = 8.5 Hz, 1H), 7.39 (d, *J* = 8.5 Hz, 1H), 4.46 (q, *J* = 7.1 Hz, 2H), 2.74 (s, 3H), 1.45 (t, *J* = 7.1 Hz, 3H). LCMS (ESI^+^): *t*_R_ = 2.32 min, *m*/*z* 232.10 (M + H)^+^.

To a solution of ethyl 2-methylquinoline-6-carboxylate *N-*oxide (0.274 g, 1.19 mmol) in anhydrous acetonitrile (10 mL) at 0 °C, K_2_CO_3_ (0.246 g, 1.78 mmol) was added in one portion, followed by *p*-toluenesulfonyl chloride (0.271 g, 1.42 mmol). The reaction mixture was stirred at 0 °C for 4 h. The reaction mixture was diluted with saturated aqueous NaHCO_3_ solution and extracted with EtOAc (2 × 10 mL). The organic layer was washed with water (1 × 10 mL) and brine (1 × 10 mL), dried over MgSO_4_, filtered, and concentrated in vacuo. The crude (dark blue-green solid) was purified by column chromatography using a gradient of 16–40% EtOAc in petroleum ether to afford an orange amorphous solid (186 mg, 41%). ^1^H NMR (500 MHz, CDCl_3_) δ 8.58 (d, *J* = 1.9 Hz, 1H), 8.34–8.25 (m, 2H), 8.01 (d, *J* = 8.8 Hz, 1H), 7.86 (d, *J* = 8.4 Hz, 2H), 7.63 (d, *J* = 8.5 Hz, 1H), 7.33 (d, *J* = 8.1 Hz, 2H), 5.32 (s, 2H), 4.45 (q, *J* = 7.1 Hz, 2H), 2.42 (s, 3H), 1.45 (t, *J* = 7.1 Hz, 3H). LCMS (ESI^+^): *t*_R_ = 3.11 min, *m*/*z* 386.22 (M + H)^+^.

##### *N*-(5-(2,3-Dihydrobenzo[*b*][1,4]dioxine-6-carboxamido)-2-methylphenyl)-2-((dimethylamino)methyl)quinoline-6-carboxamide (**12**)

A solution of ethyl 2-((tosyloxy)methyl)quinoline-6-carboxylate (62.0 mg, 0.161 mmol) in dimethylamine (2 M in THF) (0.080 mL, 0.161 mmol) was heated under microwave irradiation at 60 °C for 1 h. The reaction mixture was concentrated under reduced pressure, diluted with EtOAc, and washed with water (1 × 1 mL) and saturated aqueous NaHCO_3_ solution (1 × 1 mL). The aqueous phase was extracted with EtOAc (1 × 1 mL). The organic layers were combined, dried over Na_2_SO_4_, filtered, and concentrated under reduced pressure to afford an orange oil (42 mg). ^1^H NMR (500 MHz, CDCl_3_) δ 8.57 (d, *J* = 1.8 Hz, 1H), 8.29 (dd, *J* = 8.8, 1.9 Hz, 1H), 8.23 (d, *J* = 8.5 Hz, 1H), 8.11 (d, *J* = 8.8 Hz, 1H), 7.67 (d, *J* = 8.5 Hz, 1H), 4.45 (q, *J* = 7.2 Hz, 2H), 3.79 (s, 2H), 2.35 (s, 6H), 1.45 (t, *J* = 7.1 Hz, 3H).

Aqueous NaOH solution (1.04 M, 0.304 mL, 0.317 mmol) was added to a solution of ethyl 2-((dimethylamino)methyl)quinoline-6-carboxylate (41.0 mg, 0.159 mmol) in THF (1 mL) and MeOH (0.3 mL). The reaction mixture was stirred at room temperature overnight. A further portion of water (0.5 mL) and aqueous NaOH (1.15 M, 0.276 mL, 0.317 mmol) was added, and the resulting mixture was allowed to stir overnight. The reaction mixture was concentrated to remove the organic solvents, diluted with water, and washed with EtOAc (1 × 1 mL). The aqueous phase was acidified to ∼pH 3 with aqueous HCl solution (2 M) and then concentrated to afford the title compound as a crude pale yellow solid, which was carried onto the next step without purification. ^1^H NMR (500 MHz, DMSO-*d*_6_) δ 12.11 (br s, 1H), 8.70 (d, *J* = 1.8 Hz, 1H), 8.65 (d, *J* = 8.5 Hz, 1H), 8.25 (dd, *J* = 8.8, 1.9 Hz, 1H), 8.10 (d, *J* = 8.8 Hz, 1H), 7.84 (d, *J* = 8.5 Hz, 1H), 4.42 (br s, 2H), 2.70 (s, 6H). LCMS (ESI^+^): *t*_R_ = 0.82 min, *m*/*z* 231.11 (M + H)^+^.

HATU (72.0 mg, 0.189 mmol) was added to a solution of 2-((dimethylamino)methyl)quinoline-6-carboxylic acid (0.106 g, 0.151 mmol) and *N,N-*diisopropylethylamine (0.111 mL, 0.634 mmol) in anhydrous DMF (1.5 mL). The reaction mixture was stirred for 5 min before *N-*(3-amino-4-methylphenyl)-2,3-dihydrobenzo[*b*][1,4]dioxine-6-carboxamide (32.0 mg, 0.113 mmol) was added. The reaction mixture was stirred at room temperature overnight. The reaction mixture was diluted with water, and the resulting precipitate was isolated by filtration and washed with water. The residue was purified by column using a gradient of 4–10% MeOH in DCM to afford the title compound as an off-white amorphous solid (24 mg, 30% over 3 steps). ^1^H NMR (500 MHz, DMSO-*d*_6_) δ 10.15 (s, 1H), 10.07 (s, 1H), 8.63 (d, *J* = 1.9 Hz, 1H), 8.48 (d, *J* = 8.5 Hz, 1H), 8.26 (dd, *J* = 8.8, 2.0 Hz, 1H), 8.08 (d, *J* = 8.8 Hz, 1H), 7.88 (d, *J* = 2.1 Hz, 1H), 7.71 (d, *J* = 8.5 Hz, 1H), 7.62–7.48 (m, 3H), 7.24 (d, *J* = 8.5 Hz, 1H), 6.98 (d, *J* = 8.4 Hz, 1H), 4.30 (td, *J* = 5.1, 3.6 Hz, 4H), 3.74 (s, 2H), 2.25 (d, *J* = 6.0 Hz, 9H). ^13^C NMR (126 MHz, DMSO-*d*_6_) δ 165.39, 164.82, 162.29, 148.58, 146.81, 143.39, 137.90, 137.78, 136.69, 132.42, 130.60, 129.22, 129.18, 128.63, 128.42, 128.14, 126.70, 122.25, 121.65, 119.06, 118.65, 117.30, 117.12, 66.13, 64.86, 64.48, 45.88, 17.93. HRMS (ESI^+^): calcd for C_29_H_29_N_4_O_4_ (M + H)^+^, 497.2183; found 497.2183.

##### *N*-(5-(2,3-Dihydrobenzo[*b*][1,4]dioxine-6-carboxamido)-2-methylphenyl)-2-((4-methylpiperazin-1-yl)methyl)quinoline-6-carboxamide (**15**)

1-Methylpiperazine (0.058 mL, 0.519 mmol) was added to a solution of ethyl 2-((tosyloxy)methyl)quinoline-6-carboxylate (80.0 mg, 0.208 mmol) in anhydrous THF (1.5 mL). The reaction mixture was heated to reflux for 1.5 h. The reaction mixture was cooled to room temperature, stirred for 2 h, and concentrated under reduced pressure. The residue was diluted with EtOAc (2 mL) and washed with water (1 × 2 mL) and saturated aqueous NaHCO_3_ solution (1 × 2 mL). The aqueous phase was extracted with EtOAc (1 × 2 mL). The combined organic phases were dried over MgSO_4_, filtered, and concentrated in vacuo to afford the title compound as a yellow amorphous solid, which was carried onto the next step without purification (64 mg). ^1^H NMR (500 MHz, CDCl_3_) δ 8.56 (d, *J* = 1.9 Hz, 1H), 8.29 (dd, *J* = 8.8, 1.9 Hz, 1H), 8.22 (d, *J* = 8.5 Hz, 1H), 8.09 (d, *J* = 8.8 Hz, 1H), 7.71 (d, *J* = 8.5 Hz, 1H), 4.45 (q, *J* = 7.1 Hz, 2H), 3.86 (s, 2H), 2.61 (br s, 4H), 2.49 (br s, 4H), 2.31 (s, 3H), 1.45 (t, *J* = 7.1 Hz, 3H). HRMS (ESI^+^): calcd for C_18_H_24_N_3_O_2_ (M + H)^+^, 314.1863; found 314.1871.

Aqueous NaOH solution (0.82 M, 0.735 mL, 0.603 mmol) was added to a solution of ethyl 2-((4-methylpiperazin-1-yl)methyl)quinoline-6-carboxylate (63.0 mg, 0.201 mmol) in THF (1 mL) and MeOH (0.25 mL), and the reaction mixture was stirred at room temperature overnight. The reaction mixture was concentrated to remove the organic solvents, diluted with water, and washed with EtOAc (1 × 1 mL). The aqueous phase was acidified with aqueous HCl solution (2 M) to pH 2–3 and then concentrated to dryness. The crude product (light brown amorphous solid) was carried onto the next step without further purification (114 mg). ^1^H NMR (500 MHz, DMSO-*d*_6_) δ 13.33 (br s, 1H), 11.77 (br s, 1H), 8.72 (d, *J* = 1.9 Hz, 1H), 8.69 (d, *J* = 8.5 Hz, 1H), 8.26 (dd, *J* = 8.8, 1.9 Hz, 1H), 8.16 (d, *J* = 8.9 Hz, 1H), 7.85 (d, *J* = 8.5 Hz, 1H), 4.53 (br s, 2H), 3.83–3.45 (br m, 8H), 2.80 (s, 3H) (hydrochloric acid salt). HRMS (ESI^+^): calcd for C_16_H_20_N_3_O_2_ (M + H)^+^, 286.1550; found 286.1553.

HATU (68.0 mg, 0.178 mmol) was added to a suspension of 2-((4-methylpiperazin-1-yl)methyl)quinoline-6-carboxylic acid (81.0 mg, 0.142 mmol) and *N,N-*diisopropylethylamine (0.131 mL, 0.748 mmol) in anhydrous DMF (1 mL). The reaction mixture was stirred for 4 min before *N-*(3-amino-4-methylphenyl)-2,3-dihydrobenzo[*b*][1,4]dioxine-6-carboxamide (34.0 mg, 0.121 mmol) was added. The reaction mixture was stirred at room temperature overnight. The reaction mixture was diluted with water, and the resulting precipitate was isolated by filtration and washed with water. The residue was purified by column chromatography using a gradient of 5–18% MeOH in DCM to afford the title compound as an off-white amorphous solid (36 mg, 31% over three steps). ^1^H NMR (500 MHz, DMSO-*d*_6_) δ 10.15 (s, 1H), 10.08 (s, 1H), 8.63 (d, *J* = 1.9 Hz, 1H), 8.48 (d, *J* = 8.4 Hz, 1H), 8.26 (dd, *J* = 8.8, 2.0 Hz, 1H), 8.08 (d, *J* = 8.8 Hz, 1H), 7.88 (d, *J* = 2.1 Hz, 1H), 7.72 (d, *J* = 8.5 Hz, 1H), 7.59 (dd, *J* = 8.3, 2.2 Hz, 1H), 7.56–7.48 (m, 2H), 7.25 (d, *J* = 8.5 Hz, 1H), 6.98 (d, *J* = 8.4 Hz, 1H), 4.31 (q, *J* = 5.1 Hz, 4H), 3.79 (s, 2H), 2.50–2.32 (m, 8H), 2.24 (s, 3H), 2.17 (s, 3H). ^13^C NMR (126 MHz, DMSO-*d*_6_) δ 165.37, 164.82, 161.97, 148.63, 146.81, 143.39, 137.93, 137.78, 136.69, 132.39, 130.61, 129.23, 129.16, 128.64, 128.42, 128.14, 126.69, 122.22, 121.66, 119.07, 118.65, 117.30, 117.12, 64.86, 64.49, 55.18, 53.35, 46.19, 40.89, 17.93. HRMS (ESI^+^): calcd for C_32_H_34_N_5_O_4_ (M + H)^+^, 552.2605; found 552.2591.

#### Synthetic Route III

##### *N*-(5-(2,3-Dihydrobenzo[*b*][1,4]dioxine-6-carboxamido)-2-methylphenyl)-2-(2-(piperidin-1-yl)ethoxy)quinoline-6-carboxamide (**17**)

4-(2-Hydroxyethyl)piperidine (0.197 mL, 1.49 mmol) was added to a suspension of NaH (60%, 57.0 mg, 1.42 mmol) in anhydrous THF at 0 °C. The reaction mixture was stirred for 5 min and then allowed to warm to room temperature and stirred for 35 min before 6-bromo-2-chloroquinoline (300 mg, 1.24 mmol) was added. The reaction mixture was then heated to reflux. After 4.5 h, the reaction mixture was cooled to room temperature and diluted with first water and then saturated aqueous NaHCO_3_ solution. This mixture was extracted with DCM three times. The combined organic layers were washed with water, dried over MgSO_4_, and concentrated. The crude product was purified by column chromatography using a gradient of 4–5% MeOH in DCM to give 6-bromo-2-(2-(piperidin-1-yl)ethoxy)quinoline as a pale yellow oil (334 mg, 81%). ^1^H NMR (500 MHz, CDCl_3_) δ 7.89 (d, *J* = 8.9 Hz, 1H), 7.87–7.84 (m, 1H), 7.72–7.65 (m, 2H), 6.95 (d, *J* = 8.8 Hz, 1H), 4.62 (t, *J* = 6.1 Hz, 2H), 2.83 (t, *J* = 6.1 Hz, 2H), 1.63 (m, 6H), 1.53–0.46 (m, 4H). HRMS (ESI^+^): calcd for C_16_H_20_^79^BrN_2_O (M + H)^+^ 335.0754, found 335.0787.

*n*-BuLi (1.62 M in hexanes, 0.742 mL, 1.21 mmol) was added dropwise to a solution of 6-bromo-2-(2-(piperidin-1-yl)ethoxy)quinoline (325 mg, 0.969 mmol) in anhydrous THF (3.25 mL) at −78 °C. The reaction mixture was stirred at −78 °C for 50 min before solid CO_2_ was added. After stirring for 5 min, the reaction mixture was allowed to warm to room temperature. The reaction was quenched with water and reduced in vacuo to remove the THF. The remaining residue was diluted with water and washed with ethyl acetate. The aqueous layer was then acidified to pH 3 by the addition of aqueous 2 M HCl and concentrated to dryness to give the product as an off-white amorphous solid. The product, 2-(2-(piperidin-1-yl)ethoxy)quinoline-6-carboxylic acid hydrochloride, was used in the next synthetic step without further purification (301 mg, contains LiCl). ^1^H NMR (500 MHz, DMSO-*d*_6_) δ 8.59 (d, *J* = 2.0 Hz, 1H), 8.47 (d, *J* = 8.9 Hz, 1H), 8.16 (dd, *J* = 8.7, 2.0 Hz, 1H), 7.84 (d, *J* = 8.7 Hz, 1H), 7.16 (d, *J* = 8.9 Hz, 1H), 5.08–4.70 (m, 2H), 3.75–3.36 (m, 4H), 3.01 (tdd, *J* = 12.3, 9.1, 3.3 Hz, 2H), 1.92–1.75 (m, 4H), 1.74–1.55 (m, 1H), 1.38 (ddt, *J* = 12.7, 8.1, 4.0 Hz, 1H). HRMS (ESI^+^): calcd for C_17_H_21_N_2_O_3_ (M + H)^+^ 301.1547, found 301.1534.

HATU (111 mg, 0.293 mmol) was added to a solution of 2-(2-(piperidin-1-yl)ethoxy)quinoline-6-carboxylic acid hydrochloride (93.0 mg, contains LiCl, purity 85%) and DIPEA (0.174 mL, 0.997 mmol) in anhydrous DMF (1.5 mL). The reaction mixture was stirred for 5 min before *N-*(3-amino-4-methylphenyl)2,3-dihydrobenzo[*b*][1,4]dioxine-6-carboxamide (50.0 mg, 0.176 mmol) was added. The resulting reaction mixture was stirred at room temperature overnight. The reaction mixture was then diluted with water, and the resulting precipitate was isolated by filtration and washed with water. The crude product was purified by column chromatography using a gradient of 3.5–10% MeOH in DCM to afford the title compound as a white amorphous solid (75.0 mg, 75%). ^1^H NMR (500 MHz, DMSO-*d*_6_) δ 10.07 (s, 2H), 8.56 (d, *J* = 2.1 Hz, 1H), 8.38 (d, *J* = 8.8 Hz, 1H), 8.22 (dd, *J* = 8.8, 2.1 Hz, 1H), 7.90–7.79 (m, 2H), 7.58 (dd, *J* = 8.3, 2.2 Hz, 1H), 7.54 (d, *J* = 2.1 Hz, 1H), 7.51 (dd, *J* = 8.4, 2.2 Hz, 1H), 7.23 (d, *J* = 8.4 Hz, 1H), 7.11 (d, *J* = 8.8 Hz, 1H), 6.98 (d, *J* = 8.4 Hz, 1H), 4.57 (s, 2H), 4.37–4.24 (m, 4H), 2.75 (s, 2H), 2.50 (p, *J* = 1.8 Hz, 4H), 2.23 (s, 3H), 1.51 (d, *J* = 7.6 Hz, 4H), 1.38 (s, 2H). ^13^C NMR (126 MHz, DMSO*-d*_6_) δ 165.39, 164.81, 163.15, 148.00, 146.80, 143.39, 140.71, 137.75, 136.79, 130.57, 130.52, 129.23, 128.88, 128.57, 128.15, 127.21, 124.48, 121.65, 119.08, 118.58, 117.30, 117.12, 114.47, 64.86, 64.48, 63.73, 57.50, 54.76, 25.95, 24.30, 17.95. HRMS (ESI^+^): calcd for C_33_H_35_N_4_O_5_ (M + H)^+^ 567.2602, found 567.2635.

##### 2-(2-(Piperidin-1-yl)propoxy)quinoline-6-carboxylic Acid

1-Piperidinepropanol (0.235 mL, 1.55 mmol) was added to a suspension of NaH (60%, 59.0 mg, 1.49 mmol) in anhydrous THF (4 mL) at 0 °C. The reaction mixture was stirred for 5 min and then allowed to warm to room temperature and stirred for 40 min before 6-bromo-2-chloroquinoline (300 mg, 1.24 mmol) was added. The reaction mixture was then heated to reflux. After 6 h, the reaction mixture was cooled to room temperature and concentrated to remove the THF. The remaining residue was diluted with water first and then saturated aqueous NaHCO_3_ solution. This mixture was extracted with DCM three times. The combined organic layers were washed with water, dried over MgSO_4_, and concentrated. The crude product was purified by column chromatography using a gradient of 2.5–6% of MeOH in DCM to give 6-bromo-2-(2-(piperidin-1-yl)propoxy)quinoline as a pale yellow oil that solidified (329 mg, 76%). ^1^H NMR (500 MHz, CDCl_3_) δ 7.89 (d, *J* = 8.9 Hz, 1H), 7.86 (d, *J* = 1.9 Hz, 1H), 7.73–7.61 (m, 2H), 6.91 (d, *J* = 8.8 Hz, 1H), 4.50 (t, *J* = 6.5 Hz, 2H), 2.63–2.35 (m, 6H), 2.05 (p, *J* = 6.7 Hz, 2H), 1.75–1.55 (m, 4H), 1.47 (m, 2H). HRMS (ESI^+^): calcd for C_17_H_22_^79^BrN_2_O (M + H)^+^ 351.0893, found 351.0883.

*n*-BuLi (1.56 M in hexanes, 0.732 mL, 1.14 mmol) was added dropwise to a solution of 6-bromo-2-(2-(piperidin-1-yl)propoxy)quinoline (319 mg, 0.913 mmol) in anhydrous THF (3.1 mL) at −78 °C. The reaction mixture was stirred at −78 °C for 45 min before solid CO_2_ was added. After stirring for 5 min, the reaction mixture was allowed to warm to room temperature. The reaction was quenched with water and reduced in vacuo to remove the THF. The remaining residue was diluted with water and washed with ethyl acetate. The aqueous layer was then acidified to pH 3 by the addition of aqueous 2 M HCl and concentrated to dryness to give the product as an off-white amorphous solid. The product, 2-(2-(piperidin-1-yl)propoxy)quinoline-6-carboxylic acid hydrochloride, was used in the next synthetic step without further purification (347 mg, contains LiCl). ^1^H NMR (500 MHz, CDCl_3_) δ 7.89 (d, *J* = 8.9 Hz, 1H), 7.86 (d, *J* = 1.9 Hz, 1H), 7.73–7.61 (m, 2H), 6.91 (d, *J* = 8.8 Hz, 1H), 4.50 (t, *J* = 6.5 Hz, 2H), 2.63–2.35 (m, 6H), 2.05 (p, *J* = 6.7 Hz, 2H), 1.75–1.55 (m, 4H), 1.47 (s, 2H). HRMS (ESI^+^): calcd for C_18_H_23_N_2_O_3_ (M + H)^+^ 315.1703, found 315.1686.

##### *N*-(5-(2,3-Dihydrobenzo[*b*][1,4]dioxine-6-carboxamido)-2-methylphenyl)-2-(2-(piperidin-1-yl)propoxy)quinoline-6-carboxamide

HATU (89.0 mg, 0.234 mmol) was added to a solution of 2-(2-(piperidin-1-yl)propoxy)quinoline-6-carboxylic acid hydrochloride (76.0 mg, 0.188 mmol, contains LiCl) and DIPEA (0.139 mL, 0.797 mmol) in anhydrous DMF (1.35 mL). The reaction mixture was stirred for 5 min before *N-*(3-amino-4-methylphenyl)2,3-dihydrobenzo[*b*][1,4]dioxine-6-carboxamide (40.0 mg, 0.141 mmol) was added. The resulting reaction mixture was stirred at room temperature overnight. The reaction mixture was then diluted with water, and the resulting precipitate was isolated by filtration and washed with water. The crude product was purified by column chromatography using a gradient of 2.5–15% MeOH in DCM to give the title compound as an off-white amorphous solid (53 mg, 65%). ^1^H NMR (500 MHz, DMSO-*d*_6_) δ 10.07 (s, 2H), 8.57 (d, *J* = 2.1 Hz, 1H), 8.38 (d, *J* = 8.9 Hz, 1H), 8.22 (dd, *J* = 8.7, 2.1 Hz, 1H), 7.94–7.80 (m, 2H), 7.58 (dd, *J* = 8.3, 2.2 Hz, 1H), 7.54 (d, *J* = 2.1 Hz, 1H), 7.51 (dd, *J* = 8.5, 2.2 Hz, 1H), 7.23 (d, *J* = 8.4 Hz, 1H), 7.10 (d, *J* = 8.8 Hz, 1H), 6.98 (d, *J* = 8.4 Hz, 1H), 4.49 (t, *J* = 6.5 Hz, 2H), 4.35–4.26 (m, 4H), 2.50 (m, 6H), 2.23 (s, 3H), 2.01 (m, 2H), 1.56 (m, 4H), 1.42 (m, 2H). ^13^C NMR (126 MHz, DMSO-*d*_6_) δ 165.38, 164.81, 163.24, 148.04, 146.80, 143.39, 140.69, 137.75, 136.78, 130.57, 130.51, 129.23, 128.89, 128.60, 128.14, 127.17, 124.47, 121.65, 119.09, 118.59, 117.30, 117.12, 114.41, 64.86, 64.66, 64.48, 55.57, 54.13, 25.76, 25.45, 23.47, 17.94. HRMS (ESI^+^): calcd for C_34_H_37_N_4_O_5_ (M + H)^+^ 581.2759, found 581.2712.

##### 1-(3-(Pyrrolidin-1-yl)propoxy)quinoline-6-carboxylic Acid

Pyrrolidine (1.00 mL, 12.0 mmol) was added to a suspension of potassium carbonate (1.29 g, 9.35 mmol) and 3-bromopropanol (0.65 mL, 7.19 mmol) in anhydrous THF (3 mL) at 0 °C. The reaction mixture was then allowed to warm to room temperature and stirred overnight. The reaction was then diluted with ethyl acetate and filtered through a pad of silica gel. The filtrate was concentrated to give 3-(pyrrolidin-1-yl)propan-1-ol as a colorless oil (645 mg, 69%). ^1^H NMR (500 MHz, CDCl_3_) δ 3.88–3.73 (m, 2H), 2.82–2.67 (m, 2H), 2.66–2.51 (m, 4H), 1.81–1.75 (m, 4H), 1.75–1.69 (m, 2H).

NaH (60%, 59 mg, 1.476 mmol) was added to a solution of 3-(pyrrolidin-1-yl)propan-1-ol (199 mg, 1.540 mmol) in anhydrous THF at 0 °C. The reaction mixture was stirred for 5 min, then allowed to warm to room temperature and stirred for 35 min before 6-bromo-2-chloroquinoline (311 mg, 1.28 mmol) was added. The reaction mixture was then heated to reflux. After 3.5 h, the reaction mixture was cooled to room temperature and concentrated to remove the THF. The remaining residue was diluted with water first and then saturated aqueous NaHCO_3_ solution. This mixture was extracted with DCM three times. The combined organic layers were washed with water, dried over MgSO_4_, and concentrated. The crude product was purified by column chromatography using a gradient of 3–6% MeOH in DCM to give 6-bromo-2-(3-(pyrrolidin-1-yl)propoxy)quinoline as an off-white amorphous solid (290 mg, 67%). ^1^H NMR (500 MHz, CDCl_3_) δ 7.89 (d, *J* = 8.8 Hz, 1H), 7.87–7.84 (m, 1H), 7.73–7.63 (m, 2H), 6.92 (d, *J* = 8.8 Hz, 1H), 4.53 (t, *J* = 6.5 Hz, 2H), 2.70–2.62 (m, 2H), 2.57 (s, 5H), 2.14–2.02 (m, 2H), 1.82 (m, 4H). HRMS (ESI^+^): calcd for C_16_H_20_^79^BrN_2_O (M + H)^+^ 335.0754, found 335.0763.

*n*-BuLi (2.22 M in hexanes, 0.472 mL, 1.048 mmol) was added dropwise to a solution of 6-bromo-2-(3-(pyrrolidin-1-yl)propoxy)quinoline (281 mg, 0.838 mmol) in anhydrous THF (6 mL) at −78 °C. The reaction mixture was stirred at −78 °C for 40 min before solid CO_2_ was added. After stirring for 5 min, the reaction mixture was allowed to warm to room temperature. The reaction was quenched with water and reduced in vacuo to remove the THF. The remaining residue was diluted with water and washed with ethyl acetate. The precipitate was carried through in the aqueous layer. The aqueous layer was then acidified to pH 3 by the addition of aqueous 2 M HCl. At this point, the precipitate dissolved and the solution was concentrated to dryness. The crude product was triturated with acetonitrile and dried to give the product as a dull yellow solid. The product, 2-(3-(pyrrolidin-1-yl)propoxy)quinoline-6-carboxylic acid hydrochloride, was used in the next synthetic step without further purification (298 mg, contains LiCl). ^1^H NMR (500 MHz, DMSO-*d*_6_) δ 8.57 (d, *J* = 2.0 Hz, 1H), 8.52–8.35 (m, 1H), 8.15 (dd, *J* = 8.7, 2.0 Hz, 1H), 7.82 (dd, *J* = 8.7, 0.7 Hz, 1H), 7.10 (d, *J* = 8.9 Hz, 1H), 4.53 (t, *J* = 6.2 Hz, 2H), 3.55 (q, *J* = 5.3 Hz, 2H), 3.36–3.24 (m, 2H), 3.08–2.89 (m, 2H), 2.29–2.13 (m, 2H), 1.99 (q, *J* = 7.3, 6.5 Hz, 2H), 1.93–1.79 (m, 2H). HRMS (ESI^+^): calcd for C_17_H_21_N_2_O_3_ (M + H)^+^ 301.15467, found 301.1501.

##### *N*-(5-(2,3-Dihydrobenzo[*b*][1,4]dioxine-6-carboxamido)-2-methylphenyl)-2-(3-(pyrrolidin-1-yl)propoxy)quinoline-6-carboxamide

HATU (121 mg, 0.319 mmol) was added to a solution of 2-(3-(pyrrolidin-1-yl)propoxy)quinoline-6-carboxylic acid hydrochloride (100 mg, contains LiCl) and DIPEA (0.190 mL, 1.085 mmol) in anhydrous DMF (10 mL). The reaction mixture was stirred for 5 min before *N-*(3-amino-4-methylphenyl)2,3-dihydrobenzo[*b*][1,4]dioxine-6-carboxamide (54.0 mg, 0.192 mmol) was added followed by anhydrous DMF (1.5 mL) to rinse the vial. The resulting reaction mixture was stirred at room temperature overnight. The reaction mixture was then diluted with water, and the resulting precipitate was isolated by filtration and washed with water. The crude product was purified by column chromatography using a gradient of 5–18% MeOH in DCM to afford the title compound as an off-white amorphous solid (65 mg, 60%). ^1^H NMR (500 MHz, DMSO*-d*_6_) δ 10.07 (s, 2H), 8.57 (d, *J* = 2.0 Hz, 1H), 8.38 (d, *J* = 8.8 Hz, 1H), 8.22 (dd, *J* = 8.8, 2.1 Hz, 1H), 7.88–7.84 (m, 2H), 7.58 (dd, *J* = 8.3, 2.2 Hz, 1H), 7.54 (d, *J* = 2.2 Hz, 1H), 7.51 (dd, *J* = 8.5, 2.2 Hz, 1H), 7.24 (d, *J* = 8.3 Hz, 1H), 7.10 (d, *J* = 8.8 Hz, 1H), 6.98 (d, *J* = 8.4 Hz, 1H), 4.49 (t, *J* = 6.6 Hz, 2H), 4.33–4.28 (m, 4H), 2.65 (br s, 2H), 2.54 (br s, 4H), 2.23 (s, 3H), 1.99 (p, *J* = 6.8 Hz, 2H), 1.72 (br s, 4H). ^13^C NMR (126 MHz, DMSO-*d*_6_) δ 164.95, 164.36, 162.86, 147.62, 146.35, 142.94, 140.21, 137.31, 136.34, 130.12, 130.03, 128.78, 128.42, 128.15, 127.69, 126.73, 124.01, 121.21, 118.63, 118.13, 116.85, 116.67, 113.98, 64.40, 64.33, 64.03, 53.59, 52.24, 27.57, 23.07, 17.49. HRMS (ESI^+^): calcd for C_33_H_35_N_4_O_5_ (M + H)^+^ 567.2602, found 567.2698.

##### *N*-(5-(2,3-Dihydrobenzo[*b*][1,4]dioxine-6-carboxamido)-2-fluorophenyl)-2-(2-(pyrrolidin-1-yl)ethoxy)quinoline-6-carboxamide

2-Fluoro-5-nitroaniline (27.3 mg, 0.175 mmol), 2-(2-(pyrrolidin-1-yl)ethoxy)quinoline-6-carboxylic acid hydrochloride (50 mg, 0.175 mmol), and EDC (67.0 mg, 0.349 mmol) were dissolved in anhydrous DMF (1.0 mL) and pyridine (0.070 mL, 0.873 mmol) was added dropwise. The mixture was stirred at 20 °C for 5 h. The reaction mixture was diluted with DCM/MeOH and washed with saturated aqueous NaHCO_3_. Purification by column chromatography using a gradient of 0–50% MeOH in DCM afforded the desired product *N-*(2-fluoro-5-nitrophenyl)-2-(2-(pyrrolidin-1-yl)ethoxy)quinoline-6-carboxamide as a pale yellow solid (40 mg). LCMS (ESI^+^): *t*_R_ = 1.13 min, *m*/*z* 425.16 (M + H)^+^.

*N-*(2-fluoro-5-nitrophenyl)-2-(2-(pyrrolidin-1-yl)ethoxy)quinoline-6-carboxamide (40 mg, 0.094 mmol), ammonium chloride (35.3 mg, 0.66 mmol), and iron powder (36.8 mg, 0.66 mmol) were suspended in a mixture of ethanol (1.5 mL) and water (0.5 mL), and the resulting mixture was heated at 90 °C for 1 h. Then, the reaction mixture was cooled to room temperature and filtered through celite. The solvents were removed under vacuo to afford *N-*(5-amino-2-fluorophenyl)-2-(2-(pyrrolidin-1-yl)ethoxy)quinoline-6-carboxamide as a light beige solid (37.2 mg). LCMS (ESI^+^): *t*_R_ = 0.79 min, *m*/*z* 395.19 (M + H)^+^.

*N-*(5-Amino-2-fluorophenyl)-2-(2-(pyrrolidin-1-yl)ethoxy)quinoline-6-carboxamide (37.2 mg, 0.094 mmol), 2,3-dihydrobenzo[*b*][1,4]dioxine-6-carboxylic acid (16.90 mg, 0.094 mmol), and EDC (45.0 mg, 0.235 mmol) were dissolved in anhydrous DMF (0.6 mL); then, pyridine (0.038 mL, 0.469 mmol) was added dropwise and the resulting mixture was stirred at 20 °C for 18 h. The reaction mixture was diluted with DCM/MeOH and washed with water (5 mL) to afford a pale yellow solid as a crude product, which was purified by column chromatography using a gradient of 0–10% MeOH in DCM. This residue was then repurified via semipreparative TLC (DCM/MeOH 9/1) to afford the title compound as a white amorphous solid (5 mg, 9.3% over three steps). ^1^H NMR (500 MHz, MeOD) δ 8.48 (d, *J* = 2.04 Hz, 1H), 8.29 (d, *J* = 8.85 Hz, 1H), 8.20 (dd, *J* = 8.17, 2.04 Hz, 1H), 8.17 (dd, *J* = 6.81, 2.72 Hz, 1H), 7.92 (d, *J* = 8.17 Hz, 1H), 7.62–7.56 (m, 1H), 7.50 (d, *J* = 2.04 Hz, 1H), 7.48 (dd, *J* = 8.17, 2.04 Hz, 1H), 7.23 (app t, *J* = 9.79 Hz, 1H), 7.09 (d, *J* = 8.85 Hz, 1H), 6.96 (d, *J* = 8.17 Hz, 1H), 4.70 (t, *J* = 5.32 Hz, 2H), 4.36–4.28 (m, 4H), 3.08 (t, *J* = 5.25 Hz, 2H), 2.84–2.76 (m, 4H), 1.91–1.85 (m, 4H). ^13^C NMR (126 MHz, MeOD) δ 166.91, 166.59, 163.24, 152.49 (d, *J* = 249.95 Hz), 148.40, 147.13, 143.51, 139.86, 134.85 (d, *J* = 2.13 Hz), 129.65, 127.92, 127.43, 127.03, 125.60, 125.41 (d, *J* = 11.60 Hz), 124.46, 120.76, 119.54 (d, *J* = 9.28 Hz), 119.03, 116.81, 116.64, 115.23 (d, *J* = 18.56 Hz), 113.86, 64.54, 64.15, 64.08, 54.37, 54.13, 22.80. HRMS (ESI^+^): calcd for C_31_H_30_FN_4_O_5_ (M + H)^+^, 557.2195; found 557.2196.

##### *N*-(3-Amino-4-chlorophenyl)-2,3-dihydrobenzo[*b*][1,4]dioxine-6-carboxamide

Oxalyl chloride (0.141 mL, 1.67 mmol) was added dropwise to a solution of 1,4-benzodioxane-6-carboxylic acid (250 mg, 1.39 mmol) and *N*,*N-*dimethylformamide (3 μL, 0.035 mmol) in anhydrous DCM (7 mL) under an inert atmosphere at room temperature. Effervescence was observed, and the reaction was stirred for 2 h. The reaction mixture was concentrated, anhydrous DCM was added (10 mL), and the reaction was concentrated again. The residue was redissolved in anhydrous DCM (3 mL, followed by 3 mL, then 1 mL to rinse out the flask) and added dropwise to a solution of 4-chloro-3-nitroaniline (239 mg, 1.39 mmol) and pyridine (0.22 mL, 2.78 mmol) in anhydrous DCM (7 mL). The reaction was stirred for 4 h. The solvent was removed in vacuo, and the resulting residue was taken up in a small volume of MeOH. The solid was precipitated by the addition of water. The precipitate was isolated by filtration, washed well with water, and dried under high vacuum to afford the product as a dark yellow amorphous solid (417 mg, 90%).

A mixture of *N-*(4-chloro-3-nitrophenyl)-2,3-dihydrobenzo[*b*][1,4]dioxine-6-carboxamide (188 mg, 0.562 mmol), ammonium chloride (210 mg, 3.93 mmol), and iron powder (220 mg, 3.93 mmol) in ethanol (2.9 mL) and water (0.95 mL) was heated to reflux overnight. The reaction was allowed to cool to room temperature and filtered through celite, eluting with a mixture of EtOH in EtOAc. The reaction mixture was concentrated in vacuo, and the resulting residue was partitioned between saturated aqueous NaHCO_3_ solution and EtOAc. The organic layer was washed with water and brine, dried over MgSO_4_, and concentrated in vacuo to afford the crude product as a light brown amorphous solid (156 mg, 91%).

##### *N*-(2-Chloro-5-(2,3-dihydrobenzo[*b*][1,4]dioxine-6-carboxamido)phenyl)-2-(2-(pyrrolidin-1-yl)ethoxy)quinoline-6-carboxamide

2-(2-(pyrrolidin-1-yl)ethoxy)quinoline-6-carboxylic acid hydrochloride (75.0 mg, 0.232 mmol) was suspended in thionyl chloride (2 mL), and the reaction mixture was heated to 60 °C for 4 h. The reaction mixture was allowed to cool, and the thionyl chloride was removed in vacuo. The residue was redissolved in anhydrous DCM, and then the solvent was removed in vacuo. This procedure was repeated twice. The acid chloride was resuspended in anhydrous DCM (2 mL), then *N-*(3-amino-4-chlorophenyl)-2,3-dihydrobenzo[*b*][1,4]dioxine-6-carboxamide (78.0 mg, 0.256 mmol), followed by triethylamine (0.16 mL, 1.16 mmol) was added. Not all of the reagents were fully solubilized; therefore, anhydrous dioxane was added (1 mL); however, this did not lead to an improvement. The reaction mixture was allowed to stir at room temperature overnight. The reaction mixture was concentrated in vacuo, and the resulting residue was purified by Isolute SCX-II chromatography (eluting with MeOH, followed by 10% 2 M NH_3_ in MeOH). The crude product was further purified by column chromatography using a gradient of 0–10% MeOH in DCM, followed by purification using preparative TLC eluting with 5% MeOH in DCM. The preparative TLC elution was carried out twice to afford the title compound as a white amorphous solid (0.9 mg, 0.7%). ^1^H NMR (500 MHz, MeOD) δ 8.54 (d, *J* = 2.0 Hz, 1H), 8.37 (d, *J* = 8.9 Hz, 1H), 8.27–8.24 (m, 2H), 7.96 (d, *J* = 8.8 Hz, 1H), 7.64 (dd, *J* = 8.8, 2.5 Hz, 1H), 7.52–7.47 (m, 3H), 7.17 (d, *J* = 8.9 Hz, 1H), 6.96 (d, *J* = 8.3 Hz, 1H), 4.35–4.29 (m, 4H), 3.73–3.67 (m, 2H), 3.49–3.41 (m, 2H), 2.15–2.10 (s, 4H), 1.36–1.30 (m, 4H). HRMS (ESI^+^): calcd for C_31_H_30_^35^ClN_4_O_5_ (M + H)^+^ 573.1905, found 573.1997.

##### *N*-(5-(2,3-Dihydrobenzo[*b*][1,4]dioxine-6-carboxamido)-2-fluorophenyl)-2-(3-(pyrrolidin-1-yl)propoxy)quinoline-6-carboxamide

*N-*(3-Amino-4-fluorophenyl)-2,3-dihydrobenzo[*b*][1,4]dioxine-6-carboxamide (100 mg, 0.347 mmol), 2-(3-(pyrrolidin-1-yl)propoxy)quinoline-6-carboxylic acid (156 mg, 0.520 mmol), and EDC (166 mg, 0.867 mmol) were dissolved in anhydrous DMF (2 mL), and pyridine (0.140 mL, 1.73 mmol) was added dropwise. The reaction mixture was allowed to stir at room temperature for 72 h, then it was poured onto water, and the resulting precipitate was washed with water. The residue was purified by column chromatography using a gradient of 0–10% MeOH in DCM, washed with water, and triturated in diethyl ether to afford the title compound as a beige amorphous solid (30.0 mg, 15%). ^1^H NMR (500 MHz, DMSO-*d*_6_) δ 10.32 (br s, 1H), 10.19 (br s, 1H), 8.59 (d, *J* = 2.21 Hz, 1H), 8.41 (d, *J* = 8.84 Hz, 1H), 8.23 (dd, *J* = 8.84, 2.21 Hz, 1H), 8.13 (dd, *J* = 8.84, 2.21 Hz, 1H), 7.87 (d, *J* = 8.84 Hz, 1H), 7.66–7.61 (m, 1H), 7.55 (d, *J* = 2.21 Hz, 1H), 7.52 (dd, *J* = 8.11, 2.21 Hz, 1H), 7.29 (app t, *J* = 10.23 Hz, 1H), 7.12 (d, *J* = 8.84 Hz, 1H), 6.99 (d, *J* = 8.84 Hz, 1H), 4.53 (t, *J* = 5.57 Hz, 2H), 4.34–4.28 (m, 4H), 3.41 −2.66 (m, 6H), 2.21–2.09 (m, 2H), 1.93–1.80 (m, 4H). ^13^C NMR (126 MHz, DMSO-*d*_6_) δ 165.50, 164.95, 163.36, 151.38 (d, *J* = 244.33 Hz), 148.19, 146.91, 143.41, 140.75, 135.88 (d, *J* = 2.31 Hz), 129.87, 128.95, 128.90, 127.93, 127.25, 125.88 (d, *J* = 12.81 Hz), 124.44, 121.72, 119.30, 119.12 (d, *J* = 9.61 Hz), 117.36, 117.16, 116.03 (d, *J* = 19.22 Hz), 114.49, 64.88, 64.63, 64.49, 53.88, 52.50, 27.57, 23.47. HRMS (ESI^+^): calcd for C_32_H_32_FN_4_O_5_ (M + H)^+^, 571.2351; found 571.2321.

##### *N*-(5-(2,3-Dihydrobenzo[*b*][1,4]dioxine-6-carboxamido)-2-fluorophenyl)-2-(3-(piperidin-1-yl)propoxy)quinoline-6-carboxamide

*N-*(3-Amino-4-fluorophenyl)-2,3-dihydrobenzo[*b*][1,4]dioxine-6-carboxamide (100 mg, 0.347 mmol), 2-(2-(piperidin-1-yl)propoxy)quinoline-6-carboxylic acid (164 mg, 0.52 mmol), and EDC (166 mg, 0.867 mmol) were dissolved in anhydrous DMF (2 mL), and pyridine (0.140 mL, 1.734 mmol) was added dropwise. The resulting mixture was allowed to stir at room temperature for 72 h, then it was poured onto water, and the resulting precipitate was washed with water and purified by column chromatography using a gradient of 0–10% MeOH in DCM, followed by washing in water (5 mL) and trituration in diethyl ether to afford the title compound as a beige amorphous solid (40 mg, 20%). ^1^H NMR (500 MHz, DMSO-*d*_6_) δ 10.32 (br s, 1H), 10.19 (br s, 1H), 8.59 (d, *J* = 1.76 Hz, 1H), 8.39 (d, *J* = 8.79 Hz, 1H), 8.22 (dd, *J* = 8.79, 1.76 Hz, 1H), 8.13 (dd, *J* = 7.03 Hz, 2.34 Hz, 1H), 7.86 (d, *J* = 8.79 Hz, 1H), 7.67–7.62 (m, 1H), 7.55 (d, *J* = 1.76 Hz, 1H), 7.52 (dd, *J* = 8.21, 1.76 Hz, 1H), 7.28 (app t, *J* = 9.96 Hz, 1H), 7.11 (d, *J* = 8.90 Hz, 1H), 6.99 (d, *J* = 8.90 Hz, 1H), 4.51 (t, *J* = 6.29 Hz, 2H), 4.34–4.28 (m, 4H), 3.16–2.65 (m, 4H), 2.36–1.30 (m, 10H). ^13^C NMR (126 MHz, DMSO-*d*_6_) δ 165.51, 164.97, 163.26, 152.37 (d, *J* = 242.81 Hz), 148.18, 146.91, 143.41, 140.77, 135.87 (d, *J* = 2.19 Hz), 129.87, 128.96, 128.89, 127.92, 127.25, 125.88 (d, *J* = 14.51 Hz), 124.45, 121.72, 119.33, 119.14 (d, *J* = 8.29 Hz), 117.36, 117.16, 116.01 (d, *J* = 20.73 Hz), 114.48, 64.87, 64.59, 64.49, 55.03, 53.82, 25.40, 24.99, 23.67. HRMS (ESI^+^): calcd for C_33_H_34_FN_4_O_5_ (M + H)^+^, 585.2508; found 585.2485.

#### Synthetic Route IV

##### 2-Methyl-*N*-(2-methyl-5-nitrophenyl)quinoline-6-carboxamide

To a suspension of 2-methylquinoline-6-carboxylic acid (3.69 g, 19.72 mmol) in anhydrous DCM (35 mL), oxalyl chloride (1.8 mL, 20.15 mmol) and DMF (310 μL, 4.00 mmol) were added dropwise and the resulting green solution was allowed to stir at 20 °C for 3 h after which it was concentrated under vacuum to afford a dry pale green solid. The solid was dissolved in pyridine (35 mL), and 2-methyl-5-nitroaniline (3.0 g, 19.72 mmol) was added in one portion and was allowed to stir for 2 h. The reaction mixture was reduced *in vacuo* until dryness. The remaining residue was triturated with diethyl ether. The crude product was purified by column chromatography on silica gel in gradient DCM/EtOH 0–50% to afford the title compound as a yellow amorphous solid (5.57 g, 88%). ^1^H NMR (500 MHz, DMSO-*d*_6_) δ 10.36 (s, 1H), 8.63 (d, *J* = 1.8 Hz, 1H), 8.43 (d, *J* = 8.4 Hz, 1H), 8.40 (d, *J* = 2.4 Hz, 1H), 8.25 (dd, *J* = 8.7, 2.0 Hz, 1H), 8.07 (d, *J* = 2.9 Hz, 1H), 8.05 (d, *J* = 3.0 Hz, 1H), 7.60 (d, *J* = 8.5 Hz, 1H), 7.54 (d, *J* = 8.4 Hz, 1H), 2.71 (s, 3H), 2.44 (s, 3H). ^13^C NMR (126 MHz, DMSO-*d*_6_) δ 165.54, 161.05, 146.20, 144.90, 143.38, 142.15, 137.76, 132.04, 130.08, 129.93, 129.31, 126.62, 126.09, 124.16, 121.00, 120.94, 23.98, 18.82. HRMS (ESI^+^): calcd for C_18_H_15_NaN_3_O_3_ (M + Na)^+^, 344.1006; found 344.0999.

##### *N*-(5-Amino-2-methylphenyl)-2-methylquinoline-6-carboxamide

2-Methyl-*N*-(2-methyl-5-nitrophenyl)quinoline-6-carboxamide (4.0 g, 12.45 mmol), iron powder (6.95 g, 124.0 mmol), and ammonium chloride (2.12 g, 124.0 mmol) in ethanol (50 mL) and water (12.5 mL) were allowed to stir at 90 °C for 1 h. Then, the reaction mixture was allowed to cool to room temperature and was filtered through a short pad of celite. The eluate was concentrated *in vacuo* to afford the title compound as a light yellow amorphous solid, which was carried onto the next step without purification (1.95 g, 54%). ^1^H NMR (500 MHz, DMSO-*d*_6_) δ 9.86 (s, 1H), 8.61–8.51 (m, 1H), 8.38 (d, *J* = 8.4 Hz, 1H), 8.21 (dd, *J* = 8.8, 1.7 Hz, 1H), 8.00 (d, *J* = 8.8 Hz, 1H), 7.51 (d, *J* = 8.4 Hz, 1H), 6.91 (d, *J* = 8.1 Hz, 1H), 6.65 (d, *J* = 2.0 Hz, 1H), 6.42 (dd, *J* = 8.1, 2.2 Hz, 1H), 4.94 (s, 2H), 2.70 (s, 3H), 2.09 (s, 3H). ^13^C NMR (126 MHz, DMSO-*d*_6_) δ 165.18, 161.09, 148.86, 147.31, 137.58, 137.01, 132.25, 130.87, 128.72, 128.49, 128.38, 125.84, 123.38, 120.72, 112.75, 112.61, 25.49, 17.50. HRMS (ESI^+^): calcd for C_18_H_18_N_3_O (M + H)^+^, 292.1444; found 292.1446.

##### *N*-(5-(2,3-Dihydrobenzo[*b*][1,4]dioxine-6-carboxamido)-2-methylphenyl)-2-methylquinoline-6-carboxamide

2-Methyl-6-quinolinecarboxylic acid (600 mg, 3.21 mmol), HATU (1.46 g, 3.85 mmol), and *N-*(5-amino-2-methylphenyl)-2-methylquinoline-6-carboxamide (910 mg, 3.21 mmol) were suspended in anhydrous DMF (25 mL), and *N,N-*diisopropylethylamine (1.12 mL, 6.41 mmol) was added dropwise. The resulting solution was allowed to stir at room temperature under an inert atmosphere overnight. The reaction mixture was poured onto water, and the resulting precipitate was filtered and washed with water to afford the title compound as an off-white amorphous solid (1.38 g, 95%). ^1^H NMR (500 MHz, DMSO-*d*_6_) δ 10.14 (s, 1H), 10.08 (s, 1H), 8.61 (s, 1H), 8.41 (br d, *J* = 7.3 Hz, 1H), 8.24 (d, *J* = 8.7 Hz, 1H), 8.03 (d, *J* = 8.8 Hz, 1H), 7.87 (d, *J* = 2.1 Hz, 1H), 7.64–7.56 (m, 2H), 7.54 (d, *J* = 2.1 Hz, 1H), 7.51 (dd, *J* = 8.4, 2.2 Hz, 1H), 7.24 (d, *J* = 8.5 Hz, 1H), 6.98 (d, *J* = 8.4 Hz, 1H), 4.35–4.26 (m, 4H), 2.71 (s, 3H), 2.24 (s, 3H). ^13^C NMR (126 MHz, DMSO-*d*_6_) δ 165.44, 164.82, 161.21, 148.93, 146.80, 143.39, 137.77, 137.62, 136.74, 131.97, 130.59, 129.23, 128.81, 128.64, 128.38, 128.14, 125.86, 123.44, 121.66, 119.07, 118.63, 117.30, 117.12, 64.86, 64.48, 25.51, 17.94. HRMS (ESI^+^): calcd for C_27_H_24_N_3_O_4_ (M + H)^+^, 454.1761; found 454.1733.

##### *N*-(5-(2,3-Dihydrobenzo[*b*][1,4]dioxine-6-carboxamido)-2-methylphenyl)-2-formylquinoline-6-carboxamide

A solution of *N-*(5-(2,3-dihydrobenzo[*b*][1,4]dioxine-6-carboxamido)-2-methylphenyl)-2-methylquinoline-6-carboxamide (0.165 g, 0.364 mmol) and selenium dioxide (0.444 g, 0.400 mmol) in anhydrous 1,4-dioxane (0.6 mL) and anhydrous DMF (0.6 mL) was heated at 150 °C for 1 h after which the reaction mixture was allowed to cool to room temperature, diluted with DCM, and filtered through a pad of celite. The filtrate was concentrated under vacuum to afford the crude product as a brown solid, which was taken directly onto the next step without purification (0.17 g). ^1^H NMR (500 MHz, DMSO*-d*_6_) δ 10.29 (s, 1H), 10.17 (s, 1H), 10.09 (s, 1H), 8.81–8.77 (m, 1H), 8.41 (dd, *J* = 8.29, 1.66 Hz, 1H), 8.36 (d, *J* = 8.29 Hz, 1H), 8.17–8.12 (m, 1H), 8.08 (d, *J* = 9.12 Hz, 1H), 7.90 (d, *J* = 2.49 Hz, 1H), 7.58 (dd, *J* = 8.29, 2.49 Hz, 1H), 7.54 (d, *J* = 2.49 Hz, 1H), 7.52 (dd, *J* = 8.29, 2.49 Hz, 1H), 7.25 (d, *J* = 8.29 Hz, 1H), 6.98 (d, *J* = 8.29 Hz, 1H), 4.35–4.28 (m, 4H), 2.31 (s, 3H).

##### *N*-(5-(2,3-Dihydrobenzo[*b*][1,4]dioxine-6-carboxamido)-2-methylphenyl)-2-(piperidin-1-ylmethyl)quinoline-6-carboxamide (**14**)

A solution of *N-*(5-(2,3-dihydrobenzo[*b*][1,4]dioxine-6-carboxamido)-2-methylphenyl)-2-formylquinoline-6-carboxamide (58 mg, 0.124 mmol) and piperidine (31.7 mg, 0.372 mmol) in anhydrous DCM (1.2 mL) was allowed to stir at room temperature for 7 h. Then, sodium triacetoxyborohydride (79 mg, 0.372 mmol) was added in one portion at room temperature and the resulting mixture was allowed to stir under an inert argon atmosphere for 2 h at room temperature. The reaction mixture was diluted with DCM (5 mL), washed with brine (1 × 5 mL), and the aqueous phase was extracted with DCM/MeOH 9:1 mixture (3 × 5 mL). The organic layers were combined, dried over Na_2_SO_4_, filtered, and concentrated under reduced pressure. Purification by column chromatography using a gradient of 0–10% MeOH in DCM afforded the title compound as a pale yellow solid (20 mg, 30%). ^1^H NMR (500 MHz, DMSO-*d*_6_) δ 10.15 (s, 1H), 10.08 (s, 1H), 8.63 (s, 1H), 8.48 (d, *J* = 8.1 Hz, 1H), 8.26 (d, *J* = 9.0 Hz, 1H), 8.08 (d, *J* = 8.6 Hz, 1H), 7.91–7.85 (m, 1H), 7.73 (d, *J* = 8.4 Hz, 1H), 7.61–7.56 (m, 1H), 7.56–7.47 (m, 2H), 7.25 (d, *J* = 8.4 Hz, 1H), 6.98 (d, *J* = 8.4 Hz, 1H), 4.31 (q, *J* = 4.7 Hz, 4H), 3.77 (s, 2H), 2.43 (s, 4H), 2.24 (s, 3H), 1.55 (s, 4H), 1.43 (s, 2H). ^13^C NMR (126 MHz, DMSO-*d*_6_) δ 165.46, 164.94, 163.18, 153.31, 151.37, 148.11, 146.91, 143.41, 140.84, 135.86, 129.96, 129.00, 128.92, 127.92, 127.24, 125.92, 125.81, 124.50, 121.72, 119.34, 119.11, 117.34, 117.17, 116.07, 115.91, 114.41, 64.87, 64.49, 63.99, 53.66, 52.05, 23.28. HRMS (ESI^+^): calcd for C_32_H_32_N_4_NaO_4_ (M + Na)^+^, 559.2316; found 559.2325.

##### *N*-(5-(2,3-Dihydrobenzo[*b*][1,4]dioxine-6-carboxamido)-2-methylphenyl)-2-((4-isopropylpiperazin-1-yl)methyl)quinoline-6-carboxamide (**16**)

To a solution of *N*-(5-(2,3-dihydrobenzo[*b*][1,4]dioxine-6-carboxamido)-2-methylphenyl)-2-formylquinoline-6-carboxamide (170 mg, 0.364 mmol) in anhydrous DCM, 1-isopropylpiperazine (0.16 mL, 1.09 mmol) was added dropwise at room temperature and the resulting mixture was allowed to stir under an inert argon atmosphere for 2.5 h. Then, sodium triacetoxyborohydride (231 mg, 1.09 mmol) was added in one portion and the resulting mixture was allowed to stir overnight at room temperature. The reaction was diluted with DCM (5 mL) and washed with a NaHCO_3_ saturated aqueous solution (5 mL). The aqueous phase was extracted with DCM (3 × 5 mL), and the combined organic layers were dried over Na_2_SO_4_, filtered, and concentrated under reduced pressure to afford a brown oil as a crude product. Purification by column chromatography using a gradient of 0–10% MeOH in DCM afforded the title compound as a beige solid (30 mg, 14%). ^1^H NMR (500 MHz, DMSO-*d*_6_) δ 10.15 (s, 1H), 10.08 (s, 1H), 8.65–8.61 (m, 1H), 8.48 (d, J = 8.6 Hz, 1H), 8.29–8.23 (m, 1H), 8.08 (d, J = 8.8 Hz, 1H), 7.90–7.86 (m, 1H), 7.72 (d, J = 8.5 Hz, 1H), 7.59 (dd, J = 8.3, 2.0 Hz, 1H), 7.56–7.48 (m, 2H), 7.25 (d, J = 8.4 Hz, 1H), 6.98 (d, J = 8.4 Hz, 1H), 4.31 (q, J = 5.0 Hz, 4H), 3.78 (s, 2H), 2.65–2.59 (m, 1H), 2.47 (s, 8H), 2.24 (s, 3H), 0.96 (d, J = 6.5 Hz, 6H). ^13^C NMR (126 MHz, DMSO-d_6_) δ 165.37, 164.82, 162.08, 148.63, 146.81, 143.39, 137.89, 137.78, 136.69, 132.37, 130.60, 129.22, 129.15, 128.63, 128.41, 128.14, 128.09, 126.69, 122.21, 121.66, 119.06, 118.64, 117.30, 117.12, 64.92, 64.86, 64.49, 54.05, 53.94, 48.47, 31.16, 28.11, 18.71, 17.93. HRMS (ESI^+^): calcd for C_34_H_38_N_5_O_4_ (M + H)^+^, 580.2918; found 580.2896.

##### *N*-(2-Chloro-5-(2,3-dihydrobenzo[*b*][1,4]dioxine-6-carboxamido)phenyl)-2-formylquinoline-6-carboxamide

To a suspension of 2-methylquinoline-6-carboxylic acid (1.5 g, 8.01 mmol) in anhydrous DCM (40 mL), DMF (1.40 μL, 0.018 mmol) and oxalyl chloride (0.74 mL, 8.74 mmol) were added dropwise and the resulting green solution was allowed to stir at 20 °C for 3 h, after which it was concentrated under vacuum to afford a dry pale green solid. The solid was dissolved in pyridine (40 mL), and 2-chloro-5-nitroaniline (1.26 g, 7.28 mmol) was added in one portion. The resulting dark yellow suspension was allowed to stir for 2 h, after which it was poured onto water and the yellow precipitate was filtered and washed several times with water, diethyl ether, and finally with a minimum amount of DCM to afford the product as a yellow amorphous solid, which was used without further purification (2.20 g, 88%). ^1^H NMR (500 MHz, DMSO*-d*_6_) δ 10.59 (s, 1H), 8.65 (d, *J* = 2.0 Hz, 1H), 8.60 (d, *J* = 2.7 Hz, 1H), 8.44 (d, *J* = 8.6 Hz, 1H), 8.25 (dd, *J* = 8.6, 2.0 Hz, 1H), 8.15 (dd, *J* = 8.6, 2.7 Hz, 1H), 8.06 (d, *J* = 8.4 Hz, 1H), 7.91 (d, *J* = 8.6 Hz, 1H), 7.55 (d, *J* = 8.0 Hz, 1H), 2.71 (s, 3H). HRMS (ESI^+^): calcd for C_17_H_13_^35^ClN_3_O_3_ (M + H)^+^, 342.0640; found 342.0646.

*N-*(2-Chloro-5-nitrophenyl)-2-methylquinoline-6-carboxamide was suspended in water (7 mL) and EtOH (21 mL). Ammonium chloride (2.41 g, 45.1 mmol) and iron powder (2.52 g, 45.1 mmol) were added, and the resulting suspension was allowed to stir at 90 °C for 1 h. The reaction mixture was allowed to cool to room temperature, diluted with MeOH and DCM, and filtered through a pad of celite. The resulting filtrate was concentrated under vacuum to afford a light brown amorphous solid as a crude product, which was used directly in the next step without purification (2.00 g, 100%). ^1^H NMR (500 MHz, DMSO-*d*_6_) δ 9.96 (s, 1H), 8.58 (d, *J* = 2.2 Hz, 1H), 8.41 (d, *J* = 8.7 Hz, 1H), 8.21 (dd, *J* = 8.7, 2.2 Hz, 1H), 8.02 (d, *J* = 8.7 Hz, 1H), 7.53 (d, *J* = 7.6 Hz, 1H), 7.15 (d, *J* = 8.7 Hz, 1H), 6.87 (d, *J* = 2.2 Hz, 1H), 6.50 (dd, *J* = 8.7, 2.2 Hz, 1H), 5.41 (bs, 2H), 2.70 (s, 3H). HRMS (ESI^+^): calcd for C_17_H_15_^35^ClN_3_O (M + H)^+^, 312.0898; found 312.0902.

2,3-Dihydrobenzo[*b*][1,4]dioxine-6-carboxylic acid (1.27 g, 7.06 mmol) was suspended in anhydrous DCM (20 mL), and DMF (1.23 μL, 0.016 mmol) and oxalyl chloride (0.65 mL, 7.70 mmol) were added dropwise, and the resulting green solution was allowed to stir at 20 °C for 3 h after which it was concentrated under vacuum to afford a dry pale green solid. The solid was dissolved in pyridine (20.0 mL), and *N-*(5-amino-2-chlorophenyl)-2-methylquinoline-6-carboxamide (2.00 g, 6.42 mmol) was added in one portion. The resulting dark yellow suspension was allowed to stir for 2 h after which it was poured onto water, and the yellow precipitate was filtered and washed several times with water, diethyl ether, and finally with a minimum amount of DCM to afford the crude product as a pale yellow amorphous solid, which was carried onto the next step without purification (1.86 g, 61%). ^1^H NMR (500 MHz, DMSO-*d*_6_) δ 10.31 (s, 1H), 10.27 (s, 1H), 8.63 (d, *J* = 1.5 Hz, 1H), 8.43 (d, *J* = 8.8 Hz, 1H), 8.25 (dd, *J* = 8.8, 2.2 Hz, 1H), 8.14 (d, *J* = 2.2 Hz, 1H), 8.04 (d, *J* = 8.8 Hz, 1H), 7.75 (dd, *J* = 8.8, 2.9 Hz, 1H), 7.58 – 7.49 (m, 4H), 7.00 (d, *J* = 8.8 Hz, 1H), 4.37–4.26 (m, 4H), 2.71 (s, 3H). HRMS (ESI^+^): calcd for C_26_H_21_^35^ClN_3_O_4_ (M + H)^+^, 474.1215; found 474.1210.

A solution of *N-*(2-chloro-5-(2,3-dihydrobenzo[*b*][1,4]dioxine-6-carboxamido)phenyl)-2-methylquinoline-6-carboxamide (0.500 g, 1.06 mmol) and selenium dioxide (0.129 g, 1.16 mmol) in anhydrous DMF (12.0 mL) and 1,4-dioxane (12.0 mL) was heated at 150 °C for 2 h. A further portion of selenium dioxide (0.129 g, 1.16 mmol) was added to the reaction mixture and stirred at 150 °C for a further 1 h. The reaction mixture was allowed to cool to room temperature, diluted with DCM, and filtered through a pad of celite. The filtrate was concentrated under vacuum to afford the crude product as a yellow amorphous solid, which was carried onto the next step without purification (0.515 g). ^1^H NMR (500 MHz, DMSO-*d*_6_) δ 10.45 (s, 1H), 10.40 (s, 1H), 10.17 (s, 1H), 8.81–8.79 (m, 2H), 8.42–8.34 (m, 2H), 8.19 (app t, *J* = 7.47 Hz, 1H), 8.09 (d, *J* = 8.13 Hz, 1H), 7.79–7.74 (m, 2H), 7.55 (d, *J* = 1.99 Hz, 1H), 7.52 (dd, *J* = 8.13, 1.99 Hz, 1H), 6.99 (d, *J* = 9.04 Hz, 1H), 4.36–4.26 (m, 4H). HRMS (ESI^+^): calcd for C_26_H_19_^35^ClN_3_O_5_ (M + H)^+^, 488.1013; found 488.1012.

##### *N*-(2-Chloro-5-(2,3-dihydrobenzo[*b*][1,4]dioxine-6-carboxamido)phenyl)-2-((4-isopropylpiperazin-1-yl)methyl)quinoline-6-carboxamide (**18**)

A solution of *N*-(2-chloro-5-(2,3-dihydrobenzo[*b*][1,4]dioxine-6-carboxamido)phenyl)-2-formylquinoline-6-carboxamide (2.00 g, 4.10 mmol) and 1-isopropylpiperazine (1.58 g, 12.3 mmol) in anhydrous DCM (35 mL) was allowed to stir at 20 °C for 6 h, after which sodium triacetoxyborohydride (2.61 g, 12.3 mmol) was added in one portion, and the resulting mixture was allowed to stir at 20 °C for 2 h. The reaction was quenched with a NaHCO_3_ saturated aqueous solution (35 mL) and extracted with a DCM/MeOH 9/1 mixture (3 × 35 mL). The crude product (pale yellow solid) was purified by column chromatography using a gradient of 0–10% MeOH in DCM, followed by trituration in diethyl ether to afford the title compound as a pale yellow amorphous solid (0.830 g, 34%). ^1^H NMR (500 MHz, DMSO-*d*_6_) δ 10.33 (s, 1H), 10.28 (s, 1H), 8.66 (s, 1H), 8.50 (d, J = 8.5 Hz, 1H), 8.27 (d, J = 8.8 Hz, 1H), 8.20–8.04 (m, 2H), 7.81–7.64 (m, 2H), 7.62–7.46 (m, 3H), 7.00 (d, J = 8.4 Hz, 1H), 4.31 (q, J = 5.1 Hz, 4H), 3.81 (s, 2H), 2.59 (d, J = 43.9 Hz, 5H), 2.45 (br s, 4H), 0.99 (s, 6H). ^13^C NMR (126 MHz, DMSO-d_6_) δ 165.45, 165.11, 161.95, 148.75, 147.03, 143.43, 139.06, 138.02, 135.35, 131.83, 129.81, 129.29, 128.89, 128.37, 127.76, 126.70, 124.04, 122.31, 121.80, 120.31, 119.72, 117.37, 117.23, 64.88, 64.49, 60.05, 53.76, 48.34, 18.44. HRMS (ESI^+^): calcd for C_33_H_35_^35^ClN_5_O_4_ (M + H)^+^, 600.2372; found 600.2336.

##### *N*-(2-Chloro-5-(2,3-dihydrobenzo[*b*][1,4]dioxine-6-carboxamido)phenyl)-2-(piperazin-1-ylmethyl)quinoline-6-carboxamide (**19**)

A solution of *N-*(2-chloro-5-(2,3-dihydrobenzo[*b*][1,4]dioxine-6-carboxamido)phenyl)-2-formylquinoline-6-carboxamide (0.300 g, 0.615 mmol) and 1-(*tert*-butyl)piperazine (0.262 g, 1.85 mmol) in anhydrous DCM (5 mL) was allowed to stir at 20 °C for 12 h, after which sodium triacetoxyborohydride (0.391 g, 1.85 mmol) was added in one portion, and the resulting mixture was allowed to stir at 20 °C for 2 h. The reaction was quenched with a NaHCO_3_ saturated aqueous solution (5 mL) and extracted with a DCM/MeOH 9/1 mixture (3 × 5 mL). The crude product (pale yellow solid) was purified by column chromatography 0–10% MeOH in DCM, followed by trituration in diethyl ether to afford *tert-*butyl 4-((6-((2-chloro-5-(2,3-dihydrobenzo[*b*][1,4]dioxine-6-carboxamido)phenyl)carbamoyl)quinolin-2-yl)methyl)piperazine-1-carboxylate as a beige solid (0.060 g, 16%). ^1^H NMR (500 MHz, DMSO*-d*_6_): δ 10.34 (s, 1H), 10.29 (s, 1H), 8.66 (d, *J* = 1.6 Hz, 1H), 8.50 (d, *J* = 8.6 Hz, 1H), 8.26 (dd, *J* = 8.6, 2.4 Hz, 1H), 8.14 (d, *J* = 2.4 Hz, 1H), 8.09 (d, *J* = 8.6 Hz, 1H), 7.77–7.72 (m, 2H), 7.56 (d, *J* = 2.4 Hz, 1H), 7.54 (d, *J* = 1.6 Hz, 1H), 7.53 (dd, *J* = 7.8, 2.4 Hz, 1H), 7.00 (d, *J* = 8.6 Hz, 1H), 4.35–4.27 (m, 4H), 3.79 (s, 2H), 2.79–2.29 (m, 8H), 1.03 (br s, 9H). To a suspension of *tert-*butyl 4-((6-((2-chloro-5-(2,3-dihydrobenzo[*b*][1,4]dioxine-6-carboxamido)phenyl)carbamoyl)quinolin-2-yl)methyl)piperazine-1-carboxylate (674 mg, 1.02 mmol) in anhydrous DCM (10 mL), trifluoroacetic acid (TFA, 0.78 mL, 10.2 mmol) was added dropwise, and the resulting mixture was allowed to stir at 20 °C for 4 h. The reaction mixture was concentrated under vacuum to afford the crude product as a light brown oil. The crude was purified by column chromatography using a gradient of 0–15% MeOH in DCM, followed by trituration in diethyl ether to afford the title compound as a yellow amorphous solid (39 mg, 6.8%). ^1^H NMR (500 MHz, DMSO-*d*_6_) δ 10.32 (s, 1H), 10.27 (s, 1H), 8.65 (d, *J* = 2.0 Hz, 1H), 8.49 (d, *J* = 8.4 Hz, 1H), 8.26 (dd, *J* = 8.8, 2.0 Hz, 1H), 8.15 (d, *J* = 2.5 Hz, 1H), 8.09 (d, *J* = 8.8 Hz, 1H), 7.76–7.71 (m, 2H), 7.58–7.47 (m, 3H), 7.00 (d, *J* = 8.4 Hz, 1H), 4.40–4.23 (m, 4H), 3.76 (s, 2H), 2.74 (t, *J* = 4.8 Hz, 4H), 2.47–2.42 (br m, 4H) (1 proton missing). ^13^C NMR (126 MHz, DMSO-*d*_6_) δ 165.00, 164.64, 161.79, 148.29, 146.57, 142.97, 138.58, 137.45, 134.89, 131.32, 129.36, 128.81, 128.39, 127.85, 127.31, 126.21, 123.54, 121.85, 121.32, 119.81, 119.23, 116.92, 116.74, 65.11, 64.42, 64.03, 54.26, 45.54. HRMS (ESI^+^): calcd for C_30_H_29_^35^ClN_5_O_4_ (M + H)^+^ 558.1903, found 558.1885.

##### *N*-(2-Chloro-5-(2,3-dihydrobenzo[*b*][1,4]dioxine-6-carboxamido)phenyl)-2-((4-methylpiperazin-1-yl)methyl)quinoline-6-carboxamide (**20**)

A solution of *N*-(2-chloro-5-(2,3-dihydrobenzo[*b*][1,4]dioxine-6-carboxamido)phenyl)-2-formylquinoline-6-carboxamide (0.100 g, 0.205 mmol) and 1-methylpiperazine (61.6 mg, 0.615 mmol) in anhydrous DCM (2 mL) was allowed to stir at 20 °C for 12 h, after which sodium triacetoxyborohydride (0.130 g, 0.615 mmol) was added in one portion, and the resulting mixture was allowed to stir at 20 °C for 2 h. The reaction was quenched with a NaHCO_3_ saturated aqueous solution (5 mL) and extracted with a DCM/MeOH 9/1 mixture (3 × 5 mL). The crude product (brown oil) was purified by column chromatography using a gradient of 0–10% MeOH in DCM to afford the desired product as a white amorphous solid (0.010 g, 8.5%). ^1^H NMR (500 MHz, DMSO-*d*_6_) δ 10.31 (s, 1H), 10.23 (s, 1H), 8.65 (s, 1H), 8.48 (d, *J* = 8.7 Hz, 1H), 8.30–8.26 (m, 1H), 8.15 (s, 1H), 8.07 (d, *J* = 8.8 Hz, 1H), 7.71 (dd, *J* = 8.7, 4.2 Hz, 2H), 7.57–7.47 (m, 3H), 6.99 (d, *J* = 8.4 Hz, 1H), 4.31 (q, *J* = 4.9 Hz, 4H), 3.79 (s, 2H), 2.35 (br s, 8H), 2.16 (s, 3H). ^13^C NMR (126 MHz, DMSO-d_6_) δ 165.48, 165.11, 162.14, 148.72, 147.03, 143.44, 139.02, 138.00, 129.80, 129.24, 128.83, 128.41, 127.79, 126.70, 124.01, 122.27, 121.80, 120.25, 119.56, 117.39, 117.22, 117.22, 64.89, 64.84, 64.49, 55.21, 53.40, 46.24. HRMS (ESI^+^): calcd for C_31_H_31_^35^ClN_5_O_4_ (M + H)^+^, 572.2059; found 572.2031.

##### *N*-(2-Chloro-5-(2,3-dihydrobenzo[*b*][1,4]dioxine-6-carboxamido)phenyl)-2-((4-ethylpiperazin-1-yl)methyl)quinoline-6-carboxamide (**21**)

A solution of *N*-(2-chloro-5-(2,3-dihydrobenzo[*b*][1,4]dioxine-6-carboxamido)phenyl)-2-formylquinoline-6-carboxamide (1.03 g, 2.11 mmol) and 1-ethylpiperazine (723 mg, 6.33 mmol) in anhydrous DCM (20 mL) was allowed to stir at 20 °C for 12 h, after which sodium triacetoxyborohydride (1.34 g, 6.33 mmol) was added in one portion, and the resulting mixture was allowed to stir at 20 °C for 3 h. The reaction was quenched with a NaHCO_3_ saturated aqueous solution (20 mL) and extracted with a DCM/MeOH 9/1 mixture (3 × 20 mL). The crude product was purified by column chromatography using a gradient of 0–10% MeOH in DCM, followed by purification by Isolute SCX-II chromatography eluting with MeOH/NH_3_ to afford the title compound as a white amorphous solid (0.431 g, 35%). ^1^H NMR (500 MHz, DMSO-*d*_6_) δ 10.32 (s, 1H), 10.27 (s, 1H), 8.65 (d, *J* = 2.0 Hz, 1H), 8.49 (d, *J* = 8.4 Hz, 1H), 8.26 (dd, *J* = 8.8, 2.0 Hz, 1H), 8.20–8.04 (m, 2H), 7.80–7.66 (m, 2H), 7.62–7.46 (m, 3H), 7.00 (d, *J* = 8.4 Hz, 1H), 4.39–4.23 (m, 4H), 3.79 (s, 2H), 2.51–2.29 (m, 10H), 0.98 (t, *J* = 7.2 Hz, 3H). ^13^C NMR (126 MHz, DMSO-*d*_6_) δ 165.45, 165.11, 162.24, 148.74, 147.03, 143.43, 139.05, 138.01, 135.34, 131.83, 129.82, 129.29, 128.88, 128.36, 127.77, 126.70, 124.02, 122.32, 121.79, 120.29, 119.71, 117.38, 117.21, 64.88, 64.49, 54.09, 53.86, 48.38, 18.71. HRMS (ESI^+^): calcd for C_32_H_33_^35^ClN_5_O_4_ (M + H)^+^, 586.2216; found 586.2189.

##### 2-((4-(*tert*-Butyl)piperazin-1-yl)methyl)-*N*-(2-chloro-5-(2,3-dihydrobenzo[*b*][1,4]dioxine-6-carboxamido)phenyl)quinoline-6-carboxamide

A solution of *N*-(2-chloro-5-(2,3-dihydrobenzo[*b*][1,4]dioxine-6-carboxamido)phenyl)-2-formylquinoline-6-carboxamide (300 mg, 0.615 mmol) and 1-(*tert*-butyl)piperazine (262 mg, 1.85 mmol) in anhydrous DCM (5 mL) was allowed to stir at 20 °C for 12 h, after which sodium triacetoxyborohydride (391 mg, 1.85 mmol) was added in one portion, and the resulting mixture was allowed to stir at 20 °C for 2 h. The reaction was quenched with a NaHCO_3_ saturated aqueous solution (5 mL) and extracted with a DCM/MeOH 9/1 mixture (3 × 5 mL). The crude product (pale yellow solid) was purified by column chromatography using a gradient of 0–10% MeOH in DCM followed by washing in water and trituration in diethyl ether to afford the title compound as a beige amorphous solid (60 mg, 16%). ^1^H NMR (500 MHz, DMSO-*d*_6_) δ 10.34 (s, 1H), 10.29 (s, 1H), 8.66 (d, *J* = 1.56 Hz, 1H), 8.50 (d, *J* = 8.60 Hz, 1H), 8.26 (dd, *J* = 8.60, 2.35 Hz, 1H), 8.14 (d, *J* = 2.35 Hz, 1H), 8.09 (d, *J* = 8.60 Hz, 1H), 7.77–7.72 (m, 2H), 7.56 (d, *J* = 2.35 Hz, 1H), 7.54 (d, *J* = 1.56 Hz, 1H), 7.53 (dd, *J* = 7.82, 2.35 Hz, 1H), 7.00 (d, *J* = 8.60 Hz, 1H), 4.35–4.27 (m, 4H), 3.79 (s, 2H), 2.79–2.29 (m, 8H), 1.03 (br s, 9H). ^13^C NMR (126 MHz, DMSO-*d*_6_) δ 165.46, 165.11, 162.21, 148.75, 147.03, 143.43, 139.05, 137.95, 135.35, 131.79, 129.82, 129.28, 128.86, 128.33, 127.77, 126.69, 124.00, 122.29, 121.79, 120.27, 119.69, 117.38, 117.21, 64.88, 64.76, 64.49, 54.13, 45.77, 26.05. HRMS (ESI^+^): calcd for C_34_H_37_^35^ClN_5_O_4_ (M + H)^+^, 614.2529; found 614.2502.

##### *N*-(2-Chloro-5-(2,3-dihydrobenzo[*b*][1,4]dioxine-6-carboxamido)phenyl)-2-((4-ethyl-1,4-diazepan-1-yl)methyl)quinoline-6-carboxamide

A suspension of *N*-(2-chloro-5-(2,3-dihydrobenzo[*b*][1,4]dioxine-6-carboxamido)phenyl)-2-formylquinoline-6-carboxamide (103 mg, 0.211 mmol) and 1-ethyl-1,4-diazepane (0.12 mL, 108 mg, 0.844 mmol) in anhydrous DCM (4.5 mL sonication and a large volume of DCM were used in an attempt to fully solubilize the starting material) was allowed to stir at room temperature overnight. Then, sodium triacetoxyborohydride (179 mg, 0.844 mmol) was added and the reaction mixture was stirred at room temperature for 40 h. The reaction was quenched with saturated aqueous NaHCO_3_ solution. The aqueous layer was extracted three times with a mixture of 10% MeOH in DCM; the combined organic layer was dried (MgSO_4_) and concentrated in vacuo. This crude product was purified by Biotage chromatography using a gradient of 0–10% MeOH in DCM with a KPNH_2_ column to afford a yellow oil. This material was further purified by Isolute SCX-II column chromatography (eluting with MeOH, followed by 10% of 2 M NH_3_ in MeOH) to give a yellow gum. Finally, the gum was triturated with diethyl ether to afford a yellow amorphous solid (9 mg, 7%). ^1^H NMR (500 MHz, DMSO-*d*_6_) δ 10.31 (s, 1H), 10.26 (s, 1H), 8.65 (s, 1H), 8.49 (d, *J* = 8.5 Hz, 1H), 8.25 (d, *J* = 8.8 Hz, 1H), 8.14 (d, *J* = 2.0 Hz, 2H), 8.08 (d, *J* = 8.6 Hz, 1H), 7.75 (dd, *J* = 8.9, 2.2 Hz 1H), 7.57–7.48 (m, 3H), 7.00 (d, *J* = 8.4 Hz, 1H), 4.36–4.26 (m, 4H), 3.94 (s, 2H), 2.78–2.60 (m, 6H), 2.55–2.45 (m, 2H–hidden under DMSO peak, observed by HSQC), 1.75 (p, *J* = 5.8 Hz, 4H), 0.99 (t, *J* = 7.0 Hz, 3H). ^13^C NMR (126 MHz, DMSO-*d*_6_) δ 165.47, 165.11, 163.34, 148.72, 147.03, 143.43, 139.04, 137.91, 135.36, 131.73, 129.82, 129.26, 128.84, 128.29, 127.77, 126.67, 124.00, 122.20, 121.79, 120.27, 119.68, 117.38, 117.21, 64.88, 64.71, 64.49, 55.71, 54.81, 54.60, 53.77, 51.92, 27.75, 13.03. HRMS (ESI^+^): calcd for C_33_H_35_^35^ClN_5_O_4_ (M + H)^+^ 600.2372, found 600.2351.

##### *N*-(2-Chloro-5-(2,3-dihydrobenzo[*b*][1,4]dioxine-6-carboxamido)phenyl)-2-((4-cyclopropylpiperazin-1-yl)methyl)quinoline-6-carboxamide

A solution of *N*-(2-chloro-5-(2,3-dihydrobenzo[*b*][1,4]dioxine-6-carboxamido)phenyl)-2-formylquinoline-6-carboxamide (255 mg, 0.523 mmol) and 1-cyclopropylpiperazine (0.189 mL, 1.568 mmol) in anhydrous DCM (5 mL) was allowed to stir at 20 °C for 12 h, after which sodium triacetoxyborohydride (332 mg, 1.568 mmol) was added in one portion, and the resulting mixture was allowed to stir at 20 °C for 2 h. The reaction was quenched with a NaHCO_3_ saturated aqueous solution (5 mL) and extracted with a DCM/MeOH 9/1 mixture (3 × 5 mL). The crude product (brown solid) was purified by two rounds of column chromatography using a gradient of 0–10% MeOH in DCM followed by trituration in diethyl ether to afford the title compound as a pale yellow amorphous solid (44 mg, 14%). ^1^H NMR (500 MHz, DMSO-*d*_6_) δ 10.32 (s, 1H), 10.27 (s, 1H), 8.65 (s, 1H), 8.50 (d, *J* = 7.87 Hz, 1H), 8.26 (d, *J* = 7.87 Hz, 1H), 8.15 (d, *J* = 1.83 Hz, 1H), 8.09 (d, *J* = 7.87 Hz, 1H), 7.77–7.71 (m, 2H), 7.57–7.49 (m, 3H), 7.00 (d, *J* = 7.87 Hz, 1H), 4.36–4.24 (m, 4H), 3.78 (s, 2H), 2.57 (br s, 4H), 2.43 (br s, 4H), 1.63–1.58 (m, 1H), 0.42–0.37 (m, 2H), 0.30–0.24 (m, 2H). ^13^C NMR (126 MHz, DMSO-*d*_6_) δ 165.47, 165.12, 162.22, 148.75, 147.05, 143.45, 139.06, 138.01, 135.36, 131.81, 129.84, 129.30, 128.87, 128.34, 127.78, 126.70, 124.02, 122.31, 121.80, 120.29, 119.70, 117.39, 117.22, 64.88, 64.49, 53.46, 53.25, 30.89, 6.09. HRMS (ESI^+^): calcd for C_33_H_33_^35^ClN_5_O_4_ (M + H)^+^, 598.2216; found 598.2210.

##### 2-((4-(*sec*-Butyl)piperazin-1-yl)methyl)-*N*-(2-chloro-5-(2,3-dihydrobenzo[*b*][1,4]dioxine-6-carboxamido)phenyl)quinoline-6-carboxamide

A solution of *N*-(2-chloro-5-(2,3-dihydrobenzo[*b*][1,4]dioxine-6-carboxamido)phenyl)-2-formylquinoline-6-carboxamide (255 mg, 0.523 mmol) and 1-(*sec*-butyl)piperazine (223 mg, 1.57 mmol) in anhydrous DCM (5 mL) was allowed to stir at 20 °C for 12 h, after which sodium triacetoxyborohydride (332 mg, 1.57 mmol) was added in one portion, and the resulting mixture was allowed to stir at 20 °C for 2 h. The reaction was quenched with a NaHCO_3_ saturated aqueous solution (5 mL) and extracted with a DCM/MeOH 9/1 mixture (3 × 5 mL). The crude product (brown solid) was purified by two rounds of column chromatography using a gradient of 0–10% MeOH in DCM, followed by trituration in diethyl ether to afford the title compound as a pale yellow amorphous solid (35 mg, 11%). ^1^H NMR (500 MHz, DMSO-*d*_6_) δ 10.32 (s, 1H), 10.27 (s, 1H), 8.66 (d, *J* = 1.6 Hz, 1H), 8.50 (d, *J* = 8.4 Hz, 1H), 8.26 (dd, *J* = 8.4, 1.60 Hz, 1H), 8.15 (d, *J* = 2.3 Hz, 1H), 8.09 (d, *J* = 8.4 Hz, 1H), 7.75 (dd, *J* = 8.4, 2.36 Hz, 1H), 7.74 (d, *J* = 8.4 Hz, 1H), 7.55 (d, *J* = 2.3 Hz, 1H), 7.54 (d, *J* = 1.6 Hz, 1H), 7.52 (dd, *J* = 7.8, 2.3 Hz, 1H), 7.00 (d, *J* = 8.4 Hz, 1H), 4.35–4.27 (m, 4H), 3.81 (s, 2H), 2.87–2.17 (m, 9H), 1.51 (br s, 1H), 1.28 (br s, 1H), 0.95 (br s, 3H), 0.85 (t, *J* = 7.65 Hz, 3H). ^13^C NMR (126 MHz, DMSO-*d*_6_) δ 165.46, 165.11, 162.20, 148.75, 147.04, 143.44, 139.05, 137.98, 135.35, 131.80, 129.83, 129.29, 128.88, 128.34, 127.77, 126.69, 124.01, 122.30, 121.80, 120.28, 119.70, 117.39, 117.22, 64.89, 64.49, 60.25, 53.91, 48.15, 26.05, 14.08, 11.51. HRMS (ESI^+^): calcd for C_34_H_37_^35^ClN_5_O_4_ (M + H)^+^, 614.2529; found 614.2495.

##### (*R*)-*N*-(2-Chloro-5-(2,3-dihydrobenzo[*b*][1,4]dioxine-6-carboxamido)phenyl)-2-((4-ethyl-2-methylpiperazin-1-yl)methyl)quinoline-6-carboxamide

A suspension of *N*-(2-chloro-5-(2,3-dihydrobenzo[*b*][1,4]dioxine-6-carboxamido)phenyl)-2-formylquinoline-6-carboxamide (1.21 g, 2.48 mmol) and (*R*)-*tert*-butyl 3-methylpiperazine-1-carboxylate (0.993 g, 4.96 mmol) in anhydrous DCM (24 mL) was allowed to stir at room temperature overnight. Then, sodium triacetoxyborohydride (1.05 mg, 4.96 mmol) was added and the reaction mixture was stirred at room temperature overnight. The reaction was quenched with saturated aqueous NaHCO_3_ solution. The aqueous layer was extracted three times with a mixture of 10% MeOH in DCM; the combined organic layer was dried (Na_2_SO_4_) and concentrated in vacuo. The crude product was purified by Biotage chromatography using a gradient of 0–10% MeOH in DCM to afford the product as a yellow semisolid (560 mg, 34%). (*R*)-*tert*-Butyl 4-((6-((2-chloro-5-(2,3-dihydrobenzo[*b*][1,4]dioxine-6-carboxamido)phenyl)carbamoyl)quinolin-2-yl)methyl)-3-methylpiperazine-1-carboxylate (560 mg, 0.83 mmol) was taken up in anhydrous DCM (8 mL), and TFA (0.319 mL, 4.17 mmol) was added dropwise. The reaction was allowed to stir at room temperature for 48 h, after which time the starting material was still observed by LCMS. Therefore, further TFA (0.319 mL, 4.17 mmol) was added, and the reaction mixture was left to stir for 18 h. The solvents were removed in vacuo, and the resulting solid was taken up in 10% MeOH in DCM. The organic layer was washed twice with saturated aqueous NaHCO_3_ solution, followed by water, dried (Na_2_SO_4_), and then concentrated in vacuo. The material was partially purified by Isolute SCX-II column chromatography (eluting with MeOH, followed by 10% of 2 M NH_3_ in MeOH) to afford the crude product as a yellow solid (272 mg, ∼73% purity).

To a solution of (*R*)-*N*-(2-chloro-5-(2,3-dihydrobenzo[*b*][1,4]dioxine-6-carboxamido)phenyl)-2-((2-methylpiperazin-1-yl)methyl)quinoline-6-carboxamide (100 mg, 0.128 mmol) in anhydrous MeOH (1.5 mL) at 0 °C was added sodium cyanoborohydride (8.82 mg, 0.140 mmol) in one portion. Acetaldehyde (5.02 μL, 0.089 mmol) was then added as a cooled solution (0 °C) in MeOH (0.5 mL) dropwise for 2 min. The reaction was allowed to warm slowly to room temperature and left to stir for 48 h. The MeOH was removed in vacuo, and the resulting residue was taken up in 10% MeOH in DCM. The organic layer was washed with 1 M NaOH aqueous solution and dried (Na_2_SO_4_). The resulting residue was purified by Biotage chromatography using a gradient of 0–10% MeOH in DCM and then further purified by Isolute SCX-II chromatography (eluting with MeOH, followed by 10% of 2 M NH_3_ in MeOH) to afford the product as a yellow amorphous solid (33 mg, 43%). ^1^H NMR (500 MHz, CDCl_3_) δ 8.63 (s, 1H), 8.60 (d, J = 2.5 Hz, 1H), 8.41 (d, *J* = 1.8 Hz, 1H), 8.26 (d, *J* = 8.5 Hz, 1H), 8.19 (d, *J* = 8.8 Hz, 1H), 8.16 (dd, *J* = 8.8, 2.0 Hz, 1H), 8.07 (s, 1H), 7.99 (dd, *J* = 8.8, 2.5 Hz, 1H), 7.81 (d, *J* = 8.5 Hz, 1H), 7.46 (d, *J* = 1.8 Hz, 1H), 7.45 (d, *J* = 4.8 Hz, 1H), 7.39 (dd, *J* = 8.4, 2.2 Hz, 1H), 6.95 (d, *J* = 8.4 Hz, 1H), 4.35 (d, *J* = 14.5 Hz, 1H), 4.34–4.29 (m, 4H), 3.63 (d, *J* = 14.6 Hz, 1H), 2.83 (br. d, *J* = 10.9 Hz, 1H), 2.81–2.75 (m, 2H), 2.72–2.63 (m, 1H), 2.53–2.37 (m, 3H), 2.21–2.14 (m, 1H), 2.01 (br t, *J* = 10.5 Hz, 1H), 1.19 (d, *J* = 6.2 Hz, 3H), 1.11 (t, *J* = 7.2 Hz, 3H). ^13^C NMR (126 MHz, CDCl_3_) δ 165.05, 164.92, 163.62, 149.06, 146.96, 143.58, 137.94, 137.18, 134.67, 131.65, 130.02, 129.51, 127.79, 127.74, 126.72, 122.27, 120.48, 117.81, 117.46, 116.89, 116.79, 112.45, 77.23, 64.58, 64.19, 60.73, 60.66, 55.85, 53.00, 52.58, 52.26, 17.84, 11.91. HRMS (ESI^+^): calcd for C_33_H_35_^35^ClN_5_O_4_ (M + H)^+^ 600.2327, found 600.2352.

##### 2-(Azetidin-1-ylmethyl)-*N*-(2-chloro-5-(2,3-dihydrobenzo[*b*][1,4]dioxine-6-carboxamido)phenyl)quinoline-6-carboxamide

A solution of *N-*(2-chloro-5-(2,3-dihydrobenzo[*b*][1,4]dioxine-6-carboxamido)phenyl)-2-formylquinoline-6-carboxamide (300 mg, 0.615 mmol) and azetidine (0.124 mL, 1.84 mmol) in anhydrous DCM (2.0 mL) was allowed to stir at 20 °C for 12 h, after which sodium triacetoxyborohydride (391 mg, 1.84 mmol) was added in one portion, and the resulting mixture was allowed to stir at 20 °C for 2 h. The reaction mixture was quenched with a NaHCO_3_ saturated aqueous solution (2 mL) and extracted with a mixture of DCM/MeOH 9/1 (3 × 2 mL). The crude product was purified by column chromatography on silica gel using a gradient of 0–15% MeOH in DCM and then washed with water and triturated with diethyl ether to afford the title compound as a pale yellow amorphous solid (20 mg, 6%). ^1^H NMR (500 MHz, DMSO-*d*_6_) δ 10.35 (s, 1H), 10.31 (s, 1H), 8.66 (d, *J* = 2.2 Hz, 1H), 8.49 (d, *J* = 8.7 Hz, 1H), 8.27 (dd, *J* = 8.7, 2.2 Hz, 1H), 8.14 (d, *J* = 2.2 Hz, 1H), 8.08 (d, *J* = 8.7 Hz, 1H), 7.76 (dd, *J* = 8.7, 2.2 Hz, 1H), 7.65 (d, *J* = 7.6 Hz, 1H), 7.58–7.51 (m, 3H), 6.99 (d, *J* = 8.7 Hz, 1H), 4.35–4.27 (m, 4H), 3.91 (s, 2H), 3.39–3.27 (m, 4H), 2.06 (qn, *J* = 7.10 Hz, 2H). ^13^C NMR (126 MHz, DMSO-*d*_6_) δ 165.47, 165.11, 161.47, 148.75, 147.04, 143.43, 139.05, 138.09, 135.35, 131.80, 129.83, 129.27, 128.90, 128.41, 127.77, 126.62, 124.02, 121.91, 121.80, 120.28, 119.70, 117.39, 117.22, 65.18, 64.89, 64.49, 55.42, 49.06, 17.92. HRMS (ESI^+^): calcd for C_29_H_26_^35^ClN_4_O_4_ (M + H)^+^, 529.1637; found 529.1616.

##### *N*-(2-Chloro-5-(2,3-dihydrobenzo[*b*][1,4]dioxine-6-carboxamido)phenyl)-2-(pyrrolidin-1-ylmethyl)quinoline-6-carboxamide

A solution of *N-*(2-chloro-5-(2,3-dihydrobenzo[*b*][1,4]dioxine-6-carboxamido)phenyl)-2-formylquinoline-6-carboxamide (300 mg, 0.615 mmol) and pyrrolidine (131 mg, 1.84 mmol) in anhydrous DCM (2 mL) was allowed to stir at 20 °C for 12 h, after which sodium triacetoxyborohydride (391 mg, 1.84 mmol) was added in one portion, and the resulting mixture was allowed to stir at 20 °C for 2 h. The resulting mixture was quenched with a NaHCO_3_ saturated aqueous solution (2 mL) and extracted with a mixture of DCM/MeOH 9/1 (3 × 2 mL). The crude product was purified by column chromatography using a gradient of 0–15% MeOH in DCM, then washed with water and triturated with diethyl ether to afford the title compound as a pale yellow amorphous solid (20 mg, 6%). ^1^H NMR (500 MHz, DMSO-*d*_6_) δ 10.32 (s, 1H), 10.27 (s, 1H), 8.65 (s, 1H), 8.49 (d, *J* = 7.98 Hz, 1H), 8.26 (dd, *J* = 7.98, 2.28 Hz, 1H), 8.14 (d, *J* = 2.28 Hz, 1H), 8.09 (d, *J* = 7.98 Hz, 1H), 7.77–7.70 (m, 2H), 7.56–7.50 (m, 3H), 7.00 (d, *J* = 7.98 Hz, 1H), 4.35–4.28 (m, 4H), 3.92 (s, 2H), 2.57–2.52 (m, 4H), 1.77–1.70 (m, 4H). ^13^C NMR (126 MHz, DMSO-*d*_6_) δ 165.49, 165.11, 162.75, 148.72, 147.04, 143.44, 139.05, 137.98, 135.36, 131.77, 129.83, 129.30, 128.86, 128.33, 127.77, 126.66, 124.01, 122.29, 121.79, 120.27, 119.69, 117.39, 117.22, 64.89, 64.49, 62.45, 54.29, 23.77. HRMS (ESI^+^): calcd for C_30_H_28_^35^ClN_4_O_4_ (M + H)^+^, 543.1794; found 543.1778.

##### *N*-(2-Chloro-5-(2,3-dihydrobenzo[*b*][1,4]dioxine-6-carboxamido)phenyl)-2-((3-methylazetidin-1-yl)methyl)quinoline-6-carboxamide

A solution of 3-methylazetidine hydrochloride (33.1 mg, 0.307 mmol) in anhydrous DCM (0.5 mL) was added to a stirring solution of *N-*(2-chloro-5-(2,3-dihydrobenzo[*b*][1,4]dioxine-6-carboxamido)phenyl)-2-formylquinoline-6-carboxamide (50 mg, 0.102 mmol) in anhydrous DCM (1.5 mL) at room temperature. The reaction was stirred for 5.5 h, then NaBH(OAc)_3_ (65.2 mg, 0.307 mmol) was added, and the reaction mixture was stirred for 48 h. The reaction was quenched with saturated aqueous NaHCO_3_ solution. The aqueous layer was extracted with 3 × 10% MeOH in DCM. The combined organic layer was dried (Na_2_SO_4_) and concentrated in vacuo. The residue was purified by Biotage chromatography using a gradient of 0–10% MeOH in DCM to afford a yellow gum. This gum was further purified by Isolute SCX-II chromatography (eluting with MeOH and then 10% of 2 M NH_3_ in MeOH) to afford the title product as a yellow amorphous solid (16.5 mg, 30%). ^1^H NMR (500 MHz, CDCl_3_) δ 8.63 (br s, 1H), 8.58 (d, *J* = 2.5 Hz, 1H), 8.41 (d, *J* = 1.8 Hz, 1H), 8.28 (d, *J* = 8.5 Hz, 1H), 8.20 (d, *J* = 8.8 Hz, 1H), 8.16 (dd, *J* = 8.8, 2.0 Hz, 1H), 7.98 (dd, *J* = 8.8, 2.4 Hz, 2H), 7.64 (d, *J* = 8.5 Hz, 1H), 7.46 (s, 1H), 7.45 (d, *J* = 7.2 Hz, 1H), 7.39 (dd, *J* = 8.4, 2.2 Hz, 1H), 6.96 (d, *J* = 8.4 Hz, 1H), 4.38–4.29 (m, 4H), 3.99 (s, 2H), 3.64 (t, *J* = 7.5 Hz, 2H), 2.95 (t, *J* = 7.2 Hz, 2H), 2.70 (dt, *J* = 14.0, 7.0 Hz, 1H), 1.21 (d, *J* = 6.8 Hz, 3H). HRMS (ESI^+^): calcd for C_30_H_28_^35^ClN_4_O_4_ (M + H)^+^ 543.1794, found 543.1769.

##### *N*-(2-Chloro-5-(2,3-dihydrobenzo[*b*][1,4]dioxine-6-carboxamido)phenyl)-2-((3,3-dimethylazetidin-1-yl)methyl)quinoline-6-carboxamide

3,3-Dimethylazetidine (55.0 mg, 0.646 mmol) was added to a stirring solution of *N*-(2-chloro-5-(2,3-dihydrobenzo[*b*][1,4]dioxine-6-carboxamido)phenyl)-2-formylquinoline-6-carboxamide (210 mg, 0.431 mmol) in anhydrous DCM (1.5 mL) at room temperature under an inert atmosphere. The reaction was stirred for 1 h. Then, NaBH(OAc)_3_ (137 mg, 0.646 mmol) was added, and the reaction mixture was stirred for 18 h. The reaction was quenched with saturated aqueous NaHCO_3_ solution (4 mL). The aqueous layer was extracted with DCM (3 × 4 mL); the combined organic layer was dried (Na_2_SO_4_) and concentrated in vacuo. The residue was purified by column chromatography using a gradient of 0–10% MeOH in DCM to afford a yellow gum. This gum was further purified by Isolute SCX-II chromatography (eluting with MeOH and then 10% NH_3_ in MeOH) to afford the title product as a yellow amorphous solid (16.0 mg, 7%). ^1^H NMR (500 MHz, DMSO-*d*_6_) δ 10.35 (s, 1H), 10.27 (s, 1H), 8.70–8.66 (m, 1H), 8.55 (d, *J* = 8.5 Hz, 1H), 8.29 (dd, *J* = 9.0, 2.0 Hz, 1H), 8.17–8.09 (m, 2H), 7.74 (dd, *J* = 8.8, 2.6 Hz, 1H), 7.65 (d, *J* = 8.5 Hz, 1H), 7.57–7.49 (m, 3H), 7.00 (d, *J* = 8.4 Hz, 1H), 4.36–4.27 (m, 4H), 3.45–3.20 (m, 6H), 1.27 (s, 6H). δ HRMS (ESI^+^): calcd for C_31_H_30_^37^ClN_4_O_4_ (M + H)^+^ 559.1937, found 559.1921.

##### 2-(Azetidin-1-ylmethyl)-*N*-(5-(2,3-dihydrobenzo[*b*][1,4]dioxine-6-carboxamido)-2-fluorophenyl)quinoline-6-carboxamide

To a solution of *N-*(5-(2,3-dihydrobenzo[*b*][1,4]dioxine-6-carboxamido)-2-fluorophenyl)-2-formylquinoline-6-carboxamide (100 mg, 0.212 mmol) in anhydrous DCM (2 mL), azetidine (43 μL, 0.636 mmol) was added dropwise at room temperature, and the resulting mixture was allowed to stir for 12 h. Then, sodium triacetoxyborohydride (135 mg, 0.636 mmol) was added in one portion and the resulting mixture was allowed to stir for 2 h at room temperature. The reaction mixture was washed with brine (2 mL) and purified by column chromatography using a gradient of 0–10% MeOH in DCM + 1% 7 N NH_3_ in MeOH to afford the title compound as a white amorphous solid (10 mg, 9%). ^1^H NMR (500 MHz, MeOD) δ 8.59 (d, *J* = 2.1 Hz, 1H), 8.48 (d, *J* = 8.5 Hz, 1H), 8.29 (dd, *J* = 8.8, 2.1 Hz, 1H), 8.24–8.10 (m, 2H), 7.71–7.55 (m, 2H), 7.55–7.44 (m, 2H), 7.24 (dd, *J* = 10.1, 8.9 Hz, 1H), 6.96 (d, *J* = 8.4 Hz, 1H), 4.38–4.26 (m, 4H), 3.99 (s, 2H), 3.47 (t, *J* = 7.1 Hz, 4H), 2.21 (qn, *J* = 7.2 Hz, 2H). ^13^C NMR (126 MHz, MeOD) δ 166.69, 166.60, 160.42, 152.36 (d, *J* = 248.54 Hz), 148.53, 147.10, 143.47, 138.18, 134.84 (d, *J* = 2.22 Hz), 131.94, 128.36, 128.18, 127.96, 127.38, 126.66, 125.28 (d, *J* = 11.11 Hz), 121.58, 120.75, 119.56 (d, *J* = 6.67 Hz), 118.95, 116.79, 116.64, 115.23 (d, *J* = 20.01 Hz), 64.53, 64.13, 55.13, 29.36, 17.33. HRMS (ESI^+^): calcd for C_29_H_26_FN_4_O_4_ (M + H)^+^, 513.1933; found 513.1930.

##### *N*-(5-(2,3-Dihydrobenzo[*b*][1,4]dioxine-6-carboxamido)-2-fluorophenyl)-2-(pyrrolidin-1-ylmethyl)quinoline-6-carboxamide

To a solution of *N-*(5-(2,3-dihydrobenzo[*b*][1,4]dioxine-6-carboxamido)-2-fluorophenyl)-2-formylquinoline-6-carboxamide (100 mg, 0.212 mmol) in anhydrous DCM (2 mL), pyrrolidine (53 μL, 0.636 mmol) was added dropwise at room temperature, and the resulting mixture was allowed to stir for 12 h. Then, sodium triacetoxyborohydride (135 mg, 0.636 mmol) was added in one portion and the resulting mixture was allowed to stir for 2 h at room temperature. The reaction mixture was washed with brine (2 mL) and purified by column chromatography using a gradient of 0–10% MeOH in DCM + 1% 7 N NH_3_ in MeOH to afford the title compound as a pale yellow amorphous solid (55 mg, 49%). ^1^H NMR (500 MHz, DMSO*-d*_6_) δ 10.39 (s, 1H), 10.18 (s, 1H), 8.64 (d, *J* = 1.8 Hz, 1H), 8.48 (d, *J* = 8.5 Hz, 1H), 8.24 (dd, *J* = 8.8, 1.9 Hz, 1H), 8.14 (dd, *J* = 7.0, 2.6 Hz, 1H), 8.08 (d, *J* = 8.8 Hz, 1H), 7.72 (d, *J* = 8.5 Hz, 1H), 7.66 (ddd, *J* = 8.9, 4.1, 2.8 Hz, 1H), 7.57–7.49 (m, 2H), 7.30 (t, *J* = 9.5 Hz, 1H), 6.99 (d, *J* = 8.4 Hz, 1H), 4.31 (q, *J* = 5.1 Hz, 4H), 3.92 (s, 2H), 2.50 (br s, 4H), 1.75 (br s, 4H). ^13^C NMR (126 MHz, DMSO-*d*_6_) δ 165.52, 164.96, 162.79, 152.32 (d, *J* = 245.73 Hz), 148.72, 146.93, 143.43, 137.98, 135.93 (d, *J* = 2.12 Hz), 131.72, 129.23, 128.97, 128.44, 127.93, 126.62, 125.83 (d, *J* = 13.69 Hz), 122.28, 121.73, 119.27, 119.17 (d, *J* = 7.12 Hz), 117.36, 117.16, 116.03 (d, *J* = 20.87 Hz), 64.88, 64.50, 62.47, 54.29, 23.77. HRMS (ESI^+^): calcd for C_30_H_28_FN_4_O_4_ (M + H)^+^, 527.2089; found 527.2074.

##### *N*-(5-(2,3-Dihydrobenzo[*b*][1,4]dioxine-6-carboxamido)-2-fluorophenyl)-2-(piperidin-1-ylmethyl)quinoline-6-carboxamide

To a solution of *N-*(5-(2,3-dihydrobenzo[*b*][1,4]dioxine-6-carboxamido)-2-fluorophenyl)-2-formylquinoline-6-carboxamide (100 mg, 0.212 mmol) in anhydrous DCM (2 mL), piperidine (63 μL, 0.636 mmol) was added dropwise at room temperature, and the resulting mixture was allowed to stir under an inert argon atmosphere for 7 h. Then, sodium triacetoxyborohydride (135 mg, 0.636 mmol) was added in one portion and the resulting mixture was allowed to stir for 2 h at room temperature. The reaction mixture was washed with brine (2 mL) and purified by column chromatography using a gradient of 0–10% MeOH in DCM + 1% 7 N NH_3_ in MeOH to afford the title compound as a pale yellow amorphous solid (50 mg, 44%). ^1^H NMR (500 MHz, DMSO*-d*_6_) δ 10.39 (s, 1H), 10.18 (s, 1H), 8.64 (s, 1H), 8.48 (d, *J* = 8.6 Hz, 1H), 8.24 (d, *J* = 8.7 Hz, 1H), 8.11 (dd, *J* = 30.3, 9.0 Hz, 2H), 7.74 (d, *J* = 8.7 Hz, 1H), 7.67–7.59 (m, 1H), 7.58–7.46 (m, 2H), 7.30 (t, *J* = 9.8 Hz, 1H), 6.99 (d, *J* = 9.5 Hz, 1H), 4.31 (d, *J* = 5.6 Hz, 4H), 3.75 (s, 2H), 2.42 (br s, 4H), 1.53 (br s, 4H), 1.42 (br s, 2H). ^13^C NMR (126 MHz, DMSO*-d*_6_) δ 165.49, 164.94, 162.63, 152.34 (d, *J* = 248.13 Hz), 148.72, 146.91, 143.41, 137.92, 135.89 (d, *J* = 2.18 Hz), 131.68, 129.18, 128.96, 128.41, 127.92, 126.62, 125.81 (d, *J* = 10.90 Hz), 122.19, 121.71, 119.35, 119.18 (d, *J* = 8.17 Hz), 117.35, 117.15, 116.03 (d, *J* = 21.79 Hz), 65.65, 64.87, 64.49, 54.79, 26.09, 24.29. HRMS (ESI^+^): calcd for C_31_H_30_FN_4_O_4_ (M + H)^+^, 541.2246; found 541.2242.

##### *N*-(5-(2,3-Dihydrobenzo[*b*][1,4]dioxine-6-carboxamido)-2-fluorophenyl)-2-((2-methylpyrrolidin-1-yl)methyl)quinoline-6-carboxamide

2-Fluoro-5-nitroaniline (31.7 mg, 0.203 mmol), 2-((2-methylpyrrolidin-1-yl)methyl)quinoline-6-carboxylic acid hydrochloride (62.4 mg, 0.203 mmol), and EDC (78 mg, 0.406 mmol) were dissolved in anhydrous DMF (2 mL), and pyridine (0.082 mL, 1.02 mmol) was added dropwise. The mixture was allowed to stir at 20 °C for 17 h. The reaction mixture was washed with NaHCO_3_ saturated aqueous solution, extracted with DCM/MeOH 9/1 mixture, and dried over Na_2_SO_4_. The crude product was purified by column chromatography using a gradient of 0–10% MeOH in DCM + 1% 7 N NH_3_ in MeOH to afford the product as a yellow solid, which was carried directly onto the next step (83 mg). LCMS (ESI^+^): *m*/*z* = 409.1655, (M + H)^+^. *N-*(2-Fluoro-5-nitrophenyl)-2-((2-methylpyrrolidin-1-yl)methyl)quinoline-6-carboxamide (83 mg, 0.203 mmol), ammonium chloride (76 mg, 1.42 mmol), and iron powder (79 mg, 1.42 mmol) were combined and suspended in EtOH (3 mL) and water (1 mL) at room temperature, affording a beige suspension, which was heated at 90 °C for 1 h. The reaction mixture was cooled to room temperature and filtered through a pad of celite to remove the iron (eluting with EtOH/DCM/MeOH). The solvents were then removed in vacuo. The resulting residue was dried to afford a pale beige solid as a crude product, which was taken onto the next step without purification (77 mg). LCMS (ESI^+^), *m*/*z* = 379.1918, (M + H)^+^.

*N-*(5-Amino-2-fluorophenyl)-2-((2-methylpyrrolidin-1-yl)methyl)quinoline-6-carboxamide (77 mg, 0.203 mmol), 2,3-dihydrobenzo[*b*][1,4]dioxine-6-carboxylic acid (36.7 mg, 0.203 mmol), and EDC (98 mg, 0.509 mmol) were dissolved in anhydrous DMF (1.5 mL); then, pyridine (82 μL, 1.02 mmol) was added dropwise and the resulting mixture was allowed to stir at 20 °C for 72 h. The reaction mixture was washed with water (2 mL) and extracted with a DCM/MeOH 9/1 mixture (3 × 5 mL) to afford a pale yellow solid as a crude product, which was purified by flash column chromatography using a gradient of 0–10% MeOH in DCM + 1% 7 N NH_3_ in MeOH to afford a yellow solid as a semicrude product, which was then repurified by semipreparative TLC (10% MeOH in DCM) to afford the title compound as a pale yellow amorphous solid (30 mg, 27%). ^1^H NMR (500 MHz, MeOD) δ 8.58 (d, *J* = 1.9 Hz, 1H), 8.46 (d, *J* = 8.5 Hz, 1H), 8.27 (dd, *J* = 8.8, 2.0 Hz, 1H), 8.22–8.11 (m, 2H), 7.78 (d, *J* = 8.5 Hz, 1H), 7.58 (ddd, *J* = 8.9, 4.2, 2.7 Hz, 1H), 7.51–7.44 (m, 2H), 7.26–7.18 (m, 1H), 6.94 (d, *J* = 8.3 Hz, 1H), 4.37–4.26 (m, 5H), 3.63 (d, *J* = 13.6 Hz, 1H), 3.00 (ddd, *J* = 10.1, 7.6, 3.5 Hz, 1H), 2.64 (dq, *J* = 13.5, 6.2 Hz, 1H), 2.37 (q, *J* = 9.0 Hz, 1H), 2.11–2.00 (m, 1H), 1.77 (dtd, *J* = 12.5, 9.1, 7.9, 3.2 Hz, 2H), 1.52 (tdd, *J* = 9.9, 7.3, 4.6 Hz, 1H), 1.22 (d, *J* = 6.1 Hz, 3H). ^13^C NMR (126 MHz, MeOD) δ 166.70, 166.61, 165.65, 162.27, 152.47 (d, *J* = 244.5 Hz), 148.38, 147.12, 143.49, 137.99, 134.88 (d, *J* = 2.5 Hz), 131.93, 128.19, 127.94, 127.40, 126.73, 125.31 (d, *J* = 12.4 Hz), 122.37, 120.76, 119.54 (d, *J* = 9.1 Hz), 118.93, 116.80, 116.65, 115.23 (d, *J* = 19.1 Hz), 64.53, 64.14, 60.40, 59.89, 54.17, 32.13, 21.14, 17.67. HRMS (ESI^+^): calcd for C_31_H_30_FN_4_O_4_ (M + H)^+^, 541.2246; found 541.2236.

##### *N*-(5-(2,3-Dihydrobenzo[*b*][1,4]dioxine-6-carboxamido)-2-fluorophenyl)-2-((3-methylazetidin-1-yl)methyl)quinoline-6-carboxamide

A solution of *N*-(5-(2,3-dihydrobenzo[*b*][1,4]dioxine-6-carboxamido)-2-fluorophenyl)-2-formylquinoline-6-carboxamide (0.25 g, 0.530 mmol) and 3-methylazetidine (113 mg, 1.591 mmol) in anhydrous DCM (4 mL) was allowed to stir at 20 °C for 18 h, after which sodium triacetoxyborohydride (0.337 g, 1.591 mmol) was added in one portion, and the resulting mixture was allowed to stir at 20 °C for 3 h. The reaction was quenched with a NaHCO_3_ saturated aqueous solution and extracted with a mixture of DCM/MeOH 9/1 (3 × 5 mL). Purification by column chromatography using a gradient of 0–10% MeOH in DCM, followed by further purification by Isolute SCX-II cartridge with MeOH/NH_3_, afforded the title compound as a pale yellow amorphous solid (114 mg, 41%). ^1^H NMR (500 MHz, DMSO-*d*_6_) δ 10.46 (s, 1H), 10.20 (s, 1H), 8.71 (d, *J* = 1.7 Hz, 1H), 8.59 (d, *J* = 8.5 Hz, 1H), 8.32 (dd, *J* = 8.8, 1.9 Hz, 1H), 8.21–8.08 (m, 2H), 7.64 (ddd, *J* = 7.6, 4.3, 2.8 Hz, 2H), 7.58–7.44 (m, 2H), 7.38–7.23 (m, 1H), 6.99 (d, *J* = 8.4 Hz, 1H), 4.66 (s, 2H), 4.43–4.22 (m, 4H), 4.27–4.02 (m, 2H), 3.71 (s, 2H), 2.95–2.76 (m, 1H), 1.23 (d, *J* = 6.4 Hz, 3H). ^13^C NMR (126 MHz, DMSO-*d*_6_) δ 165.48, 164.95, 161.35, 152.37 (d, *J* = 244.0 Hz), 148.71, 146.91, 143.41, 138.17, 135.90 (d, *J* = 1.6 Hz), 131.77, 129.20, 129.02, 128.56, 127.93, 126.60, 125.80 (d, *J* = 14.0 Hz), 121.89, 121.72, 119.28, 119.23 (d, *J* = 7.8 Hz), 117.35, 117.17, 116.07 (d, *J* = 20.4 Hz), 66.85, 64.88, 64.49, 25.90, 21.53, 19.29. HRMS (ESI^+^): calcd for C_30_H_28_FN_4_O_4_ (M + H)^+^, 527.2068; found 527.2089.

##### *N*-(5-(2,3-Dihydrobenzo[*b*][1,4]dioxine-6-carboxamido)-2-fluorophenyl)-2-((3,3-dimethylazetidin-1-yl)methyl)quinoline-6-carboxamide

A solution of *N*-(5-(2,3-dihydrobenzo[*b*][1,4]dioxine-6-carboxamido)-2-fluorophenyl)-2-formylquinoline-6-carboxamide (0.25 g, 0.530 mmol) and 3,3-dimethylazetidine (135 mg, 1.59 mmol) in anhydrous DCM (4 mL) was allowed to stir at 20 °C for 18 h, after which sodium triacetoxyborohydride (0.337 g, 1.59 mmol) was added in one portion, and the resulting mixture was allowed to stir at 20 °C for 3 h. The reaction was quenched with a NaHCO_3_ saturated aqueous solution and extracted with a mixture of DCM/MeOH 9/1 (3 × 5 mL). Purification by column chromatography using a gradient of 0–10% MeOH in DCM, followed by further purification by Isolute SCX-II cartridge with MeOH/NH_3_, afforded the title compound as a pale yellow solid (156 mg, 54%). ^1^H NMR (500 MHz, DMSO-*d*_6_) δ 10.40 (s, 1H), 10.19 (s, 1H), 8.66 (d, *J* = 2.0 Hz, 1H), 8.51 (d, *J* = 8.5 Hz, 1H), 8.26 (dd, *J* = 8.7, 2.1 Hz, 1H), 8.18–8.06 (m, 2H), 7.69–7.62 (m, 2H), 7.57–7.49 (m, 2H), 7.29 (dd, *J* = 10.1, 9.0 Hz, 1H), 6.99 (d, *J* = 8.4 Hz, 1H), 4.35–4.27 (m, 4H), 4.07 (br s, 2H), 3.22 (br s, 4H), 1.24 (s, 6H). ^13^C NMR (126 MHz, DMSO-*d*_6_) δ 165.46, 164.95, 161.71, 152.28 (d, *J* = 250.1 Hz), 148.68, 146.92, 143.41, 138.23, 135.90, 135.88 (d, *J* = 2.1 Hz), 131.83, 129.20, 129.03, 128.61, 127.92, 126.62, 125.80 (d, *J* = 15.0 Hz), 121.88, 121.72, 119.28, 119.21 (d, *J* = 6.0 Hz), 117.35, 117.16, 116.10 (d, *J* = 21.0 Hz), 66.80, 64.88, 64.49, 32.19, 27.33. HRMS (ESI^+^): calcd for C_31_H_30_FN_4_O_4_ (M + H)^+^, 541.2246; found 541.2240.

##### *N*-(5-(2,3-Dihydrobenzo[*b*][1,4]dioxine-6-carboxamido)-2-fluorophenyl)-2-(piperazin-1-ylmethyl)quinoline-6-carboxamide

A solution of *N-*(5-(2,3-dihydrobenzo[*b*][1,4]dioxine-6-carboxamido)-2-fluorophenyl)-2-formylquinoline-6-carboxamide (255 mg, 0.541 mmol) and *tert-*butyl piperazine-1-carboxylate (302 mg, 1.62 mmol) in anhydrous DCM (5 mL) was allowed to stir at 20 °C for 12 h, after which sodium triacetoxyborohydride (344 mg, 1.62 mmol) was added in one portion, and the resulting mixture was allowed to stir at 20 °C for 2 h. The reaction was quenched with a NaHCO_3_ saturated aqueous solution (5 mL) and extracted with a DCM/MeOH 9/1 mixture (3 × 5 mL). The crude product (pale yellow solid) was purified by column chromatography using a gradient of 0–6% MeOH in DCM, followed by trituration in diethyl ether to afford the desired product as a pale beige amorphous solid (325 mg, 94%). ^1^H NMR (500 MHz, DMSO*-d*_6_) δ 10.39 (s, 1H), 10.18 (s, 1H), 8.65 (d, *J* = 2.0 Hz, 1H), 8.50 (d, *J* = 8.6 Hz, 1H), 8.25 (dd, *J* = 8.8, 2.1 Hz, 1H), 8.19–8.06 (m, 2H), 7.75 (d, *J* = 8.5 Hz, 1H), 7.65 (ddd, *J* = 9.0, 4.3, 2.6 Hz, 1H), 7.57–7.49 (m, 2H), 7.30 (dd, *J* = 10.1, 9.0 Hz, 1H), 6.99 (d, *J* = 8.5 Hz, 1H), 4.35–4.27 (m, 4H), 3.82 (s, 2H), 3.33 (br s, 4H), 2.44 (br s, 4H), 1.40 (s, 9H). ^13^C NMR (126 MHz, DMSO*-d*_6_) δ 165.46, 164.94, 161.81, 154.27, 152.26 (d, *J* = 245.8 Hz), 148.76, 146.91, 143.41, 138.07, 135.89 (d, *J* = 2.9 Hz), 131.80, 129.23, 128.99, 128.49, 127.92, 126.66, 125.83 (d, *J* = 12.1 Hz), 122.30, 121.71, 119.27, 119.18 (d, *J* = 7.89 Hz), 117.35, 117.15, 116.02 (d, *J* = 21.0 Hz), 79.26, 64.87, 64.69, 64.49, 53.18, 28.52.

To a suspension of *tert-*butyl 4-((6-((5-(2,3-dihydrobenzo[*b*][1,4]dioxine-6-carboxamido)-2-fluorophenyl)carbamoyl)quinolin-2-yl)methyl)piperazine-1-carboxylate (300 mg, 0.468 mmol) in anhydrous DCM (5 mL), TFA (0.179 mL, 2.338 mmol) was added dropwise, and the resulting mixture was allowed to stir at 20 °C for 3 h. The reaction mixture was concentrated under reduced pressure to afford the crude product as a light brown oil. The crude was purified by column chromatography using a gradient of 0–15% MeOH in DCM followed by trituration in diethyl ether to afford the title compound as an amorphous white solid (174 mg, 68.7%). ^1^H NMR (500 MHz, DMSO*-d*_6_) δ 10.41 (s, 1H), 10.20 (s, 1H), 8.73 (br s, 1H), 8.67 (d, *J* = 1.7 Hz, 1H), 8.52 (d, *J* = 8.7 Hz, 1H), 8.27 (dd, *J* = 8.7, 1.7 Hz, 1H), 8.15 (dd, *J* = 7.1, 2.7 Hz, 1H), 8.09 (d, *J* = 8.7 Hz, 1H), 7.74 (d, *J* = 8.4 Hz, 1H), 7.66–7.61 (m, 1H), 7.54 (d, *J* = 2.1 Hz, 1H), 7.52 (dd, *J* = 8.4, 2.1 Hz, 1H), 7.30 (app t, *J* = 10.1 Hz, 1H), 6.99 (d, *J* = 8.4 Hz, 1H), 4.35–4.27 (m, 4H), 3.89 (s, 2H), 3.19–3.09 (m, 4H), 2.76–2.66 (m, 4H). ^13^C NMR (126 MHz, DMSO-*d*_6_) δ 164.98, 164.50, 160.59, 151.87 (d, *J* = 243.8 Hz), 148.27, 146.47, 142.96, 137.76, 135.45 (d, *J* = 2.7 Hz), 131.45, 128.79, 128.57, 128.13, 127.46, 126.25, 125.30 (d, *J* = 13.2 Hz), 121.95, 121.26, 118.86, 118.78 (d, *J* = 7.8 Hz), 116.90, 116.70, 115.59 (d, *J* = 20.9 Hz), 64.42, 64.04, 63.67, 49.45, 43.03. HRMS (ESI^+^): calcd for C_30_H_29_FN_5_O_4_ (M + H)^+^, 542.2198; found 542.2190.

##### *N*-(5-(2,3-Dihydrobenzo[*b*][1,4]dioxine-6-carboxamido)-2-fluorophenyl)-2-((4-methylpiperazin-1-yl)methyl)quinoline-6-carboxamide

A solution of *N*-(5-(2,3-dihydrobenzo[*b*][1,4]dioxine-6-carboxamido)-2-fluorophenyl)-2-formylquinoline-6-carboxamide (5.15 g, 10.92 mmol) and 1-methylpiperazine (3.28 g, 32.8 mmol) in anhydrous DCM (90 mL) was allowed to stir at 20 °C for 18 h, after which sodium triacetoxyborohydride (6.95 g, 32.8 mmol) was added in one portion, and the resulting mixture was allowed to stir at 20 °C for 1.5 h. The reaction was quenched with NaHCO_3_ aqueous saturated solution (50 mL) and extracted with a DCM/MeOH 9/1 mixture (3 × 50 mL). Purification by column chromatography on silica gel using a gradient of 0–20% MeOH in DCM followed by washing in water and trituration in diethyl ether afforded the title compound as an amorphous white solid (2.85 g, 47%). ^1^H NMR (500 MHz, DMSO-*d*_6_) δ 10.40 (s, 1H), 10.19 (s, 1H), 8.65 (d, *J* = 1.7 Hz, 1H), 8.49 (d, *J* = 8.4 Hz, 1H), 8.25 (dd, *J* = 8.4, 1.7 Hz, 1H), 8.14 (dd, *J* = 7.6, 2.5 Hz, 1H), 8.08 (d, *J* = 8.8 Hz, 1H), 7.72 (d, *J* = 8.4 Hz, 1H), 7.68–7.63 (m, 1H), 7.55 (d, *J* = 2.5 Hz, 1H), 7.52 (dd, *J* = 8.4, 1.7 Hz, 1H), 7.29 (app t, *J* = 9.2 Hz, 1H), 6.99 (d, *J* = 9.2 Hz, 1H), 4.37–4.27 (m, 4H), 3.79 (s, 2H), 2.54–2.27 (m, 8H), 2.16 (br s, 3H). ^13^C NMR (126 MHz, DMSO-*d*_6_) δ 165.49, 164.96, 162.20, 152.38 (d, *J* = 243.33 Hz), 148.74, 146.93, 143.42, 138.01, 135.89 (d, *J* = 2.33 Hz), 131.75, 129.22, 128.99, 128.46, 127.93, 126.66, 125.80 (d, *J* = 13.1 Hz), 122.28, 121.73, 119.31, 119.18 (d, *J* = 6.7 Hz), 117.36, 117.17, 116.04 (d, *J* = 20.6 Hz), 64.87, 64.49, 55.21, 53.41, 46.24. HRMS (ESI^+^): calcd for C_31_H_31_FN_5_O_4_ (M + H)^+^, 556.2355; found 556.2329.

##### 2-((4-(*sec*-Butyl)piperazin-1-yl)methyl)-*N*-(5-(2,3-dihydrobenzo[*b*][1,4]dioxine-6-carboxamido)-2-fluorophenyl)quinoline-6-carboxamide

A solution of *N*-(5-(2,3-dihydrobenzo[*b*][1,4]dioxine-6-carboxamido)-2-fluorophenyl)-2-formylquinoline-6-carboxamide (100 mg, 0.212 mmol) and 1-(*sec*-butyl)piperazine (0.103 mL, 0.636 mmol) in anhydrous DCM (2 mL) was allowed to stir at 20 °C for 6 h, after which sodium triacetoxyborohydride (135 mg, 0.636 mmol) was added in one portion, and the resulting mixture was allowed to stir at 20 °C for 2 h. The reaction was quenched with a NaHCO_3_ (5 mL) aqueous saturated solution and extracted with a DCM/MeOH 9/1 mixture (3 × 5 mL). Purification by column chromatography on silica gel using a gradient of 0–10% MeOH in DCM followed by trituration in diethyl ether afforded the title compound as an amorphous pale pink solid (51 mg, 40%). ^1^H NMR (500 MHz, DMSO-*d*_6_) δ 10.39 (s, 1H), 10.19 (s, 1H), 8.65 (d, *J* = 1.7 Hz, 1H), 8.49 (d, *J* = 8.5 Hz, 1H), 8.25 (dd, *J* = 8.5, 1.7 Hz, 1H), 8.14 (dd, *J* = 6.8, 2.6 Hz, 1H), 8.08 (d, *J* = 9.1 Hz, 1H), 7.73 (d, *J* = 8.5 Hz, 1H), 7.68–7.63 (m, 1H), 7.54 (d, *J* = 1.70 Hz, 1H), 7.52 (d, *J* = 8.5, 2.5 Hz, 1H), 7.29 (app t, *J* = 9.9 Hz, 1H), 6.99 (d, *J* = 8.5 Hz, 1H), 4.36–4.27 (m, 4H), 3.79 (s, 2H), 2.61–2.28 (m, 9H), 1.55–1.38 (m, 1H), 1.35–1.16 (m, 1H), 0.91 (br s, 3H), 0.84 (t, *J* = 7.97 Hz, 3H). ^13^C NMR (126 MHz, DMSO-*d*_6_) δ 165.49, 164.96, 162.30, 152.30 (d, *J* = 247.7 Hz), 148.75, 146.93, 143.43, 137.97, 135.89 (d, *J* = 2.0 Hz), 131.75, 129.22, 128.99, 128.45, 127.93, 126.66, 125.82 (d, *J* = 13.6 Hz), 122.29, 121.73, 119.28, 119.19 (d, *J* = 7.5 Hz), 117.36, 117.16, 116.05 (d, *J* = 21.1 Hz), 64.88, 64.50, 60.14, 54.07, 48.14, 26.26, 14.13, 11.56. HRMS (ESI^+^): calcd for C_34_H_37_FN_5_O_4_ (M + H)^+^, 598.2824; found 598.2808.

##### 2-((4-(*tert*-Butyl)piperazin-1-yl)methyl)-*N*-(5-(2,3-dihydrobenzo[*b*][1,4]dioxine-6-carboxamido)-2-fluorophenyl)quinoline-6-carboxamide

A solution of *N*-(5-(2,3-dihydrobenzo[*b*][1,4]dioxine-6-carboxamido)-2-fluorophenyl)-2-formylquinoline-6-carboxamide (100 mg, 0.212 mmol) and 1-(*tert*-butyl)piperazine (91 mg, 0.636 mmol) in anhydrous DCM (2.0 mL) was allowed to stir at 20 °C for 12 h, after which sodium triacetoxyborohydride (135 mg, 0.636 mmol) was added in one portion, and the resulting mixture was allowed to stir at 20 °C for 2 h. The reaction was quenched with a NaHCO_3_ saturated aqueous solution (5 mL) and extracted with a mixture of DCM/MeOH 9/1 (3 × 5 mL). The crude product was purified by column chromatography on silica gel using a gradient of 0–15% MeOH in DCM and then washed with water and triturated with diethyl ether to afford the title compound as an amorphous beige solid (63 mg, 50%). ^1^H NMR (500 MHz, DMSO-*d*_6_) δ 10.50 (s, 1H), 10.29 (s, 1H), 8.69 (d, *J* = 1.5 Hz, 1H), 8.50 (d, *J* = 8.4 Hz, 1H), 8.27 (dd, *J* = 8.4, 1.5 Hz, 1H), 8.13 (dd, *J* = 7.3, 2.4 Hz, 1H), 8.09 (d, *J* = 8.5 Hz, 1H), 7.74 (d, *J* = 8.4 Hz, 1H), 7.61–7.66 (m, 1H), 7.57 (d, *J* = 2.2 Hz, 1H), 7.55 (d, *J* = 8.5, 2.2 Hz, 1H), 7.28 (app t, *J* = 9.8 Hz, 1H), 6.98 (d, *J* = 8.5 Hz, 1H), 4.35–4.27 (m, 4H), 3.85 (s, 2H), 3.09–2.54 (m, 8H), 1.20 (br s, 9H). ^13^C NMR (126 MHz, DMSO-*d*_6_) δ 165.48, 164.96, 161.49, 152.55 (d, *J* = 247.7 Hz) 148.73, 146.92, 143.41, 138.04, 135.94 (d, *J* = 2.3 Hz), 131.78, 129.21, 129.00, 128.50, 127.91, 126.66, 125.74 (d, *J* = 12.4 Hz), 122.33, 121.73, 119.63, 119.42 (d, *J* = 7.1 Hz), 117.35, 117.23, 116.02 (d, *J* = 20.7 Hz), 64.87, 64.43, 56.26, 53.11, 45.83, 25.59. HRMS (ESI^+^): calcd for C_34_H_37_FN_5_O_4_ (M + H)^+^, 598.2824; found 598.2809.

##### (*R*)-*N*-(5-(2,3-Dihydrobenzo[*b*][1,4]dioxine-6-carboxamido)-2-fluorophenyl)-2-((4-ethyl-2-methylpiperazin-1-yl)methyl)quinoline-6-carboxamide

A solution of *N*-(5-(2,3-dihydrobenzo[*b*][1,4]dioxine-6-carboxamido)-2-fluorophenyl)-2-formylquinoline-6-carboxamide (0.200 g, 0.424 mmol) and (*R*)-*tert*-butyl 3-methylpiperazine-1-carboxylate (255 mg, 1.27 mmol) in anhydrous DCM (2.00 mL) was allowed to stir at 20 °C for 12 h, after which sodium triacetoxyborohydride (0.270 g, 1.27 mmol) was added in one portion, and the resulting mixture was allowed to stir at 20 °C for 2 h. The reaction was quenched with NaHCO_3_ aqueous saturated solution (5 mL) and extracted with a mixture of DCM/MeOH 9/1 (3 × 5 mL). The organic layers were dried over Na_2_SO_4_ and concentrated under reduced pressure to afford the crude product (*R*)-*tert*-butyl 4-((6-((5-(2,3-dihydrobenzo[*b*][1,4]dioxine-6-carboxamido)-2-fluorophenyl)carbamoyl)quinolin-2-yl)methyl)-3-methylpiperazine-1-carboxylate (278 mg), which was dissolved in anhydrous DCM (2.5 mL) and treated with TFA (0.162 mL, 2.12 mmol). The resulting mixture was allowed to stir for 18 h, after which it was concentrated under vacuum to afford the crude product, which was taken onto the next step without purification (185 mg).

To a solution of (*R*)-*N-*(5-(2,3-dihydrobenzo[*b*][1,4]dioxine-6-carboxamido)-2-fluorophenyl)-2-((2-methylpiperazin-1-yl)methyl)quinoline-6-carboxamide (0.185 g, 0.333 mmol) in anhydrous MeOH (3 mL) at 0 °C, sodium cyanoborohydride (23.02 mg, 0.366 mmol) was added in one portion, followed by the dropwise addition of acetaldehyde (0.013 mL, 0.233 mmol), and the resulting solution was allowed to warm to 20 °C and stirred under argon for 18 h. The solvent was removed under reduced pressure, and the crude was redissolved in DCM and washed with NaOH aqueous solution (1 M). It was purified by column chromatography on silica gel using a gradient of 0–20% MeOH in DCM and then washed with water and triturated with diethyl ether to afford the title compound as an amorphous white solid (27 mg, 14%). ^1^H NMR (500 MHz, DMSO-*d*_6_) δ 10.38 (s, 1H), 10.18 (s, 1H), 8.64 (d, *J* = 2.2 Hz, 1H), 8.48 (d, *J* = 8.8 Hz, 1H), 8.24 (dd, *J* = 8.8, 2.2 Hz, 1H), 8.14 (dd, *J* = 6.6, 2.2 Hz, 1H), 8.07 (d, *J* = 8.8 Hz, 1H), 7.74 (d, *J* = 8.8 Hz, 1H), 7.68–7.63 (m, 1H), 7.54 (d, *J* = 2.2 Hz, 1H), 7.52 (d, *J* = 8.8, 2.2 Hz, 1H), 7.29 (app t, *J* = 10.3 Hz, 1H), 6.99 (d, *J* = 8.8 Hz, 1H), 4.35–4.28 (m, 4H), 4.23 (d, *J* = 14.2 Hz, 1H), 3.53 (d, *J* = 14.1 Hz, 1H), 2.74–2.57 (m, 3H), 2.34–2.21 (m, 4H), 2.12–1.99 (m, 1H), 1.95–1.82 (m, 1H), 1.09 (d, *J* = 6.5 Hz, 3H), 0.98 (t, *J* = 7.0 Hz, 3H). ^13^C NMR (126 MHz, DMSO-*d*_6_) δ 165.49, 164.95, 163.37, 153.31, 152.41 (d, *J* = 241.12 Hz), 146.91, 143.41, 137.80, 135.91 (d, *J* = 2.70 Hz), 131.65, 129.15, 128.99, 128.44, 127.92, 126.58, 125.81 (d, *J* = 13.49 Hz), 122.32, 121.75, 119.36, 119.21 (d, *J* = 6.28 Hz), 117.34, 117.19, 116.02 (d, *J* = 20.16 Hz), 64.87, 64.49, 60.70, 55.68, 53.09, 52.34, 52.02, 29.47, 17.61, 12.44. HRMS (ESI^+^): calcd for C_33_H_35_FN_5_O_4_ (M + H)^+^, 584.2668; found 584.2665.

##### *N*-(5-(2,3-Dihydrobenzo[*b*][1,4]dioxine-6-carboxamido)-2-fluorophenyl)-2-((4-ethyl-1,4-diazepan-1-yl)methyl)quinoline-6-carboxamide

A solution of *N*-(5-(2,3-dihydrobenzo[*b*][1,4]dioxine-6-carboxamido)-2-fluorophenyl)-2-formylquinoline-6-carboxamide (250 mg, 0.530 mmol) and 1-ethyl-1,4-diazepane (204 mg, 1.59 mmol) in anhydrous DCM (5 mL) was allowed to stir at 20 °C for 18 h, after which sodium triacetoxyborohydride (337 mg, 1.59 mmol) was added in one portion, and the resulting mixture was allowed to stir at 20 °C for 3 h. The reaction was quenched with a NaHCO_3_ saturated aqueous solution (5 mL) and extracted with a mixture of DCM/MeOH 9/1 (3 × 5 mL). Purification by column chromatography on silica gel using a gradient of 0–10% MeOH in DCM, followed by elution through an Isolute SCX-II cartridge using MeOH/NH_3_, and trituration with MeOH, afforded the title compound as an amorphous pale yellow solid (40 mg, 13%). ^1^H NMR (500 MHz, DMSO-*d*_6_) δ 10.43 (s, 1H), 10.23 (s, 1H), 8.68 (d, *J* = 1.7 Hz, 1H), 8.51 (d, *J* = 8.5 Hz, 1H), 8.27 (dd, *J* = 8.8, 1.9 Hz, 1H), 8.14 (dd, *J* = 7.0, 2.4 Hz, 1H), 8.08 (d, *J* = 8.8 Hz, 1H), 7.80 (d, *J* = 8.5 Hz, 1H), 7.66 (ddd, *J* = 8.7, 4.1, 2.7 Hz, 1H), 7.58–7.50 (m, 2H), 7.29 (t, *J* = 9.5 Hz, 1H), 6.99 (d, *J* = 8.4 Hz, 1H), 4.31 (q, *J* = 5.0 Hz, 5H), 4.00 (s, 2H), 3.07 (s, 4H), 2.94 (s, 2H), 2.82–2.73 (m, 2H), 1.97 (s, 2H), 1.20 (s, 4H). ^13^C NMR (126 MHz, DMSO-*d*_6_) δ 165.47, 164.96, 162.47, 152.56 (d, *J* = 242.7 Hz), 148.71, 146.92, 143.42, 138.11, 134.86 (d, *J* = 2.5 Hz), 131.79, 129.22, 129.04, 128.53, 127.92, 126.70, 125.81 (d, *J* = 13.9 Hz), 122.28, 121.75, 119.41, 119.28 (d, *J* = 8.8 Hz), 117.34, 117.20, 116.01 (d, *J* = 20.2 Hz), 64.88, 64.49, 64.17, 54.26, 53.74, 52.32, 51.78, 49.58, 26.29, 10.79. HRMS (ESI^+^): calcd for C_33_H_35_FN_5_O_4_ (M + H)^+^, 584.2673; found 584.2697.

##### 2-((4-Cyclopropylpiperazin-1-yl)methyl)-*N*-(5-(2,3-dihydrobenzo[*b*][1,4]dioxine-6-carboxamido)-2-fluorophenyl)quinoline-6-carboxamide

A solution of *N*-(5-(2,3-dihydrobenzo[*b*][1,4]dioxine-6-carboxamido)-2-fluorophenyl)-2-formylquinoline-6-carboxamide (100 mg, 0.212 mmol) and 1-cyclopropylpiperazine (0.077 mL, 0.636 mmol) in anhydrous DCM (2 mL) was allowed to stir at 20 °C for 6 h, after which sodium triacetoxyborohydride (135 mg, 0.636 mmol) was added in one portion, and the resulting mixture was allowed to stir at 20 °C for 2 h. The reaction was quenched with NaHCO_3_ aqueous saturated solution (5 mL) and extracted with a DCM/MeOH 9/1 mixture (3 × 5 mL). Purification by column chromatography on silica gel using a gradient of 0–10% MeOH in DCM, then triturated with diethyl ether, afforded the desired product as a pale yellow amorphous solid (47 mg, 38.1%). ^1^H NMR (500 MHz, DMSO-*d*_6_) δ 10.38 (s, 1H), 10.18 (s, 1H), 8.65 (d, *J* = 1.7 Hz, 1H), 8.49 (d, *J* = 8.5 Hz, 1H), 8.25 (dd, *J* = 9.1, 2.6 Hz, 1H), 8.14 (dd, *J* = 6.8, 2.6 Hz, 1H), 8.08 (d, *J* = 9.15 Hz, 1H), 7.73 (d, *J* = 8.5 Hz, 1H), 7.68–7.63 (m, 1H), 7.54 (d, *J* = 1.7 Hz, 1H), 7.52 (dd, *J* = 7.7, 1.7 Hz, 1H), 7.30 (app t, *J* = 9.9 Hz, 1H), 6.99 (d, *J* = 8.5 Hz, 1H), 4.34–4.28 (m, 4H), 3.78 (s, 2H), 2.57 (br s, 4H), 2.44 (br s, 4H), 1.61 (app heptet, *J* = 3.3 Hz, 1H), 0.42–0.37 (m, 2H), 0.29–0.25 (m, 2H). ^13^C NMR (126 MHz, DMSO-*d*_6_) δ 165.48, 164.95, 162.19, 152.33 (d, *J* = 243.7 Hz), 148.73, 146.92, 143.42, 138.00, 135.91 (d, *J* = 2.4 Hz), 131.74, 129.22, 128.98, 128.45, 127.93, 126.66, 125.81 (d, *J* = 12.2 Hz), 122.28, 121.72, 119.27, 119.18 (d, *J* = 7.4 Hz), 117.36, 117.16, 116.05 (d, *J* = 21.2 Hz), 64.88, 64.49, 53.47, 53.30, 53.26, 38.48, 6.10. HRMS (ESI^+^): calcd for C_33_H_33_FN_5_O_4_ (M + H)^+^, 582.2511; found 582.2487.

##### *N*-(2-Bromo-5-nitrophenyl)-2-methylquinoline-6-carboxamide

To a suspension of 2-methylquinoline-6-carboxylic acid (1.50 g, 8.01 mmol) in anhydrous DCM (20 mL), DMF (1.11 μL, 0.014 mmol) followed by oxalyl chloride (0.588 mL, 6.95 mmol) was added dropwise, and the resulting green solution was allowed to stir at 20 °C for 3 h, after which it was concentrated under vacuum to afford a dry pale green solid. The solid was dissolved in pyridine (20 mL), and 2-bromo-5-nitroaniline (1.26 g, 5.79 mmol) was added in one portion. The resulting dark yellow suspension was allowed to stir for 2 h, after which it was poured into water. The resulting yellow precipitate was filtered and washed several times with water, diethyl ether, and finally with a minimum amount of DCM to afford the crude product as an amorphous yellow solid, which was used without further purification (2.47 g). ^1^H NMR (500 MHz, DMSO*-d*_6_) δ 10.56 (s, 1H), 8.65 (d, *J* = 1.9 Hz, 1H), 8.52 (app t, *J* = 1.8 Hz, 1H), 8.44 (d, *J* = 8.3 Hz, 1H), 8.26 (dd, *J* = 8.9, 1.88 Hz, 1H), 8.09–8.05 (m, 3H), 7.55 (d, *J* = 8.3 Hz, 1H), 2.71 (s, 3H). HRMS (ESI^+^): calcd for C_17_H_13_^79^BrN_3_O_3_ (M + H)^+^, 386.0135; found 386.0129.

##### *N*-(5-Amino-2-bromophenyl)-2-methylquinoline-6-carboxamide

To a solution of *N-*(2-bromo-5-nitrophenyl)-2-methylquinoline-6-carboxamide (2.00 g, 5.18 mmol) in water (7 mL) and EtOH (21 mL), ammonium chloride (1.939 g, 36.3 mmol) and iron powder (2.03 g, 36.3 mmol) were added, and the resulting suspension was allowed to stir at 90 °C for 1 h. The reaction mixture was allowed to cool to room temperature, diluted with MeOH and DCM, and filtered through a pad of celite. The resulting filtrate was concentrated under vacuum to afford a light brown solid as a crude product, which was taken onto the next step without purification (1.80 g, 98%). ^1^H NMR (500 MHz, DMSO*-d*_6_) δ 9.94 (s, 1H), 8.59 (d, *J* = 1.8 Hz, 1H), 8.40 (d, *J* = 8.8 Hz, 1H), 8.22 (dd, *J* = 8.8, 1.8 Hz, 1H), 8.02 (d, *J* = 8.8 Hz, 1H), 7.52 (d, *J* = 8.8 Hz, 1H), 7.28 (d, *J* = 8.8 Hz, 1H), 6.86 (d, *J* = 1.8 Hz, 1H), 6.45 (dd, *J* = 8.8, 1.8 Hz, 1H), 5.40 (br s, 2H), 2.70 (s, 3H). ^13^C NMR (126 MHz, DMSO*-d*_6_) δ 165.22, 161.30, 149.30, 148.96, 137.63, 136.84, 132.78, 131.74, 128.87, 128.64, 128.23, 125.84, 123.47, 114.07, 105.01, 49.06, 25.51. HRMS (ESI^+^): calcd for C_17_H_15_^79^BrN_3_O (M + H)^+^, 358.0393; found 358.0386.

##### *N*-(2-Bromo-5-(2,3-dihydrobenzo[*b*][1,4]dioxine-6-carboxamido)phenyl)-2-methylquinoline-6-carboxamide

To a suspension of 2,3-dihydrobenzo[*b*][1,4]dioxine-6-carboxylic acid (1.00 g, 5.56 mmol) in anhydrous DCM (20 mL), DMF (0.972 μL, 0.013 mmol) and oxalyl chloride (0.513 mL, 6.06 mmol) were added dropwise, and the resulting green solution was allowed to stir at 20 °C for 3 h, after which it was concentrated under vacuum to afford a dry pale green solid. The solid was dissolved in pyridine (20 mL), and *N-*(5-amino-2-bromophenyl)-2-methylquinoline-6-carboxamide (1.80 g, 5.05 mmol) was added in one portion. The resulting dark yellow suspension was allowed to stir for 72 h, after which it was poured into water. The resulting yellow precipitate was filtered and washed several times with water, diethyl ether, and finally with a minimum amount of DCM to afford the crude product as a pale yellow amorphous solid, which was used without further purification (2.11 g, 81%). ^1^H NMR (500 MHz, DMSO*-d*_6_) δ 10.29 (s, 1H), 10.27 (s, 1H), 8.63 (s, 1H), 8.42 (d, *J* = 9.1 Hz, 1H), 8.25 (d, *J* = 7.3 Hz, 1H), 8.10 (s, 1H), 8.05 (d, *J* = 8.2 Hz, 1H), 7.69 (br s, 2H), 7.58–7.49 (m, 3H), 6.99 (d, *J* = 9.1 Hz, 1H), 4.37–4.27 (m, 4H), 2.71 (s, 3H). ^13^C NMR (126 MHz, DMSO*-d*_6_) δ 165.49, 165.13, 161.43, 149.03, 147.04, 143.43, 139.67, 137.68, 136.89, 132.90, 131.43, 128.95, 128.81, 128.25, 127.76, 125.86, 123.54, 121.79, 120.72, 120.18, 117.39, 117.21, 114.50, 64.88, 64.49, 25.51. HRMS (ESI^+^): calcd for C_26_H_21_^79^BrN_3_NaO_4_ (M + Na)^+^, 540.0529; found 520.0542.

##### *N*-(2-Bromo-5-(2,3-dihydrobenzo[*b*][1,4]dioxine-6-carboxamido)phenyl)-2-formylquinoline-6-carboxamide

A solution of *N-*(2-bromo-5-(2,3-dihydrobenzo[*b*][1,4]dioxine-6-carboxamido)phenyl)-2-methylquinoline-6-carboxamide (1.00 g, 1.93 mmol) and selenium dioxide (0.235 g, 2.12 mmol) in anhydrous DMF (4 mL) and anhydrous 1,4-dioxane (12 mL) was heated at 150 °C for 1 h. The reaction mixture was allowed to cool to room temperature, and it was diluted with DCM and filtered through a pad of celite. The filtrate was concentrated under vacuum to afford the crude product as a yellow amorphous solid, which was used without purification (1.00 g, 97%). ^1^H NMR (500 MHz, DMSO*-d*_6_) δ 10.47 (s, 1H), 10.28 (s, 1H), 10.17 (d, *J* = 0.5 Hz, 1H), 8.81–8.77 (m, 2H), 8.42–8.36 (m, 2H), 8.13–8.11 (m, 1H), 8.09 (d, *J* = 8.4 Hz, 1H), 7.70 (d, *J* = 1.1 Hz, 2H), 7.55 (d, *J* = 2.2 Hz, 1H), 7.52 (dd, *J* = 8.6, 2.2 Hz, 1H), 7.00 (d, *J* = 8.6 Hz, 1H), 4.36–4.23 (m, 4H). HRMS (ESI^+^): calcd for C_26_H_19_^79^BrN_3_O_5_ (M + H)^+^ 532.0503; found 532.0513.

##### 2-(Azetidin-1-ylmethyl)-*N*-(2-bromo-5-(2,3-dihydrobenzo[*b*][1,4]dioxine-6-carboxamido)phenyl)quinoline-6-carboxamide

A solution of *N-*(2-bromo-5-(2,3-dihydrobenzo[*b*][1,4]dioxine-6-carboxamido)phenyl)-2-formylquinoline-6-carboxamide (0.500 g, 0.939 mmol) and azetidine (161 mg, 2.82 mmol) in anhydrous DCM (8 mL) was allowed to stir at 20 °C for 12 h, after which sodium triacetoxyborohydride (0.597 g, 2.82 mmol) was added in one portion, and the resulting mixture was allowed to stir at 20 °C for 1 h. The reaction was quenched with NaHCO_3_ aqueous saturated solution (10 mL) and extracted with a DCM/MeOH 9/1 mixture (3 × 10 mL). Purification by column chromatography on silica gel using a gradient of 0–20% MeOH in DCM, followed by washing with water, trituration with diethyl ether and elution through an Isolute SCX-II cartridge using MeOH/NH_3_, afforded the title compound as a pale beige amorphous solid (54 mg, 10%). ^1^H NMR (500 MHz, DMSO*-d*_6_) δ 10.33 (s, 1H), 10.27 (s, 1H), 8.67 (d, *J* = 1.5 Hz, 1H), 8.53 (d, *J* = 8.0 Hz, 1H), 8.29 (dd, *J* = 8.8, 1.5 Hz, 1H), 8.13–8.09 (m, 2H), 7.69 (d, *J* = 1.5 Hz, 1H), 7.65 (d, *J* = 8.8 Hz, 2H), 7.55 (d, *J* = 2.4 Hz, 1H), 7.52 (dd, *J* = 8.0, 1.5 Hz, 1H), 7.00 (d, *J* = 8.0 Hz, 1H), 4.35–4.28 (m, 4H) 4.19 (br s, 2H), 3.59 (br s, 4H), 2.18 (br s, 2H). ^13^C NMR (126 MHz, DMSO-*d*_6_) δ 165.31, 165.13, 157.8, 148.47, 147.05, 143.43, 140.01, 139.71, 138.62, 136.81, 132.90, 132.34, 129.29, 128.99, 128.82, 127.74, 126.77, 121.81, 120.80, 120.29, 117.38, 117.23, 114.57, 64.89, 64.48, 61.17, 55.23, 17.44. HRMS (ESI^+^): calcd for C_29_H_26_^81^BrN_4_O_4_ (M + H)^+^, 575.1116; found 575.1088.

##### *N*-(2-Bromo-5-(2,3-dihydrobenzo[*b*][1,4]dioxine-6-carboxamido)phenyl)-2-((4-ethylpiperazin-1-yl)methyl)quinoline-6-carboxamide

A solution of *N*-(2-bromo-5-(2,3-dihydrobenzo[*b*][1,4]dioxine-6-carboxamido)phenyl)-2-formylquinoline-6-carboxamide (0.500 g, 0.939 mmol) and 1-ethylpiperazine (322 mg, 2.82 mmol) in anhydrous DCM (8 mL) was allowed to stir at 20 °C for 12 h, after which sodium triacetoxyborohydride (0.597 g, 2.82 mmol) was added in one portion, and the resulting mixture was allowed to stir at 20 °C for 1 h. The reaction was quenched with NaHCO_3_ aqueous saturated solution (10 mL) and extracted with a DCM/MeOH 9/1 mixture (3 × 10 mL). Purification by column chromatography on silica gel using a gradient of 0–20% MeOH in DCM, followed by elution through an Isolute SCX-II cartridge using MeOH/NH_3_, afforded the title compound as a bright yellow amorphous solid (110 mg, 18%). ^1^H NMR (500 MHz, DMSO-*d*_6_) δ 10.31 (s, 1H), 10.27 (s, 1H), 8.66 (d, *J* = 2.18 Hz, 1H), 8.50 (d, *J* = 8.70 Hz, 1H), 8.27 (dd, *J* = 8.70, 2.18 Hz, 1H), 8.13–8.08 (m, 2H), 7.73 (d, *J* = 8.70 Hz, 1H), 7.70–7.68 (m, 2H), 7.55 (d, *J* = 2.18 Hz, 1H), 7.52 (dd, *J* = 8.70, 2.18 Hz, 1H), 7.00 (d, *J* = 8.70 Hz, 1H), 4.35–4.28 (m, 4H), 3.81 (br s, 2H), 2.51 (br s, 10 H), 1.02 (br t, *J* = 6.68 Hz, 3H). ^13^C NMR (126 MHz, DMSO-*d*_6_) δ 165.39, 165.12, 162.02, 148.74, 147.05, 143.44, 139.69, 138.01, 136.85, 132.90, 131.89, 129.30, 128.83, 128.32, 127.75, 126.71, 122.32, 121.79, 120.73, 120.20, 117.39, 117.21, 114.49, 64.88, 64.68, 64.49, 53.13, 52.61, 51.94, 12.18. HRMS (ESI^+^): calcd for C_32_H_33_^79^BrN_5_O_4_ (M + H)^+^, 630.1710; found 632.1694.

### Synthesis of the Clinical Candidate CCT361814

#### *N*-(5-(2,3-Dihydrobenzo[*b*][1,4]dioxine-6-carboxamido)-2-fluorophenyl)-2-((4-ethylpiperazin-1-yl)methyl)quinoline-6-carboxamide (**22**)

### Step 1. *N*-(2-Fluoro-5-nitrophenyl)-2-methylquinoline-6-carboxamide

Oxalyl chloride (3.25 mL, 38.4 mmol) was added dropwise to a solution of 2-methylquinoline-6-carboxylic acid (6.59 g, 35.2 mmol) and DMF (0.0062 mL, 0.080 mmol) in anhydrous DCM (80 mL). The reaction mixture was stirred at room temperature for 3 h and then concentrated under reduced pressure. The residue was dissolved in DCM (30 mL) and concentrated again under reduced pressure. The resulting dry residue was dissolved in pyridine (80 mL), and 2-fluoro-5-nitroaniline (5.00 g, 32.0 mmol) was added in one portion. The reaction mixture was stirred at room temperature for 18 h and then poured into water (100 mL). The green precipitate was filtered and washed with water (3 × 20 mL), diethyl ether (3 × 20 mL), and DCM (10 mL) to afford the title compound as a light green solid, which was carried onto the next step without further purification (10.4 g). ^1^H NMR (500 MHz, DMSO-*d*_6_) δ 10.70 (s, 1H), 8.72 (dd, *J* = 6.45, 2.93 Hz, 1H), 8.63 (d, *J* = 2.02 Hz, 1H), 8.43 (d, *J* = 8.46 Hz, 1H), 8.23 (dd, *J* = 8.48, 2.02 Hz, 1H), 8.21–8.16 (m, 1H), 8.05 (d, *J* = 8.86 Hz, 1H), 7.65 (app t, *J* = 9.25 Hz, 1H), 7.54 (d, *J* = 8.46 Hz, 1H), 2.71 (s, 3H). ^13^C NMR (126 MHz, DMSO-*d*_6_) δ 165.53, 161.22, 158.67 (d, *J* = 258.2 Hz), 148.65, 143.72, 137.32, 130.36, 128.88, 128.48, 128.00, 127.08 (d, *J* = 13.9 Hz), 125.33, 123.18, 122.14 (d, *J* = 9.6 Hz), 121.25 (d, *J* = 3.8 Hz), 117.19 (d, *J* = 22.8 Hz), 25.07. ^19^F NMR (470 MHz, DMSO-*d*_6_) δ −110.20. HRMS (ESI^+^): calcd for C_17_H_13_FN_3_O_3_ (M + H)^+^, 326.0935; found 326.0931.

### Step 2. *N*-(5-Amino-2-fluorophenyl)-2-methylquinoline-6-carboxamide

To a solution of *N-*(2-fluoro-5-nitrophenyl)-2-methylquinoline-6-carboxamide (10.4 g, 32.0 mmol) in ethanol (120 mL) and water (40 mL), ammonium chloride (12.0 g, 224 mmol) and iron powder (12.5 g, 224 mmol) were added in one portion, and the resulting suspension was allowed to stir at 90 °C for 1 h. The reaction mixture was allowed to cool to room temperature, diluted with MeOH (20 mL) and DCM (20 mL), and filtered through a pad of celite. The resulting filtrate was concentrated under vacuum to afford a light brown solid, which was redissolved in a mixture of DCM/MeOH (9:1, 150 mL) and washed with saturated aqueous NaHCO_3_ (150 mL). The organic phase was dried over Na_2_SO_4_, filtered, and concentrated under reduced pressure to afford a yellow solid as a crude product, which was taken directly onto the next step without further purification (9.46 g). ^1^H NMR (500 MHz, DMSO-*d*_6_) δ 10.05 (s, 1H), 8.57 (d, *J* = 1.67 Hz, 1H), 8.39 (d, *J* = 8.74 Hz, 1H), 8.19 (dd, *J* = 8.74, 1.67 Hz, 1H), 8.01 (d, *J* = 8.74 Hz, 1H), 7.52 (d, *J* = 8.33 Hz, 1H), 6.94 (dd, *J* = 9.78, 8.28 Hz, 1H), 6.89 (dd, *J* = 6.58, 2.74 Hz, 1H), 6.46–6.39 (m, 1H), 5.05 (br s, 2H), 2.70 (s, 3H). ^13^C NMR (126 MHz, DMSO-*d*_6_) δ 164.93, 160.84, 148.49, 147.72 (d, *J* = 233.9 Hz), 145.08 (d, *J* = 1.9 Hz), 137.19, 131.19, 128.44, 128.33, 127.95, 125.50 (d, *J* = 13.1 Hz), 125.34, 122.99, 115.54 (d, *J* = 20.6 Hz), 111.52, 111.39 (d, *J* = 6.6 Hz), 25.05. ^19^F NMR (470 MHz, DMSO-*d*_6_) δ −138.12. HRMS (ESI^+^): calcd for C_17_H_15_FN_3_O (M + H)^+^, 296.1194; found 296.1191.

### Step 3. *N*-(5-(2,3-Dihydrobenzo[*b*][1,4]dioxine-6-carboxamido)-2-fluorophenyl)-2-methylquinoline-6-carboxamide

To a suspension of 2,3-dihydrobenzo[*b*][1,4]dioxine-6-carboxylic acid (12.7 g, 70.5 mmol) in anhydrous DCM (100 mL) under an inert atmosphere was dropwise added a catalytic amount of anhydrous DMF (6.16 μL, 0.080 mmol) and oxalyl chloride (6.51 mL, 77.0 mmol) and the resulting green solution was allowed to stir at room temperature for 3 h. After which time, the reaction mixture was concentrated under vacuum to afford a dry pale green solid. The solid was dissolved in pyridine (100 mL), and *N-*(5-amino-2-fluorophenyl)-2-methylquinoline-6-carboxamide (9.46 g, 32.0 mmol) was added in one portion. The resulting dark yellow suspension was allowed to stir for 2 h and was then poured onto water (100 mL). The yellow precipitate was filtered and washed with water (3 × 20 mL), diethyl ether (3 × 20 mL), and DCM (10 mL) to afford the crude product as a pale yellow solid, which was taken directly onto the next step without further purification (12.5 g). ^1^H NMR (500 MHz, DMSO-*d*_6_): δ 10.37 (s, 1H), 10.18 (s, 1H), 8.62 (d, *J* = 1.65 Hz, 1H), 8.41 (d, *J* = 8.77 Hz, 1H), 8.23 (dd, *J* = 8.77, 2.19 Hz, 1H), 8.13 (dd, *J* = 7.01, 2.63 Hz, 1H), 8.03 (d, *J* = 8.51 Hz, 1H), 7.68–7.63 (m, 1H), 7.55–7.53 (m, 2H), 7.52 (dd, *J* = 8.51, 2.09 Hz, 1H), 7.29 (dd, *J* = 9.98, 8.69 Hz, 1H), 6.99 (d, *J* = 8.51 Hz, 1H), 4.34–4.28 (m, 4H), 2.71 (s, 3H). ^13^C NMR (126 MHz, DMSO-*d*_6_) δ 165.09, 164.48, 160.95, 151.83 (d, *J* = 243.4 Hz), 148.57, 146.45, 142.95, 137.22, 135.42 (d, *J* = 2.0 Hz), 130.87, 128.50, 128.41, 127.94, 127.47, 125.39 (d, *J* = 9.5 Hz), 125.35, 123.05, 121.25, 118.81, 118.75 (d, *J* = 13.0 Hz), 116.89, 116.69, 115.56 (d, *J* = 21.2 Hz), 64.41, 64.03, 25.06. ^19^F NMR (470 MHz, DMSO-*d*_6_) δ −126.65. HRMS (ESI+): calcd for C_26_H_21_FN_3_O_4_ (M + H)^+^, 458.1511; found 458.1499.

### Step 4. *N*-(5-(2,3-Dihydrobenzo[*b*][1,4]dioxine-6-carboxamido)-2-fluorophenyl)-2-formylquinoline-6-carboxamide

A solution of *N-*(5-(2,3-dihydrobenzo[*b*][1,4]dioxine-6-carboxamido)-2-fluorophenyl)-2-methylquinoline-6-carboxamide (5.00 g, 10.9 mmol) and selenium dioxide (1.33 g, 12.0 mmol) in anhydrous DMF (40 mL) and 1,4-dioxane (120 mL) was heated at reflux for 1 h under an inert atmosphere. After which time, the reaction mixture was allowed to cool to room temperature, diluted with DCM (20 mL), and filtered through a pad of celite. The filtrate was concentrated under vacuum (using a heptane/EtOAc azeotrope to remove DMF) to afford the crude product as a yellow solid, which was carried onto the next step without further purification (5.15 g). ^1^H NMR (500 MHz, DMSO-*d*_6_) δ 10.54 (s, 1H), 10.19 (s, 1H), 10.17 (s, 1H), 8.81–8.77 (m, 2H), 8.39 (dd, *J* = 8.73, 1.95 Hz, 1H), 8.36 (d, *J* = 8.73 Hz, 1H), 8.17 (dd, *J* = 6.93, 2.57 Hz, 1H), 8.09 (d, *J* = 9.26 Hz, 1H), 7.69–7.64 (m, 1H), 7.55 (d, *J* = 1.99 Hz, 1H), 7.52 (dd, *J* = 8.30, 1.99 Hz, 1H), 7.31 (app t, *J* = 9.97 Hz, 1H), 6.99 (d, *J* = 8.30 Hz, 1H), 4.36–4.27 (m, 4H). HRMS (ESI+): calcd for C_26_H_19_FN_3_O_5_ (M + H)^+^, 472.1303; found 472.1286.

### Step 5. *N*-(5-(2,3-Dihydrobenzo[*b*][1,4]dioxine-6-carboxamido)-2-fluorophenyl)-2-((4-ethylpiperazin-1-yl)methyl)quinoline-6-carboxamide (**22**)

A solution of *N*-(5-(2,3-dihydrobenzo[*b*][1,4]dioxine-6-carboxamido)-2-fluorophenyl)-2-formylquinoline-6-carboxamide (1.19 g, 2.52 mmol) and 1-ethylpiperazine (7.57 mmol, 0.87 g, 0.96 mL) in anhydrous DCM (20 mL) was allowed to stir at 20 °C for 6 h. After which time, sodium triacetoxyborohydride (1.61 g, 7.57 mmol) was added in one portion and the resulting mixture was allowed to stir at 20 °C for 2 h. The reaction was quenched with saturated aqueous NaHCO_3_ (20 mL) and extracted with a mixture of DCM/MeOH (9:1, 3 × 20 mL). Purification by column chromatography on silica gel in gradient with DCM/MeOH (0–10%) afforded a yellow solid, which was redissolved in DCM/MeOH (9:1, 100 mL) and washed with water (100 mL). The organic phase was dried over Na_2_SO_4_, filtered, and concentrated under reduced pressure. The final trituration of the resulting residue with diethyl ether (20 mL) afforded the title compound as a white solid (0.95 g, 66%). ^1^H NMR (500 MHz, DMSO-*d*_6_) δ 10.39 (s, 1H), 10.18 (s, 1H), 8.65 (d, *J* = 1.9 Hz, 1H), 8.49 (d, *J* = 8.4 Hz, 1H), 8.25 (dd, *J* = 8.8, 2.0 Hz, 1H), 8.14 (dd, *J* = 7.1, 2.6 Hz, 1H), 8.08 (d, *J* = 8.8 Hz, 1H), 7.73 (d, *J* = 8.5 Hz, 1H), 7.67–7.64 (m, 1H), 7.55 (d, *J* = 1.8 Hz, 1H), 7.53 (dd, *J* = 8.5, 1.8 Hz, 1H),7.29 (app t, *J* = 9.2 Hz, 1H), 6.99 (d, *J* = 8.4 Hz, 1H), 4.39–4.18 (m, 4H), 3.79 (s, 2H), 2.48–2.41 (m, 8H), 2.32 (q, *J* = 7.2 Hz, 2H), 0.98 (t, *J* = 7.2 Hz, 3H). ^13^C NMR (126 MHz, DMSO) δ 165.02, 164.50, 161.69, 151.85 (d, *J* = 242.7 Hz), 148.28, 146.46, 142.96, 137.53, 135.44 (d, *J* = 2.4 Hz), 131.29, 128.76, 128.53, 128.00, 127.47, 126.20, 125.35 (d, J = 12.9 Hz), 121.81, 121.27, 118.83, 118.72 (d, *J* = 7.3 Hz), 116.89, 116.71, 115.57 (d, *J* = 21.0 Hz), 64.42, 64.38, 64.04, 52.93, 52.34, 51.60, 11.91. ^19^F NMR (470 MHz, DMSO-*d*_6_) δ −126.63. HRMS (ESI+): calcd for C_32_H_33_FN_5_O_4_ (M + H)^+^, 570.2511; found 570.2532.

## Supporting Information Available

In vitro compound optimization data, in vivo prediction from in vitro data, pharmacokinetic and in vivo efficacy data, ER-stress in vivo biomarker discovery, full chemistry experimental, NMR spectra of key compounds, physicochemical property experimental, full biology experimental, and pharmacokinetics experimental (PDF)
SMILES molecular formula strings (CSV)

## Abbreviations Used

MDR: multidrug resistance
MDR1: multidrug resistance protein 1
PGP: p-glycoprotein
CRBN: cereblon
TGI: tumor growth inhibition
SD: Sprague-Dawley rats
HSF1: heat shock factor protein 1
SAR: structure–activity relationship
HSP72: heat shock 70 kDa protein 1
HTS: high-throughput screen
nM: nanomolar
μM: micromolar
IC_50_: half-minimal (50%) inhibitory concentration
GI_50_: concentration for 50% of maximal inhibition of cell proliferation
SEM: standard error of the mean
tPSA: topological polar surface area
MLMs: mouse liver microsomes
KS: kinetic solubility
HBF: hepatic blood flow
PK: pharmacokinetics
AUC: area under the curve
AUC_u_: unbound area under the curve
C_av_^0–24h^: average concentration over a 24 hour period
po: per os, oral dose
qd: quaque die, once-a-day dose
CL_tb_: total blood clearance
CL_u_: unbound clearance
CL_int_: intrinsic clearance
V_ss_: volume of distribution at steady state
V_du_: unbound volume of distribution
%F: percentage oral bioavailability
*f*_up_: fraction unbound in plasma
f_ub_: fraction unbound in blood
f_ua_: fraction unbound in the assay
B:P: blood-to-plasma ratio
CYP450: cytochrome P450
DMF: dimethylformamide
DCM: dichloromethane
ND: not determined
SF: significant figure
HLMs: human liver microsomes
RLMs: rat liver microsomes
WT: wild-type
KO: knockout
MMP: matched molecular pair
n: number of repeats
N: number of distinct samples
MHeps: mouse hepatocytes
