# Supplementary material for: HSF1 Pathway Inhibitor Clinical Candidate (CCT361814/NXP800) Developed from a Phenotypic Screen as a Potential Treatment for Refractory Ovarian Cancer and Other Malignancies
Source: J Med Chem. 2023 Apr 5;66(8):5907–36. doi: 10.1021/acs.jmedchem.3c00156 (PMC10150365; doi:10.1021/acs.jmedchem.3c00156)
Supplement: Supplementary file 1 — jm3c00156_si_001.pdf [file jm3c00156_si_001.pdf]

Supporting Information

An HSF1-Pathway Inhibitor Clinical Candidate (CCT361814/NXP800) Developed from a Phenotypic Screen as a Potential Treatment for Refractory Ovarian Cancer and other Malignancies

A. Elisa Pasqua,[1] Swee Y. Sharp,[1] Nicola E. A. Chessum,[1] Angela Hayes,[1] Loredana Pellegrino,[1] Michael J. Tucker,[1] Asadh Miah,[1] Birgit Wilding,[1] Lindsay E. Evans,[1] Carl S. Rye,[1] N. Yi Mok,[1] Manjuan Liu,[1] Alan T. Henley,[1] Sharon Gowan,[1] Emmanuel De Billy,[1] Robert te Poele,[1] Marissa Powers,[1] Suzanne A. Eccles,[1] Paul A. Clarke,[1] Florence I. Raynaud,[1] Paul Workman,[1]* Keith Jones,[1]* Matthew D. Cheeseman,[1]**

[1]Centre for Cancer Drug Discovery and Division of Cancer Therapeutics at The Institute of Cancer Research, London, SW7 3RP, United Kingdom
*Email: for P.A.C. paul.clarke@icr.ac.uk, for P.W. paul.workman@icr.ac.uk, for K.J. keith.jones@icr.ac.uk or for M.D.C. matthew.cheeseman@icr.ac.uk, phone (+44) 208 722 4168

Table of Contents

In vitro Compound Optimization Data S2 to S3

In vivo Predictions from in vitro Data S4 to S7

Pharmacokinetic and in vivo Efficacy Data S8 to S11

Chemistry Experimental Procedures S12 to S30

NMR Spectra of Key Compounds S31 to S43

UV-Traces of in vivo Compounds S44

Physicochemical Properties Experimental S45-S46

Biology Experimental Procedures S47

Pharmacokinetics Experimental Procedures S48 to S50

References S51

In vitro Compound Optimization Data

Ovarian Cancer Cell line profiling

Table S1. Human ovarian cancer cell line panel for the standard of care carboplatin, CCT251236 **1** and CCT361814 **22**

Human Cancer Cell-Line[b]	pGI_{50}±SEM (n) Carboplatin[b]	pGI_{50}±SEM (n) CCT251236 1	pGI_{50}±SEM (n) CCT361814 22[b]
SK-OV-3	<4.7 (1)	8.65±0.10 (25)	8.07±0.03 (45)
IGROV1	ND	7.89±0.11 (5)	7.64±0.06 (5)
OVCAR3	ND	7.46±0.07 (2)	7.17±0.02 (2)
OVCAR5	<5.0 (1)	7.31±0.14 (4)	6.86±0.02 (2)
OVCAR8	ND	7.26±0.01 (2)	6.77±0.10 (2)
CaOV3	ND	7.57±0.03 (3)	7.23±0.05 (3)
PA1	5.8 (1)	8.28±0.05 (3)	7.89±0.01 (3)
CH1	ND	8.50±0.09 (12)	7.80±0.08 (11)
RMG-1	<4.7 (1)	7.34±0.03 (2)	7.33±0.12 (4)

ND=not determined. pGI_{50}=-logGI_{50} (M). pGI_{50}±SEM=geometric mean±standard error of the mean. The numbers of repeats are in parentheses. [a]Carboplatin was purchased from Sigma-Aldrich (http://www.sigmaaldrich.com/catalog/product/sigma/c2538?lang=en&region=GB, February 2017). [b]Growth inhibition was measured after 96 hours of treatment and compared to the vehicle control in the CellTiter-Blue Assay.

Physicochemical properties and compound optimization

Table S2. Structure-Property Relationships of the Solubilizing Group in the Chloro-Series

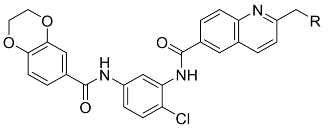

R	MDR -fold[a]	$LogD_{7.4}$[b]	KS (μM)[c]	MLM/RLM/HLM/MHeps Cl_{int} (μL/min/mg protein or μL/min/10^6 cells)[d]	Pred. Cl_u (mL/min/kg) MLM/RLM/HLM/MHeps[f]	SK-OV-3 GI_{50} pGI_{50}±SEM (n)[g]
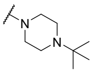	2.5	2.5	80	9/14/36/ 27[e]	35/25/36/ 913	21 nM 7.67±0.02 (4)
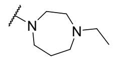	3.7	ND	ND	25/ND/ND/ ND	95/ND/ND/ ND	11 nM 7.95±0.03 (3)
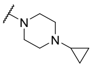	1.7	ND	ND	56/ND/ND/ ND	200/ND/ND/ ND	34 nM 7.47±0.03 (3)
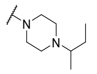	1.5	ND	ND	29/16/49/ ND	110/28/44/ ND	13 nM 7.88±0.01 (3)
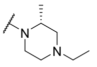	1.9	ND	ND	67/ND/ND/ ND	240/ND/ND/ ND	16 nM 7.79±0.02 (3)
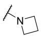	1.5	2.5	20	31/29/13/ ND	120/51/12/ ND	12 nM 7.93±0.07 (8)
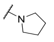	1.6	2.6	60	38/19/15/ ND	140/34/14/ ND	19 nM 7.73±0.06 (7)
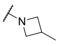	1.1	ND	30	31/21/18/ ND	120/37/16/ ND	8.7 nM 8.06±0.04 (3)
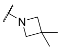	1.4	ND	10	51/ND/ND/ ND	180/ND/ND/ ND	13 nM 7.89±0.08 (3)

ND=not determined. All results are quoted to 2 SF unless otherwise stated [a]Results are quoted as the geometric mean of n=3 repeats; the fold resistance is given by the ratio of GI_{50}s in $CH1^{doxR}$ cells and $CH1^{WT}$ cells of the compounds in the CellTiter-Blue Assay. [b]Measured via an HPLC method, n=1. [c]Measured via an HPLC method from phosphate buffer at pH 7.4, all values quoted to 1 SF, the dynamic range of the assay is 1-100 μM, n=1. [d]Mouse/rat/human liver microsome (MLM/RLM/HLM/MHeps) assays were carried out at Cyprotex, n=1. In vitro Cl_{int} (μL/min/mg of protein or μL/min/10^6 cells) is calculated from the half-life using standard procedures. Assumes the fraction unbound in the assay is 1 for all microsome assays. [e]Assumes the fraction unbound in the assay is 0.2. [f]Calculated from the in vitro Cl_{int} using scaling factors and applying the well-stirred model, see supporting information for details. [g]The number of repeats, n, are described in parentheses, growth inhibition was measured after 96 hours of treatment and compared to the vehicle control, all results are quoted as the geometric mean±SEM, pGI_{50}=-logGI_{50} (M).

Table S3. MDR Pilot Screen and Halogen Substitution MMPs

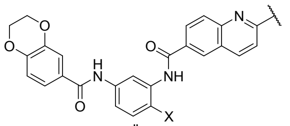

X	Solubilizing Group	SK-OV-3 pGI_{50}±SEM (n)[a]	$CH1^{WT}$ pGI_{50} / $CH1^{DOXR}$ pGI_{50}	MDR-fold resistance[b]
F	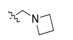	7.58±0.03 (3)	7.44 / 7.22	1.6 (p=0.05)
Cl		7.93±0.07 (8)	7.77 / 7.61	1.5 (p=0.03)
Br		7.95±0.24 (3)	7.92 / 7.86	1.2 (p=0.5)
CH_3	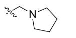	8.21±0.08 (3)	7.51 / 7.27	1.7 (p=0.2)
F		7.59±0.05 (3)	7.37 / 7.21	1.5 (p=0.2)
Cl		7.73±0.06 (7)	7.64 / 7.45	1.6 (p=0.07)
CH_3	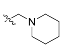	7.66±0.08 (3)	7.68 / 7.48	1.5 (p=0.05)
F		7.14±0.12 (3)	7.11 / 7.20	0.8 (p=0.1)
CH_3	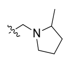	8.15±0.16 (3)	7.34 / 7.02	2.1 (p=0.2)
F		7.66±0.07 (3)	7.20 / 7.19	1.0 (p=0.9)
F	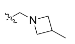	7.68±0.03 (3)	7.58 / 7.24	2.2 (p=0.1)
Cl		8.06±0.04 (3)	7.72 / 7.67	1.1 (p=0.3)
F	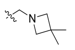	7.80±0.01 (3)	7.73 / 7.43	1.9 (p=0.1)
Cl		7.89±0.08 (3)	7.78 / 7.64	1.4 (p=0.07)
F	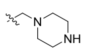	8.07±0.02 (3)	7.75 / 6.93	6.6 (p=0.004)
Cl		7.94±0.01 (3)	7.74 / 7.03	5.2 (p<0.001)
CH_3	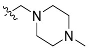	8.02±0.05 (3)	7.74 / 7.23	3.2 (p=0.2)
F		8.03±0.04 (8)	7.85 / 7.60	1.8 (p=0.001)
Cl		7.72±0.16 (3)	7.75 / 7.68	1.2 (p = 0.3)
CH_3	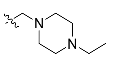	8.22±0.01 (3)	7.57 / 7.05	3.3 (p=0.1)
F		8.07±0.02 (45)	7.80 / 7.55	1.8 (p<0.001)
Cl		7.96±0.09 (10)	7.90 / 7.77	1.4 (p=0.02)
Br		7.91±0.03 (3)	7.80 / 7.79	1.0 (p=0.8)

X	Solubilizing Group	SK-OV-3 pGI_{50}±SEM (n)[a]	$CH1^{WT}$ pGI_{50} / $CH1^{DOXR}$ pGI_{50}	MDR-fold resistance[b]
CH_3	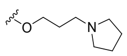	9.12±0.11 (3)	8.44 / 7.82	4.2 (p=0.003)
F		8.88±0.11 (3)	8.83 / 8.57	1.8 (p=0.01)
CH_3	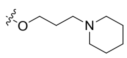	8.87±0.09 (8)	9.20 / 8.39	6.5 (p=0.01)
F		8.73±0.04 (3)	8.40 / 8.30	1.3 (p=0.5)
CH_3	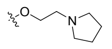	8.65±0.10 (25)	8.50 / 8.03	2.9 (p<0.001)
F		8.59±0.13 (3)	7.91 / 7.96	0.9 (p=0.5)
Cl		8.22±0.04 (3)	7.75 / 7.60	1.4 (p=0.5)
F	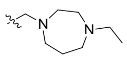	8.05±0.07 (3)	7.86 / 6.96	7.8 (p=0.007)
Cl		7.95±0.03 (3)	7.81 / 7.24	3.7 (p=0.004)
F	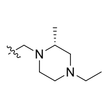	7.73±0.03 (4)	7.71 / 7.33	2.4 (p=0.004)
Cl		7.79±0.02 (3)	7.78 / 7.50	1.9 (p=0.04)
F	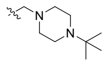	7.69±0.04 (3)	7.62 / 7.02	4.0 (p<0.001)
Cl		7.67±0.02 (4)	7.71 / 7.31	2.5 (p<0.001)
F	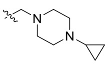	7.90±0.10 (3)	7.75 / 7.40	2.2 (p=0.1)
Cl		7.47±0.03 (3)	7.49 / 7.25	1.7 (p=0.07)
F	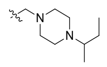	8.31±0.08 (4)	7.95 / 7.63	2.1 (p=0.003)
Cl		7.88±0.01 (3)	7.73 / 7.54	1.5 (p=0.02)
CH_3	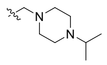	8.06±0.05 (3)	7.71 / 6.78	8.4 (p=0.003)
F		7.74±0.02 (3)	7.28 / 7.10	1.5 (p=0.4)
Cl		7.83±0.08 (8)	7.84 / 7.60	1.7 (p<0.001)

pGI_{50}=-log GI_{50} (M). [a]The number of repeats, n, are described in parentheses. Growth inhibition was measured after 96 hours of treatment and compared to the vehicle control using the CellTiter-Blue assay. All results are quoted as the geometric mean±SEM. b [a]Results are quoted as the geometric mean of at least n=3 biological repeats; the fold resistance is given by the ratio of GI_{50}s in $CH1^{doxR}$ cells and $CH1^{WT}$ cells of the compounds in the CellTiter-Blue Assay, the p-value was calculated by comparing the geometric mean of the relevant GI_{50} values rates using a two-tailed Student's t-test with Welch's correction.

In vivo predictions from in vitro Data

All in vitro metabolism assays were carried out at Cyprotex (http://www.cyprotex.com/services, accessed 28 September, 2017).

Table S4. Free Fractions of In vivo Compounds in Biomedia

Compd	Assay medium, f_{ua}[a]	Species	Plasma, f_{up}[b]	Blood:Plasma, B:P[b]	Blood, f_{ub}[c]
		Athymic Mouse	0.025 (0.031-0.020)	1.7 (1.7-1.7)	0.015
**1**	0.47 (0.54-0.42)	BALB/c Mouse	0.010 (0.011-0.0099	1.2 (1.4-1.0)	0.0083
		Sprague Dawley Rat	0.029 (0.036-0.023)	1.5 (1.6-1.4)	0.019
**16**	ND	BALB/c Mouse	0.042 (0.071-0.024)	1.3 (1.5-0.9)	0.032
**17**	ND	BALB/c Mouse	0.022 (0.027-0.017)	1.5 (2.9-0.8)	0.015
**18**	0.16 (0.18-0.15)	BALB/c Mouse	0.012 (0.014-0.010)	1.1 (1.3-1.0)[g]	0.011
**21**	0.15 (0.15-0.14)	BALB/c Mouse	0.013 (0.015-0.011)	2.4 (2.9-2.0)[g]	0.0053
		Athymic Mouse	0.037 (0.044-0.031)	5.3 (5.5-5.2)	0.0070
		BALB/c Mouse	0.042 (0.045-0.040)	3.5 (3.6-3.4)	0.012
**22**	0.43 (0.45-0.41)	Sprague Dawley Rat	0.034 (0.041-0.029)	1.1 (1.2-0.9)	0.033
		Minipig[d]	0.11 (0.32-0.037)[f]	1.6 (2.0-1.3)	0.069
		Beagle Dog[d]	0.32 (0.40-0.25)[e]	2.3 (3.4-1.6)[e,f]	0.14
		Human	0.0037 (0.0052-0.0027)	0.7 (0.8-0.6)	0.0053

All data was reprocessed using GraphPad Prism Version 7.01. ND=not determined. The 90% confidence interval of the geometric mean is the parenthesis. [a]Measured by dialysis from the CellTiter-Blue assay medium. [b]Measured from n=3 statistical repeats of pooled sample unless otherwise stated. [c]f_{ub}=fraction unbound in blood, f_{up}= fraction unbound in plasma, B:P=blood to plasma ratio, $f_{ub}=f_{up}/B{:}P$ [d]Carried out at Cyprotex (http://www.cyprotex.com/admepk/protein_binding/plasma-protein-binding, 28 September 2017; http://www.cyprotex.com/admepk/protein_binding/blood-to-plasma-ratio, January 2017). [e]Measured in male dogs. [f]N=2. [g]Measured from pooled blood samples of both athymic and BALB/c mice.

Table S5. In vitro/in vivo Clearance Scaling Factors[1]

Species	Body Weight (kg)	Liver Weight per Body Weight (g/kg)	Liver weight (g)	Microsomal Protein per Liver Weight (mg/g)	Hepatocytes per Liver Weight (10^6 cells/g)	Q_H (mL/min/kg)
Mouse	0.02	88	1.75	45	135	90
Rat	0.25	40	10	45	135	70
Dog	12.5	32	400	45	240	40
Monkey	3.5	32	112	45	120	44
Human	70	20	1470	45	120	20

$$Predicted\ In\ vivo\ intrinsic\ clearance\ (pred.\ in\ vivo\ Cl_{int}) = \ In\ Vitro\ Clint * Microsomes\ or\ heps\ per\ liver * liver\ weight\ per\ body\ weight$$

Figure S1. Predicted in vivo Cl_{int} values were converted to predicted in vivo Cl_{tb} using the well-stirred model:

$$predicted\ in\ vivo\ Cl_{tb} = \frac{Q_H * f_{ub} * pred.\ in\ vivo\ Cl_{int}}{Q_H + f_{ub} * pred.\ in\ vivo\ Cl_{int}}$$

Cl_{tb}=total blood clearance, Cl_{int}=intrinsic clearance, Q_H=hepatic blood flow, f_{ub}=fraction unbound in blood.

Table S6. Summary of in vitro Hepatocyte Assay across Multiple Species for Bisamide 22

Species	In vitro Cl_{int} (μL/min/10^6 cells)	Predicted in vivo Cl_u (mL/min/kg)
Mouse	53	900
Rat	38	410
Minipig	860	ND
Dog	31	96
Human	9.0	54

All data was reprocessed using GraphPad Prism Version 7.01. ND= not determined. [h]Mouse hepatocytes assay was carried out at Cyprotex, n=1. In vitro Cl_{int} (μL/min/10^6 cells) is calculated from the half-life using standard procedures. [i]Assumes the fraction unbound in the assay is 0.4.

Figure S2. In vitro/in vivo Unbound Clearance Correlation for Bisamide 22

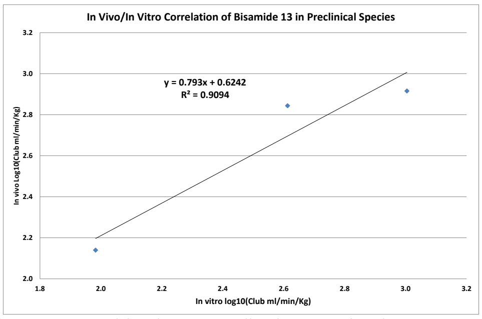

The correlation of unbound clearance values from in vitro hepatocytes and in vivo PK studies in BALB/c mice SD rats and beagle dogs was used to normalize the predicted human unbound clearance from human hepatocytes.[1,2]

Figure S3. Predicted Efficacious Human Dose of Bisamide 22

$$Dose = \frac{AUC_u^{0-24h} * Cl_u}{F}$$

$AUC_u^{0\text{-}24h}$ was assumed from the mouse efficacy study. Cl_u was taken as either the value predicted from human hepatocytes or from rat single species scaling. F=oral bioavailability and was assumed to be 0.4.

Table S7. Plasma Stability Assay of Bisamide 1

Compd	Species	Compound remaining (% of 0 min)				
		0 min	15 min	30 min	60 min	120 min
1	Mouse	100	97.8	91.3	93.3	85.9
1	Human	100	104	96.4	97.0	95.1

Compounds were tested at 1 µM in pooled plasma, 2.5% DMSO, 37 ˚C, results are single-point data.

Table S8. CACO-2 In vitro Permeability Assay Data

Compd	Analysis	Exp.[a]	A2B (nm/s)[b]	Recovery (%)[c]	A2B + LY335979 (nm/s)[d]	Recovery (%)[c]	B2A (nm/s)[e]	Recovery (%)[c]	B2A + LY335979 (nm/s)[d]	Recovery (%)[c]	Efflux Ratio (B2A/A2B)[f]	Efflux Ratio + LY335979 (B2A/A2B)[f]
1		1	0.57	ND	ND	ND	23	ND	ND	ND	16 (130-2.0, p=0.058)	6.5
		2	3.1	90	2.3	50	46	70	15	100		
		3	8.1	ND	ND	ND	55	ND	ND	ND		
	Geometric Mean (90% CI)	/	2.4 (23-0.25)	90	2.3	50	39 (82-18)	70	15	100		
22		4	5.3	50	8.3	40	17	70	9	80	2.8 (6.1-1.3, p=0.039)	1.5 (2.4-0.96, p=0.12)
		5	15	70	14	50	45	100	19	80		
		6	11	80	8.4	50	15	100	13	70		
		7	4.1	50	5.9	50	20	70	14	70		
	Geometric Mean (90% CI)	/	7.7 (16-3.7)	60	8.7 (13-5.7)	50	21 (39-12)	80	13	80		

All data was reprocessed using GraphPad Prism Version 7.01. ND=not determined. Each individual value represents the arithmetic mean of 3 statistical repeats. [a]Each experiment represents an independent biological repeat. [b]A2B=permeability rate from apical to basolateral layer. [c]Total compound recovered from the assay. [d]The PGP-inhibitor LY335979 was purchased from Selleckchem (http://www.selleckchem.com/products/LY335979.html?gclid=Cj0KEQiAtqHEBRCNrdC6rYq9_oYBEiQAejvRlx363RNFLpuolVytgelcRwxlV63cXpyPHe4pxhZXulYaAhdQ8P8HAQ, accessed September 2017) and was used in cells at 5 µM. [e]B2A=permeability rate from basolateral to apical layer. [f]Calculated from the ratio geometric mean permeability rates, the 90% CI of this ratio is described the parenthesis, the p-value was calculated by comparing the geometric mean of the relevant permeability rates using a two-tailed Student's t-test with Welch's correction.

Figure S4. Rescue of Multidrug Resistance with the PGP Inhibitor Verapamil

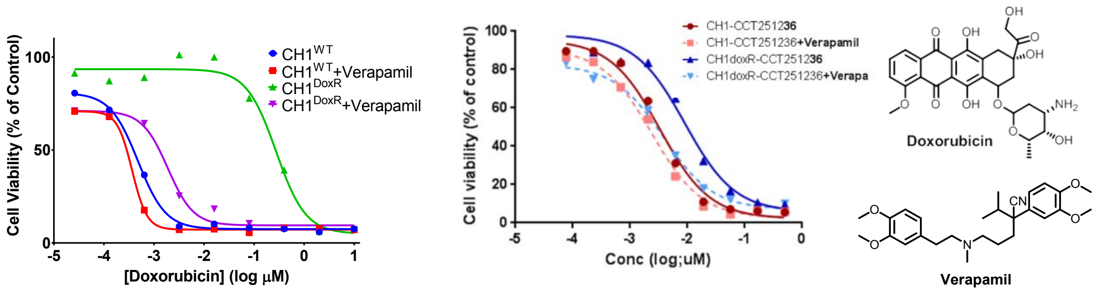

Ovarian Cancer Cell line	Verapamil (PGP-inhibitor)	Doxorubicin GI_{50} (nM)	Chemical probe **1** GI_{50} (nM)
$CH1^{WT}$	No	0.4	33
$CH1^{WT}$	Yes	0.3	24
$CH1^{doxR}$	No	305	90
$CH1^{doxR}$	Yes	1.9	38

The antiproliferative activity of the cytotoxic doxorubicin was assayed in the human ovarian cancer cell line $CH1^{WT}$ and an acquired doxorubicin-resistant cell line $CH1^{doxR}$. Growth inhibition was measured after 96 hours of treatment and compared to the vehicle control in the CellTiter-Blue assay. In the wild-type cell line, no difference in antiproliferative activity was observed with or without co-treatment with the PGP-inhibitor verapamil (2 µM); in the doxorubicin-resistant cell line the antiproliferative activity of doxorubicin is rescued to basal levels after treatment with verapamil.

Figure S5. Analysis of Ortho-Substituents on the Benzamide Conformation

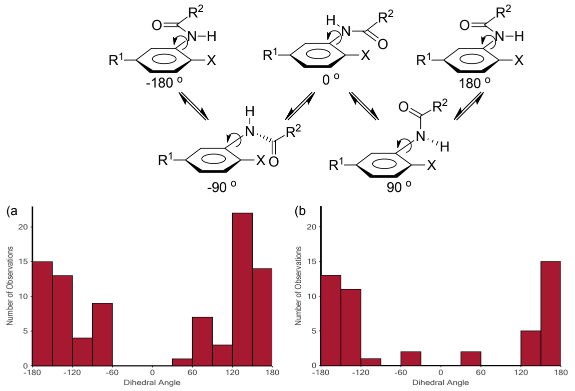

Histograms showing the distribution of dihedral angles of *ortho*-substituted benzamides (along the highlighted bonds) retrieved from the Cambridge Structural Database. (a) *ortho*-methylbenzamides (76 crystal structures, 88 dihedral angle observations); (b) *ortho*-fluorobenzamides (41 crystal structures, 49 dihedral angle observations).

Table S9. Cerep SafetyScreen-87 for CCT361814 Bisamide 22

The 44 selected targets in the safety screen profiling panel are recommended by 4 major pharmaceutical companies[3] and provides early identification of significant off-target interactions for the optimization of safety margins. SafetyScreen-87 is an extension of the SafetyScreen-44 that is enhanced with additional targets providing a more thorough assessment of potential safety liabilities. This assay package consists of 87 primary molecular targets including 13 enzyme and 74 binding assays. http://www.cerep.fr/cerep/utilisateurs/pages/Downloads/Documents/Marketing/Pharmacology%20&%20ADME/Standard%20profiles/SafetyScreen87_2014v2LD.pdf, accessed September, 2017).

Assay	Species	% Inh.	Assay	Species	% Inh.
ATPase, Na^+/K^+, heart	pig	5	Glucocorticoid	human	9
Cholinesterase, Acetyl, ACES	human	2	Glutamate, AMPA	rat	5
Cyclooxygenase COX-1	human	-24	Glutamate, Metabotropic, $mGlu_5$	human	-2
Cyclooxygenase COX-2	human	-5	Glutamate, Kainate	rat	-3
**Monoamine Oxidase MAO-A**	**human**	**77**	Glutamate, NMDA, Agonism	human	2
Monoamine Oxidase MAO-B	human	36	Glutamate, NMDA, Glycine	rat	-24
Peptidase, Angiotensin Converting Enzyme	rabbit	13	Glutamate, NMDA, Phencyclidine	rat	7
Peptidase, CTSG (Cathepsin G)	human	1	Glutamate, NMDA, Polyamine	rat	3
Phosphodiesterase PDE3	human	3	Glycine, Strychnine-sensitive	rat	-3
Phosphodiesterase PDE4	human	37	Histamine H_1	rat	36
Protein Serine/Threonine Kinase, PKC, Non-Selective	rat	9	**Histamine H_2**	**human**	**62**
Protein Tyrosine Kinase, Insulin Receptor	human	-7	Leukotriene, Cysteinyl $CysLT_1$	human	7
Protein Tyrosine Kinase, LCK	human	-8	Melanocortin MC_1	human	-3
Adenosine A1	human	11	Melanocortin MC_4	human	2
**Adenosine A_{2A}**	**human**	**91**	Muscarinic M_1	human	43
Adrenergic α_{1A}	rat	38	**Muscarinic M_2**	**human**	**52**
Adrenergic α_{1B}	rat	9	Muscarinic M_3	human	30
Adrenergic α_{1D}	human	25	Muscarinic M_4	human	40
**Adrenergic α_{2A}**	**human**	**80**	Neuropeptide YY_1	human	13
**Adrenergic α_{2B}**	**human**	**79**	Nicotonic Acetylcholine	human	36
Adrenergic β_1	human	4	Nicotonic Acetylcholine α1, Bungarotoxin	human	39
Adrenergic β_2	human	12	Opiate δ_1 (OP1, DOP)	human	9
Androgen (Testosterone)	human	33	**Opiate κ (OP2, KOP)**	**human**	**51**
Angiotensin AT_1	human	1	Opiate μ (OP3, MOP)	human	44
Bradykinin B_2	human	0	Platelet Activating Factor (PAF)	human	15
Calcium Channel L-type, Benzothiazepine	rat	11	Potassium Channel [K_{ATP}]	human	-5
Calcium Channel L-type, Dihydropyridine	rat	14	**Potassium Channel hERG**	**human**	**62**
Calcium Channel L-type, Phenylalkylamine	rat	19	PPARγ	human	11
Calcium Channel N-type	rat	9	Progesterone PR-B	human	5
Cannabinoid CB_1	human	9	Serotonin (5-Hydroxytryptamine) 5-HT_{1A}	human	4
**Cannabinoid CB_2**	**human**	**79**	Serotonin (5-Hydroxytryptamine) 5-HT_{1B}	human	43
Chemokine CCR1	human	2	**Serotonin (5-Hydroxytryptamine) 5-HT_{2A}**	**human**	**64**
Chemokine CXCR2 (IL-$8R_B$)	human	7	**Serotonin (5-Hydroxytryptamine) 5-HT_{2B}**	**human**	**72**
Cholecystokinin CCK_1 (CCK_A)	human	9	**Serotonin (5-Hydroxytryptamine) 5-HT_{2C}**	**human**	**55**
Cholecystokinin CCK_1 (CCK_B)	human	-4	Serotonin (5-Hydroxytryptamine) 5-HT_3	human	-10
Dopamine D_1	human	41	Sodium Channel, Site 2	rat	28
Dopamine D_{2L}	human	20	Tachykinin NK_1	human	14
Dopamine D_{2S}	human	5	**Transporter, Adenosine**	**Guinea pig**	**72**
Endothelin ET_A	human	-2	Transporter, Dopamine (DAT)	human	38
Estrogen ER_α	human	-1	Transporter, GABA	rat	-7
$GABA_A$, Chloride Channel, TBOB	rat	12	Transporter, Norepinephrine (NET)	human	42
$GABA_A$, Flunitrazepam, Central	rat	6	Transporter, Serotonin (5-Hydroxytryptamine) (SERT)	human	25
$GABA_A$, Ro-15-1788, Hippocampus, Central	rat	9	Vasopressin V_{1A}	human	20
$GABA_{B1A}$	human	4			

Compound **22** was screened at 10 μM and the %inhibition represents the arithmetic mean of n=2 repeats. Proteins in bold were defined as significant (>50%) hits for further study.

Figure S6. Follow-up Assays for Bisamide 22

The significant hits (>50%@10 μM) from the SafetySceen-87 assay with assessed in individual cell-based assay for both agonism and antagonism of the relevant receptor where possible. No activity was observed in the agonism mode and only adenosine A2A confirmed as an antagonist (http://www.cerep.fr/cerep/users/pages/catalog/Affiche_CondExp_Test.asp?test=2527http://www.cerep.fr/cerep/users/pages/catalog/Affiche_CondExp_Test.asp?test=2527, accessed September 2017). IC_{50}=2.0 μM.

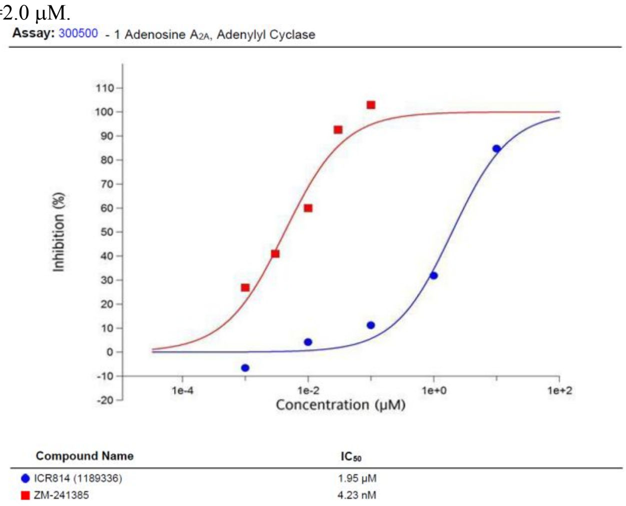

Figure S7. CYP450 Isoform Inhibition with Bisamide 22

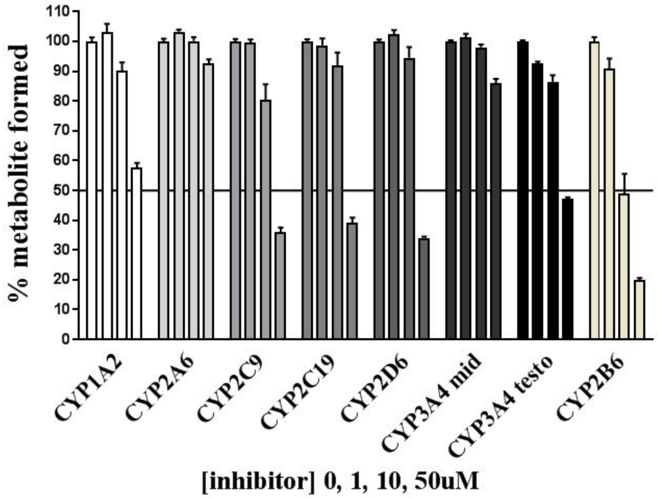

No significant CYP450 inhibition was observed at free concentrations relevant to the in vivo activity. See experimental section for details. Two structurally unrelated compounds (mid=midazolam; testo=testosterone) were used as CYP3A4 substrates. 1A2: Phenacetin 2A6: Coumarin 2B6: Bupropion 2C9: Tolbutamide 2C19: Mephenytoin 2D6: Bufuralol 3A4: Midazolam and testosterone

Table S10. hERG Inhibition with Bisamide 22

Bisamide **22** was tested in the Cyprotex hERG safety assay, a cell-based assay which employs the Ionworks™ HT System (Molecular Devices) as an automated patch clamp electrophysiology measurement (http://www.cyprotex.com/toxicology/cardiotoxicity/hergsafety, accessed September, 2017). IC_{50}=33.3 µM (SE IC_{50}=10.1 µM, n=16).

**Compd**	**% Mean Inhibition**						
	**0 µM**	**0.016 µM**	**0.08 µM**	**0.4 µM**	**2 µM**	**10 µM**	**50 µM**
**22**	0	-4.80	-8.67	-11.0	-1.14	22.3	46.9

Table S11. Thermodynamic Solubility Data for Bisamide 22 in Simulated Bio-relevant Fluids

Aqueous thermodynamic solubility was measured at a concentration of 1 mg/mL. Compound **22** was equilibrated in Fasted State Simulated Gastric Fluid (FaSSGF), pH 1.6; in Fasted State Simulated Intestinal Fluid (FaSSIF), pH 6.5; or in Fed State Simulated Intestinal Fluid (FeSSIF), pH 6.5 (http://www.pharmidex.com/service/physiocochemical-properties, accessed September, 2017).

**Compd**	**mg/mL**	**Assay**	**Concentration (mg/mL)**	**Concentration (µM)**
		FaSSGF	0.920*	1600
**22**	1	FaSSIF	0.027	48
		FeSSIF	0.034	60

*top limit of the assay.

Pharmacokinetic and in vivo efficacy data

Dosing vehicle

All animals were dosed po and iv using our standard 25 % w/v hydroxypropyl β-cyclodextrin in 50 mM sodium citrate buffer pH 5 vehicle unless otherwise stated. The formulation (10% DMSO, 90 % of a 25 % (2-hydroxypropyl)-β-cyclodextrin in 50 mM citrate buffer pH 5) was prepared by dissolving citric acid monohydrate (10.5 g) and trisodium citrate dehydrate (14.8 g) in sterile water (500 mL, respectively). The citric acid solution (87.5 mL) was then added to the sodium citrate dehydrate solution (163 mL) to generate the pH 5 citrate buffer. (2-hydroxypropyl)-β-cyclodextrin (50 g, average MW~1460 g/mol) was then added to the pH 5 citrate buffer (150 mL) and the pH measured to be 5.07. The bisamide was dissolved in DMSO (~20 mL) and then added to the 25 % (2-hydroxypropyl)-β-cyclodextrin in 50 mM citrate buffer pH 5 (~180 mL). The mixture was then sonicated to give a clear solution. Unused formulation was stored for up to 1 week at 5 °C.

Table S12. In Vivo Blood PK Profiles of the Selected Bisamides in Rodent and Non-Rodent Species

Compd	Species	Dose po/iv (mg/kg)	T_{max} (h)	C_{max} (nM)	AUC^{0-t} (h*nM)	Cl_{tb} (mL/min/kg)[a]	V_{ss} (L/kg)[a]	$t_{1/2}$ (h)	F (%)[i]	f_{ub}	AUC_u^{0-t} (h*nM)[b]	Free C_{av}^{0-24h} (nM)[c]	Cl_u (mL/min/kg)[d]	V_{du} (L/kg)[e]
16	Mouse	5/5	1.7	130 (420-41)	430 (890-214)[f]	34 (36-32)	2.6 (2.9-2.3)	1.2	11	0.032	14	0.66	1100	80
17	Mouse	5/5	2	180 (210-160)	830 (910-750)[f]	40 (48-32)	5.1 (6.0-4.4)	1.7	24	0.015	12	0.83	2700	340
18	Mouse	5/1[j]	1.7	550 (670-450)	2400 (2800-2000)[f]	33 (35-31)	4.9 (7.9-3.4)	2.0	63	0.011	26	1.6	3000	450
21	Mouse	5/5	1.7	880 (1200-650)	3900 (4900-3000)[f]	28 (33-25)	5.7 (6.5-4.9)	2.3	100	0.0053	21	1.3	5300	1100
22	Mouse	5/5	2.0	1500 (1800-1300)	6000 (7800-4600)[g]	10 (10-9.7)	1.9 (2.3-1.6)	4.0	42	0.012	72	3.3	830	160
1	Rat	5/5	>6	63 (86-45)	840 (1000-700)[g]	20 (30-14)	4.4 (5.7-3.4)	3.2	11	0.019	16	0.72	1100	230
22	Rat[h]	5/1[j]	6	240	2600[g]	24	7.3	3.1	45	0.033	86	3.7	730	220
22	Dog[a]	2.5/0.5[j]	2.0	69	250[f]	21	2.2	1.4	9.1[i]	0.14	35	1.9	150	16

All values are quoted to 2 SF and are the geometric mean of n=3 individual animal unless otherwise stated. Immunocompetent BALB/c mice, SD rats and beagle dogs were used. PK parameters are calculated from the blood concentration-time curve using standard methods unless otherwise stated. All values are quoted to 2 SF. The 90% confidence intervals are in parentheses. [a]This study was carried out at Charles River Laboratories http://www.criver.com/products-services/safety-assessment/toxicology/drug-metabolism-pharmacokinetics/pk, January 2016. The geometric mean of n=4 individual dogs (2 males and 2 females). PK parameters were calculated from the plasma concentration-time curve from 0-6 h and were converted to blood PK parameters using the blood to plasma ratio. [b]$AUC_u=AUC*f_{ub}$. [c]free $C_{av}^{0-24h}=AUC_u^{inf}/24$. [d]$Cl_u=Cl/f_{ub}$. [e]$V_{du}=V_{ss}/f_{ub}$. [f]t=6 h. [g]t=24 h. [h]The rat blood PK was determined as a composite profile of 6 animals. [i]Calculated from po AUC^{last}/iv AUC^{last}Assumes linear scaling of AUC with dose.

Figure S8. In vivo Validation of Multidrug Resistance Strategy

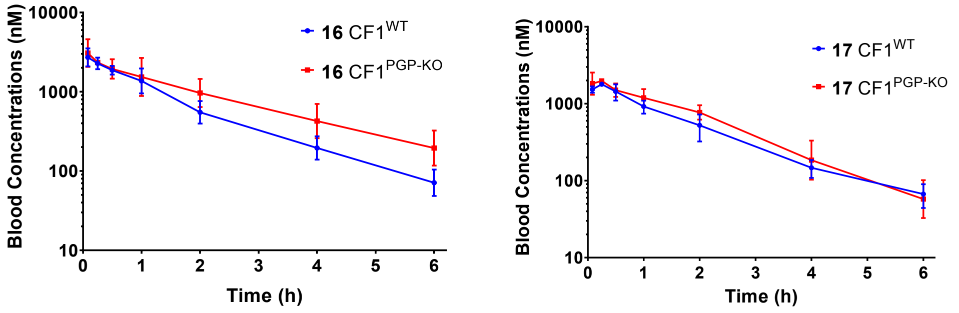

Compd	Dose iv (mg/kg)	mouse strain	Cl_{tb} (mL/min/kg)	Extraction Ratio (% of HBF)	p-value	Vss (L/kg)	p-value
16	5	CF1[WT]	35 (42-30)	39 (46-33)	0.024	3.2 (3.7-2.8)	0.39
		CF1[PGP-KO]	24 (31-19)	27 (21-34)		3.6 (4.4-2.9	
17		CF1[WT]	47 (62-35)	52 (39-70)	0.13	4.7 (5.4-4.0)	0.030
		CF1[PGP-KO]	38 (43-37)	42 (47-37)		3.6 (3.5-3.8)	

All data was reprocessed using GraphPad Prism Version 7.01. The graph data points represent the geometric mean±95% confidence interval of n=3 individual mice. PK parameters were calculated from blood concentrations using standard procedures. The values in the table represent the geometric mean of n=3 mice with the 90% confidence interval in parentheses, p-values were calculated using Student's t-test with Welch's correction. Cl_{tb}=total blood clearance, V_{ss}=steady state volume of distribution. The extraction ratio was calculated by assuming that mouse hepatic blood flow is 90 mL/min/kg.

Figure S9. Immunodeprived Athymic Mice Tolerability Study of Bisamide 22

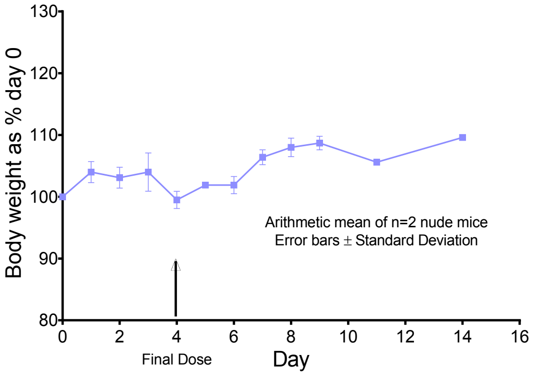

All data was reprocessed using GraphPad Prism Version 7.01. Bisamide **22** was dosed 50 mg/kg po qd as a solution in our standard vehicle ((Vehicle=10% DMSO, 90% of a 25% (2-hydroxypropyl)-β-cyclodextrin in 50 mM citrate buffer pH 5) for 4 days into non-tumor bearing athymic mice. Body weights were measured once-a-day.

Figure S10. Mouse Body Weights During Bisamide 22 Efficacy Study

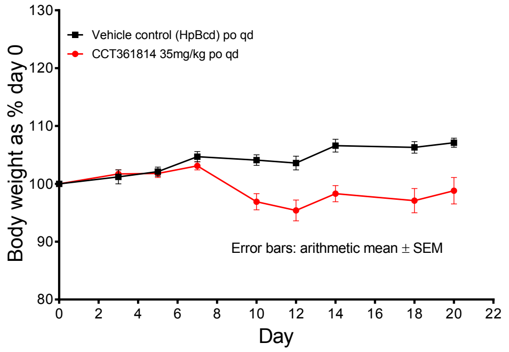

All data was reprocessed using GraphPad Prism Version 7.01. SK-OV-3 derived tumor bearing immunocompromised athymic mice were dose with 35 mg/Kg po qd. Vehicle control n=10, bisamide **22** (CCT361814) 35 mg/kg po qd n=10 (Vehicle=10% DMSO, 90% of a 25% (2-hydroxypropyl)-β-cyclodextrin in 50 mM citrate buffer pH 5).

Figure S11. Tumor Weights at the End of Bisamide 22 Efficacy Study

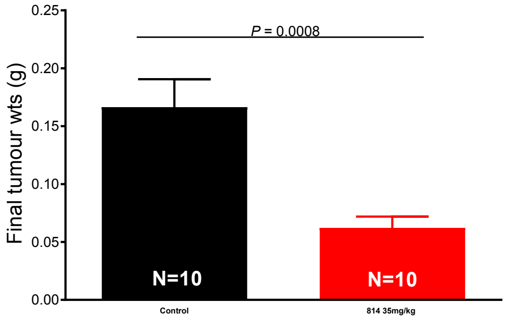

All data was reprocessed using GraphPad Prism Version 7.01. SK-OV-3 derived tumor bearing immunocompromised athymic mice were dose with 35 mg/kg po qd. Vehicle control n=10, bisamide **22** (CCT361814) 35 mg/kg po qd n=10 (Vehicle=10% DMSO, 90% of a 25% (2-hydroxypropyl)-β-cyclodextrin in 50 mM citrate buffer pH 5). P-values were calculated by comparison of the arithmetic means of treated and non-treated tumors using a 2-tailed Students t-test.

Table S13 Statistical Analysis of Fluorobisamide 22 SKOV3 Efficacy Study

Tumor growth:

Two-way ANOVA	Ordinary				
Alpha	0.05				
Source of Variation	% of total variation	P value	P value summary	Significant?	
Interaction	14.91	<0.0001	****	Yes	
Row Factor	7.4	0.0032	**	Yes	
Column Factor	28.5	<0.0001	****	Yes	
ANOVA table	SS	DF	MS	F (DFn, DFd)	P value
Interaction	0.1364	8	0.01706	F (8, 162) = 6.14	P<0.0001
Row Factor	0.0677	8	0.008462	F (8, 162) = 3.046	P=0.0032
Column Factor	0.2607	1	0.2607	F (1, 162) = 93.84	P<0.0001
Residual	0.45	162	0.002778		
Number of missing values	0				
Number of families	1				
Number of comparisons per family	9				
Alpha	0.05				
Sidak's multiple comparisons test	Mean Diff.	95.00% CI of diff. Significant? Summary Adjusted P Value			

**Vehicle Control** - 35 mg/Kg po qd					
0	-0.006	-0.07207 to 0.06007	No	ns	>0.9999
3	0.018	-0.04807 to 0.08407	No	ns	0.9951
5	0.034	-0.03207 to 0.1001	No	ns	0.7710
7	0.04	-0.02607 to 0.1061	No	ns	0.5788
10	0.082	0.01593 to 0.1481	Yes	**	0.0058
12	0.098	0.03193 to 0.1641	Yes	***	0.0005
14	0.109	0.04293 to 0.1751	Yes	****	<0.0001
18	0.15	0.08393 to 0.2161	Yes	****	<0.0001
20	0.16	0.09393 to 0.2261	Yes	****	<0.0001

Test details	Mean 1	Mean 2	Mean Diff.	SE of diff.	N1	N2	t	DF
**Vehicle Control** - 35 mg/Kg po qd								
0	0.078	0.084	-0.006	0.02357	10	10	0.2546	162
3	0.097	0.079	0.018	0.02357	10	10	0.7637	162
5	0.112	0.078	0.034	0.02357	10	10	1.442	162
7	0.121	0.081	0.04	0.02357	10	10	1.697	162
10	0.148	0.066	0.082	0.02357	10	10	3.479	162
12	0.162	0.064	0.098	0.02357	10	10	4.158	162
14	0.168	0.059	0.109	0.02357	10	10	4.624	162
18	0.214	0.064	0.15	0.02357	10	10	6.364	162
20	0.22	0.06	0.16	0.02357	10	10	6.788	162

Table S14 Statistical Analysis of Fluorobisamide 22 SKOV3 PKPD Study

CHAC1 PD Biomarker:

1Way ANOVA					
Data sets analyzed	A : Control	B : 1 hr	C : 4 hrs	D : 6 hrs	E : 8 hrs
ANOVA summary					
F	9.037				
P value	<0.0001				
P value summary	****				
Significant diff. among means (P < 0.05)?	Yes				
R square	0.5879				
Brown-Forsythe test					
F (DFn, DFd)	1.302 (6, 38)				
P value	0.2798				
P value summary	ns				
Are SDs significantly different (P < 0.05)?	No				
Bartlett's test					
Bartlett's statistic (corrected)					
P value					
P value summary					
Are SDs significantly different (P < 0.05)?					
ANOVA table	SS	DF	MS	F (DFn, DFd)	P value
Treatment (between columns)	2.047	6	0.3411	F (6, 38) = 9.037	P<0.0001
Residual (within columns)	1.435	38	0.03775		
Total	3.481	44			
Data summary					
Number of treatments (columns)	7				
Number of values (total)	45				

Number of comparisons per family	6						
Alpha	0.05						
Dunnett's multiple comparisons test	Mean Diff.	95.00% CI of diff.	Significant?	Summary	Adjusted P Value	A-?	
Control vs. 1 hr	-0.1438	-0.4265 to 0.139	No	ns	0.5698	B	1 hr
Control vs. 4 hrs	-0.4241	-0.7069 to -0.1414	Yes	**	0.0014	C	4 hrs
Control vs. 6 hrs	-0.6523	-0.9727 to -0.3319	Yes	****	0.0001	D	6 hrs
Control vs. 8 hrs	-0.3212	-0.6039 to -0.03838	Yes	*	0.0206	E	8 hrs
Control vs. 16 hrs	-0.3607	-0.6435 to -0.07791	Yes	**	0.0077	F	16 hrs
Control vs. 24 hrs	0.09296	-0.2275 to 0.4134	No	ns	0.9294	G	24 hrs

Test details	Mean 1	Mean 2	Mean Diff.	SE of diff.	n1	n2	q	DF
Control vs. 1 hr	2.854	2.998	-0.1438	0.1053	23	4	1.366	38
Control vs. 4 hrs	2.854	3.278	-0.4241	0.1053	23	4	4.029	38
Control vs. 6 hrs	2.854	3.506	-0.6523	0.1193	23	3	5.469	38
Control vs. 8 hrs	2.854	3.175	-0.3212	0.1053	23	4	3.051	38
Control vs. 16 hrs	2.854	3.215	-0.3607	0.1053	23	4	3.427	38
Control vs. 24 hrs	2.854	2.761	0.09296	0.1193	23	3	0.7794	38

Table S15. HSPA1 mRNA Cycle Threshold

Table Analyzed	Data 10				
Data sets analyzed	A : Control	B : 1 h	C : 4 h	D : 6 h	E : 8 h
ANOVA summary					
F	11.52				
P value	<0.0001				
P value summary	****				
Significant diff. among means (P < 0.05)?	Yes				
R square	0.6452				
Brown-Forsythe test					
F (DFn, DFd)	1.859 (6, 38)				
P value	0.1136				
P value summary	ns				
Are SDs significantly different (P < 0.05)?	No				
Bartlett's test					
Bartlett's statistic (corrected)					
P value					
P value summary					
Are SDs significantly different (P < 0.05)?					
ANOVA table	SS	DF	MS	F (DFn, DFd)	P value
Treatment (between columns)	7.749	6	1.291	F (6, 38) = 11.52	P<0.0001
Residual (within columns)	4.261	38	0.1121		
Total	12.01	44			
Data summary					
Number of treatments (columns)	7				
Number of values (total)	45				

Number of families	1							
Number of comparisons per family	6							
Alpha	0.05							
Dunnett's multiple comparisons test	Mean Diff.	95.00% CI of diff.	Significant?	Summary	Adjusted P Value	A-?		
Control vs. 1 h	-0.2723	-0.7596 to 0.2151	No	ns	0.4772	B	1 h	
Control vs. 4 h	1.065	0.5128 to 1.617	Yes	****	0.0001	C	4 h	
Control vs. 6 h	1.206	0.6535 to 1.758	Yes	****	0.0001	D	6 h	
Control vs. 8 h	0.4852	-0.002131 to 0.9726	No	ns	0.0514	E	8 h	
Control vs. 16 h	0.04524	-0.4421 to 0.5326	No	ns	0.9996	F	16 h	
Control vs. 24 h	0.4475	-0.03988 to 0.9349	No	ns	0.0822	G	24 h	
Test details	Mean 1	Mean 2	Mean Diff.	SE of diff.	n1	n2	q	DF
Control vs. 1 h	-0.0002608	0.272	-0.2723	0.1814	23	4	1.501	38
Control vs. 4 h	-0.0002608	-1.065	1.065	0.2056	23	3	5.181	38
Control vs. 6 h	-0.0002608	-1.206	1.206	0.2056	23	3	5.866	38
Control vs. 8 h	-0.0002608	-0.4855	0.4852	0.1814	23	4	2.675	38
Control vs. 16 h	-0.0002608	-0.0455	0.04524	0.1814	23	4	0.2494	38
Control vs. 24 h	-0.0002608	-0.4478	0.4475	0.1814	23	4	2.467	38

ER-Stress in vivo Biomarker Discovery

Figure S12. CHAC1 mRNA induction following treatment with compound 22

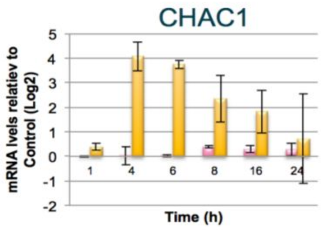

Increased levels of CHAC1 gene expression as measured by mRNA after treatment with bisamide **22** (50 mg/kg) yellow (dosing vehicle in pink) in a 1 day PK/PD experiment in SK-OV-3 human ovarian cancer xenograft.

Figure S13. In vitro Induction of CHAC1

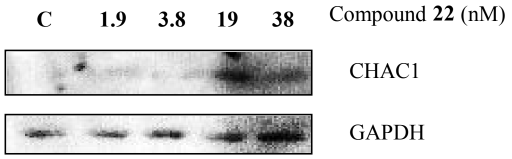

SK-OV-3 cells were treated with compound **22** for 48 h. The concentrations used are quoted as free concentrations (f_{ua}=0.44).

Figure S14. In vivo Induction of CHAC1 by Western Blot Assay

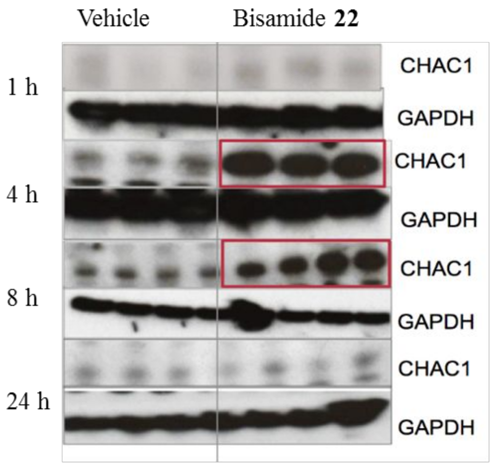

Clear induction of CHAC1 protein in SK-OV-3 tumors following a single 50 mg/kg po dose in athymic mice as shown in the Western blot at 4 h and 8 h.

Figure S15. CHAC1 MSD Assay Validation with Recombinant Protein

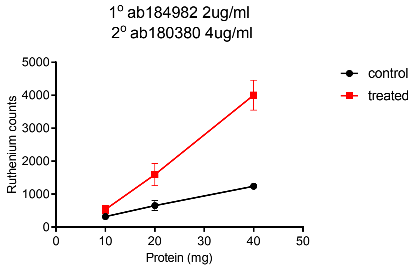

Selected validation curves for CHAC1 antibodies ab184982 and ab180380.

Chemistry Experimental Procedures

All final compounds were screened through our in-house computational PAINS filter and gave no structural alerts as potential assay interference compounds.[4] Unless otherwise stated, reactions were conducted in oven dried glassware under an atmosphere of nitrogen or argon using anhydrous solvents. All commercially obtained reagents and solvents were used as received. Thin layer chromatography (TLC) was performed on pre-coated aluminum sheets of silica (60 F254 nm, Merck) and visualized using short-wave UV light. Flash column chromatography was carried out on Merck silica gel 60 (particle size 40-65 μm). Column chromatography was also performed on Biotage SP1 or Isolera 4 purification systems using Biotage Flash silica cartridges (SNAP KP-Sil). Ion exchange chromatography was performed using acidic Biotage Isolute Flash SCX-2 columns.

Semi-preparative HPLC: 500 uL standard injections (with needle wash) of the sample were made on a Phenomenex Gemini C18 column (5μ, 250x21.2 mm, Phenomenex, Torrence, USA). Chromatographic separation at room temperature was carried out using a 1200 Series Preparative HPLC (Agilent, USA) over a 15 minutes gradient elution from 90:10 to 0:100 water:methanol (both modified with 0.1% formic acid) at a flow rate of 20 mL/min. UV-Vis spectra were acquired at 254 nm on a 1200 Series Prep Scale diode array detector (Agilent). Post-UV and pre-MS splitting was achieved using an Active Split (Agilent) before being infused into a 6120 Series Quad mass spectrometer fitted with an ESI/APCI Multimode ionization source (Agilent). Collection was triggered by UV signal and collected on a 1200 Series Fraction Collector (Agilent). ^{1}H-NMR spectra were recorded on Bruker Avance 500 (500 MHz) spectrometers using an internal deuterium lock. Chemical shifts are quoted in parts per million (ppm) using the following internal references: $CDCl_3$ (δH 7.26), MeOD (δH 3.31) and DMSO-d_6 (δH 2.50). Signal multiplicities are recorded as singlet (s), doublet (d), triplet (t), quartet (q), quintet (qn), and multiplet (m), doublet of doublets (dd), doublet of doublet of doublets (ddd), broad (br), obscured (obs) or apparent (app). Coupling constants, *J*, are measured to the nearest 0.1 Hz. ^{13}C-NMR spectra were recorded on Bruker Avance 500 spectrometers at 126 MHz using an internal deuterium lock. Chemical shifts are quoted to 0.01 ppm, unless greater accuracy was required, using the following internal references: $CDCl_3$ (δC 77.0), MeOD (δC 49.0) and DMSO-d_6 (δC 39.5). High resolution mass spectra were recorded on an Agilent 1200 series HPLC and diode array detector coupled to a 6210 time of flight mass spectrometer with dual multimode APCI/ESI source (Methods I-IV) or on a Waters Acquity UPLC and diode array detector coupled to a Waters G2 QToF mass spectrometer fitted with a multimode ESI/APCI source (Methods V-VI). Analytical separation was carried out according to the methods listed below. The mobile phase was a mixture of methanol (solvent A) and water (solvent B), both containing formic acid at 0.1%, UV detection was at 254 nm.

Method I: Analytical separation was carried out at 30ºC on a Merck Purospher STAR column (RP-18e, 30 x 4 mm) using a flow rate of 1.5 mL/min in a 4 minute gradient elution. Gradient elution was as follows: 10:90 (A/B) to 90:10 (A/B) over 2.5 min, 90:10 (A/B) for 1 min, and then reversion back to 10:90 (A/B) over 0.3 min, finally 10:90 (A/B) for 0.2 min. Method II: Analytical separation was carried out at 30ºC on a Merck Chromolith Flash column (RP-18e, 25 x 2 mm) using a flow rate of 0.75 mL/min in a 4 minute gradient elution. Gradient elution was as follows: 5:95 (A/B) to 100:0 (A/B) over 2.5 min, 100:0 (A/B) for 1 min, and then reversion back to 5:95 (A/B) over 0.1 min, finally 5:95 (A/B) for 0.4 min. Method III: Analytical separation was carried out at 40ºC on a Merck Purospher STAR column (RP-18e, 30 x 4 mm) using a flow rate of 3 mL/min in a 2 minutes gradient elution. Gradient elution was as follows: 10:90 (A/B) to 90:10 (A/B) over 1.25 min, 90:10 (A/B) for 0.5 min, and then reversion back to 10:90 (A/B) over 0.15 min, finally 10:90 (A/B) for 0.1 min. Method IV: Analytical separation was carried out at 40ºC on a Merck Purospher STAR column (RP-18e, 30 x 4 mm) using a flow rate of 1.5 mL/min in a 2 minutes gradient elution. Gradient elution was as follows: 5:95 (A/B) to 100:0 (A/B) over 1.25 min, 100:0 (A/B) for 0.5 min, and then reversion back to 5:95 (A/B) over 0.05 min, finally 5:95 (A/B) for 0.2 min. Method V: Waters Acquity UPLC, Phenomenex Kinetex XB-C18 column (30 x 2.1 mm, 1.7u, 100A) at 30 °C using flow rate of 0.3 mL/min in a 4 minute gradient elution. Gradient elution was as follows: 10:90 (A/B) to 90:10 (A/B) over 3 min, 90:10 (A/B) for 0.5 min, and then reversion back to 10:90 (A/B) over 0.3 min, finally 10:90 (A/B) for 0.2 min. Method VI: Waters Acquity UPLC, Phenomenex Kinetex C18 column (30 x 2.1 mm, 2.6u, 100A), flow rate and gradient elution according to Method V. The following reference masses were used for HRMS analysis: Agilent 1200 series: caffeine $[M + H]^+$ 195.087652; hexakis(1*H*,1*H*,3*H*-tetrafluoropentoxy)phosphazene $[M + H]^+$ 922.009798 and hexakis(2,2-difluoroethoxy)phosphazene $[M + H]^+$ 622.02896 or reserpine $[M + H]^+$ 609.280657; Waters Acquity UPLC: Leucine Enkephalin fragment ion $[M + H]^+$ 397.1876. All compounds were >95 % purity by LCMS analysis unless otherwise stated.

Synthetic Route I

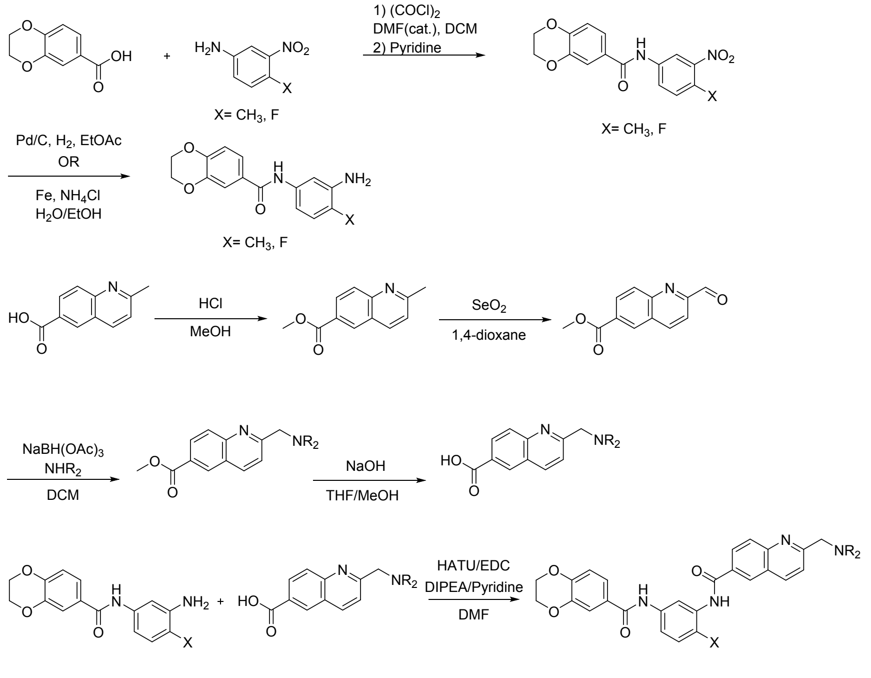

N-(3-Amino-4-methylphenyl)-2,3-dihydrobenzo[*b*][1,4]dioxine-6-carboxamide **S1**

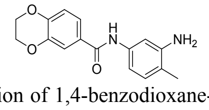

Oxalyl chloride (1.40 mL, 16.6 mmol) was added dropwise to a solution of 1,4-benzodioxane-6-carboxylic acid (2.49 g, 13.8 mmol) and DMF (0.027 mL, 0.340 mmol) in anhydrous DCM (34 mL). The reaction mixture was stirred at room temperature for 3.5 h, and then concentrated. The residue was dissolved in DCM and concentrated again. This residue was dissolved in anhydrous DCM (12 mL) and added dropwise to a solution of 4-methyl-3-nitroaniline (2.10 g, 13.8 mmol) and pyridine (2.23 mL, 27.6 mmol) in anhydrous DCM (25 mL). The reaction mixture was stirred at room temperature for 2 h, and then concentrated. The resulting

solid was suspended in MeOH, diluted with water and then isolated by filtration and washed with water to afford the title compound (4.24 g, 98%) as a pale tan colored amorphous solid. ^{1}H NMR (500 MHz, DMSO-d_6) δ 10.39 (s, 1H), 8.54 (d, *J* = 2.2 Hz, 1H), 7.99 (dd, *J* = 8.4, 2.3 Hz, 1H), 7.55 (d, *J* = 2.1 Hz, 1H), 7.52 (dd, *J* = 8.4, 2.2 Hz, 1H), 7.47 (dd, *J* = 8.4, 0.8 Hz, 1H), 7.01 (d, *J* = 8.4 Hz, 1H), 4.34 – 4.29 (m, 4H), 2.49 (s, 3H). HRMS (ESI$^+$): calcd for $C_{16}H_{15}N_2O_5$ $(M + H)^+$, 315.0976; found 315.0982.

Palladium (10% on activated carbon, 0.567 g, 5.33 mmol) was added to a suspension of *N*-(4-methyl-3-nitrophenyl)-2,3-dihydrobenzo[*b*][1,4]dioxine-6-carboxamide (4.24 g, 13.5 mmol) in ethanol (90 mL) and ethyl acetate (90 mL). The reaction mixture was stirred under hydrogen (1 atm) at 28 ˚C overnight, filtered through Celite® with EtOAc, and concentrated, to afford the title compound (3.80 g, 99%) as a pale yellow amorphous solid. ^{1}H NMR (500 MHz, DMSO-d_6) δ 9.70 (s, 1H), 7.49 (d, *J* = 2.2 Hz, 1H), 7.46 (dd, *J* = 8.3, 2.2 Hz, 1H), 7.10 (d, *J* = 2.0 Hz, 1H), 6.95 (d, *J* = 8.4 Hz, 1H), 6.83 (d, *J* = 8.1 Hz, 1H), 6.79 (dd, *J* = 8.1, 2.0 Hz, 1H), 4.81 (s, 2H), 4.32 – 4.26 (m, 4H), 2.01 (s, 3H). HRMS (ESI$^+$): calcd for $C_{16}H_{17}N_2O_3$ $(M + H)^+$, 285.1234; found 285.1233.

Methyl 2-formylquinoline-6-carboxylate **S2**

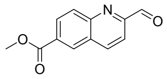

To a solution of 2-methylquinoline-6-carboxylic acid (3.00 g, 16.0 mmol) in anhydrous MeOH (40 mL) under argon at room temperature, 4M HCl in 1,4-dioxane (16.0 mL, 64.1 mmol) was added dropwise and the resulting mixture was heated at 85°C for 4 h. Then the reaction mixture was allowed to cool to room temperature, concentrated under reduced pressure, diluted with EtOAc (40 mL) and washed with NaOH 1M (2 x 40 mL), water (1 x 40 mL) and brine (1 x 40 mL). The organic layer was dried over $MgSO_4$, filtered and concentrated under reduced pressure to afford a light tan colored solid as crude product, which was carried onto the next step without purification (2.36 g, 73%). ^{1}H NMR (500 MHz, $CDCl_3$) δ 8.54 (d, *J* = 1.9 Hz, 1H), 8.27 (dd, *J* = 8.8, 1.9 Hz, 1H), 8.16 – 8.12 (m, 1H), 8.04 (dt, *J* = 8.8, 0.7 Hz, 1H), 7.35 (d, *J* = 8.4 Hz, 1H), 3.98 (s, 3H), 2.77 (s, 3H). HRMS (ESI$^+$): calcd for $C_{12}H_{12}NO_2$ $(M + H)^+$, 202.08676; found 202.08626.

To a suspension of selenium dioxide (0.873 g, 7.87 mmol) in anhydrous 1,4-dioxane (11 mL) under argon at room temperature, methyl-2-methylquinoline-6-carboxylate (1.44 g, 7.16 mmol) was added in one portion and the resulting suspension was allowed to stir at 80 ºC for 18 h. The reaction was allowed to cool to room temperature, filtered through Celite® and concentrated under vacuo to afford an orange solid as crude product, which was purified by column chromatography on silica gel using a gradient of 10-20% EtOAc in petroleum ether to afford the clean product as a pale yellow amorphous solid (1.28 g, 83%). ^{1}H-NMR (500 MHz, $CDCl_3$) δ 10.26 (d, *J* = 0.6 Hz, 1H), 8.68 (d, *J* = 1.6 Hz, 1H), 8.45 (d, *J* = 8.7 Hz, 1H), 8.42 (dd, *J* = 8.7, 1.6 Hz, 1H), 8.32 (d, *J* = 8.7 Hz, 1H), 8.11 (d, *J* = 8.2 Hz, 1H), 4.04 (s, 3H). HRMS (ESI$^+$): calcd for $C_{12}H_{10}NO_3$ $(M + H)^+$, 216.0660; found 216.0658.

N-(5-(2,3-dihydrobenzo[*b*][1,4]dioxine-6-carboxamido)-2-methylphenyl)-2-(pyrrolidin-1-ylmethyl)quinoline-6-carboxamide **11**

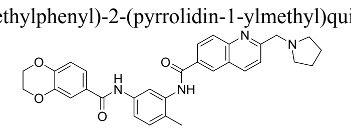

Pyrrolidine (0.144 mL, 1.74 mmol) was added to a suspension of methyl 2-formylquinoline-6-carboxylate (0.250 g, 1.16 mmol) in anhydrous DCM (5 mL). The reaction mixture was allowed to stir at room temperature for 6 h. Then sodium triacetoxyborohydride (0.369 g, 1.74 mmol) was added in one portion and the reaction mixture was stirred overnight at room temperature. The reaction mixture was diluted with DCM (5 mL), washed with $NaHCO_3$ saturated aqueous solution (1 x 10 mL). The two layers were separated and the aqueous phase was extracted with DCM (1 x 10 mL). The organic layer was dried over $MgSO_4$, filtered and concentrated under reduced pressure. The residue was purified by column chromatography using a gradient of 2-5% MeOH in DCM to afford the title compound as a light brown amorphous solid (225 mg, 71%). ^{1}H NMR (500 MHz, $CDCl_3$) δ 8.58 (d, *J* = 1.9 Hz, 1H), 8.32 – 8.24 (m, 2H), 8.10 (d, *J* = 8.8 Hz, 1H), 7.85 (br s, 1H), 4.14 (br s, 2H), 4.00 (s, 3H), 2.83 (br s, 4H), 1.94 (br s, 4H). HRMS (ESI$^+$): calcd for $C_{16}H_{19}N_2O_2$ $(M + H)^+$, 271.1441; found 271.14438.

Aqueous NaOH solution (1.02 M) (2.29 mL, 2.33 mmol) was added to a solution of methyl 2-(pyrrolidin-1-ylmethyl)quinoline-6-carboxylate (0.210 g, 0.777 mmol) in THF (3 mL), followed by MeOH (1 mL) to ensure a homogeneous solution. The reaction mixture was stirred at room temperature overnight. Then the reaction mixture was heated to 35 ºC and a further 1.25 mL of NaOH aqueous solution (1.02 M) was added and the reaction mixture was allowed to stir overnight. The reaction mixture was concentrated to remove THF and MeOH. The remaining aqueous layer was washed with EtOAc (1 x 5 mL), acidified to pH 3 with 2M aqueous HCl . A precipitate formed and was filtered off. The filtrate was then concentrated to dryness to afford the title compound as a brown solid which was carried onto the next step without purification (630 mg, contains NaCl, quantitative yield assumed for the next synthetic step). LCMS (ESI$^+$): t_R=1.42 min, m/z=257 $(M+H)^+$.

2-(7-aza-1*H*-benzotriazole-1-yl)-1,1,3,3-tetramethyluronium hexafluorophosphate (HATU) (0.323 g, 0.850 mmol) was added to a solution of 2-(pyrrolidin-1-ylmethyl)quinoline-6-carboxylic acid (0.199 g, 0.680 mmol) and *N,N*-diisopropylethylamine (0.594 mL, 3.40 mmol) in anhydrous DMF (4 mL). The reaction mixture was stirred for 5 minutes, before *N*-(3-amino-4-methylphenyl)-2,3-dihydrobenzo[*b*][1,4]dioxine-6-carboxamide (0.193 g, 0.680 mmol) was added. The reaction mixture was allowed to stir at room temperature overnight. Then a further portion of *N,N*-diisopropylethylamine (263 μL), and HATU (258 mg) was added and the resulting mixture was allowed to stir for 6 h. The reaction mixture was diluted with water (8 mL) and the resulting precipitate was isolated by filtration and washed with water. The residue was purified by column chromatography using a gradient of 5-12% MeOH in DCM to afford 65 mg of semi-crude product as an orange-brown solid. Re-purification by semi-preparative HPLC afforded the title compound as a pale yellow amorphous solid (27 mg, 6.7% over 2 steps). ^{1}H NMR (500 MHz, DMSO-d_6) δ 10.15 (s, 1H), 10.08 (s, 1H), 8.63 (d, *J* = 2.0 Hz, 1H), 8.48 (d, *J* = 8.5 Hz, 1H), 8.26 (dd, *J* = 8.8, 2.1 Hz, 1H), 8.16 (s, 1H), 8.08 (d, *J* = 8.8 Hz, 1H), 7.88 (d, *J* = 2.2 Hz, 1H), 7.72 (d, *J* = 8.5 Hz, 1H), 7.59 (dd, *J* = 8.2, 2.2 Hz, 1H), 7.54 (d, *J* = 2.1 Hz, 1H), 7.51 (dd, *J* = 8.5, 2.2 Hz, 1H), 7.25 (d, *J* = 8.4 Hz, 1H), 6.98 (d, *J* = 8.4 Hz, 1H), 4.34 – 4.27 (m, 4H), 3.94 (s, 2H), 2.61 – 2.52 (m, 4H), 2.25 (s, 3H), 1.77 – 1.73 (m, 4H) (formic acid salt). ^{13}C NMR (126 MHz, DMSO-d_6) δ 165.39, 164.82, 163.84, 162.24, 148.59, 146.81, 143.39, 137.96, 137.78, 136.69, 132.39, 130.60, 129.22, 129.16, 128.64, 128.45, 128.14, 126.67, 122.22, 121.66, 119.06, 118.65, 117.30, 117.12, 65.37, 64.86, 64.48, 62.29, 54.29, 23.74, 17.94. HRMS (ESI$^+$): calcd for $C_{31}H_{31}N_4O_4$ $(M + H)^+$, 523.23398; found 523.23419.

N-(5-(2,3-Dihydrobenzo[*b*][1,4]dioxine-6-carboxamido)-2-methylphenyl)-2-((2-methylpyrrolidin-1-yl)methyl)quinoline-6-carboxamide **13**

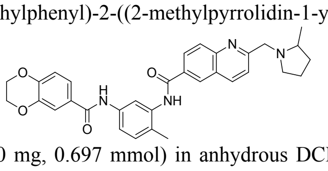

To a solution of methyl 2-formylquinoline-6-carboxylate (150 mg, 0.697 mmol) in anhydrous DCM (7 mL), 2-methylpyrrolidine (0.213 mL, 2.09 mmol) was added dropwise at room temperature and the resulting mixture was stirred for 2.5 h. Then sodium triacetoxyborohydride (443 mg, 2.09 mmol) was added in one portion and the resulting mixture was stirred overnight at room temperature. The reaction mixture was diluted with DCM (10 mL) and washed with $NaHCO_3$ saturated aqueous solution (20 mL). The aqueous phase was extracted with DCM (3 x 10 mL) and the combined organic layers were dried over Na_2SO_4, filtered and concentrated under reduced pressure to afford a yellow oil as crude product, which was carried onto the next step without purification (194 mg). ^{1}H NMR (500 MHz, $CDCl_3$) δ 8.51 (d, *J* = 2.0 Hz, 1H), 8.22 (dd, *J* = 8.8, 2.0 Hz, 1H), 8.19 – 8.12 (m, 1H), 8.05 (dt, *J* = 8.9, 0.8 Hz, 1H), 7.67 (d, *J* = 8.5 Hz, 1H), 4.23 (d, *J* =

14.1 Hz, 1H), 3.94 (s, 3H), 3.57 (d, J = 14.1 Hz, 1H), 2.99 – 2.91 (m, 1H), 2.61 – 2.50 (m, 1H), 2.27 (q, J = 8.9 Hz, 1H), 2.07 – 1.86 (m, 1H), 1.79 – 1.57 (m, 2H), 1.45 (dddd, J = 12.5, 10.7, 8.5, 6.1 Hz, 1H), 1.13 (d, J = 6.0 Hz, 3H). LCMS (ESI$^+$): t_R=0.88 min, m/z=285, (M+H)$^+$.

To a solution of methyl 2-((2-methylpyrrolidin-1-yl)methyl)quinoline-6-carboxylate (194 mg, 0.682 mmol) in anhydrous THF (3.2 mL), 2 M aqueous NaOH solution (1.70 mL, 3.41 mmol) was added dropwise and MeOH (1.3 mL) was added to increase the miscibility of the two layers. The resulting red/brown solution was allowed to stir at 20 °C for 3 h. The reaction mixture was concentrated under reduced pressure and the remaining aqueous layer was acidified to pH 3 with 1 M aqueous HCl and then washed with EtOAc (1 x 5 mL). The organic phase was discarded and the aqueous phase was concentrated under reduced pressure to afford a beige amorphous solid as crude product, which was carried onto the next step without purification. HRMS (ESI$^+$): calcd for $C_{16}H_{19}N_2O_2$ (M + H)$^+$, 272.1472; found 272.1468.

To a solution of 2-(7-aza-1*H*-benzotriazole-1-yl)-1,1,3,3-tetramethyluronium hexafluorophosphate (HATU) (176 mg, 0.462 mmol), 2-((2-methylpyrrolidin-1-yl)methyl)quinoline-6-carboxylic acid hydrochloride (100 mg, 0.326 mmol) in anhydrous DMF (2.5 mL) with *N,N*-diisopropylethylamine (0.322 mL, 1.85 mmol), *N*-(3-amino-4-methylphenyl)-2,3-dihydrobenzo[*b*][1,4]dioxine-6-carboxamide (105 mg, 0.370 mmol) was added in one portion and the resulting mixture was allowed to stir at 20 °C under an inert argon atmosphere for 18 h. The reaction mixture was poured onto water to afford a light brown precipitate, which was washed with water. The crude product was purified by column chromatography using a gradient of 0-20% EtOAc in DCM. A second purification by column chromatography using a gradient of 0-10% MeOH in DCM + 1% NH_3 in MeOH afforded the title compound as a light brown amorphous solid (24 mg, ~12%). ^{1}H NMR (500 MHz, MeOD) δ 8.69 – 8.55 (m, 1H), 8.49 (d, J = 8.5 Hz, 1H), 8.31 (dd, J = 8.8, 1.8 Hz, 1H), 8.17 (d, J = 8.8 Hz, 1H), 7.87 – 7.70 (m, 2H), 7.59 – 7.42 (m, 3H), 7.31 (d, J = 8.4 Hz, 1H), 6.95 (d, J = 8.4 Hz, 1H), 4.43 (d, J = 15.1 Hz, 1H), 4.34 – 4.24 (m, 4H), 3.75 (s, 1H), 3.11 (s, 1H), 2.77 (s, 1H), 2.51 (s, 1H), 2.34 (s, 3H), 2.12 (s, 1H), 1.83 (s, 2H), 1.64 – 1.49 (m, 1H), 1.27 (d, J = 5.9 Hz, 3H). ^{13}C NMR (126 MHz, DMSO-d_6) δ 165.39, 164.83, 148.55, 146.81, 143.39, 137.88, 137.79, 136.69, 132.43, 130.61, 129.22, 129.15, 128.65, 128.50, 128.14, 126.68, 122.41, 121.67, 119.07, 118.66, 117.31, 117.14, 64.86, 64.49, 60.40, 60.30, 60.08, 54.50, 32.72, 21.94, 19.24, 17.95. HRMS (ESI$^+$): calcd for $C_{32}H_{32}N_4NaO_4$ (M + Na)$^+$, 559.2316; found 559.2308.

N-(5-(2,3-Dihydrobenzo[*b*][1,4]dioxine-6-carboxamido)-2-methylphenyl)-2-((4-ethylpiperazin-1-yl)methyl)quinoline-6-carboxamide **S3**

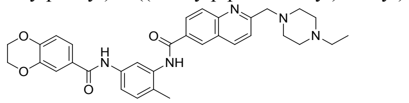

To a solution of methyl 2-formylquinoline-6-carboxylate (150 mg, 0.697 mmol) in anhydrous DCM, 1-ethylpiperazine (0.266 mL, 2.09 mmol) was added dropwise at room temperature and the resulting mixture was allowed to stir under an inert argon atmosphere for 2.5 h. Then sodium triacetoxyborohydride (443 mg, 2.09 mmol) was added in one portion and the resulting mixture was allowed to stir overnight at room temperature. The reaction mixture was diluted with DCM (20 mL) and quenched with $NaHCO_3$ saturated aqueous solution (20 mL). The aqueous phase was extracted with DCM (3 x 10 mL) and the combined organic layers were dried over Na_2SO_4 and concentrated under reduced pressure to afford a yellow amorphous solid as crude product, which was carried onto the next step without purification (197 mg). ^{1}H NMR (500 MHz, $CDCl_3$) δ 8.55 (d, J = 1.9 Hz, 1H), 8.31 – 8.15 (m, 2H), 8.08 (dt, J = 8.9, 0.7 Hz, 1H), 7.69 (d, J = 8.5 Hz, 1H), 3.97 (s, 3H), 3.85 (s, 2H), 2.57 (d, J = 47.4 Hz, 8H), 2.42 (q, J = 7.2 Hz, 2H), 1.08 (t, J = 7.2 Hz, 3H). ^{13}C NMR (126 MHz, $CDCl_3$) δ 166.67, 162.22, 149.53, 137.47, 130.66, 129.33, 128.90, 127.62, 126.49, 121.86, 65.10, 53.37, 52.76, 52.36, 52.29, 11.94.

To a solution of methyl 2-((4-ethylpiperazin-1-yl)methyl)quinoline-6-carboxylate (197 mg, 0.629 mmol) in THF (3.0 mL), 2 M aqueous NaOH (1.57 mL, 3.14 mmol) was added dropwise at 20 °C and MeOH (1.2 mL) was added to increase the miscibility of the two layers. The resulting red/brown solution was allowed to stir at 20°C for 2 h. The reaction mixture was concentrated under reduced pressure in order to remove THF and MeOH, then the aqueous layer was acidified to pH 3 with 1 M aqueous HCl and washed with EtOAc (3 x 5mL). The aqueous layer was concentrated under vacuo to afford a salmon solid as crude product, which was carried onto the next step without purification . ^{1}H NMR (500 MHz, DMSO-d_6) δ 13.37 (br s, 1H), 8.80 – 8.62 (m, 2H), 8.27 (dd, J = 8.9, 2.0 Hz, 1H), 8.16 (d, J = 8.8 Hz, 1H), 7.84 (d, J = 8.4 Hz, 1H), 4.56 (s, 2H), 3.62 (br s, 8H), 3.18 (br s, 2H), 1.26 (t, J = 7.3 Hz, 3H). HRMS (ESI$^+$): calcd for $C_{17}H_{22}N_3O_2$ (M + H)$^+$, 302.1764; found 302.1762.

To a solution of 2-(7-Aza-1*H*-benzotriazole-1-yl)-1,1,3,3-tetramethyluronium hexafluorophosphate (HATU) (159 mg, 0.418 mmol) and 2-((4-ethylpiperazin-1-yl)methyl)quinoline-6-carboxylic acid hydrochloride salt (100 mg, 0.298 mmol) in anhydrous DMF (2.3 mL) with *N,N*-diisopropylethylamine (0.291 mL, 1.67 mmol), *N*-(3-amino-4-methylphenyl)-2,3-dihydrobenzo[*b*][1,4]dioxine-6-carboxamide (95.0 mg, 0.334 mmol) was added in one portion and the resulting mixture was allowed to stir at 20°C for 18 h. The reaction mixture was poured onto water (3 mL) to afford a pale yellow precipitate which was washed with water (3 x 5 mL). Then the solid was purified by flash column chromatography eluting with 20% EtOAc in DCM and then a gradient of 0-10% MeOH in DCM + 1% 7N NH_3 in MeOH to afford the title compound as a pale yellow amorphous solid (48 mg, 25%). ^{1}H NMR (500 MHz, MeOD) δ 8.58 (d, J = 2.0 Hz, 1H), 8.45 (d, J = 8.5 Hz, 1H), 8.29 (dd, J = 8.9, 2.1 Hz, 1H), 8.13 (d, J = 8.8 Hz, 1H), 7.85 – 7.73 (m, 2H), 7.59 – 7.39 (m, 3H), 7.29 (dd, J = 8.3, 0.9 Hz, 1H), 6.93 (d, J = 8.4 Hz, 1H), 4.40 – 4.17 (m, 4H), 3.89 (s, 2H), 2.93 – 2.37 (m, 10H), 2.33 (s, 3H), 1.11 (t, J = 7.2 Hz, 3H). ^{13}C NMR (126 MHz, MeOD) δ 166.87, 166.54, 161.28, 148.33, 147.01, 143.45, 138.07, 136.96, 135.72, 132.24, 130.35, 130.19, 128.13, 128.01, 127.92, 127.59, 126.83, 122.13, 120.71, 119.45, 119.21, 116.76, 116.61, 64.52, 64.13, 64.01, 52.60, 52.20, 51.87, 48.44, 16.39, 10.37. HRMS (ESI$^+$): calcd for $C_{33}H_{36}N_5O_4$ (M + H)$^+$, 567.2793; found 567.2789.

N-(3-Amino-4-fluorophenyl)-2,3-dihydrobenzo[*b*][1,4]dioxine-6-carboxamide **S4**

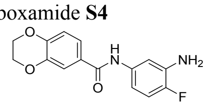

Oxalyl chloride (18.5 mL, 211 mmol) was added to a stirred solution of 1,4-benzodioxane-6-carboxylic acid (34.6 g, 192 mmol) and pyridine (31.1 mL, 384 mmol) in anhydrous DCM (400 mL) at 0 ºC. After 1 h, the reaction mixture was concentrated in vacuo. The remaining residue was re-dissolved in anhydrous DCM (200 mL) and concentrated in vacuo. The remaining residue was re-dissolved in DCM (40 mL) and added to a stirred solution of 4-fluoro-3-nitroaniline (30 g, 192 mmol) and pyridine (31.1 mL, 384 mmol) in DCM (400 mL) at 0ºC. After stirring for 16 h, the reaction mixture was concentrated in vacuo and diluted with MeOH (400 mL) and water (400 mL). A precipitate formed and was isolated by filtration and washed with water. The solid was dried under vacuum to afford the desired product as a yellow amorphous solid (52.2 g, 85 %). ^{1}H NMR (500 MHz, DMSO-d_6) δ 10.47 (s, 1H), 8.69 (dd, J = 6.9, 2.8 Hz, 1H), 8.13 (ddd, J = 9.1, 4.0, 2.8 Hz, 1H), 7.66 – 7.44 (m, 3H), 7.01 (d, J = 8.4 Hz, 1H), 4.32 (td, J = 5.3, 3.6 Hz, 4H). ^{13}C NMR (126 MHz, DMSO-d_6) δ 165.30, 150.98 (d, J = 252.69 Hz), 147.28, 143.49, 136.69 (d, J = 7.65 Hz), 136.57 (d, J = 2.77 Hz), 127.95 (d, J = 8.74 Hz), 127.19, 121.83, 119.10 (d, J = 22.18 Hz), 117.50, 117.21, 117.03 (d, J = 2.17 Hz), 64.91, 64.49. HRMS (ESI+): calcd for $C_{15}H_{12}FN_2O_5$ (M + H)$^+$ 319.0725; found 319.0729.

Ammonium chloride (10.3g, 192 mmol) and iron (10.7 g, 192 mmol) were added to a mixture of *N*-(4-fluoro-3-nitrophenyl)-2,3-dihydrobenzo[*b*][1, 4]dioxine-6-carboxamide (12.228 g, 38.4 mmol) in ethanol (120 mL) and water (40 mL). The reaction was refluxed at 90 °C for 1 h. The reaction was cooled to room temperature and diluted with DCM (30 mL) and MeOH (30 mL). The resulting mixture was filtered through Celite® and washed through with MeOH. The filtrate was concentrated under reduced pressure. The crude solid was diluted in aqueous saturated $NaHCO_3$ solution (150 mL) to make a slurry which was filtered. The solid was collected, washed with water and then diluted with toluene and dried in vacuo to afford the crude product as a beige amorphous solid, used as crude in the next synthetic step (6.25 g). ^{1}H NMR (500 MHz, DMSO-d_6) δ 9.81 (s, 1H), 7.55 – 7.41 (m, 2H), 7.28 (d, J = 7.8 Hz, 1H), 7.02 – 6.78 (m, 3H), 5.14 (s, 2H), 4.30 (d, J = 5.6 Hz, 4H). ^{13}C NMR (126 MHz, DMSO-d_6) δ 164.63, 147.57 (d, J = 227.57 Hz), 146.62, 143.34, 136.56 (d, J = 14.52 Hz), 136.06 (d, J = 2.88 Hz), 128.37, 121.59, 117.24, 117.08, 114.86 (d, J = 21.65 Hz), 109.02 (d, J = 2.54 Hz), 108.65 (d, J = 5.94 Hz), 64.84, 64.48. HRMS (ESI+): calcd for $C_{15}H_{14}FN_2O_3$ (M + H)$^+$ 289.0988; found 289.0992.

2-((4-isopropylpiperazin-1-yl)methyl)quinoline-6carboxylic acid **S5**

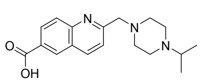

Pyrrolidine (0.399 mL, 2.79 mmol) was added to a suspension of methyl 2-formylquinoline-6-carboxylate (0.200 g, 0.929 mmol) in anhydrous DCM (9 mL). The reaction mixture was stirred at room temperature for 2.5 h. Then sodium triacetoxyborohydride (0.591 g, 2.79 mmol) was added in one portion and the reaction mixture was stirred overnight at room temperature. The reaction mixture was diluted with DCM (20 mL), washed with $NaHCO_3$ saturated aqueous solution (1 x 20

mL). The two layers were separated and the aqueous layer was extracted with DCM (3 x 10 mL). The combined organic layers were dried over $MgSO_4$ and concentrated to afford the crude product as an amorphous orange solid (322 mg). ^{1}H NMR (500 MHz, $CDCl_3$) δ 8.54 (d, *J* = 1.9 Hz, 1H), 8.25 (dd, *J* = 8.8, 1.9 Hz, 1H), 8.18 (dd, *J* = 8.6, 0.7 Hz, 1H), 8.07 (d, *J* = 8.7 Hz, 1H), 7.69 (d, *J* = 8.5 Hz, 1H), 3.96 (s, 3H), 3.84 (s, 2H), 2.67 – 2.52 (m, 9H), 1.04 (s, 3H), 1.03 (s, 3H). ^{13}C NMR (126 MHz, $CDCl_3$) δ 166.81, 162.43, 149.68, 137.55, 130.78, 129.46, 129.00, 127.71, 126.60, 122.00, 77.42, 77.16, 76.91, 65.25, 54.56, 53.88, 52.48, 48.78, 18.77. HRMS (ESI+): calcd for $C_{19}H_{26}N_3O_2$ $(M + H)^+$ 328.202; found 328.2031.

To a solution of methyl 2-((4-isopropylpiperazin-1-yl)methyl)quinoline-6-carboxylate (320 mg, 0.977 mmol) in THF (6.0 mL), 2 M aqueous NaOH (2.44 mL, 4.89 mmol) was added dropwise at 20 °C and MeOH (2.4 mL) was added to increase the miscibility of the two layers. The resulting red/brown solution was allowed to stir at 20°C for 2 h. The reaction mixture was concentrated under reduced pressure in order to remove THF and MeOH, then the aqueous layer was acidified to pH 3 with 1 M aqueous HCl and washed with EtOAc (3 x 5mL). The aqueous layer was concentrated under vacuo to afford a salmon solid as crude product, which was carried onto the next step without purification (306 mg, contains NaCl). ^{1}H NMR (500 MHz, DMSO-d_6) δ 8.73 (d, *J* = 1.9 Hz, 1H), 8.71 (d, *J* = 8.6 Hz, 1H), 8.27 (dd, *J* = 8.7, 1.9 Hz, 1H), 8.18 (d, *J* = 8.8 Hz, 1H), 7.89 (dd, *J* = 8.5, 1.8 Hz, 1H), 3.74 – 3.41 (m, 11H), 1.30 (m, 6H). LCMS (ESI$^+$): t_R=0.70 min, m/z=314, $(M+H)^+$.

N-(5-(2,3-Dihydrobenzo[*b*][1,4]dioxine-6-carboxamido)-2-fluorophenyl)-2-((4-isopropylpiperazin-1-yl)methyl)quinoline-6-carboxamide **S6**

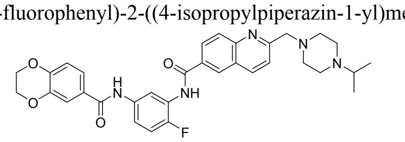

N-(3-Amino-4-fluorophenyl)-2,3-dihydrobenzo[*b*][1,4]dioxine-6-carboxamide (100 mg, 0.347 mmol), 2-((4-isopropylpiperazin-1-yl)methyl)quinoline-6-carboxylic acid hydrochloride (130 mg, 0.372 mmol) and EDC (166 mg, 0.867 mmol) were dissolved in anhydrous DMF (2.5 mL), then pyridine (0.140 mL, 1.73 mmol) was added dropwise and the resulting mixture was allowed to stir at 20°C for 20 h. A further portion of 2-((4-isopropylpiperazin-1-yl)methyl)quinoline-6-carboxylic acid (130 mg, 0.416 mmol), EDC (166 mg, 0.867 mmol) and pyridine (0.140 mL, 1.73 mmol) was added and the resulting mixture was allowed to stir for a total of 72 h at 20 °C. The reaction was quenched with water (5 mL) and extracted with DCM/MeOH 9/1 (3 x 5 mL). Purification by column chromatography using a gradient of 0-10% MeOH in DCM + 1% 7N NH_3 in MeOH followed by trituration in diethyl ether afforded the title compound as an orange amorphous solid (50 mg, 9.2% over 3 steps). ^{1}H-NMR (500 MHz, DMSO-d_6) δ 10.41 (br s, 1H), 10.21 (br s, 1H), 8.65 (s, 1H), 8.49 (d, *J* = 8.78 Hz, 1H), 8.25 (dd, *J* = 8.78, 1.88 Hz, 1H), 8.14 (dd, *J* = 6.90, 2.51 Hz, 1H), 8.08 (d, *J* = 8.78 Hz, 1H), 7.73 (d, *J* = 8.78 Hz, 1H), 7.68 – 7.63 (m, 1H), 7.55 (d, *J* = 2.51 Hz, 1H), 7.52 (dd, *J* = 8.78, 1.88 Hz, 1H), 7.29 (app t, *J* = 9.28 Hz, 1H), 6.99 (d, *J* = 8.78 Hz, 1H), 4.36 – 4.27 (m, 4H), 3.79 (br s, 2H), 3.15 – 2.34 (m, 9H), 0.99 (br s, 6H). ^{13}C NMR (126 MHz, MeOD) δ 166.69, 166.62, 161.22, 152.52 (d, *J* = 245.41 Hz), 151.49, 148.39, 147.12, 143.49, 138.19, 134.88 (d, *J* = 3.22 Hz), 131.99, 128.21, 127.99, 127.37, 126.82, 125.27 (d, *J* = 11.96 Hz), 122.18, 120.76, 119.62 (d, *J* = 7.97 Hz), 119.01, 116.80, 116.64, 115.28 (d, *J* = 21.26 Hz), 64.54, 64.15, 63.80, 55.27, 52.30, 29.34, 16.98. HRMS (ESI$^+$): calcd for $C_{33}H_{35}FN_5O_4$ $(M + H)^+$, 584.2668; found 584.2636.

Synthetic Route II

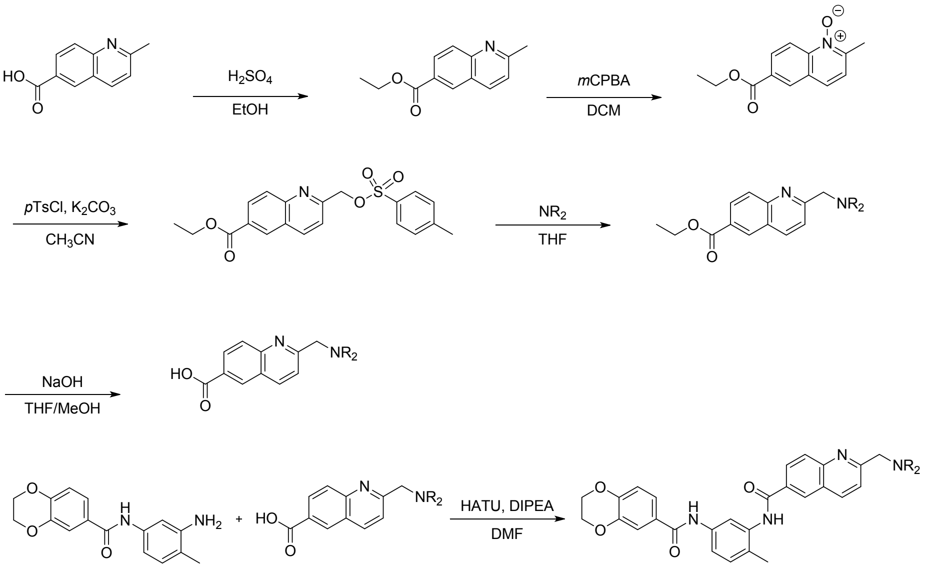

Ethyl 2-((tosyloxy)methyl)quinoline-6-carboxylate **S7**

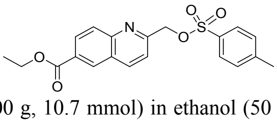

To a stirred solution of 2-methylquinoline-6-carboxylic acid (2.00 g, 10.7 mmol) in ethanol (50 mL) was added sulfuric acid (0.4 mL, 10.7 mmol). The reaction was heated to 80 °C under argon for 22 h. The solvent was removed in vacuo. The resulting residue was the taken up in water (100 mL). The solution was basified (~ pH 10) by the addition of 2M aqueous NaOH solution. The resulting precipitate was collected by filtration and washed with copious water, then dried under vacuum to afford a pale pink amorphous solid (1.57 g, 68%). ^{1}H NMR (500 MHz, $CDCl_3$) δ 8.56 (d, *J* = 1.9 Hz, 1H), 8.29 (dd, *J* = 8.8, 1.9 Hz, 1H), 8.19 – 8.13 (m, 1H), 8.05 (dt, *J* = 8.8, 0.7 Hz, 1H), 7.36 (d, *J* = 8.4 Hz, 1H), 4.46 (q, *J* = 7.1 Hz, 2H), 2.79 (s, 3H), 1.46 (t, *J* = 7.1 Hz, 3H). LCMS (ESI$^+$): t_R=2.24 min, m/z 216 $(M + H)^+$.

3-Chloroperbenzoic acid (0.695 g, 3.02 mmol) was added to a solution of ethyl 2-methylquinoline-6-carboxylate (0.5 g, 2.32 mmol) in anhydrous DCM (7 mL) at 0 ºC. The reaction mixture was then allowed to warm to room temperature and stirred overnight. The orange reaction mixture was washed with 10% aqueous Na_2SO_3 solution (1 x 10 mL) and saturated aqueous $NaHCO_3$ solution (1 x 10 mL). The two layers were separated and the aqueous layer was diluted with brine and extracted with DCM (3 x 10 mL). The combined organic phases were dried over $MgSO_4$, filtered and concentrated in vacuo. The crude orange oil was crystallized from EtOAc/PE. The solid was isolated by filtration and washed with PE/EtOAc (3/1 mixture). A second product fraction was isolated after concentration of the filtrate. This solid was triturated with PE/EtOAc (~4/1) and isolated by filtration. The title compound was obtained as a pale orange amorphous solid (381 mg, 71%). ^{1}H NMR (500 MHz, $CDCl_3$) δ 8.82 (d, *J* = 9.1 Hz, 1H), 8.58 (d, *J* = 1.7 Hz, 1H), 8.33 (dd, *J* = 9.1, 1.8 Hz, 1H), 7.74 (d, *J* = 8.5 Hz, 1H), 7.39 (d, *J* = 8.5 Hz, 1H), 4.46 (q, *J* = 7.1 Hz, 2H), 2.74 (s, 3H), 1.45 (t, *J* = 7.1 Hz, 3H). LCMS (ESI$^+$): t_R= 2.32 min, m/z 232.10 $(M + H)^+$.

To a solution of ethyl 2-methylquinoline-6-carboxylate *N*-oxide (0.274 g, 1.19 mmol) in anhydrous acetonitrile (10 mL) at 0 °C, K_2CO_3 (0.246 g, 1.78 mmol) was added in one portion, followed by *p*-toluenesulfonyl chloride (0.271 g, 1.42 mmol). The reaction mixture was stirred at 0 °C for 4 h. The reaction mixture was diluted with saturated aqueous $NaHCO_3$ solution and extracted with EtOAc (2 x 10 mL). The organic layer was washed with water (1 x 10 mL), brine (1 x 10 mL),

dried over $MgSO_4$, filtered and concentrated in vacuo. The crude (dark blue-green solid) was purified by column chromatography using a gradient of 16-40% EtOAc in petroleum ether to afford an orange amorphous solid (186 mg, 41%). ^{1}H NMR (500 MHz, $CDCl_3$) δ 8.58 (d, J = 1.9 Hz, 1H), 8.34 – 8.25 (m, 2H), 8.01 (d, J = 8.8 Hz, 1H), 7.86 (d, J = 8.4 Hz, 2H), 7.63 (d, J = 8.5 Hz, 1H), 7.33 (d, J = 8.1 Hz, 2H), 5.32 (s, 2H), 4.45 (q, J = 7.1 Hz, 2H), 2.42 (s, 3H), 1.45 (t, J = 7.1 Hz, 3H). LCMS (ESI$^+$): t_R= 3.11 min, m/z 386.22 (M + H)$^+$.

N-(5-(2,3-Dihydrobenzo[*b*][1,4]dioxine-6-carboxamido)-2-methylphenyl)-2-((dimethylamino)methyl)quinoline-6-carboxamide **12**

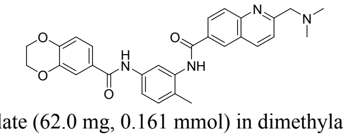

A solution of ethyl 2-((tosyloxy)methyl)quinoline-6-carboxylate (62.0 mg, 0.161 mmol) in dimethylamine (2M in THF) (0.080 mL, 0.161 mmol) was heated under microwave irradiation at 60 °C for 1 h. The reaction mixture was concentrated under reduced pressure, diluted with EtOAc, washed with water (1 x 1 mL) and saturated aqueous $NaHCO_3$ solution (1 x 1 mL). The aqueous phase was extracted with EtOAc (1 x 1 mL). The organic layers were combined, dried over Na_2SO_4, filtered and concentrated under reduced pressure to afford an orange oil (42 mg). ^{1}H NMR (500 MHz, $CDCl_3$) δ 8.57 (d, J = 1.8 Hz, 1H), 8.29 (dd, J = 8.8, 1.9 Hz, 1H), 8.23 (d, J = 8.5 Hz, 1H), 8.11 (d, J = 8.8 Hz, 1H), 7.67 (d, J = 8.5 Hz, 1H), 4.45 (q, J = 7.2 Hz, 2H), 3.79 (s, 2H), 2.35 (s, 6H), 1.45 (t, J = 7.1 Hz, 3H).
Aqueous NaOH solution (1.04 M, 0.304 mL, 0.317 mmol) was added to a solution of ethyl 2-((dimethylamino)methyl)quinoline-6-carboxylate (41.0 mg, 0.159 mmol) in THF (1 mL) and MeOH (0.3 mL). The reaction mixture was stirred at room temperature overnight. A further portion of water (0.5 mL) and aqueous NaOH (1.15 M, 0.276 mL, 0.317 mmol) was added and the resulting mixture was allowed to stir overnight. The reaction mixture was concentrated to remove the organic solvents, diluted with water, washed with EtOAc (1 x 1 mL). The aqueous phase was acidified to ~ pH 3 with aqueous HCl solution (2M) and then concentrated to afford the title compound as a crude pale yellow solid, which was carried onto the next step without purification. ^{1}H NMR (500 MHz, DMSO-d_6) δ 12.11 (br s, 1H), 8.70 (d, J = 1.8 Hz, 1H), 8.65 (d, J = 8.5 Hz, 1H), 8.25 (dd, J = 8.8, 1.9 Hz, 1H), 8.10 (d, J = 8.8 Hz, 1H), 7.84 (d, J = 8.5 Hz, 1H), 4.42 (br s, 2H), 2.70 (s, 6H). LCMS (ESI$^+$): t_R=0.82 min, m/z 231.11 (M + H)$^+$.
HATU (72.0 mg, 0.189 mmol) was added to a solution of 2-((dimethylamino)methyl)quinoline-6-carboxylic acid (0.106 g, 0.151 mmol) and *N,N*-diisopropylethylamine (0.111 mL, 0.634 mmol) in anhydrous DMF (1.5 mL). The reaction mixture stirred for 5 minutes, before *N*-(3-amino-4-methylphenyl)-2,3-dihydrobenzo[*b*][1,4]dioxine-6-carboxamide (32.0 mg, 0.113 mmol) was added. The reaction mixture was stirred at room temperature overnight. The reaction mixture was diluted with water and the resulting precipitate isolated by filtration and washed with water. The residue was purified by column using a gradient of 4-10% MeOH in DCM to afford the title compound as an off-white amorphous solid (24 mg, 30% over 3 steps). ^{1}H NMR (500 MHz, DMSO-d_6) δ 10.15 (s, 1H), 10.07 (s, 1H), 8.63 (d, J = 1.9 Hz, 1H), 8.48 (d, J = 8.5 Hz, 1H), 8.26 (dd, J = 8.8, 2.0 Hz, 1H), 8.08 (d, J = 8.8 Hz, 1H), 7.88 (d, J = 2.1 Hz, 1H), 7.71 (d, J = 8.5 Hz, 1H), 7.62 – 7.48 (m, 3H), 7.24 (d, J = 8.5 Hz, 1H), 6.98 (d, J = 8.4 Hz, 1H), 4.30 (td, J = 5.1, 3.6 Hz, 4H), 3.74 (s, 2H), 2.25 (d, J = 6.0 Hz, 9H). ^{13}C NMR (126 MHz, DMSO-d_6) δ 165.39, 164.82, 162.29, 148.58, 146.81, 143.39, 137.90, 137.78, 136.69, 132.42, 130.60, 129.22, 129.18, 128.63, 128.42, 128.14, 126.70, 122.25, 121.65, 119.06, 118.65, 117.30, 117.12, 66.13, 64.86, 64.48, 45.88, 17.93. HRMS (ESI$^+$): calcd for $C_{29}H_{29}N_4O_4$ (M + H)$^+$, 497.21833; found 497.21829.

N-(5-(2,3-Dihydrobenzo[*b*][1,4]dioxine-6-carboxamido)-2-methylphenyl)-2-((4-methylpiperazin-1-yl)methyl)quinoline-6-carboxamide **15**

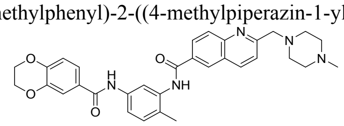

1-Methylpiperazine (0.058 mL, 0.519 mmol) was added to a solution of ethyl 2-((tosyloxy)methyl)quinoline-6-carboxylate (80.0 mg, 0.208 mmol) in anhydrous THF (1.5 mL). The reaction mixture was heated to reflux for 1.5 h. The reaction mixture was cooled to room temperature, stirred for 2 h and concentrated under reduced pressure. The residue was diluted with EtOAc (2 mL) and washed with water (1 x 2 mL) and saturated aqueous $NaHCO_3$ solution (1 x 2 mL). The aqueous phase was extracted with EtOAc (1 x 2 mL). The combined organic phases were dried over $MgSO_4$, filtered and concentrated in vacuo to afford the title compound as a yellow amorphous solid, which was carried onto the next step without purification (64 mg). ^{1}H NMR (500 MHz, $CDCl_3$) δ 8.56 (d, J = 1.9 Hz, 1H), 8.29 (dd, J = 8.8, 1.9 Hz, 1H), 8.22 (d, J = 8.5 Hz, 1H), 8.09 (d, J = 8.8 Hz, 1H), 7.71 (d, J = 8.5 Hz, 1H), 4.45 (q, J = 7.1 Hz, 2H), 3.86 (s, 2H), 2.61 (br s, 4H), 2.49 (br s, 4H), 2.31 (s, 3H), 1.45 (t, J = 7.1 Hz, 3H). HRMS (ESI$^+$): calcd for $C_{18}H_{24}N_3O_2$ (M + H)$^+$, 314.1863; found 314.18711.
Aqueous NaOH solution (0.82 M, 0.735 mL, 0.603 mmol) was added to a solution of ethyl 2-((4-methylpiperazin-1-yl)methyl)quinoline-6-carboxylate (63.0 mg, 0.201 mmol) in THF (1 mL) and MeOH (0.25 mL), and the reaction mixture was stirred at room temperature overnight. The reaction mixture was concentrated to remove the organic solvents, diluted with water, washed with EtOAc (1 x 1 mL). The aqueous phase was acidified with aqueous HCl solution (2M) to pH 2-3, then concentrated to dryness. The crude product (light brown amorphous solid) was carried onto the next step without further purification (114 mg). ^{1}H NMR (500 MHz, DMSO-d_6) δ 13.33 (br s, 1H), 11.77 (br s, 1H), 8.72 (d, J = 1.9 Hz, 1H), 8.69 (d, J = 8.5 Hz, 1H), 8.26 (dd, J = 8.8, 1.9 Hz, 1H), 8.16 (d, J = 8.9 Hz, 1H), 7.85 (d, J = 8.5 Hz, 1H), 4.53 (br s, 2H), 3.83 – 3.45 (br m, 8H), 2.80 (s, 3H) (hydrochloric acid salt). HRMS (ESI$^+$): calcd for $C_{16}H_{20}N_3O_2$ (M + H)$^+$, 286.155; found 286.15533.
HATU (68.0 mg, 0.178 mmol) was added to a suspension of 2-((4-methylpiperazin-1-yl)methyl)quinoline-6-carboxylic acid (81.0 mg, 0.142 mmol) and *N,N*-diisopropylethylamine (0.131 mL, 0.748 mmol) in anhydrous DMF (1 mL). The reaction mixture was stirred for 4 minutes before *N*-(3-amino-4-methylphenyl)-2,3-dihydrobenzo[*b*][1,4]dioxine-6-carboxamide (34.0 mg, 0.121 mmol) was added. The reaction mixture was stirred at room temperature overnight. The reaction mixture was diluted with water and the resulting precipitate isolated by filtration and washed with water. The residue was purified by column chromatography using a gradient of 5-18% MeOH in DCM to afford the title compound as an off-white amorphous solid (36 mg, 31% over 3 steps). ^{1}H NMR (500 MHz, DMSO-d_6) δ 10.15 (s, 1H), 10.08 (s, 1H), 8.63 (d, J = 1.9 Hz, 1H), 8.48 (d, J = 8.4 Hz, 1H), 8.26 (dd, J = 8.8, 2.0 Hz, 1H), 8.08 (d, J = 8.8 Hz, 1H), 7.88 (d, J = 2.1 Hz, 1H), 7.72 (d, J = 8.5 Hz, 1H), 7.59 (dd, J = 8.3, 2.2 Hz, 1H), 7.56 – 7.48 (m, 2H), 7.25 (d, J = 8.5 Hz, 1H), 6.98 (d, J = 8.4 Hz, 1H), 4.31 (q, J = 5.1 Hz, 4H), 3.79 (s, 2H), 2.50 – 2.32 (m, 8H), 2.24 (s, 3H), 2.17 (s, 3H). ^{13}C NMR (126 MHz, DMSO-d_6) δ 165.37, 164.82, 161.97, 148.63, 146.81, 143.39, 137.93, 137.78, 136.69, 132.39, 130.61, 129.23, 129.16, 128.64, 128.42, 128.14, 126.69, 122.22, 121.66, 119.07, 118.65, 117.30, 117.12, 64.86, 64.49, 55.18, 53.35, 46.19, 40.89, 17.93. HRMS (ESI$^+$): calcd for $C_{32}H_{34}N_5O_4$ (M + H)$^+$, 552.26053; found 552.25911.

Synthetic route III

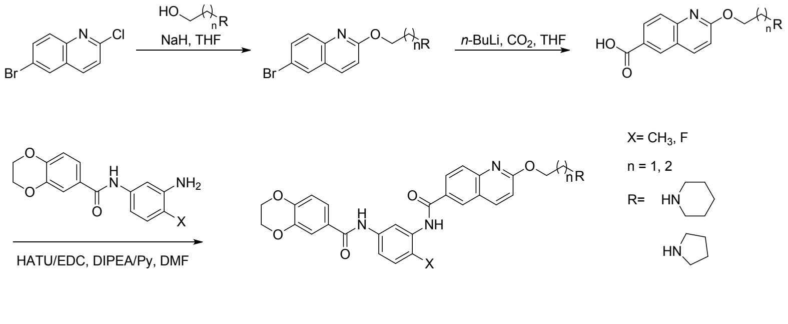

N-(5-(2,3-Dihydrobenzo[*b*][1,4]dioxine-6-carboxamido)-2-methylphenyl)-2-(2-(piperidin-1-yl)ethoxy)quinoline-6-carboxamide **17**

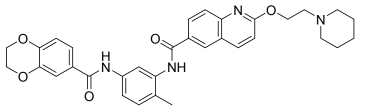

4-(2-Hydroxyethyl)piperidine (0.197 mL, 1.49 mmol) was added to a suspension of NaH (60%, 57.0 mg, 1.42 mmol) in anhydrous THF at 0 ºC. The reaction mixture was stirred for 5 minutes, then allowed to warm to room temperature and stirred for 35 minutes before 6-bromo-2-chloroquinoline (300 mg, 1.24 mmol) was added. The reaction mixture was then heated to reflux. After 4.5 h the reaction mixture was cooled to room temperature, diluted with first water, and then saturated aqueous $NaHCO_3$ solution. This mixture was extracted with DCM three times. The combined organic layers were washed with water, dried over $MgSO_4$ and concentrated. The crude product was purified by column chromatography using a gradient of 4-5% MeOH in DCM to give 6-bromo-2-(2-(piperidin-1-yl)ethoxy)quinoline as pale yellow oil (334 mg, 81%). ^{1}H NMR (500 MHz, $CDCl_3$) δ 7.89 (d, *J* = 8.9 Hz, 1H), 7.87 – 7.84 (m, 1H), 7.72 – 7.65 (m, 2H), 6.95 (d, *J* = 8.8 Hz, 1H), 4.62 (t, *J* = 6.1 Hz, 2H), 2.83 (t, *J* = 6.1 Hz, 2H), 1.63 (m, 6H), 1.53 – 0.46 (m, 4H). HRMS (ESI$^+$): calcd for $C_{16}H_{20}{}^{79}BrN_2O$ $(M + H)^+$ 335.0754, found 335.0787.

n-BuLi (1.62 M in hexanes, 0.742 mL, 1.21 mmol) was added dropwise to a solution of 6-bromo-2-(2-(piperidin-1-yl)ethoxy)quinoline (325 mg, 0.969 mmol) in anhydrous THF (3.25 mL) at -78 ºC. The reaction mixture was stirred at -78 ºC for 50 minutes before solid CO_2 was added. After stirring for 5 minutes, the reaction mixture was allowed to warm to room temperature. The reaction was quenched with water and reduced in vacuo to remove the THF. The remaining residue was diluted with water and washed with ethyl acetate. The aqueous layer was then acidified to pH 3 by addition of aqueous 2M HCl and concentrated to dryness to give the product as off-white amorphous solid. The product, 2-(2-(piperidin-1-yl)ethoxy)quinoline-6-carboxylic acid hydrochloride, was used in the next synthetic step without further purification (301 mg, contains LiCl). ^{1}H NMR (500 MHz, DMSO-d_6) δ 8.59 (d, *J* = 2.0 Hz, 1H), 8.47 (d, *J* = 8.9 Hz, 1H), 8.16 (dd, *J* = 8.7, 2.0 Hz, 1H), 7.84 (d, *J* = 8.7 Hz, 1H), 7.16 (d, *J* = 8.9 Hz, 1H), 5.08 – 4.70 (m, 2H), 3.75 – 3.36 (m, 4H), 3.01 (tdd, *J* = 12.3, 9.1, 3.3 Hz, 2H), 1.92 – 1.75 (m, 4H), 1.74 – 1.55 (m, 1H), 1.38 (ddt, *J* = 12.7, 8.1, 4.0 Hz, 1H). HRMS (ESI$^+$): calcd for $C_{17}H_{21}N_2O_3$ $(M + H)^+$ 301.1547, found 301.1534.

HATU (111 mg, 0.293 mmol) was added to a solution of 2-(2-(piperidin-1-yl)ethoxy)quinoline-6-carboxylic acid hydrochloride (93.0 mg, contains LiCl, purity 85%) and DIPEA (0.174 mL, 0.997 mmol) in anhydrous DMF (1.5 mL). The reaction mixture was stirred for 5 minutes, before *N*-(3-amino-4-methylphenyl)2,3-dihydrobenzo[*b*][1,4]dioxine-6-carboxamide (50.0 mg, 0.176 mmol) was added. The resulting reaction mixture was stirred at room temperature overnight. The reaction mixture was then diluted with water and the resulting precipitate was isolated by filtration and washed with water. The crude product was purified by column chromatography using a gradient of 3.5-10% MeOH in DCM to afford the title compound as a white amorphous solid (75.0 mg, 75%). ^{1}H NMR (500 MHz, DMSO-d_6) δ 10.07 (s, 2H), 8.56 (d, *J* = 2.1 Hz, 1H), 8.38 (d, *J* = 8.8 Hz, 1H), 8.22 (dd, *J* = 8.8, 2.1 Hz, 1H), 7.90 – 7.79 (m, 2H), 7.58 (dd, *J* = 8.3, 2.2 Hz, 1H), 7.54 (d, *J* = 2.1 Hz, 1H), 7.51 (dd, *J* = 8.4, 2.2 Hz, 1H), 7.23 (d, *J* = 8.4 Hz, 1H), 7.11 (d, *J* = 8.8 Hz, 1H), 6.98 (d, *J* = 8.4 Hz, 1H), 4.57 (s, 2H), 4.37 – 4.24 (m, 4H), 2.75 (s, 2H), 2.50 (p, *J* = 1.8 Hz, 4H), 2.23 (s, 3H), 1.51 (d, *J* = 7.6 Hz, 4H), 1.38 (s, 2H). ^{13}C NMR (126 MHz, DMSO-d_6) δ 165.39, 164.81, 163.15, 148.00, 146.80, 143.39, 140.71, 137.75, 136.79, 130.57, 130.52, 129.23, 128.88, 128.57, 128.15, 127.21, 124.48, 121.65, 119.08, 118.58, 117.30, 117.12, 114.47, 64.86, 64.48, 63.73, 57.50, 54.76, 25.95, 24.30, 17.95. HRMS (ESI$^+$): calcd for $C_{33}H_{35}N_4O_5$ $(M + H)^+$ 567.2602, found 567.2635.

2-(2-(piperidin-1-yl)propoxy)quinoline-6-carboxylic acid **S8**

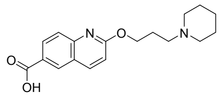

1-Piperidinepropanol (0.235 mL, 1.55 mmol) was added to a suspension of NaH (60%, 59.0 mg, 1.49 mmol) in anhydrous THF (4 mL) at 0 ºC. The reaction mixture was stirred for 5 minutes, then allowed to warm to room temperature and stirred for 40 minutes before 6-bromo-2-chloroquinoline (300 mg, 1.24 mmol) was added. The reaction mixture was then heated to reflux. After 6 h the reaction mixture was cooled to room temperature, and concentrated to remove the THF. The remaining residue was diluted with first water, and then saturated aqueous $NaHCO_3$ solution. This mixture was extracted with DCM three times. The combined organic layers were washed with water, dried over $MgSO_4$ and concentrated. The crude product was purified by column chromatography using a gradient of 2.5-6% of MeOH in DCM to give 6-bromo-2-(2-(piperidin-1-yl)propoxy)quinoline as a pale yellow oil that solidified (329 mg, 76%). ^{1}H NMR (500 MHz, $CDCl_3$) δ 7.89 (d, *J* = 8.9 Hz, 1H), 7.86 (d, *J* = 1.9 Hz, 1H), 7.73 – 7.61 (m, 2H), 6.91 (d, *J* = 8.8 Hz, 1H), 4.50 (t, *J* = 6.5 Hz, 2H), 2.63 – 2.35 (m, 6H), 2.05 (p, *J* = 6.7 Hz, 2H), 1.75 – 1.55 (m, 4H), 1.47 (m, 2H). HRMS (ESI$^+$): calcd for $C_{17}H_{22}{}^{79}BrN_2O$ $(M + H)^+$ 351.0893, found 351.0883.

n-BuLi (1.56 M in hexanes, 0.732 mL, 1.14 mmol) was added dropwise to a solution of 6-bromo-2-(2-(piperidin-1-yl)propoxy)quinoline (319 mg, 0.913 mmol) in anhydrous THF (3.1 mL) at -78 ºC. The reaction mixture was stirred at -78 ºC for 45 minutes before solid CO_2 was added. After stirring for 5 minutes, the reaction mixture was allowed to warm to room temperature. The reaction was quenched with water and reduced in vacuo to remove the THF. The remaining residue was diluted with water and washed with ethyl acetate. The aqueous layer was then acidified to pH 3 by addition of aqueous 2M HCl and concentrated to dryness to give the product as off-white amorphous solid. The product, 2-(2-(piperidin-1-yl)propoxy)quinoline-6-carboxylic acid hydrochloride, was used in the next synthetic step without further purification (347 mg, contains LiCl). ^{1}H NMR (500 MHz, $CDCl_3$) δ 7.89 (d, *J* = 8.9 Hz, 1H), 7.86 (d, *J* = 1.9 Hz, 1H), 7.73 – 7.61 (m, 2H), 6.91 (d, *J* = 8.8 Hz, 1H), 4.50 (t, *J* = 6.5 Hz, 2H), 2.63 – 2.35 (m, 6H), 2.05 (p, *J* = 6.7 Hz, 2H), 1.75 – 1.55 (m, 4H), 1.47 (s, 2H). HRMS (ESI$^+$): calcd for $C_{18}H_{23}N_2O_3$ $(M + H)^+$ 315.1703, found 315.1686.

N-(5-(2,3-Dihydrobenzo[*b*][1,4]dioxine-6-carboxamido)-2-methylphenyl)-2-(2-(piperidin-1-yl)propoxy)quinoline-6-carboxamide **S9**

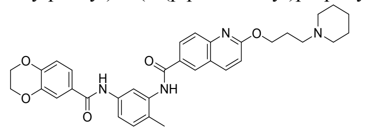

HATU (89.0 mg, 0.234 mmol) was added to a solution of 2-(2-(piperidin-1-yl)propoxy)quinoline-6-carboxylic acid hydrochloride (76.0 mg, 0.188 mmol, contains LiCl) and DIPEA (0.139 mL, 0.797 mmol) in anhydrous DMF (1.35 mL). The reaction mixture was stirred for 5 minutes, before *N*-(3-amino-4-methylphenyl)2,3-dihydrobenzo[*b*][1,4]dioxine-6-carboxamide (40.0 mg, 0.141 mmol) was added. The resulting reaction mixture was stirred at room temperature overnight. The reaction mixture was then diluted with water and the resulting precipitate was isolated by filtration and washed with water. The crude product was purified by column chromatography using a gradient of 2.5-15% MeOH in DCM to give the title compound as an off-white amorphous solid (53 mg, 65%). ^{1}H NMR (500 MHz, DMSO-d_6) δ 10.07 (s, 2H), 8.57 (d, *J* = 2.1 Hz, 1H), 8.38 (d, *J* = 8.9 Hz, 1H), 8.22 (dd, *J* = 8.7, 2.1 Hz, 1H), 7.94 – 7.80 (m, 2H), 7.58 (dd, *J* = 8.3, 2.2 Hz, 1H), 7.54 (d, *J* = 2.1 Hz, 1H), 7.51 (dd, *J* = 8.5, 2.2 Hz, 1H), 7.23 (d, *J* = 8.4 Hz, 1H), 7.10 (d, *J* = 8.8 Hz, 1H), 6.98 (d, *J* = 8.4 Hz, 1H), 4.49 (t, *J* = 6.5 Hz, 2H), 4.35 – 4.26 (m, 4H), 2.50 (m, 6H), 2.23 (s, 3H), 2.01 (m, 2H), 1.56 (m, 4H), 1.42 (m, 2H). ^{13}C NMR (126 MHz, DMSO-d_6) δ 165.38, 164.81, 163.24, 148.04, 146.80, 143.39, 140.69, 137.75, 136.78, 130.57, 130.51, 129.23, 128.89, 128.60, 128.14, 127.17, 124.47, 121.65, 119.09, 118.59, 117.30, 117.12, 114.41, 64.86, 64.66, 64.48, 55.57, 54.13, 25.76, 25.45, 23.47, 17.94. HRMS (ESI$^+$): calcd for $C_{34}H_{37}N_4O_5$ $(M + H)^+$ 581.2759, found 581.2712.

1-(3-(pyrrolidin-1-yl)propoxy)quinoline-6-carboxylic acid **S10**

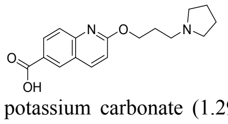

Pyrrolidine (1.00 mL, 12.0 mmol) was added to a suspension of potassium carbonate (1.29g, 9.35 mmol) and 3-bromopropanol (0.651 mL, 7.19 mmol) in anhydrous THF (3 mL) at 0 ºC. The reaction mixture was then allowed to warm to room temperature and stirred at room temperature overnight. The reaction was then diluted with ethyl acetate and filtered through a pad of silica gel. The filtrate was concentrated to give 3-(pyrrolidin-1-yl)propan-1-ol as a colorless oil (645 mg, 69%). ^{1}H NMR (500 MHz, $CDCl_3$) δ 3.88 – 3.73 (m, 2H), 2.82 – 2.67 (m, 2H), 2.66 – 2.51 (m, 4H), 1.81 – 1.75 (m, 4H), 1.75 – 1.69 (m, 2H).

NaH (60%, 59 mg, 1.476 mmol) was added to a solution of 3-(pyrrolidin-1-yl)propan-1-ol (199 mg, 1.540 mmol) in anhydrous THF at 0 ºC. The reaction mixture was stirred for 5 min, then allowed to warm to room temperature and stirred for 35 minutes before 6-bromo-2-chloroquinoline (311 mg, 1.28 mmol) was added.

The reaction mixture was then heated to reflux. After 3.5 h the reaction mixture was cooled to room temperature, and concentrated to remove the THF. The remaining residue was diluted with first water, and then saturated aqueous $NaHCO_3$ solution. This mixture was extracted with DCM three times. The combined organic layers were washed with water, dried over $MgSO_4$ and concentrated. The crude product was purified by column chromatography using a gradient of 3-6% MeOH in DCM to give 6-bromo-2-(3-(pyrrolidin-1-yl)propoxy)quinoline as an off-white amorphous solid (290 mg, 67%). ^{1}H NMR (500 MHz, $CDCl_3$) δ 7.89 (d, *J* = 8.8 Hz, 1H), 7.87 – 7.84 (m, 1H), 7.73 – 7.63 (m, 2H), 6.92 (d, *J* = 8.8 Hz, 1H), 4.53 (t, *J* = 6.5 Hz, 2H), 2.70 – 2.62 (m, 2H), 2.57 (s, 5H), 2.14 – 2.02 (m, 2H), 1.82 (m, 4H). HRMS (ESI$^+$): calcd for $C_{16}H_{20}{}^{79}BrN_2O$ (M + H)$^+$ 335.0754, found 335.0763.

n-BuLi (2.22 M in hexanes, 0.472 mL, 1.048 mmol) was added dropwise to a solution of 6-bromo-2-(3-(pyrrolidin-1-yl)propoxy)quinoline (281 mg, 0.838 mmol) in anhydrous THF (6 mL) at -78 ºC. The reaction mixture was stirred at -78 ºC for 40 minutes before solid CO_2 was added. After stirring for 5 minutes, the reaction mixture was allowed to warm to room temperature. The reaction was quenched with water and reduced in vacuo to remove the THF. The remaining residue was diluted with water and washed with ethyl acetate. The precipitate was carried through in the aqueous layer. The aqueous layer was then acidified to pH 3 by addition of aqueous 2 M HCl. At this point the precipitate dissolved and the solution was concentrated to dryness. The crude product was triturated with acetonitrile and dried to give the product as dull yellow solid. The product, 2-(3-(pyrrolidin-1-yl)propoxy)quinoline-6-carboxylic acid hydrochloride, was used in the next synthetic step without further purification (298 mg, contains LiCl). ^{1}H NMR (500 MHz, DMSO-*d$_6$)* δ 8.57 (d, *J* = 2.0 Hz, 1H), 8.52 – 8.35 (m, 1H), 8.15 (dd, *J* = 8.7, 2.0 Hz, 1H), 7.82 (dd, *J* = 8.7, 0.7 Hz, 1H), 7.10 (d, *J* = 8.9 Hz, 1H), 4.53 (t, *J* = 6.2 Hz, 2H), 3.55 (q, *J* = 5.3 Hz, 2H), 3.36 – 3.24 (m, 2H), 3.08 – 2.89 (m, 2H), 2.29 – 2.13 (m, 2H), 1.99 (q, *J* = 7.3, 6.5 Hz, 2H), 1.93 – 1.79 (m, 2H). HRMS (ESI$^+$): calcd for $C_{17}H_{21}N_2O_3$ (M + H)$^+$ 301.15467, found 301.1501.

N-(5-(2,3-Dihydrobenzo[*b*][1,4]dioxine-6-carboxamido)-2-methylphenyl)-2-(3-(pyrrolidin-1-yl)propoxy)quinoline-6-carboxamide **S11**

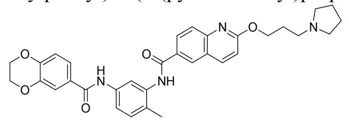

HATU (121 mg, 0.319 mmol) was added to a solution of 2-(3-(pyrrolidin-1-yl)propoxy)quinoline-6-carboxylic acid hydrochloride (100 mg, contains LiCl) and DIPEA (0.190 mL, 1.085 mmol) in anhydrous DMF (10 mL). The reaction mixture was stirred for 5 minutes, before *N*-(3-amino-4-methylphenyl)2,3-dihydrobenzo[*b*][1,4]dioxine-6-carboxamide (54.0 mg, 0.192 mmol) was added followed by anhydrous DMF (1.5 mL) to rinse the vial. The resulting reaction mixture was stirred at room temperature overnight. The reaction mixture was then diluted with water and the resulting precipitate was isolated by filtration and washed with water. The crude product was purified by column chromatography using a gradient of 5-18% MeOH in DCM to afford the title compound as an off-white amorphous solid (65 mg, 60%).

^{1}H NMR (500 MHz, DMSO-*d$_6$)* δ 10.07 (s, 2H), 8.57 (d, *J* = 2.0 Hz, 1H), 8.38 (d, *J* = 8.8 Hz, 1H), 8.22 (dd, *J* = 8.8, 2.1 Hz, 1H), 7.88 – 7.84 (m, 2H), 7.58 (dd, *J* = 8.3, 2.2 Hz, 1H), 7.54 (d, *J* = 2.2 Hz, 1H), 7.51 (dd, *J* = 8.5, 2.2 Hz, 1H), 7.24 (d, *J* = 8.3 Hz, 1H), 7.10 (d, *J* = 8.8 Hz, 1H), 6.98 (d, *J* = 8.4 Hz, 1H), 4.49 (t, *J* = 6.6 Hz, 2H), 4.33-4.28 (m, 4H), 2.65 (br s, 2H), 2.54 (br s, 4H), 2.23 (s, 3H), 1.99 (p, *J* = 6.8 Hz, 2H), 1.72 (br s, 4H). ^{13}C NMR (126 MHz, DMSO-*d$_6$)* δ 164.95, 164.36, 162.86, 147.62, 146.35, 142.94, 140.21, 137.31, 136.34, 130.12, 130.03, 128.78, 128.42, 128.15, 127.69, 126.73, 124.01, 121.21, 118.63, 118.13, 116.85, 116.67, 113.98, 64.40, 64.33, 64.03, 53.59, 52.24, 27.57, 23.07, 17.49. HRMS (ESI$^+$): calcd for $C_{33}H_{35}N_4O_5$ (M + H)$^+$ 567.2602, found 567.2698.

N-(5-(2,3-Dihydrobenzo[*b*][1,4]dioxine-6-carboxamido)-2-fluorophenyl)-2-(2-(pyrrolidin-1-yl)ethoxy)quinoline-6-carboxamide **S12**

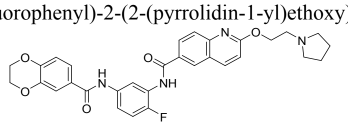

2-Fluoro-5-nitroaniline (27.3 mg, 0.175 mmol), 2-(2-(pyrrolidin-1-yl)ethoxy)quinoline-6-carboxylic acid hydrochloride (50 mg, 0.175 mmol) and EDC (67.0 mg, 0.349 mmol) were dissolved in anhydrous DMF (1.0 mL) and pyridine (0.070 mL, 0.873 mmol) was added dropwise. The mixture was stirred at 20 °C for 5 h. The reaction mixture was diluted with DCM/MeOH and washed with saturated aqueous $NaHCO_3$. Purification by column chromatography using a gradient of 0-50% MeOH in DCM afforded the desired product *N*-(2-fluoro-5-nitrophenyl)-2-(2-(pyrrolidin-1-yl)ethoxy)quinoline-6-carboxamide as a pale yellow solid (40 mg). LCMS (ESI$^+$): t_R=1.13 min, m/z 425.16 (M + H)$^+$.

N-(2-fluoro-5-nitrophenyl)-2-(2-(pyrrolidin-1-yl)ethoxy)quinoline-6-carboxamide (40 mg, 0.094 mmol), ammonium chloride (35.3 mg, 0.66 mmol) and iron powder (36.8 mg, 0.66 mmol) were suspended in a mixture of ethanol (1.5 mL) and water (0.5 mL) and the resulting mixture was heated at 90 °C for 1 h. Then the reaction mixture was cooled to room temperature and filtered through Celite®. The solvents were removed under vacuo to afford *N*-(5-amino-2-fluorophenyl)-2-(2-(pyrrolidin-1-yl)ethoxy)quinoline-6-carboxamide as a light beige solid (37.2 mg). LCMS (ESI$^+$): t_R=0.79 min, m/z 395.19 (M + H)$^+$.

N-(5-Amino-2-fluorophenyl)-2-(2-(pyrrolidin-1-yl)ethoxy)quinoline-6-carboxamide (37.2 mg, 0.094 mmol), 2,3-dihydrobenzo[*b*][1,4]dioxine-6-carboxylic acid (16.90 mg, 0.094 mmol) and EDC (45.0 mg, 0.235 mmol) were dissolved in anhydrous DMF (0.6 mL), then pyridine (0.038 mL, 0.469 mmol) was added dropwise and the resulting mixture was stirred at 20 °C for 18 h. The reaction mixture was diluted with DCM/MeOH and washed with water (5 mL) to afford a pale yellow solid as crude product, which was purified by column chromatography using a gradient of 0-10% MeOH in DCM. This residue was then re-purified via semi-preparative TLC (DCM/MeOH 9/1) to afford the title compound as a white amorphous solid (5 mg, 9.3% over 3 steps). ^{1}H-NMR (500 MHz, MeOD): δ 8.48 (d, *J* = 2.04 Hz, 1H), 8.29 (d, *J* = 8.85 Hz, 1H), 8.20 (dd, *J* = 8.17, 2.04 Hz, 1H), 8.17 (dd, *J* = 6.81, 2.72 Hz, 1H), 7.92 (d, *J* = 8.17 Hz, 1H), 7.62 – 7.56 (m, 1H), 7.50 (d, *J* = 2.04 Hz, 1H), 7.48 (dd, *J* = 8.17, 2.04 Hz, 1H), 7.23 (app t, *J* = 9.79 Hz, 1H), 7.09 (d, *J* = 8.85 Hz, 1H), 6.96 (d, *J* = 8.17 Hz, 1H), 4.70 (t, *J* = 5.32 Hz, 2H), 4.36 – 4.28 (m, 4H), 3.08 (t, *J* = 5.25 Hz, 2H), 2.84 – 2.76 (m, 4H), 1.91 – 1.85 (m, 4H). ^{13}C NMR (126 MHz, MeOD) δ 166.91, 166.59, 163.24, 152.49 (d, *J* = 249.95 Hz), 148.40, 147.13, 143.51, 139.86, 134.85 (d, *J* = 2.13 Hz), 129.65, 127.92, 127.43, 127.03, 125.60, 125.41 (d, *J* = 11.60 Hz), 124.46, 120.76, 119.54 (d, *J* = 9.28 Hz), 119.03, 116.81, 116.64, 115.23 (d, *J* = 18.56 Hz), 113.86, 64.54, 64.15, 64.08, 54.37, 54.13, 22.80. HRMS (ESI$^+$): calcd for $C_{31}H_{30}FN_4O_5$ (M + H)$^+$, 557.2195; found 557.2196.

N-(3-amino-4-chlorophenyl)-2,3-dihydrobenzo[*b*][1,4]dioxine-6-carboxamide **S13**

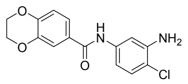

Oxalyl chloride (0.141 mL, 1.67 mmol) was added dropwise to a solution of 1,4-benzodioxane-6-carboxylic acid (250 mg, 1.39 mmol) and *N*,*N*-dimethylformamide (3 µL, 0.035 mmol) in anhydrous DCM (7 mL) under an inert atmosphere at room temperature. Effervescence was observed and the reaction was stirred for 2 h. The reaction mixture was concentrated, anhydrous DCM was added (10 mL) and the reaction concentrated again. The residue was re-dissolved in anhydrous DCM (3 mL, followed by 3 mL, then 1 mL to rinse out the flask) and added dropwise to a solution of 4-chloro-3-nitroaniline (239 mg, 1.39 mmol) and pyridine (0.224 mL, 2.78 mmol) in anhydrous DCM (7 mL). The reaction was stirred for 4 h. The solvent was removed in vacuo and the resulting residue was taken up in a small volume of MeOH. The solid was precipitated by addition of water. The precipitate was isolated by filtration, washed well with water and dried under high vacuum to afford the product as a dark yellow amorphous solid (417 mg, 90%).

A mixture of *N*-(4-chloro-3-nitrophenyl)-2,3-dihydrobenzo[*b*][1,4]dioxine-6-carboxamide (188 mg, 0.562 mmol), ammonium chloride (210 mg, 3.93 mmol), and iron powder (220 mg, 3.93 mmol) in ethanol (2.9 mL) and water (0.95 mL) was heated to reflux overnight. The reaction was allowed to cool to room temperature and filtered through Celite®, eluting with a mixture of EtOH in EtOAc. The reaction mixture was concentrated in vacuo and the resulting residue partitioned between saturated aqueous $NaHCO_3$ solution and EtOAc. The organic layer was washed with water and brine, dried overMgSO$_4$, and concentrated in vacuo to afford the crude product as a light brown amorphous solid (156 mg, 91%).

N-(2-Chloro-5-(2,3-dihydrobenzo[*b*][1,4]dioxine-6-carboxamido)phenyl)-2-(2-(pyrrolidin-1-yl)ethoxy)quinoline-6-carboxamide **S14**

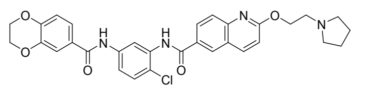

2-(2-(pyrrolidin-1-yl)ethoxy)quinoline-6-carboxylic acid hydrochloride[5] (75.0 mg, 0.232 mmol) was suspended in thionyl chloride (2 mL) and the reaction mixture heated to 60 ºC for 4 h. The reaction mixture was allowed to cool and the thionyl chloride was removed in vacuo. The residue was re-dissolved in anhydrous DCM and then the solvent was removed in vacuo. This procedure was repeated twice. The acid chloride was re-suspended in anhydrous DCM (2 mL) then *N*-(3-amino-4-chlorophenyl)-2,3-dihydrobenzo[*b*][1,4]dioxine-6-carboxamide (78.0 mg, 0.256 mmol), followed by triethylamine (0.162 mL, 1.162 mmol) was added. Not all the reagents were fully solubilized therefore anhydrous dioxane was added (1 mL), however, this did not lead to an improvement. The reaction mixture was allowed to stir at room temperature overnight. The reaction mixture was concentrated in vacuo and the resulting residue purified by Isolute SCX-II chromatography (eluting with MeOH, followed by 10% 2M NH_3 in MeOH). The crude product was further purified by column chromatography using a gradient of 0-10% MeOH in DCM, followed by purification using preparative TLC eluting with 5% MeOH in DCM. The preparative TLC elution was carried out twice to afford the title compound as a white amorphous solid (0.9 mg, 0.7%). ^{1}H NMR (500 MHz, MeOD) δ 8.54 (d, *J* = 2.0 Hz, 1H), 8.37 (d, *J* = 8.9 Hz, 1H), 8.27 – 8.24 (m, 2H), 7.96 (d, *J* = 8.8 Hz, 1H), 7.64 (dd, *J* = 8.8, 2.5 Hz, 1H), 7.52 – 7.47 (m, 3H), 7.17 (d, *J* = 8.9 Hz, 1H), 6.96 (d, *J* = 8.3 Hz, 1H), 4.35 – 4.29 (m, 4H), 3.73 – 3.67 (m, 2H), 3.49 – 3.41 (m, 2H), 2.15 – 2.10 (s, 4H), 1.36 – 1.30 (m, 4H). HRMS (ESI$^+$): calcd for $C_{31}H_{30}{}^{35}ClN_4O_5$ (M + H)$^+$ 573.1905, found 573.1997.

N-(5-(2,3-Dihydrobenzo[*b*][1,4]dioxine-6-carboxamido)-2-fluorophenyl)-2-(3-(pyrrolidin-1-yl)propoxy)quinoline-6-carboxamide **S15**

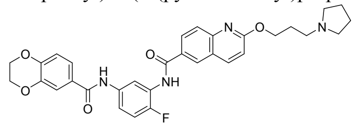

N-(3-Amino-4-fluorophenyl)-2,3-dihydrobenzo[*b*][1,4]dioxine-6-carboxamide (100 mg, 0.347 mmol), 2-(3-(pyrrolidin-1-yl)propoxy)quinoline-6-carboxylic acid (156 mg, 0.520 mmol) and EDC (166 mg, 0.867 mmol) were dissolved in anhydrous DMF (2 mL,) and pyridine (0.140 mL, 1.73 mmol) was added dropwise. The reaction mixture was allowed to stir at room temperature for 72 h, then it was poured onto water and the resulting precipitate was washed with water. The residue was purified by column chromatography using a gradient of 0-10% MeOH in DCM, washed with water and triturated in diethyl ether to afford the title compound as a beige amorphous solid (30.0 mg, 15%). ^{1}H-NMR (500 MHz, DMSO-d_6) δ 10.32 (br s, 1H), 10.19 (br s, 1H), 8.59 (d, *J* = 2.21 Hz, 1H), 8.41 (d, *J* = 8.84 Hz, 1H), 8.23 (dd, *J* = 8.84, 2.21 Hz, 1H), 8.13 (dd, *J* = 8.84, 2.21 Hz, 1H), 7.87 (d, *J* = 8.84 Hz, 1H), 7.66 – 7.61 (m, 1H), 7.55 (d, *J* = 2.21 Hz, 1H), 7.52 (dd, *J* = 8.11, 2.21 Hz, 1H), 7.29 (app t, *J* = 10.23 Hz, 1H), 7.12 (d, *J* = 8.84 Hz, 1H), 6.99 (d, *J* = 8.84 Hz, 1H), 4.53 (t, *J* = 5.57 Hz, 2H), 4.34 – 4.28 (m, 4H), 3.41 –2.66 (m, 6H), 2.21 – 2.09 (m, 2H), 1.93 – 1.80 (m, 4H). ^{13}C NMR (126 MHz, DMSO-d_6) δ 165.50, 164.95, 163.36, 151.38 (d, *J* = 244.33 Hz), 148.19, 146.91, 143.41, 140.75, 135.88 (d, *J* = 2.31 Hz), 129.87, 128.95, 128.90, 127.93, 127.25, 125.88 (d, *J* = 12.81 Hz), 124.44, 121.72, 119.30, 119.12 (d, *J* = 9.61 Hz), 117.36, 117.16, 116.03 (d, *J* = 19.22 Hz), 114.49, 64.88, 64.63, 64.49, 53.88, 52.50, 27.57, 23.47. HRMS (ESI$^+$): calcd for $C_{32}H_{32}FN_4O_5$ (M + H)$^+$, 571.2351; found 571.2321.

N-(5-(2,3-Dihydrobenzo[*b*][1,4]dioxine-6-carboxamido)-2-fluorophenyl)-2-(3-(piperidin-1-yl)propoxy)quinoline-6-carboxamide **S16**

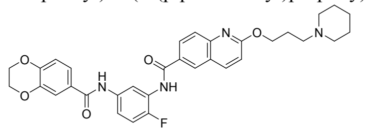

N-(3-Amino-4-fluorophenyl)-2,3-dihydrobenzo[b][1,4]dioxine-6-carboxamide (100 mg, 0.347 mmol), 2-(2-(piperidin-1-yl)propoxy)quinoline-6-carboxylic acid (164 mg, 0.52 mmol) and EDC (166 mg, 0.867 mmol) were dissolved in anhydrous DMF (2 mL,) and pyridine (0.140 mL, 1.734 mmol) was added dropwise. The resulting mixture was allowed to stir at room temperature for 72 h, then it was poured onto water and the resulting precipitate was washed with water and purified by column chromatography using a gradient of 0-10% MeOH in DCM, followed by wash in water (5 mL) and trituration in diethyl ether to afford the title compound as a beige amorphous solid (40 mg, 20%). ^{1}H-NMR (500 MHz, DMSO-d_6): δ 10.32 (br s, 1H), 10.19 (br s, 1H), 8.59 (d, *J* = 1.76 Hz, 1H), 8.39 (d, *J* = 8.79 Hz, 1H), 8.22 (dd, *J* = 8.79, 1.76 Hz, 1H), 8.13 (dd, *J* = 7.03 Hz, 2.34 Hz, 1H), 7.86 (d, *J* = 8.79 Hz, 1H), 7.67 – 7.62 (m, 1H), 7.55 (d, *J* = 1.76 Hz, 1H), 7.52 (dd, *J* = 8.21, 1.76 Hz, 1H), 7.28 (app t, *J* = 9.96 Hz, 1H), 7.11 (d, *J* = 8.90 Hz, 1H), 6.99 (d, *J* = 8.90 Hz, 1H), 4.51 (t, *J* = 6.29 Hz, 2H), 4.34 – 4.28 (m, 4H), 3.16 – 2.65 (m, 4H), 2.36 – 1.30 (m, 10H). ^{13}C NMR (126 MHz, DMSO-d_6) δ 165.51, 164.97, 163.26, 152.37 (d, *J* = 242.81 Hz), 148.18, 146.91, 143.41, 140.77, 135.87 (d, *J* = 2.19 Hz), 129.87, 128.96, 128.89, 127.92, 127.25, 125.88 (d, *J* = 14.51 Hz), 124.45, 121.72, 119.33, 119.14 (d, *J* = 8.29 Hz), 117.36, 117.16, 116.01 (d, *J* = 20.73 Hz), 114.48, 64.87, 64.59, 64.49, 55.03, 53.82, 25.40, 24.99, 23.67. HRMS (ESI$^+$): calcd for $C_{33}H_{34}FN_4O_5$ (M + H)$^+$, 585.2508; found 585.2485.

Synthetic Route IV

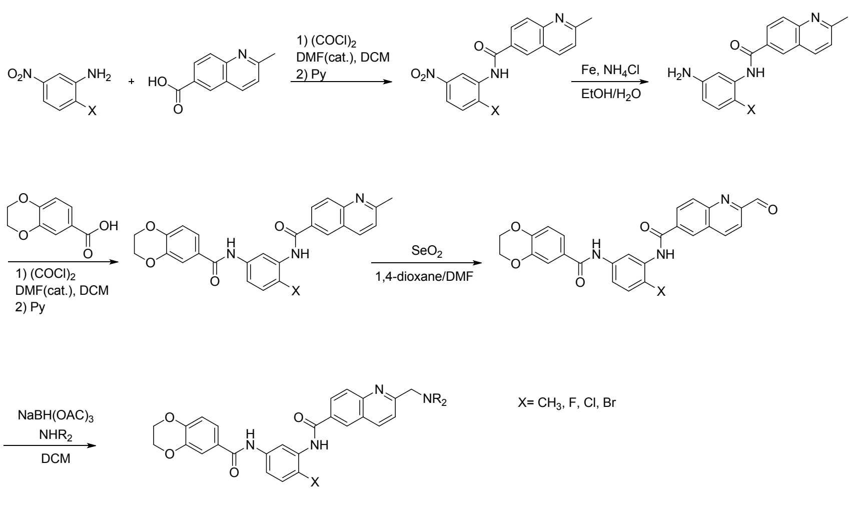

2-Methyl-*N*-(2-methyl-5-nitrophenyl)quinoline-6-carboxamide **S28**

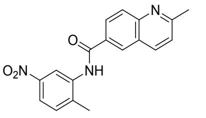

To a suspension of 2-methylquinoline-6-carboxylic acid (3.69 g, 19.72 mmol) in anhydrous DCM (35 mL), oxalyl chloride (1.8 mL, 20.15 mmol) and DMF (310 µL, 4.00 mmol) were added dropwise and the resulting green solution was allowed to stir at 20 °C for 3 h after which it was concentrated under vacuum to afford a

dry pale green solid. The solid was dissolved in pyridine (35 mL) and 2-methyl-5-nitroaniline (3.0 g, 19.72 mmol) was added in one portion and was allowed to stir for 2 h. The reaction mixture was reduced *in vacuo* until dryness. The remaining residue was triturated with diethyl ether. The crude product was purified by column chromatography on silica gel in gradient DCM/EtOH 0-50 % to afford the title compound as a yellow amorphous solid (5.57 g, 88 %). ^{1}H NMR (500 MHz, DMSO-d_6) δ 10.36 (s, 1H), 8.63 (d, *J* = 1.8 Hz, 1H), 8.43 (d, *J* = 8.4 Hz, 1H), 8.40 (d, *J* = 2.4 Hz, 1H), 8.25 (dd, *J* = 8.7, 2.0 Hz, 1H), 8.07 (d, *J* = 2.9 Hz, 1H), 8.05 (d, *J* = 3.0 Hz, 1H), 7.60 (d, *J* = 8.5 Hz, 1H), 7.54 (d, *J* = 8.4 Hz, 1H), 2.71 (s, 3H), 2.44 (s, 3H). ^{13}C NMR (126 MHz, DMSO-d_6) δ 165.54, 161.05, 146.20, 144.90, 143.38, 142.15, 137.76, 132.04, 130.08, 129.93, 129.31, 126.62, 126.09, 124.16, 121.00, 120.94, 23.98, 18.82. HRMS (ESI$^+$): calcd for $C_{18}H_{15}NaN_3O_3$ (M + Na)$^+$, 344.1006; found 344.0999.

N-(5-Amino-2-methylphenyl)-2-methylquinoline-6-carboxamide **S29**

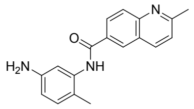

2-Methyl-*N*-(2-methyl-5-nitrophenyl)quinoline-6-carboxamide (4.0 g, 12.45 mmol), iron powder (6.95 g, 124.0 mmol) and ammonium chloride (2.12 g, 124.0 mmol) in ethanol (50 mL) and water (12.5 mL) were allowed to stir at 90 °C for 1 h. Then the reaction mixture was allowed to cool to room temperature and was filtered through a short pad of Celite®. The eluate was concentrated *in vacuo* to afford the title compound as a light yellow amorphous solid, which was carried onto the next step without purification (1.95 g, 54 %). ^{1}H NMR (500 MHz, DMSO-d_6) δ 9.86 (s, 1H), 8.61 – 8.51 (m, 1H), 8.38 (d, *J* = 8.4 Hz, 1H), 8.21 (dd, *J* = 8.8, 1.7 Hz, 1H), 8.00 (d, *J* = 8.8 Hz, 1H), 7.51 (d, *J* = 8.4 Hz, 1H), 6.91 (d, *J* = 8.1 Hz, 1H), 6.65 (d, *J* = 2.0 Hz, 1H), 6.42 (dd, *J* = 8.1, 2.2 Hz, 1H), 4.94 (s, 2H), 2.70 (s, 3H), 2.09 (s, 3H). ^{13}C NMR (126 MHz, DMSO-d_6) δ 165.18, 161.09, 148.86, 147.31, 137.58, 137.01, 132.25, 130.87, 128.72, 128.49, 128.38, 125.84, 123.38, 120.72, 112.75, 112.61, 25.49, 17.50. HRMS (ESI$^+$): calcd for $C_{18}H_{18}N_3O$ (M + H)$^+$, 292.1444; found 292.1446.

N-(5-(2,3-Dihydrobenzo[*b*][1,4]dioxine-6-carboxamido)-2-methylphenyl)-2-methylquinoline-6-carboxamide **S30**

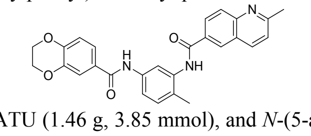

2-Methyl-6-quinolinecarboxylic acid (600 mg, 3.21 mmol), HATU (1.46 g, 3.85 mmol), and *N*-(5-amino-2-methylphenyl)-2-methylquinoline-6-carboxamide (910 mg, 3.21 mmol) were suspended in anhydrous DMF (25 mL) and *N,N*-diisopropylethylamine (1.12 mL, 6.41 mmol) was added dropwise. The resulting solution was allowed to stir at room temperature under inert atmosphere overnight. The reaction mixture was poured onto water and the resulting precipitate was filtered and washed with water, to afford the title compound as an off-white amorphous solid (1.38 g, 95 %). ^{1}H NMR (500 MHz, DMSO-d_6) δ 10.14 (s, 1H), 10.08 (s, 1H), 8.61 (s, 1H), 8.41 (br d, *J* = 7.3 Hz, 1H), 8.24 (d, *J* = 8.7 Hz, 1H), 8.03 (d, *J* = 8.8 Hz, 1H), 7.87 (d, *J* = 2.1 Hz, 1H), 7.64 – 7.56 (m, 2H), 7.54 (d, *J* = 2.1 Hz, 1H), 7.51 (dd, *J* = 8.4, 2.2 Hz, 1H), 7.24 (d, *J* = 8.5 Hz, 1H), 6.98 (d, *J* = 8.4 Hz, 1H), 4.35 – 4.26 (m, 4H), 2.71 (s, 3H), 2.24 (s, 3H). ^{13}C NMR (126 MHz, DMSO-d_6) δ 165.44, 164.82, 161.21, 148.93, 146.80, 143.39, 137.77, 137.62, 136.74, 131.97, 130.59, 129.23, 128.81, 128.64, 128.38, 128.14, 125.86, 123.44, 121.66, 119.07, 118.63, 117.30, 117.12, 64.86, 64.48, 25.51, 17.94. HRMS (ESI$^+$): calcd for $C_{27}H_{24}N_3O_4$ (M + H)$^+$, 454.1761; found 454.1733.

N-(5-(2,3-Dihydrobenzo[*b*][1,4]dioxine-6-carboxamido)-2-methylphenyl)-2-formylquinoline-6-carboxamide **S31**

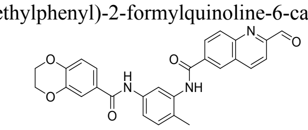

A solution of *N*-(5-(2,3-dihydrobenzo[*b*][1,4]dioxine-6-carboxamido)-2-methylphenyl)-2-methylquinoline-6-carboxamide (0.165 g, 0.364 mmol) and selenium dioxide (0.444 g, 0.400 mmol) in anhydrous 1,4-dioxane (0.6 mL) and anhydrous DMF (0.6 mL) was heated at 150 °C for 1 h after which the reaction mixture was allowed to cool to room temperature, diluted with DCM and filtered through a pad of Celite®. The filtrate was concentrated under vacuum to afford the crude product as a brown solid which was taken directly onto the next step without purification (0.17 g). ^{1}H-NMR (500 MHz, DMSO-d_6): δ 10.29 (s, 1H), 10.17 (s, 1H), 10.09 (s, 1H), 8.81 – 8.77 (m, 1H), 8.41 (dd, *J* = 8.29, 1.66 Hz, 1H), 8.36 (d, *J* = 8.29 Hz, 1H), 8.17 – 8.12 (m, 1H), 8.08 (d, *J* = 9.12 Hz, 1H), 7.90 (d, *J* = 2.49 Hz, 1H), 7.58 (dd, *J* = 8.29, 2.49 Hz, 1H), 7.54 (d, *J* = 2.49 Hz, 1H), 7.52 (dd, *J* = 8.29, 2.49 Hz, 1H), 7.25 (d, *J* = 8.29 Hz, 1H), 6.98 (d, *J* = 8.29 Hz, 1H), 4.35 – 4.28 (m, 4H), 2.31 (s, 3H).

N-(5-(2,3-Dihydrobenzo[*b*][1,4]dioxine-6-carboxamido)-2-methylphenyl)-2-(piperidin-1-ylmethyl)quinoline-6-carboxamide **14**

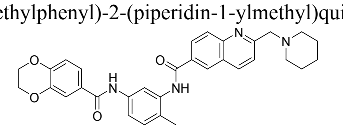

A solution of *N*-(5-(2,3-dihydrobenzo[*b*][1,4]dioxine-6-carboxamido)-2-methylphenyl)-2-formylquinoline-6-carboxamide (58 mg, 0.124 mmol) and piperidine (31.7 mg, 0.372 mmol) in anhydrous DCM (1.2 mL), was allowed to stir at room temperature for 7 h. Then sodium triacetoxyborohydride (79 mg, 0.372 mmol) was added in one portion at room temperature and the resulting mixture was allowed to stir under an inert argon atmosphere for 2 h at room temperature. The reaction mixture was diluted with DCM (5 mL), washed with brine (1 x 5 mL) and the aqueous phase was extracted with DCM/MeOH 9:1 mixture (3 x 5 mL). The organic layers were combined, dried over Na_2SO_4, filtered and concentrated under reduced pressure. Purification by column chromatography using a gradient of 0-10% MeOH in DCM afforded the title compound as a pale yellow solid (20 mg, 30%). ^{1}H NMR (500 MHz, DMSO-d_6) δ 10.15 (s, 1H), 10.08 (s, 1H), 8.63 (s, 1H), 8.48 (d, *J* = 8.1 Hz, 1H), 8.26 (d, *J* = 9.0 Hz, 1H), 8.08 (d, *J* = 8.6 Hz, 1H), 7.91 – 7.85 (m, 1H), 7.73 (d, *J* = 8.4 Hz, 1H), 7.61 – 7.56 (m, 1H), 7.56 – 7.47 (m, 2H), 7.25 (d, *J* = 8.4 Hz, 1H), 6.98 (d, *J* = 8.4 Hz, 1H), 4.31 (q, *J* = 4.7 Hz, 4H), 3.77 (s, 2H), 2.43 (s, 4H), 2.24 (s, 3H), 1.55 (s, 4H), 1.43 (s, 2H). ^{13}C NMR (126 MHz, DMSO-d_6) δ 165.46, 164.94, 163.18, 153.31, 151.37, 148.11, 146.91, 143.41, 140.84, 135.86, 129.96, 129.00, 128.92, 127.92, 127.24, 125.92, 125.81, 124.50, 121.72, 119.34, 119.11, 117.34, 117.17, 116.07, 115.91, 114.41, 64.87, 64.49, 63.99, 53.66, 52.05, 23.28. HRMS (ESI$^+$): calcd for $C_{32}H_{32}N_4NaO_4$ (M + Na)$^+$, 559.2316; found 559.2325.

N-(5-(2,3-Dihydrobenzo[*b*][1,4]dioxine-6-carboxamido)-2-methylphenyl)-2-((4-isopropylpiperazin-1-yl)methyl)quinoline-6-carboxamide **16**

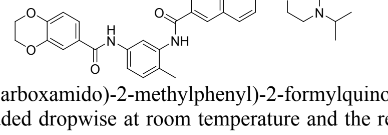

To a solution of *N*-(5-(2,3-dihydrobenzo[*b*][1,4]dioxine-6-carboxamido)-2-methylphenyl)-2-formylquinoline-6-carboxamide (170 mg, 0.364 mmol) in anhydrous DCM, 1-isopropylpiperazine (0.16 mL, 1.09 mmol) was added dropwise at room temperature and the resulting mixture was allowed to stir under an inert argon atmosphere for 2.5 h. Then sodium triacetoxyborohydride (231 mg, 1.09 mmol) was added in one portion and the resulting mixture was allowed to stir overnight at room temperature. The reaction was diluted with DCM (5 mL) and washed with $NaHCO_3$ saturated aqueous solution (5 mL). The aqueous phase was extracted with DCM (3 x 5 mL) and the combined organic layers were dried over Na_2SO_4, filtered and concentrated under reduced pressure to afford a brown oil as crude product. Purification by column chromatography using a gradient of 0-10% MeOH in DCM afforded the title compound as a beige solid (30 mg, 14%). ^{1}H NMR (500 MHz, DMSO-d_6) δ 10.15 (s, 1H), 10.08 (s, 1H), 8.65 – 8.61 (m, 1H), 8.48 (d, *J* = 8.6 Hz, 1H), 8.29 – 8.23 (m, 1H), 8.08 (d, *J* = 8.8 Hz, 1H), 7.90 – 7.86 (m, 1H), 7.72 (d, *J* = 8.5 Hz, 1H), 7.59 (dd, *J* = 8.3, 2.0 Hz, 1H), 7.56 – 7.48 (m, 2H), 7.25 (d, *J* = 8.4 Hz, 1H), 6.98 (d, *J* = 8.4 Hz, 1H), 4.31 (q, *J* = 5.0 Hz, 4H), 3.78 (s, 2H), 2.65 – 2.59 (m, 1H), 2.47 (s, 8H), 2.24 (s, 3H), 0.96 (d, *J* = 6.5 Hz, 6H). ^{13}C NMR (126 MHz, DMSO-d_6) δ 165.37, 164.82, 162.08, 148.63, 146.81,

143.39, 137.89, 137.78, 136.69, 132.37, 130.60, 129.22, 129.15, 128.63, 128.41, 128.14, 128.09, 126.69, 122.21, 121.66, 119.06, 118.64, 117.30, 117.12, 64.92, 64.86, 64.49, 54.05, 53.94, 48.47, 31.16, 28.11, 18.71, 17.93. HRMS (ESI$^+$): calcd for $C_{34}H_{38}N_5O_4$ (M + H)$^+$, 580.2918; found 580.2896.

N-(2-Chloro-5-(2,3-dihydrobenzo[*b*][1,4]dioxine-6-carboxamido)phenyl)-2-formylquinoline-6-carboxamide **S35**

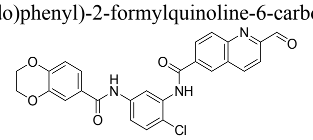

To a suspension of 2-methylquinoline-6-carboxylic acid (1.5 g, 8.01 mmol) in anhydrous DCM (40 mL), DMF (1.40 µl, 0.018 mmol) and oxalyl chloride (0.74 mL, 8.74 mmol) were added dropwise and the resulting green solution was allowed to stir at 20 °C for 3 h, after which it was concentrated under vacuum to afford a dry pale green solid. The solid was dissolved in pyridine (40mL) and 2-chloro-5-nitroaniline (1.26 g, 7.28 mmol) was added in one portion. The resulting dark yellow suspension was allowed to stir for 2 h, after which it was poured onto water and the yellow precipitate was filtered and washed several times with water, diethyl ether and finally with a minimum amount of DCM to afford the product as a yellow amorphous solid which was used without further purification (2.20 g, 88%). ^{1}H-NMR (500 MHz, DMSO-d_6^{1}H-NMR (500 MHz, DMSO-d_6): δ 10.59 (s, 1H), 8.65 (d, *J* = 2.0 Hz, 1H), 8.60 (d, *J* = 2.7 Hz, 1H), 8.44 (d, *J* = 8.6 Hz, 1H), 8.25 (dd, *J* = 8.6, 2.0 Hz, 1H), 8.15 (dd, *J* = 8.6, 2.7 Hz, 1H), 8.06 (d, *J* = 8.4 Hz, 1H), 7.91 (d, *J* = 8.6 Hz, 1H), 7.55 (d, *J* = 8.0 Hz, 1H), 2.71 (s, 3H). HRMS (ESI$^+$): calcd for $C_{17}H_{13}{}^{35}ClN_3O_3$ (M + H)$^+$, 342.0640; found 342.0646.

N-(2-chloro-5-nitrophenyl)-2-methylquinoline-6-carboxamide was suspended in water (7mL) and EtOH (21 mL).Ammonium chloride (2.41 g, 45.1 mmol) and iron powder (2.52 g, 45.1 mmol) were added and the resulting suspension was allowed to stir at 90 °C for 1 h. The reaction mixture was allowed to cool to room temperature, diluted with MeOH and DCM and filtered through a pad of Celite®. The resulting filtrate was concentrated under vacuum to afford a light brown amorphous solid as crude product, which was used directly in the next step without purification (2.00 g, 100%). ^{1}H-NMR (500 MHz, DMSO-d_6): δ 9.96 (s, 1H), 8.58 (d, *J* = 2.2 Hz, 1H), 8.41 (d, *J* = 8.7 Hz, 1H), 8.21 (dd, *J* = 8.7, 2.2 Hz, 1H), 8.02 (d, *J* = 8.7 Hz, 1H), 7.53 (d, *J* = 7.6 Hz, 1H), 7.15 (d, *J* = 8.7 Hz, 1H), 6.87 (d, *J* = 2.2 Hz, 1H), 6.50 (dd, *J* = 8.7, 2.2 Hz, 1H), 5.41 (bs, 2H), 2.70 (s, 3H). HRMS (ESI$^+$): calcd for $C_{17}H_{15}{}^{35}ClN_3O$ (M + H)$^+$, 312.0898; found 312.0902.

2,3-Dihydrobenzo[*b*][1,4]dioxine-6-carboxylic acid (1.27 g, 7.06 mmol) was suspended in anhydrous DCM (20 mL), DMF (1.23 µl, 0.016 mmol) and oxalyl chloride (0.65 mL, 7.70 mmol) were added dropwise and the resulting green solution was allowed to stir at 20 °C for 3 h after which it was concentrated under vacuum to afford a dry pale green solid. The solid was dissolved in pyridine (20.0 mL) and *N*-(5-amino-2-chlorophenyl)-2-methylquinoline-6-carboxamide (2.00 g, 6.42 mmol) was added in one portion. The resulting dark yellow suspension was allowed to stir for 2 h after which it was poured onto water and the yellow precipitate was filtered and washed several times with water, diethyl ether and finally with a minimum amount of DCM to afford the crude product as a pale yellow amorphous solid, which was carried onto the next step without purification (1.86 g, 61%). ^{1}H-NMR (500 MHz, DMSO-d_6): δ 10.31 (s, 1H), 10.27 (s, 1H), 8.63 (d, *J* = 1.5 Hz, 1H), 8.43 (d, *J* = 8.8 Hz, 1H), 8.25 (dd, *J* = 8.8, 2.2 Hz, 1H), 8.14 (d, *J* = 2.2 Hz, 1H), 8.04 (d, *J* = 8.8 Hz, 1H), 7.75 (dd, *J* = 8.8, 2.9 Hz, 1H), 7.58-7.49 (m, 4H), 7.00 (d, *J* = 8.8 Hz, 1H), 4.37-4.26 (m, 4H), 2.71 (s, 3H). HRMS (ESI$^+$): calcd for $C_{26}H_{21}{}^{35}ClN_3O_4$ (M + H)$^+$, 474.1215; found 474.1210.

A solution of *N*-(2-chloro-5-(2,3-dihydrobenzo[*b*][1,4]dioxine-6-carboxamido)phenyl)-2-methylquinoline-6-carboxamide (0.500 g, 1.06 mmol) and selenium dioxide (0.129 g, 1.16 mmol) in anhydrous DMF (12.0 mL) and 1,4-dioxane (12.0 mL) was heated at 150 °C for 2 h. A further portion of selenium dioxide (0.129 g, 1.16 mmol) was added to the reaction mixture and stirred at 150 °C for a further 1 h. The reaction mixture was allowed to cool to room temperature and it was diluted with DCM and filtered through a pad of Celite®. The filtrate was concentrated under vacuum to afford the crude product as a yellow amorphous solid which was carried onto the next step without purification (0.515 g). ^{1}H-NMR (500 MHz, DMSO-d_6): δ 10.45 (s, 1H), 10.40 (s, 1H), 10.17 (s, 1H), 8.81 – 8.79 (m, 2H), 8.42 – 8.34 (m, 2H), 8.19 (app t, *J* = 7.47 Hz, 1H), 8.09 (d, *J* = 8.13 Hz, 1H), 7.79 – 7.74 (m, 2H), 7.55 (d, *J* = 1.99 Hz, 1H), 7.52 (dd, *J* = 8.13, 1.99 Hz, 1H), 6.99 (d, *J* = 9.04 Hz, 1H), 4.36 – 4.26 (m, 4H). HRMS (ESI$^+$): calcd for $C_{26}H_{19}{}^{35}ClN_3O_5$ (M + H)$^+$, 488.1013; found 488.1012.

N-(2-Chloro-5-(2,3-dihydrobenzo[*b*][1,4]dioxine-6-carboxamido)phenyl)-2-((4-isopropylpiperazin-1-yl)methyl)quinoline-6-carboxamide **18**

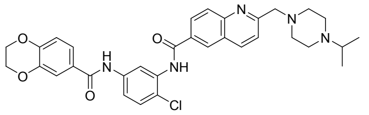

A solution of *N*-(2-chloro-5-(2,3-dihydrobenzo[*b*][1,4]dioxine-6-carboxamido)phenyl)-2-formylquinoline-6-carboxamide (2.00 g, 4.10 mmol) and 1-isopropylpiperazine (1.58 g, 12.3 mmol) in anhydrous DCM (35 mL) was allowed to stir at 20 °C for 6 h, after which sodium triacetoxyborohydride (2.61 g, 12.3 mmol) was added in one portion and the resulting mixture was allowed to stir at 20 °C for 2 h. The reaction was quenched with $NaHCO_3$ saturated aqueous solution (35 mL) and extracted with a DCM/MeOH 9/1 mixture (3 x 35 mL). The crude product (pale yellow solid) was purified by column chromatography using a gradient of 0-10% MeOH in DCM, followed by trituration in diethyl ether to afford the title compound as a pale yellow amorphous solid (0.830 g, 34%). ^{1}H NMR (500 MHz, DMSO-d_6) δ 10.33 (s, 1H), 10.28 (s, 1H), 8.66 (s, 1H), 8.50 (d, *J* = 8.5 Hz, 1H), 8.27 (d, *J* = 8.8 Hz, 1H), 8.20 – 8.04 (m, 2H), 7.81 – 7.64 (m, 2H), 7.62 – 7.46 (m, 3H), 7.00 (d, *J* = 8.4 Hz, 1H), 4.31 (q, *J* = 5.1 Hz, 4H), 3.81 (s, 2H), 2.59 (d, *J* = 43.9 Hz, 5H), 2.45 (br s, 4H), 0.99 (s, 6H). ^{13}C NMR (126 MHz, DMSO-d_6) δ 165.45, 165.11, 161.95, 148.75, 147.03, 143.43, 139.06, 138.02, 135.35, 131.83, 129.81, 129.29, 128.89, 128.37, 127.76, 126.70, 124.04, 122.31, 121.80, 120.31, 119.72, 117.37, 117.23, 64.88, 64.49, 60.05, 53.76, 48.34, 18.44. HRMS (ESI$^+$): calcd for $C_{33}H_{35}{}^{35}ClN_5O_4$ (M + H)$^+$, 600.2372; found 600.2336.

N-(2-Chloro-5-(2,3-dihydrobenzo[*b*][1,4]dioxine-6-carboxamido)phenyl)-2-(piperazin-1-ylmethyl)quinoline-6-carboxamide **19**

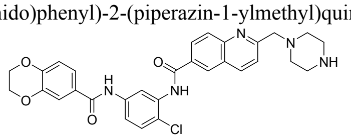

A solution of *N*-(2-chloro-5-(2,3-dihydrobenzo[*b*][1,4]dioxine-6-carboxamido)phenyl)-2-formylquinoline-6-carboxamide (0.300 g, 0.615 mmol) and 1-(*tert*-butyl)piperazine (0.262 g, 1.85 mmol) in anhydrous DCM (5 mL) was allowed to stir at 20 °C for 12 h, after which sodium triacetoxyborohydride (0.391 g, 1.85 mmol) was added in one portion and the resulting mixture was allowed to stir at 20 °C for 2 h. The reaction was quenched with $NaHCO_3$ saturated aqueous solution (5 mL) and extracted with a DCM/MeOH 9/1 mixture (3 x 5 mL). The crude product (pale yellow solid) was purified by column chromatography 0-10% MeOH in DCM, followed by trituration in diethyl ether to afford *tert*-butyl 4-((6-((2-chloro-5-(2,3-dihydrobenzo[*b*][1,4]dioxine-6 carboxamido)phenyl)carbamoyl)quinolin-2-yl)methyl)piperazine-1-carboxylate as a beige solid (0.060 g, 16%). ^{1}H-NMR (500 MHz, DMSO-d_6): δ 10.34 (s, 1H), 10.29 (s, 1H), 8.66 (d, *J* = 1.6 Hz, 1H), 8.50 (d, *J* = 8.6 Hz, 1H), 8.26 (dd, *J* = 8.6, 2.4 Hz, 1H), 8.14 (d, *J* = 2.4 Hz, 1H), 8.09 (d, *J* = 8.6 Hz, 1H), 7.77 – 7.72 (m, 2H), 7.56 (d, *J* = 2.4 Hz, 1H), 7.54 (d, *J* = 1.6 Hz, 1H), 7.53 (dd, *J* = 7.8, 2.4 Hz, 1H), 7.00 (d, *J* = 8.6 Hz, 1H), 4.35 – 4.27 (m, 4H), 3.79 (s, 2H), 2.79 – 2.29 (m, 8H), 1.03 (br s, 9H). To a suspension of *tert*-butyl 4-((6-((2-chloro-5-(2,3-dihydrobenzo[*b*][1,4]dioxine-6-carboxamido)phenyl)carbamoyl)quinolin-2-yl)methyl)piperazine-1-carboxylate (674 mg, 1.02 mmol) in anhydrous DCM (10 mL), TFA (0.78 mL, 10.2 mmol) was added dropwise and the resulting mixture was allowed to stir at 20 °C for 4 h. The reaction mixture was concentrated under vacuum to afford the crude product as a light brown oil. The crude was purified by column chromatography using a gradient of 0-15% MeOH in DCM, followed by trituration in diethyl ether to afford the title compound as a yellow amorphous solid (39 mg, 6.8%). ^{1}H NMR (500 MHz, DMSO-d_6) δ 10.32 (s, 1H), 10.27 (s, 1H), 8.65 (d, *J* = 2.0 Hz, 1H), 8.49 (d, *J* = 8.4 Hz, 1H), 8.26 (dd, *J* = 8.8, 2.0 Hz, 1H), 8.15 (d, *J* = 2.5 Hz, 1H), 8.09 (d, *J* = 8.8 Hz, 1H), 7.76 – 7.71 (m, 2H), 7.58 – 7.47 (m, 3H), 7.00 (d, *J* = 8.4 Hz, 1H), 4.40 – 4.23 (m, 4H), 3.76 (s, 2H), 2.74 (t, *J* = 4.8 Hz, 4H), 2.47 – 2.42 (br m, 4H) (1 proton missing). ^{13}C NMR (126 MHz, DMSO-d_6) δ 165.00, 164.64, 161.79, 148.29, 146.57, 142.97, 138.58, 137.45,

134.89, 131.32, 129.36, 128.81, 128.39, 127.85, 127.31, 126.21, 123.54, 121.85, 121.32, 119.81, 119.23, 116.92, 116.74, 65.11, 64.42, 64.03, 54.26, 45.54. HRMS (ESI+): calcd for $C_{30}H_{29}{}^{35}ClN_5O_4$ (M + H)+ 558.1903, found 558.1885.

N-(2-Chloro-5-(2,3-dihydrobenzo[*b*][1,4]dioxine-6-carboxamido)phenyl)-2-((4-methylpiperazin-1-yl)methyl)quinoline-6-carboxamide **20**

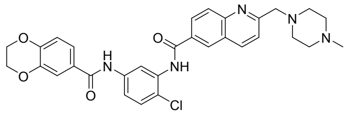

A solution of *N*-(2-chloro-5-(2,3-dihydrobenzo[*b*][1,4]dioxine-6-carboxamido)phenyl)-2-formylquinoline-6-carboxamide (0.100 g, 0.205 mmol) and 1-methylpiperazine (61.6 mg, 0.615 mmol) in anhydrous DCM (2 mL) was allowed to stir at 20 °C for 12 h, after which sodium triacetoxyborohydride (0.130 g, 0.615 mmol) was added in one portion and the resulting mixture was allowed to stir at 20 °C for 2 h. The reaction was quenched with $NaHCO_3$ saturated aqueous solution (5 mL) and extracted with a DCM/MeOH 9/1 mixture (3 x 5 mL). The crude product (brown oil) was purified by column chromatography using a gradient of 0-10% MeOH in DCM to afford the desired product as a white amorphous solid (0.010 g, 8.5%). ^{1}H NMR (500 MHz, DMSO-d_6) δ 10.31 (s, 1H), 10.23 (s, 1H), 8.65 (s, 1H), 8.48 (d, *J* = 8.7 Hz, 1H), 8.30 – 8.26 (m, 1H), 8.15 (s, 1H), 8.07 (d, *J* = 8.8 Hz, 1H), 7.71 (dd, *J* = 8.7, 4.2 Hz, 2H), 7.57 – 7.47 (m, 3H), 6.99 (d, *J* = 8.4 Hz, 1H), 4.31 (q, *J* = 4.9 Hz, 4H), 3.79 (s, 2H), 2.35 (br s, 8H), 2.16 (s, 3H). ^{13}C NMR (126 MHz, DMSO-d_6) δ 165.48, 165.11, 162.14, 148.72, 147.03, 143.44, 139.02, 138.00, 129.80, 129.24, 128.83, 128.41, 127.79, 126.70, 124.01, 122.27, 121.80, 120.25, 119.56, 117.39, 117.22, 117.22, 64.89, 64.84, 64.49, 55.21, 53.40, 46.24. HRMS (ESI+): calcd for $C_{31}H_{31}{}^{35}ClN_5O_4$ (M + H)+, 572.2059; found 572.2031.

N-(2-Chloro-5-(2,3-dihydrobenzo[*b*][1,4]dioxine-6-carboxamido)phenyl)-2-((4-ethylpiperazin-1-yl)methyl)quinoline-6-carboxamide **21**

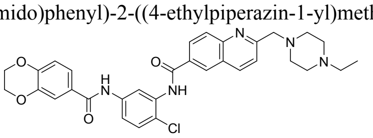

A solution of *N*-(2-chloro-5-(2,3-dihydrobenzo[*b*][1,4]dioxine-6-carboxamido)phenyl)-2-formylquinoline-6-carboxamide (1.03 g, 2.11 mmol) and 1-ethylpiperazine (723 mg, 6.33 mmol) in anhydrous DCM (20 mL) was allowed to stir at 20 °C for 12 h, after which sodium triacetoxyborohydride (1.34 g, 6.33 mmol) was added in one portion and the resulting mixture was allowed to stir at 20 °C for 3 h. The reaction was quenched with $NaHCO_3$ saturated aqueous solution (20 mL) and extracted with a DCM/MeOH 9/1 mixture (3 x 20 mL). The crude product was purified by column chromatography using a gradient of 0-10% MeOH in DCM, followed by purification by Isolute SCX-II chromatography eluting with MeOH/NH_3 to afford the title compound as a white amorphous solid (0.431 g, 35%). ^{1}H NMR (500 MHz, DMSO-d_6) δ 10.32 (s, 1H), 10.27 (s, 1H), 8.65 (d, *J* = 2.0 Hz, 1H), 8.49 (d, *J* = 8.4 Hz, 1H), 8.26 (dd, *J* = 8.8, 2.0 Hz, 1H), 8.20 – 8.04 (m, 2H), 7.80 – 7.66 (m, 2H), 7.62 – 7.46 (m, 3H), 7.00 (d, *J* = 8.4 Hz, 1H), 4.39 – 4.23 (m, 4H), 3.79 (s, 2H), 2.51 – 2.29 (m, 10H), 0.98 (t, *J* = 7.2 Hz, 3H). ^{13}C NMR (126 MHz, DMSO-d_6) δ 165.45, 165.11, 162.24, 148.74, 147.03, 143.43, 139.05, 138.01, 135.34, 131.83, 129.82, 129.29, 128.88, 128.36, 127.77, 126.70, 124.02, 122.32, 121.79, 120.29, 119.71, 117.38, 117.21, 64.88, 64.49, 54.09, 53.86, 48.38, 18.71. HRMS (ESI+): calcd for $C_{32}H_{33}{}^{35}ClN_5O_4$ (M + H)+, 586.2216; found 586.2189.

2-((4-(*tert*-Butyl)piperazin-1-yl)methyl)-*N*-(2-chloro-5-(2,3-dihydrobenzo[*b*][1,4]dioxine-6-carboxamido)phenyl)quinoline-6-carboxamide **S36**

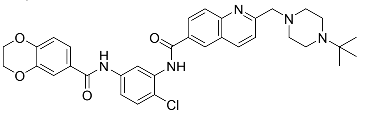

A solution of *N*-(2-chloro-5-(2,3-dihydrobenzo[*b*][1,4]dioxine-6-carboxamido)phenyl)-2-formylquinoline-6-carboxamide (300 mg, 0.615 mmol) and 1-(*tert*-butyl)piperazine (262 mg, 1.845 mmol) in anhydrous DCM (5 mL) and was allowed to stir at 20 °C for 12 h, after which sodium triacetoxyborohydride (391 mg, 1.845 mmol) was added in one portion and the resulting mixture was allowed to stir at 20 °C for 2 h. The reaction was quenched with $NaHCO_3$ saturated aqueous solution (5 mL) and extracted with a DCM/MeOH 9/1 mixture (3 x 5 mL). The crude product (pale yellow solid) was purified by column chromatography using a gradient of 0-10% MeOH in DCM followed by wash in water and trituration in diethyl ether to afford the title compound as a beige amorphous solid (60 mg, 16%). ^{1}H-NMR (500 MHz, DMSO-d_6): δ 10.34 (s, 1H), 10.29 (s, 1H), 8.66 (d, *J* = 1.56 Hz, 1H), 8.50 (d, *J* = 8.60 Hz, 1H), 8.26 (dd, *J* = 8.60, 2.35 Hz, 1H), 8.14 (d, *J* = 2.35 Hz, 1H), 8.09 (d, *J* = 8.60 Hz, 1H), 7.77 – 7.72 (m, 2H), 7.56 (d, *J* = 2.35 Hz, 1H), 7.54 (d, *J* = 1.56 Hz, 1H), 7.53 (dd, *J* = 7.82, 2.35 Hz, 1H), 7.00 (d, *J* = 8.60 Hz, 1H), 4.35 – 4.27 (m, 4H), 3.79 (s, 2H), 2.79 – 2.29 (m, 8H), 1.03 (br s, 9H). ^{13}C NMR (126 MHz, DMSO-d_6) δ 165.46, 165.11, 162.21, 148.75, 147.03, 143.43, 139.05, 137.95, 135.35, 131.79, 129.82, 129.28, 128.86, 128.33, 127.77, 126.69, 124.00, 122.29, 121.79, 120.27, 119.69, 117.38, 117.21, 64.88, 64.76, 64.49, 54.13, 45.77, 26.05. HRMS (ESI+): calcd for $C_{34}H_{37}{}^{35}ClN_5O_4$ (M + H)+, 614.2529; found 614.2502.

N-(2-Chloro-5-(2,3-dihydrobenzo[*b*][1,4]dioxine-6-carboxamido)phenyl)-2-((4-ethyl-1,4-diazepan-1-yl)methyl)quinoline-6-carboxamide **S37**

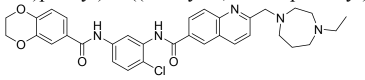

A suspension of *N*-(2-chloro-5-(2,3-dihydrobenzo[*b*][1,4]dioxine-6-carboxamido)phenyl)-2-formylquinoline-6-carboxamide (103 mg, 0.211 mmol) and 1-ethyl-1,4-diazepane (0.12 mL, 108 mg, 0.844 mmol) in anhydrous DCM (4.5 mL – sonication and a large volume of DCM were used in an attempt to fully solubilize the starting material) was allowed to stir at room temperature overnight. Then, sodium triacetoxyborohydride (179 mg, 0.844 mmol) was added and the reaction mixture stirred at room temperature for 40 h. The reaction was quenched with saturated aqueous $NaHCO_3$ solution. The aqueous layer was extracted three times with mixture of 10% MeOH in DCM, the combined organic layer was dried ($MgSO_4$) and concentrated *in vacuo*. This crude product was purified by Biotage chromatography using a gradient of 0-10% MeOH in DCM with a KPNH_2 column to afford a yellow oil. This material was further purified by Isolute SCX-II column chromatography (eluting with MeOH, followed by 10% 2M NH_3 in MeOH) to give a yellow gum. Finally, the gum was triturated with diethyl ether to afford a yellow amorphous solid (9 mg, 7%). ^{1}H NMR (500 MHz, DMSO-d_6) δ 10.31 (s, 1H), 10.26 (s, 1H), 8.65 (s, 1H), 8.49 (d, *J* = 8.5 Hz, 1H), 8.25 (d, *J* = 8.8 Hz, 1H), 8.14 (d, *J* = 2.0 Hz, 2H), 8.08 (d, *J* = 8.6 Hz, 1H), 7.75 (dd, *J* = 8.9, 2.2 Hz 1H), 7.57 – 7.48 (m, 3H), 7.00 (d, *J* = 8.4 Hz, 1H), 4.36 – 4.26 (m, 4H), 3.94 (s, 2H), 2.78 – 2.60 (m, 6H), 2.55 – 2.45 (m, 2H – hidden under DMSO peak, observed by HSQC), 1.75 (p, *J* = 5.8 Hz, 4H), 0.99 (t, *J* = 7.0 Hz, 3H). ^{13}C NMR (126 MHz, DMSO-d_6) δ 165.47, 165.11, 163.34, 148.72, 147.03, 143.43, 139.04, 137.91, 135.36, 131.73, 129.82, 129.26, 128.84, 128.29, 127.77, 126.67, 124.00, 122.20, 121.79, 120.27, 119.68, 117.38, 117.21, 64.88, 64.71, 64.49, 55.71, 54.81, 54.60, 53.77, 51.92, 27.75, 13.03. HRMS (ESI+): calcd for $C_{33}H_{35}{}^{35}ClN_5O_4$ (M + H)+ 600.2372, found 600.2351.

N-(2-Chloro-5-(2,3-dihydrobenzo[*b*][1,4]dioxine-6-carboxamido)phenyl)-2-((4-cyclopropylpiperazin-1-yl)methyl)quinoline-6-carboxamide **S38**

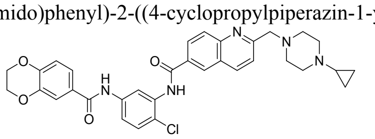

A solution of *N*-(2-chloro-5-(2,3-dihydrobenzo[*b*][1,4]dioxine-6-carboxamido)phenyl)-2-formylquinoline-6-carboxamide (255 mg, 0.523 mmol) and 1-cyclopropylpiperazine (0.189 mL, 1.568 mmol) in anhydrous DCM (5 mL) was allowed to stir at 20 °C for 12 h, after which sodium triacetoxyborohydride (332 mg, 1.568 mmol) was added in one portion and the resulting mixture was allowed to stir at 20 °C for 2 h. The reaction was quenched with $NaHCO_3$ saturated aqueous solution (5 mL) and extracted with a DCM/MeOH 9/1 mixture (3 x 5 mL). The crude product (brown solid) was purified by two rounds of column chromatography using a gradient of 0-10% MeOH in DCM followed by trituration in diethyl ether to afford the title compound as a pale yellow amorphous solid (44 mg, 14%). ^{1}H-NMR (500 MHz, DMSO-*d$_6$*): δ 10.32 (s, 1H), 10.27 (s, 1H), 8.65 (s, 1H), 8.50 (d, *J* = 7.87 Hz, 1H), 8.26 (d, *J* = 7.87 Hz, 1H), 8.15 (d, *J* = 1.83 Hz, 1H), 8.09 (d, *J* = 7.87 Hz, 1H), 7.77 – 7.71 (m, 2H), 7.57 – 7.49 (m, 3H), 7.00 (d, *J* = 7.87 Hz, 1H), 4.36 – 4.24 (m, 4H), 3.78 (s, 2H), 2.57 (br s, 4H), 2.43 (br s, 4H), 1.63 – 1.58 (m, 1H), 0.42 – 0.37 (m, 2H), 0.30 – 0.24 (m, 2H). ^{13}C NMR (126 MHz, DMSO-*d$_6$*) δ 165.47, 165.12, 162.22, 148.75, 147.05, 143.45, 139.06, 138.01, 135.36, 131.81, 129.84, 129.30, 128.87, 128.34, 127.78, 126.70, 124.02, 122.31, 121.80, 120.29, 119.70, 117.39, 117.22, 64.88, 64.49, 53.46, 53.25, 30.89, 6.09. HRMS (ESI$^+$): calcd for $C_{33}H_{33}{}^{35}ClN_5O_4$ (M + H)$^+$, 598.2216; found 598.2210.

2-((4-(*sec*-Butyl)piperazin-1-yl)methyl)-*N*-(2-chloro-5-(2,3-dihydrobenzo[*b*][1,4]dioxine-6-carboxamido)phenyl)quinoline-6-carboxamide **S39**

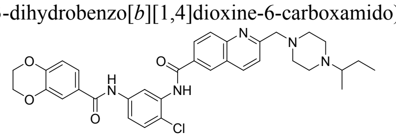

A solution of *N*-(2-chloro-5-(2,3-dihydrobenzo[*b*][1,4]dioxine-6-carboxamido)phenyl)-2-formylquinoline-6-carboxamide (255 mg, 0.523 mmol) and 1-(*sec*-butyl)piperazine (223 mg, 1.57 mmol) in anhydrous DCM (5 mL) and was allowed to stir at 20 °C for 12 h, after which sodium triacetoxyborohydride (332 mg, 1.57 mmol) was added in one portion and the resulting mixture was allowed to stir at 20 °C for 2 h. The reaction was quenched with $NaHCO_3$ saturated aqueous solution (5 mL) and extracted with a DCM/MeOH 9/1 mixture (3 x 5 mL). The crude product (brown solid) was purified by two rounds of column chromatography using a gradient of 0-10% MeOH in DCM, followed by trituration in diethyl ether to afford the title compound as a pale yellow amorphous solid (35 mg, 11%). ^{1}H-NMR (500 MHz, DMSO-*d$_6$*): δ 10.32 (s, 1H), 10.27 (s, 1H), 8.66 (d, *J* = 1.6 Hz, 1H), 8.50 (d, *J* = 8.4 Hz, 1H), 8.26 (dd, *J* = 8.4, 1.60 Hz, 1H), 8.15 (d, *J* = 2.3 Hz, 1H), 8.09 (d, *J* = 8.4 Hz, 1H), 7.75 (dd, *J* = 8.4, 2.36 Hz, 1H), 7.74 (d, *J* = 8.4 Hz, 1H), 7.55 (d, *J* = 2.3 Hz, 1H), 7.54 (d, *J* = 1.6 Hz, 1H), 7.52 (dd, *J* = 7.8, 2.3 Hz, 1H), 7.00 (d, *J* = 8.4 Hz, 1H), 4.35 – 4.27 (m, 4H), 3.81 (s, 2H), 2.87 – 2.17 (m, 9H), 1.51 (br s, 1H), 1.28 (br s, 1H), 0.95 (br s, 3H), 0.85 (t, *J* = 7.65 Hz, 3H). ^{13}C NMR (126 MHz, DMSO-*d$_6$*) δ 165.46, 165.11, 162.20, 148.75, 147.04, 143.44, 139.05, 137.98, 135.35, 131.80, 129.83, 129.29, 128.88, 128.34, 127.77, 126.69, 124.01, 122.30, 121.80, 120.28, 119.70, 117.39, 117.22, 64.89, 64.49, 60.25, 53.91, 48.15, 26.05, 14.08, 11.51. HRMS (ESI$^+$): calcd for $C_{34}H_{37}{}^{35}ClN_5O_4$ (M + H)$^+$, 614.2529; found 614.2495.

(*R*)-*N*-(2-Chloro-5-(2,3-dihydrobenzo[*b*][1,4]dioxine-6-carboxamido)phenyl)-2-((4-ethyl-2-methylpiperazin-1-yl)methyl)quinoline-6-carboxamide **S40**

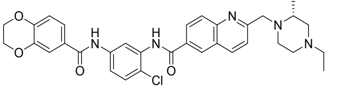

A suspension of *N*-(2-chloro-5-(2,3-dihydrobenzo[*b*][1,4]dioxine-6-carboxamido)phenyl)-2-formylquinoline-6-carboxamide (1.21 g, 2.48 mmol) and (*R*)-*tert*-butyl 3-methylpiperazine-1-carboxylate (0.993 g, 4.96 mmol) in anhydrous DCM (24 mL) was allowed to stir at room temperature overnight. Then, sodium triacetoxyborohydride (1.05 mg, 4.96 mmol) was added and the reaction mixture stirred at room temperature overnight. The reaction was quenched with saturated aqueous $NaHCO_3$ solution. The aqueous layer was extracted three times with mixture of 10% MeOH in DCM, the combined organic layer was dried (Na_2SO_4) and concentrated *in vacuo.* The crude product was purified by Biotage chromatography using a gradient of 0-10% MeOH in DCM to afford the product as a yellow semi-solid (560 mg, 34%). (*R*)-*tert*-butyl4-((6-((2-chloro-5-(2,3-dihydrobenzo[*b*][1,4]dioxine-6-carboxamido)phenyl)carbamoyl)quinolin-2-yl)methyl)-3-methylpiperazine-1-carboxylate (560 mg, 0.83 mmol) was taken up in anhydrous DCM (8 mL) and TFA (0.319 mL, 4.17 mmol) was added dropwise. The reaction was allowed to stir at room temperature for 48 h, after which time starting material was still observed by LCMS. Therefore, further TFA (0.319 mL, 4.17 mmol) was added and the reaction mixture left to stir for 18 h. The solvents were removed *in vacuo* and the resulting solid taken up in 10% MeOH in DCM. The organic layer was washed twice with saturated aqueous $NaHCO_3$ solution, followed by water, dried (Na_2SO_4), and then concentrated *in vacuo*. The material was partially purified by Isolute SCX-II column chromatography (eluting with MeOH, followed by 10% 2M NH_3 in MeOH) to afford the crude product as a yellow solid (272 mg, ~73% purity). To a solution of (*R*)-*N*-(2-chloro-5-(2,3-dihydrobenzo[*b*][1,4]dioxine-6-carboxamido)phenyl)-2-((2-methylpiperazin-1-yl)methyl)quinoline-6-carboxamide (100 mg, 0.128 mmol) in anhydrous MeOH (1.5 mL) at 0 °C was added sodium cyanoborohydride (8.82 mg, 0.140 mmol) in one portion. Acetaldehyde (5.02 µl, 0.089 mmol) was then added as a cooled solution (0 °C) in MeOH (0.5 ml) dropwise over 2 min. The reaction was allowed to warm slowly to room temperature and left to stir for 48 h. The MeOH was removed *in vacuo* and the resulting residue taken up in 10% MeOH in DCM. The organic layer was washed with 1M NaOH aqueous solution and dried (Na_2SO_4). The resulting residue was purified by Biotage chromatography using a gradient of 0-10% MeOH in DCM and then further purified by Isolute SCX-II chromatography (eluting with MeOH, followed by 10% 2M NH_3 in MeOH) to afford the product as a yellow amorphous solid (33 mg, 43%). ^{1}H NMR (500 MHz, Chloroform-*d*) δ 8.63 (s, 1H), 8.60 (d, *J* = 2.5 Hz, 1H), 8.41 (d, *J* = 1.8 Hz, 1H), 8.26 (d, *J* = 8.5 Hz, 1H), 8.19 (d, *J* = 8.8 Hz, 1H), 8.16 (dd, *J* = 8.8, 2.0 Hz, 1H), 8.07 (s, 1H), 7.99 (dd, *J* = 8.8, 2.5 Hz, 1H), 7.81 (d, *J* = 8.5 Hz, 1H), 7.46 (d, *J* = 1.8 Hz, 1H), 7.45 (d, *J* = 4.8 Hz, 1H), 7.39 (dd, *J* = 8.4, 2.2 Hz, 1H), 6.95 (d, *J* = 8.4 Hz, 1H), 4.35 (d, *J* = 14.5 Hz, 1H), 4.34 – 4.29 (m, 4H), 3.63 (d, *J* = 14.6 Hz, 1H), 2.83 (br d, *J* = 10.9 Hz, 1H), 2.81 – 2.75 (m, 2H), 2.72 – 2.63 (m, 1H), 2.53 – 2.37 (m, 3H), 2.21 – 2.14 (m, 1H), 2.01 (br t, *J* = 10.5 Hz, 1H), 1.19 (d, *J* = 6.2 Hz, 3H), 1.11 (t, *J* = 7.2 Hz, 3H). ^{13}C NMR (126 MHz, $CDCl_3$) δ 165.05, 164.92, 163.62, 149.06, 146.96, 143.58, 137.94, 137.18, 134.67, 131.65, 130.02, 129.51, 127.79, 127.74, 126.72, 122.27, 120.48, 117.81, 117.46, 116.89, 116.79, 112.45, 77.23, 64.58, 64.19, 60.73, 60.66, 55.85, 53.00, 52.58, 52.26, 17.84, 11.91. HRMS (ESI$^+$): calcd for $C_{33}H_{35}{}^{35}ClN_5O_4$ (M + H)$^+$ 600.2327, found 600.2352.

2-(Azetidin-1-ylmethyl)-*N*-(2-chloro-5-(2,3-dihydrobenzo[*b*][1,4]dioxine-6-carboxamido)phenyl)quinoline-6-carboxamide **S41**

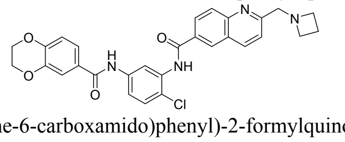

A solution of *N*-(2-chloro-5-(2,3-dihydrobenzo[*b*][1,4]dioxine-6-carboxamido)phenyl)-2-formylquinoline-6-carboxamide (300 mg, 0.615 mmol) and azetidine (0.124 mL, 1.84 mmol) in anhydrous DCM (2.0 mL) was allowed to stir at 20 °C for 12 h, after which sodium triacetoxyborohydride (391 mg, 1.84 mmol) was added in one portion and the resulting mixture was allowed to stir at 20 °C for 2 h. The reaction mixture was quenched with $NaHCO_3$ saturated aqueous solution (2 mL) and extracted with a mixture of DCM/MeOH 9/1 (3 x 2 mL). The crude product was purified by column chromatography on silica gel using a gradient of 0-15% MeOH in DCM, then washed with water and triturated with diethyl ether to afford the title compound as a pale yellow amorphous solid (20 mg, 6%). ^{1}H-NMR (500 MHz, DMSO-*d$_6$*): δ 10.35 (s, 1H), 10.31 (s, 1H), 8.66 (d, *J* = 2.2 Hz, 1H), 8.49 (d, *J* = 8.7 Hz, 1H), 8.27 (dd, *J* = 8.7, 2.2 Hz, 1H), 8.14 (d, *J* = 2.2 Hz, 1H), 8.08 (d, *J* = 8.7 Hz, 1H), 7.76 (dd, *J* = 8.7, 2.2 Hz, 1H), 7.65 (d, *J* = 7.6 Hz, 1H), 7.58 – 7.51 (m, 3H), 6.99 (d, *J* = 8.7 Hz, 1H), 4.35 – 4.27 (m, 4H), 3.91 (s, 2H), 3.39 – 3.27 (m, 4H), 2.06 (qn, *J* = 7.10 Hz, 2H). ^{13}C NMR (126 MHz, DMSO-*d$_6$*) δ 165.47, 165.11, 161.47, 148.75, 147.04, 143.43, 139.05, 138.09, 135.35, 131.80, 129.83, 129.27, 128.90, 128.41, 127.77, 126.62, 124.02, 121.91, 121.80, 120.28, 119.70, 117.39, 117.22, 65.18, 64.89, 64.49, 55.42, 49.06, 17.92. HRMS (ESI$^+$): calcd for $C_{29}H_{26}{}^{35}ClN_4O_4$ (M + H)$^+$, 529.1637; found 529.1616.

N-(2-Chloro-5-(2,3-dihydrobenzo[*b*][1,4]dioxine-6-carboxamido)phenyl)-2-(pyrrolidin-1-ylmethyl)quinoline-6-carboxamide **S42**

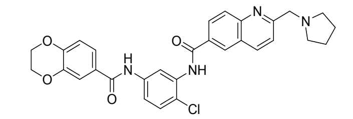

A solution of *N*-(2-chloro-5-(2,3-dihydrobenzo[*b*][1,4]dioxine-6-carboxamido)phenyl)-2-formylquinoline-6-carboxamide (300 mg, 0.615 mmol) and pyrrolidine (131 mg, 1.84 mmol) in anhydrous DCM (2 mL) was allowed to stir at 20 °C for 12 h, after which sodium triacetoxyborohydride (391 mg, 1.84 mmol) was added in one portion and the resulting mixture was allowed to stir at 20 °C for 2 h. The resulting mixture was quenched with $NaHCO_3$ saturated aqueous solution (2 mL) and extracted with a mixture of DCM/MeOH 9/1 (3 x 2 mL). The crude product was purified by column chromatography using a gradient of 0-15% MeOH in DCM, then washed with water and triturated with diethyl ether to afford the title compound as a pale yellow amorphous solid (20 mg, 6%). ^{1}H-NMR (500 MHz, DMSO-d_6): δ 10.32 (s, 1H), 10.27 (s, 1H), 8.65 (s, 1H), 8.49 (d, *J* = 7.98 Hz, 1H), 8.26 (dd, *J* = 7.98, 2.28 Hz, 1H), 8.14 (d, *J* = 2.28 Hz, 1H), 8.09 (d, *J* = 7.98 Hz, 1H), 7.77 – 7.70 (m, 2H), 7.56 – 7.50 (m, 3H), 7.00 (d, *J* = 7.98 Hz, 1H), 4.35 – 4.28 (m, 4H), 3.92 (s, 2H), 2.57 – 2.52 (m, 4H), 1.77 – 1.70 (m, 4H). ^{13}C NMR (126 MHz, DMSO-d_6) δ 165.49, 165.11, 162.75, 148.72, 147.04, 143.44, 139.05, 137.98, 135.36, 131.77, 129.83, 129.30, 128.86, 128.33, 127.77, 126.66, 124.01, 122.29, 121.79, 120.27, 119.69, 117.39, 117.22, 64.89, 64.49, 62.45, 54.29, 23.77. HRMS (ESI$^+$): calcd for $C_{30}H_{28}{}^{35}ClN_4O_4$ (M + H)$^+$, 543.1794; found 543.1778.

N-(2-Chloro-5-(2,3-dihydrobenzo[*b*][1,4]dioxine-6-carboxamido)phenyl)-2-((3-methylazetidin-1-yl)methyl)quinoline-6-carboxamide **S43**

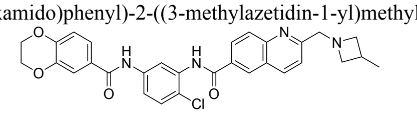

A solution of 3-methylazetidine hydrochloride (33.1 mg, 0.307 mmol) in anhydrous DCM (0.5 mL) was added to a stirring solution of *N*-(2-chloro-5-(2,3-dihydrobenzo[*b*][1,4]dioxine-6-carboxamido)phenyl)-2-formylquinoline-6-carboxamide (50 mg, 0.102 mmol) in anhydrous DCM (1.5 mL) at room temperature .The reaction was stirred for 5.5 h, then $NaBH(OAc)_3$ (65.2 mg, 0.307 mmol) was added and the reaction mixture was stirred for 48 h. The reaction was quenched with saturated aqueous $NaHCO_3$ solution. The aqueous layer was extracted with 3 x 10% MeOH in DCM. The combined organic layer was dried (Na_2SO_4) and concentrated *in vacuo*. The residue was purified by Biotage chromatography using a gradient of 0-10% MeOH in DCM to afford a yellow gum. This gum was further purified by Isolute SCX-II chromatography (eluting with MeOH then 10% 2M NH_3 in MeOH) to afford the title product as a yellow amorphous solid (16.5 mg, 30%). ^{1}H NMR (500 MHz, Chloroform-*d*) δ 8.63 (br s, 1H), 8.58 (d, *J* = 2.5 Hz, 1H), 8.41 (d, *J* = 1.8 Hz, 1H), 8.28 (d, *J* = 8.5 Hz, 1H), 8.20 (d, *J* = 8.8 Hz, 1H), 8.16 (dd, *J* = 8.8, 2.0 Hz, 1H), 7.98 (dd, *J* = 8.8, 2.4 Hz, 2H), 7.64 (d, *J* = 8.5 Hz, 1H), 7.46 (s, 1H), 7.45 (d, *J* = 7.2 Hz, 1H), 7.39 (dd, *J* = 8.4, 2.2 Hz, 1H), 6.96 (d, *J* = 8.4 Hz, 1H), 4.38 – 4.29 (m, 4H), 3.99 (s, 2H), 3.64 (t, *J* = 7.5 Hz, 2H), 2.95 (t, *J* = 7.2 Hz, 2H), 2.70 (dt, *J* = 14.0, 7.0 Hz, 1H), 1.21 (d, *J* = 6.8 Hz, 3H). HRMS (ESI$^+$): calcd for $C_{30}H_{28}{}^{35}ClN_4O_4$ (M + H)$^+$ 543.1794, found 543.1769.

N-(2-Chloro-5-(2,3-dihydrobenzo[*b*][1,4]dioxine-6-carboxamido)phenyl)-2-((3,3-dimethylazetidin-1-yl)methyl)quinoline-6-carboxamide **S44**

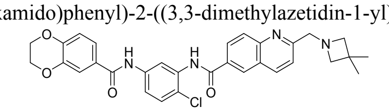

3,3-Dimethylazetidine (55.0 mg, 0.646 mmol) was added to a stirring solution of *N*-(2-chloro-5-(2,3-dihydrobenzo[*b*][1,4]dioxine-6-carboxamido)phenyl)-2-formylquinoline-6-carboxamide (210 mg, 0.431mmol) in anhydrous DCM (1.5 mL) at room temperature under an inert atmosphere. The reaction was stirred for 1 h. Then, $NaBH(OAc)_3$ (137 mg, 0.646 mmol) was added and the reaction mixture was stirred for 18 h. The reaction was quenched with saturated aqueous $NaHCO_3$ solution (4 mL). The aqueous layer was extracted with DCM (3 x 4 mL), the combined organic layer was dried (Na_2SO_4) and concentrated *in vacuo*. The residue was purified by column chromatography using a gradient of 0-10% MeOH in DCM to afford a yellow gum. This gum was further purified by Isolute SCX-II chromatography (eluting with MeOH then 10% NH_3 in MeOH) to afford the title product as a yellow amorphous solid (16.0 mg, 7%). ^{1}H NMR (500 MHz, DMSO-d_6) δ 10.35 (s, 1H), 10.27 (s, 1H), 8.70 – 8.66 (m, 1H), 8.55 (d, *J* = 8.5 Hz, 1H), 8.29 (dd, *J* = 9.0, 2.0 Hz, 1H), 8.17 – 8.09 (m, 2H), 7.74 (dd, *J* = 8.8, 2.6 Hz, 1H), 7.65 (d, *J* = 8.5 Hz, 1H), 7.57 – 7.49 (m, 3H), 7.00 (d, *J* = 8.4 Hz, 1H), 4.36 – 4.27 (m, 4H), 3.45 – 3.20 (m, 6H), 1.27 (s, 6H). δ HRMS (ESI$^+$): calcd for $C_{31}H_{30}{}^{37}ClN_4O_4$ (M + H)$^+$ 559.1937, found 559.1921.

2-(Azetidin-1-ylmethyl)-*N*-(5-(2,3-dihydrobenzo[*b*][1,4]dioxine-6-carboxamido)-2-fluorophenyl)quinoline-6-carboxamide **S45**

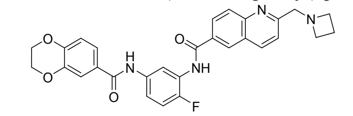

To a solution of *N*-(5-(2,3-dihydrobenzo[*b*][1,4]dioxine-6-carboxamido)-2-fluorophenyl)-2-formylquinoline-6-carboxamide (100 mg, 0.212 mmol) in anhydrous DCM (2 mL), azetidine (43 µL, 0.636 mmol) was added dropwise at room temperature and the resulting mixture was allowed to stir 12 h. Then sodium triacetoxyborohydride (135 mg, 0.636 mmol) was added in one portion and the resulting mixture was allowed to stir for 2 h at room temperature. The reaction mixture was washed with brine (2 mL) and purified by column chromatography using a gradient of 0-10% MeOH in DCM + 1% 7N NH_3 in MeOH to afford the title compound as a white amorphous solid (10 mg, 9%).. ^{1}H NMR (500 MHz, MeOD) δ 8.59 (d, *J* = 2.1 Hz, 1H), 8.48 (d, *J* = 8.5 Hz, 1H), 8.29 (dd, *J* = 8.8, 2.1 Hz, 1H), 8.24 – 8.10 (m, 2H), 7.71 – 7.55 (m, 2H), 7.55 – 7.44 (m, 2H), 7.24 (dd, *J* = 10.1, 8.9 Hz, 1H), 6.96 (d, *J* = 8.4 Hz, 1H), 4.38 – 4.26 (m, 4H), 3.99 (s, 2H), 3.47 (t, *J* = 7.1 Hz, 4H), 2.21 (qn, *J* = 7.2 Hz, 2H). ^{13}C NMR (126 MHz, MeOD) δ 166.69, 166.60, 160.42, 152.36 (d, *J* = 248.54 Hz), 148.53, 147.10, 143.47, 138.18, 134.84 (d, *J* = 2.22 Hz), 131.94, 128.36, 128.18, 127.96, 127.38, 126.66, 125.28 (d, *J* = 11.11 Hz), 121.58, 120.75, 119.56 (d, *J* = 6.67 Hz), 118.95, 116.79, 116.64, 115.23 (d, *J* = 20.01 Hz), 64.53, 64.13, 55.13, 29.36, 17.33. HRMS (ESI$^+$): calcd for $C_{29}H_{26}FN_4O_4$ (M + H)$^+$,513.1933; found513.1930.

N-(5-(2,3-Dihydrobenzo[*b*][1,4]dioxine-6-carboxamido)-2-fluorophenyl)-2-(pyrrolidin-1-ylmethyl)quinoline-6-carboxamide **S46**

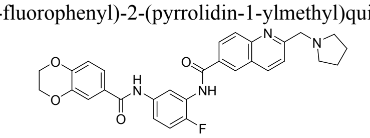

To a solution of *N*-(5-(2,3-dihydrobenzo[*b*][1,4]dioxine-6-carboxamido)-2-fluorophenyl)-2-formylquinoline-6-carboxamide (100 mg, 0.212 mmol) in anhydrous DCM (2 mL), pyrrolidine (53 µL, 0.636 mmol) was added dropwise at room temperature and the resulting mixture was allowed to stir for 12 h. Then sodium triacetoxyborohydride (135 mg, 0.636 mmol) was added in one portion and the resulting mixture was allowed to stir for 2 h at room temperature. The reaction mixture was washed with brine (2 mL) and purified by column chromatography using a gradient of 0-10% MeOH in DCM + 1% 7N NH_3 in MeOH to afford the title compound as a pale yellow amorphous solid (55 mg, 49%). ^{1}H NMR (500 MHz, DMSO-d_6) δ 10.39 (s, 1H), 10.18 (s, 1H), 8.64 (d, *J* = 1.8 Hz, 1H), 8.48 (d, *J* = 8.5 Hz, 1H), 8.24 (dd, *J* = 8.8, 1.9 Hz, 1H), 8.14 (dd, *J* = 7.0, 2.6 Hz, 1H), 8.08 (d, *J* = 8.8 Hz, 1H), 7.72 (d, *J* = 8.5 Hz, 1H), 7.66 (ddd, *J* = 8.9, 4.1, 2.8 Hz, 1H), 7.57 – 7.49 (m, 2H), 7.30 (t, *J* = 9.5 Hz, 1H), 6.99 (d, *J* = 8.4 Hz, 1H), 4.31 (q, *J* = 5.1 Hz, 4H), 3.92 (s, 2H), 2.50 (br s, 4H), 1.75 (br s, 4H). ^{13}C NMR (126 MHz, DMSO-d_6) δ 165.52, 164.96, 162.79, 152.32 (d, *J* = 245.73 Hz), 148.72, 146.93, 143.43, 137.98, 135.93 (d, *J* = 2.12 Hz), 131.72, 129.23, 128.97, 128.44, 127.93, 126.62, 125.83 (d, *J* = 13.69 Hz), 122.28, 121.73, 119.27, 119.17 (d, *J* = 7.12 Hz), 117.36, 117.16, 116.03 (d, *J* = 20.87 Hz), 64.88, 64.50, 62.47, 54.29, 23.77. HRMS (ESI$^+$): calcd for $C_{30}H_{28}FN_4O_4$ (M + H)$^+$, 527.2089; found 527.2074.

N-(5-(2,3-Dihydrobenzo[*b*][1,4]dioxine-6-carboxamido)-2-fluorophenyl)-2-(piperidin-1-ylmethyl)quinoline-6-carboxamide **S47**

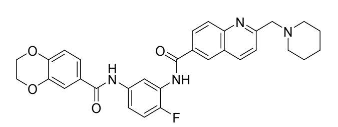

To a solution of *N*-(5-(2,3-dihydrobenzo[*b*][1,4]dioxine-6-carboxamido)-2-fluorophenyl)-2-formylquinoline-6-carboxamide (100 mg, 0.212 mmol) in anhydrous DCM (2 mL), piperidine (63 µL, 0.636 mmol) was added dropwise at room temperature and the resulting mixture was allowed to stir under an inert argon atmosphere for 7 h. Then sodium triacetoxyborohydride (135 mg, 0.636 mmol) was added in one portion and the resulting mixture was allowed to stir for 2 h at room temperature. The reaction mixture was washed with brine (2 mL) and purified by column chromatography using a gradient of 0-10% MeOH in DCM + 1% 7N NH_3 in MeOH to afford the title compound as a pale yellow amorphous solid (50 mg, 44%). ^{1}H NMR (500 MHz, DMSO-d_6) δ 10.39 (s, 1H), 10.18 (s, 1H), 8.64 (s, 1H), 8.48 (d, J = 8.6 Hz, 1H), 8.24 (d, J = 8.7 Hz, 1H), 8.11 (dd, J = 30.3, 9.0 Hz, 2H), 7.74 (d, J = 8.7 Hz, 1H), 7.67 – 7.59 (m, 1H), 7.58 – 7.46 (m, 2H), 7.30 (t, J = 9.8 Hz, 1H), 6.99 (d, J = 9.5 Hz, 1H), 4.31 (d, J = 5.6 Hz, 4H), 3.75 (s, 2H), 2.42 (br s, 4H), 1.53 (br s, 4H), 1.42 (br s, 2H). ^{13}C NMR (126 MHz, DMSO-d_6) δ 165.49, 164.94, 162.63, 152.34 (d, J = 248.13 Hz), 148.72, 146.91, 143.41, 137.92, 135.89 (d, J = 2.18 Hz), 131.68, 129.18, 128.96, 128.41, 127.92, 126.62, 125.81 (d, J = 10.90 Hz), 122.19, 121.71, 119.35, 119.18 (d, J = 8.17 Hz), 117.35, 117.15, 116.03 (d, J = 21.79 Hz), 65.65, 64.87, 64.49, 54.79, 26.09, 24.29. HRMS (ESI$^+$): calcd for $C_{31}H_{30}FN_4O_4$ (M + H)$^+$, 541.2246; found 541.2242.

N-(5-(2,3-Dihydrobenzo[*b*][1,4]dioxine-6-carboxamido)-2-fluorophenyl)-2-((2-methylpyrrolidin-1-yl)methyl)quinoline-6-carboxamide **S48**

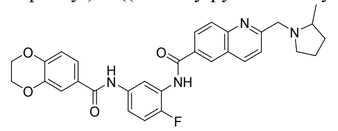

2-Fluoro-5-nitroaniline (31.7 mg, 0.203 mmol), 2-((2-methylpyrrolidin-1-yl)methyl)quinoline-6-carboxylic acid hydrochloride (62.4 mg, 0.203 mmol) and EDC (78 mg, 0.406 mmol) were dissolved in anhydrous DMF (2 mL) and pyridine (0.082 mL, 1.02 mmol) was added dropwise. The mixture was allowed to stir at 20 °C for 17 h. The reaction mixture was washed with $NaHCO_3$ saturated aqueous solution, extracted with DCM/MeOH 9/1 mixture and dried over Na_2SO_4. The crude product was purified by column chromatography using a gradient of 0-10% MeOH in DCM + 1% 7N NH_3 in MeOH to afford the product as a yellow solid which was carried directly onto the next step (83 mg). LC-MS (ESI$^+$): m/z=409.1655, (M + H)$^+$. *N*-(2-Fluoro-5-nitrophenyl)-2-((2-methylpyrrolidin-1-yl)methyl)quinoline-6-carboxamide (83 mg, 0.203 mmol), ammonium chloride (76 mg, 1.42 mmol) and iron powder (79 mg, 1.42 mmol) were combined and suspended in EtOH (3 mL) and water (1 mL) at room temperature, affording a beige suspension, which was heated at 90 °C for 1 h. The reaction mixture was cooled to room temperature, filtered through a pad of Celite® to remove the iron (eluting with EtOH/DCM/MeOH). The solvents were then removed *in vacuo*. The resulting residue was dried to afford a pale beige solid as crude product, which was taken onto the next step without purification (77 mg). LC-MS (ESI$^+$):, m/z=379.1918, (M + H)$^+$. *N*-(5-Amino-2-fluorophenyl)-2-((2-methylpyrrolidin-1-yl)methyl)quinoline-6-carboxamide (77 mg, 0.203 mmol), 2,3-dihydrobenzo[*b*][1,4]dioxine-6-carboxylic acid (36.7 mg, 0.203 mmol) and EDC (98 mg, 0.509 mmol) were dissolved in anhydrous DMF (1.5 mL), then pyridine (82 µL, 1.02 mmol) was added dropwise and the resulting mixture was allowed to stir at 20 °C for 72 h. The reaction mixture was washed with water (2 mL) and extracted with DCM/MeOH 9/1 mixture (3 x 5 mL) to afford a pale yellow solid as crude product, which was purified by flash column chromatography using a gradient of 0-10% MeOH in DCM + 1% 7N NH_3 in MeOH to afford a yellow solid as semi-crude product, which was then re-purified by semi-preparative TLC (10% MeOH in DCM) to afford the title compound as a pale yellow amorphous solid (30 mg, 27 %). ^{1}H NMR (500 MHz, MeOD) δ 8.58 (d, J = 1.9 Hz, 1H), 8.46 (d, J = 8.5 Hz, 1H), 8.27 (dd, J = 8.8, 2.0 Hz, 1H), 8.22 – 8.11 (m, 2H), 7.78 (d, J = 8.5 Hz, 1H), 7.58 (ddd, J = 8.9, 4.2, 2.7 Hz, 1H), 7.51 – 7.44 (m, 2H), 7.26 – 7.18 (m, 1H), 6.94 (d, J = 8.3 Hz, 1H), 4.37 – 4.26 (m, 5H), 3.63 (d, J = 13.6 Hz, 1H), 3.00 (ddd, J = 10.1, 7.6, 3.5 Hz, 1H), 2.64 (dq, J = 13.5, 6.2 Hz, 1H), 2.37 (q, J = 9.0 Hz, 1H), 2.11 – 2.00 (m, 1H), 1.77 (dtd, J = 12.5, 9.1, 7.9, 3.2 Hz, 2H), 1.52 (tdd, J = 9.9, 7.3, 4.6 Hz, 1H), 1.22 (d, J = 6.1 Hz, 3H). ^{13}C NMR (126 MHz, MeOD) δ 166.70, 166.61, 165.65, 162.27, 152.47 (d, J = 244.5 Hz), 148.38, 147.12, 143.49, 137.99, 134.88 (d, J = 2.5 Hz), 131.93, 128.19, 127.94, 127.40, 126.73, 125.31 (d, J = 12.4 Hz), 122.37, 120.76, 119.54 (d, J = 9.1 Hz), 118.93, 116.80, 116.65, 115.23 (d, J = 19.1 Hz), 64.53, 64.14, 60.40, 59.89, 54.17, 32.13, 21.14, 17.67. HRMS (ESI$^+$): calcd for $C_{31}H_{30}FN_4O_4$ (M + H)$^+$, 541.2246; found 541.2236.

N-(5-(2,3-Dihydrobenzo[*b*][1,4]dioxine-6-carboxamido)-2-fluorophenyl)-2-((3-methylazetidin-1-yl)methyl)quinoline-6-carboxamide **S49**

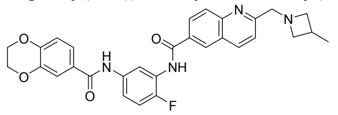

A solution of *N*-(5-(2,3-dihydrobenzo[*b*][1,4]dioxine-6-carboxamido)-2-fluorophenyl)-2-formylquinoline-6-carboxamide (0.25 g, 0.530 mmol) and 3-methylazetidine (113 mg, 1.591 mmol) in anhydrous DCM (4 mL) was allowed to stir at 20 °C for 18 h, after which sodium triacetoxyborohydride (0.337 g, 1.591 mmol) was added in one portion and the resulting mixture was allowed to stir at 20 °C for 3 h. The reaction was quenched with $NaHCO_3$ saturated aqueous solution and extracted with a mixture DCM/MeOH 9/1 (3 x 5 mL). Purification by column chromatography using a gradient of 0-10% MeOH in DCM , followed by further purification by Isolute SCX-II cartridge with MeOH/NH_3, afforded the title compound as a pale yellow amorphous solid (114 mg, 41%). ^{1}H NMR (500 MHz, DMSO-d_6) δ 10.46 (s, 1H), 10.20 (s, 1H), 8.71 (d, J = 1.7 Hz, 1H), 8.59 (d, J = 8.5 Hz, 1H), 8.32 (dd, J = 8.8, 1.9 Hz, 1H), 8.21 – 8.08 (m, 2H), 7.64 (ddd, J = 7.6, 4.3, 2.8 Hz, 2H), 7.58 – 7.44 (m, 2H), 7.38 – 7.23 (m, 1H), 6.99 (d, J = 8.4 Hz, 1H), 4.66 (s, 2H), 4.43 – 4.22 (m, 4H), 4.27 – 4.02 (m, 2H), 3.71 (s, 2H), 2.95 – 2.76 (m, 1H), 1.23 (d, J = 6.4 Hz, 3H). ^{13}C NMR (126 MHz, DMSO-d_6) δ 165.48, 164.95, 161.35, 152.37 (d, J = 244.0 Hz), 148.71, 146.91, 143.41, 138.17, 135.90 (d, J = 1.6 Hz), 131.77, 129.20, 129.02, 128.56, 127.93, 126.60, 125.80 (d, J = 14.0 Hz), 121.89, 121.72, 119.28, 119.23 (d, J = 7.8 Hz), 117.35, 117.17, 116.07 (d, J = 20.4 Hz), 66.85, 64.88, 64.49, 25.90, 21.53, 19.29. HRMS (ESI$^+$): calcd for $C_{30}H_{28}FN_4O_4$ (M + H)$^+$, 527.2068; found 527.2089.

N-(5-(2,3-Dihydrobenzo[*b*][1,4]dioxine-6-carboxamido)-2-fluorophenyl)-2-((3,3-dimethylazetidin-1-yl)methyl)quinoline-6-carboxamide **S50**

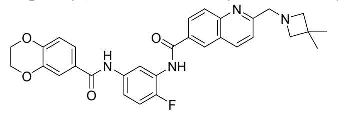

A solution of *N*-(5-(2,3-dihydrobenzo[*b*][1,4]dioxine-6-carboxamido)-2-fluorophenyl)-2-formylquinoline-6-carboxamide (0.25 g, 0.530 mmol) and 3,3-dimethylazetidine (135 mg, 1.59 mmol) in anhydrous DCM (4 mL) was allowed to stir at 20 °C for 18 h, after which sodium triacetoxyborohydride (0.337 g, 1.59 mmol) was added in one portion and the resulting mixture was allowed to stir at 20 °C for 3 h. The reaction was quenched with $NaHCO_3$ saturated aqueous solution and extracted with a mixture DCM/MeOH 9/1 (3 x 5 mL). Purification by column chromatography using a gradient of 0-10% MeOH in DCM , followed by further purification by Isolute SCX-II cartridge with MeOH/NH_3, afforded the title compound as a pale yellow solid (156 mg, 54%). ^{1}H NMR (500 MHz, DMSO-d_6) δ 10.40 (s, 1H), 10.19 (s, 1H), 8.66 (d, J = 2.0 Hz, 1H), 8.51 (d, J = 8.5 Hz, 1H), 8.26 (dd, J = 8.7, 2.1 Hz, 1H), 8.18 – 8.06 (m, 2H), 7.69 – 7.62 (m, 2H), 7.57 – 7.49 (m, 2H), 7.29 (dd, J = 10.1, 9.0 Hz, 1H), 6.99 (d, J = 8.4 Hz, 1H), 4.35 – 4.27 (m, 4H), 4.07 (br s, 2H), 3.22 (br s, 4H), 1.24 (s, 6H). ^{13}C NMR (126 MHz, DMSO-d_6) δ 165.46, 164.95, 161.71, 152.28 (d, J = 250.1 Hz), 148.68, 146.92, 143.41, 138.23, 135.90, 135.88 (d, J = 2.1 Hz), 131.83, 129.20, 129.03, 128.61, 127.92, 126.62, 125.80 (d, J = 15.0 Hz), 121.88, 121.72, 119.28, 119.21 (d, J = 6.0 Hz), 117.35, 117.16, 116.10 (d, J = 21.0 Hz), 66.80, 64.88, 64.49, 32.19, 27.33. HRMS (ESI$^+$): calcd for $C_{31}H_{30}FN_4O_4$ (M + H)$^+$,541.2246; found541.2240.

N-(5-(2,3-Dihydrobenzo[*b*][1,4]dioxine-6-carboxamido)-2-fluorophenyl)-2-(piperazin-1-ylmethyl)quinoline-6-carboxamide **S51**

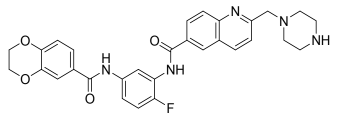

A solution of *N*-(5-(2,3-dihydrobenzo[*b*][1,4]dioxine-6-carboxamido)-2-fluorophenyl)-2-formylquinoline-6-carboxamide (255 mg, 0.541 mmol) and *tert*-butyl piperazine-1-carboxylate (302 mg, 1.62 mmol) in anhydrous DCM (5 mL) was allowed to stir at 20 °C for 12 h, after which sodium triacetoxyborohydride (344 mg, 1.62 mmol) was added in one portion and the resulting mixture was allowed to stir at 20 °C for 2 h. The reaction was quenched with $NaHCO_3$ saturated aqueous solution (5 mL) and extracted with a DCM/MeOH 9/1 mixture (3 x 5 mL). The crude product (pale yellow solid) was purified by column chromatography using a gradient of 0-6% MeOH in DCM, followed by trituration in diethyl ether to afford the desired product as a pale beige amorphous solid (325 mg, 94%). ^{1}H NMR (500 MHz, DMSO-*d$_6$)* δ 10.39 (s, 1H), 10.18 (s, 1H), 8.65 (d, *J* = 2.0 Hz, 1H), 8.50 (d, *J* = 8.6 Hz, 1H), 8.25 (dd, *J* = 8.8, 2.1 Hz, 1H), 8.19 – 8.06 (m, 2H), 7.75 (d, *J* = 8.5 Hz, 1H), 7.65 (ddd, *J* = 9.0, 4.3, 2.6 Hz, 1H), 7.57 – 7.49 (m, 2H), 7.30 (dd, *J* = 10.1, 9.0 Hz, 1H), 6.99 (d, *J* = 8.5 Hz, 1H), 4.35 – 4.27 (m, 4H), 3.82 (s, 2H), 3.33 (br s, 4H), 2.44 (br s, 4H), 1.40 (s, 9H). ^{13}C NMR (126 MHz, DMSO-*d$_6$)* δ 165.46, 164.94, 161.81, 154.27, 152.26 (d, *J* = 245.8 Hz), 148.76, 146.91, 143.41, 138.07, 135.89 (d, *J* = 2.9 Hz), 131.80, 129.23, 128.99, 128.49, 127.92, 126.66, 125.83 (d, *J* = 12.1 Hz), 122.30, 121.71, 119.27, 119.18 (d, *J* = 7.89 Hz), 117.35, 117.15, 116.02 (d, *J* = 21.0 Hz), 79.26, 64.87, 64.69, 64.49, 53.18, 28.52. To a suspension of *tert*-butyl 4-((6-((5-(2,3-dihydrobenzo[*b*][1,4]dioxine-6-carboxamido)-2-fluorophenyl)carbamoyl)quinolin-2-yl)methyl)piperazine-1-carboxylate (300 mg, 0.468 mmol) in anhydrous DCM (5 mL,), TFA (0.179 mL, 2.338 mmol) was added dropwise and the resulting mixture was allowed to stir at 20 °C for 3 h. The reaction mixture was concentrated under reduced pressure to afford the crude product as a light brown oil. The crude was purified by column chromatography using a gradient of 0-15% MeOH in DCM followed by trituration in diethyl ether to afford the title compound as an amorphous white solid (174 mg, 68.7%). ^{1}H-NMR (500 MHz, DMSO-*d$_6$*): δ 10.41 (s, 1H), 10.20 (s, 1H), 8.73 (br s, 1H), 8.67 (d, *J* = 1.7 Hz, 1H), 8.52 (d, *J* = 8.7 Hz, 1H), 8.27 (dd, *J* = 8.7, 1.7 Hz, 1H), 8.15 (dd, *J* = 7.1, 2.7 Hz, 1H), 8.09 (d, *J* = 8.7 Hz, 1H), 7.74 (d, *J* = 8.4 Hz, 1H), 7.66 – 7.61 (m, 1H), 7.54 (d, *J* = 2.1 Hz, 1H), 7.52 (dd, *J* = 8.4, 2.1 Hz, 1H), 7.30 (app t, *J* = 10.1 Hz, 1H), 6.99 (d, *J* = 8.4 Hz, 1H), 4.35 – 4.27 (m, 4H), 3.89 (s, 2H), 3.19 – 3.09 (m, 4H), 2.76 – 2.66 (m, 4H). ^{13}C NMR (126 MHz, DMSO-*d$_6$*) δ 164.98, 164.50, 160.59, 151.87 (d, *J* = 243.8 Hz), 148.27, 146.47, 142.96, 137.76, 135.45 (d, *J* = 2.7 Hz), 131.45, 128.79, 128.57, 128.13, 127.46, 126.25, 125.30 (d, *J* = 13.2 Hz), 121.95, 121.26, 118.86, 118.78 (d, *J* = 7.8 Hz), 116.90, 116.70, 115.59 (d, *J* = 20.9 Hz), 64.42, 64.04, 63.67, 49.45, 43.03. HRMS (ESI$^+$): calcd for $C_{30}H_{29}FN_5O_4$ $(M + H)^+$, 542.2198; found 542.2190.

N-(5-(2,3-Dihydrobenzo[*b*][1,4]dioxine-6-carboxamido)-2-fluorophenyl)-2-((4-methylpiperazin-1-yl)methyl)quinoline-6-carboxamide **S52**

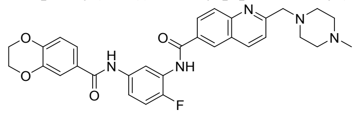

A solution of *N*-(5-(2,3-dihydrobenzo[*b*][1,4]dioxine-6-carboxamido)-2-fluorophenyl)-2-formylquinoline-6-carboxamide (5.15 g, 10.92 mmol) and 1-methylpiperazine (3.28 g, 32.8 mmol) in anhydrous DCM (90 mL) was allowed to stir at 20 °C for 18 h, after which sodium triacetoxyborohydride (6.95 g, 32.8 mmol) was added in one portion and the resulting mixture was allowed to stir at 20 °C for 1.5 h. The reaction was quenched with $NaHCO_3$ aqueous saturated solution (50 mL) and extracted with DCM/MeOH 9/1 mixture (3 x 50 mL). Purification by column chromatography on silica gel using a gradient of 0-20% MeOH in DCM, followed by wash in water and trituration in diethyl ether afforded the title compound as an amorphous white solid (2.85 g, 47%). ^{1}H-NMR (500 MHz, DMSO-*d$_6$*): δ 10.40 (s, 1H), 10.19 (s, 1H), 8.65 (d, *J* = 1.7 Hz, 1H), 8.49 (d, *J* = 8.4 Hz, 1H), 8.25 (dd, *J* = 8.4, 1.7 Hz, 1H), 8.14 (dd, *J* = 7.6, 2.5 Hz, 1H), 8.08 (d, *J* = 8.8 Hz, 1H), 7.72 (d, *J* = 8.4 Hz, 1H), 7.68 – 7.63 (m, 1H), 7.55 (d, *J* = 2.5 Hz, 1H), 7.52 (dd, *J* = 8.4, 1.7 Hz, 1H), 7.29 (app t, *J* = 9.2 Hz, 1H), 6.99 (d, *J* = 9.2 Hz, 1H), 4.37 – 4.27 (m, 4H), 3.79 (s, 2H), 2.54 – 2.27 (m, 8H), 2.16 (br s, 3H). ^{13}C NMR (126 MHz, DMSO-*d$_6$*) δ 165.49, 164.96, 162.20, 152.38 (d, *J* = 243.33 Hz), 148.74, 146.93, 143.42, 138.01, 135.89 (d, *J* = 2.33 Hz), 131.75, 129.22, 128.99, 128.46, 127.93, 126.66, 125.80 (d, *J* = 13.1 Hz), 122.28, 121.73, 119.31, 119.18 (d, *J* = 6.7 Hz), 117.36, 117.17, 116.04 (d, *J* = 20.6 Hz), 64.87, 64.49, 55.21, 53.41, 46.24. HRMS (ESI$^+$): calcd for $C_{31}H_{31}FN_5O_4$ $(M + H)^+$, 556.2355; found 556.2329.

2-((4-(*sec*-Butyl)piperazin-1-yl)methyl)-*N*-(5-(2,3-dihydrobenzo[*b*][1,4]dioxine-6-carboxamido)-2-fluorophenyl)quinoline-6-carboxamide **S53**

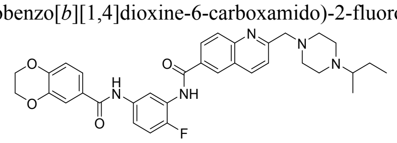

A solution of *N*-(5-(2,3-dihydrobenzo[*b*][1,4]dioxine-6-carboxamido)-2-fluorophenyl)-2-formylquinoline-6-carboxamide (100 mg, 0.212 mmol) and 1-(*sec*-butyl)piperazine (0.103 mL, 0.636 mmol) in anhydrous DCM (2 mL) was allowed to stir at 20 °C for 6 h, after which sodium triacetoxyborohydride (135 mg, 0.636 mmol) was added in one portion and the resulting mixture was allowed to stir at 20 °C for 2 h. The reaction was quenched with $NaHCO_3$ (5 ml) aqueous saturated solution and extracted with DCM/MeOH 9/1 mixture (3 x 5 mL). Purification by column chromatography on silica gel using a gradient of 0-10% MeOH in DCM, followed by trituration in diethyl ether afforded the title compound as an amorphous pale pink solid (51 mg, 40%). ^{1}H-NMR (500 MHz, DMSO-*d$_6$*): δ 10.39 (s, 1H), 10.19 (s, 1H), 8.65 (d, *J* = 1.7 Hz, 1H), 8.49 (d, *J* = 8.5 Hz, 1H), 8.25 (dd, *J* = 8.5, 1.7 Hz, 1H), 8.14 (dd, *J* = 6.8, 2.6 Hz, 1H), 8.08 (d, *J* = 9.1 Hz, 1H), 7.73 (d, *J* = 8.5 Hz, 1H), 7.68 – 7.63 (m, 1H), 7.54 (d, *J* = 1.70 Hz, 1H), 7.52 (d, *J* = 8.5, 2.5 Hz, 1H), 7.29 (app t, *J* = 9.9 Hz, 1H), 6.99 (d, *J* = 8.5 Hz, 1H), 4.36 – 4.27 (m, 4H), 3.79 (s, 2H), 2.61 – 2.28 (m, 9H), 1.55 – 1.38 (m, 1H), 1.35 – 1.16 (m, 1H), 0.91 (br s, 3H), 0.84 (t, *J* = 7.97 Hz, 3H). ^{13}C NMR (126 MHz, DMSO-*d$_6$*) δ 165.49, 164.96, 162.30, 152.30 (d, *J* = 247.7 Hz), 148.75, 146.93, 143.43, 137.97, 135.89 (d, *J* = 2.0 Hz), 131.75, 129.22, 128.99, 128.45, 127.93, 126.66, 125.82 (d, *J* = 13.6 Hz), 122.29, 121.73, 119.28, 119.19 (d, *J* = 7.5 Hz), 117.36, 117.16, 116.05 (d, *J* = 21.1 Hz), 64.88, 64.50, 60.14, 54.07, 48.14, 26.26, 14.13, 11.56. HRMS (ESI$^+$): calcd for $C_{34}H_{37}FN_5O_4$ $(M + H)^+$, 598.2824; found 598.2808.

2-((4-(*tert*-Butyl)piperazin-1-yl)methyl)-*N*-(5-(2,3-dihydrobenzo[*b*][1,4]dioxine-6-carboxamido)-2-fluorophenyl)quinoline-6-carboxamide **S54**

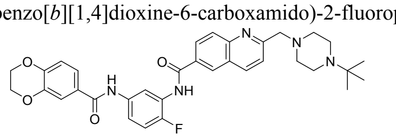

A solution of *N*-(5-(2,3-dihydrobenzo[*b*][1,4]dioxine-6-carboxamido)-2-fluorophenyl)-2-formylquinoline-6-carboxamide (100 mg, 0.212 mmol) and 1-(*tert*-butyl)piperazine (91 mg, 0.636 mmol) in anhydrous DCM (2.000 mL) was allowed to stir at 20 °C for 12 h, after which sodium triacetoxyborohydride (135 mg, 0.636 mmol) was added in one portion and the resulting mixture was allowed to stir at 20 °C for 2 h. The reaction was quenched with $NaHCO_3$ saturated aqueous solution (5 mL) and extracted with a mixture of DCM/MeOH 9/1 (3 x 5 mL). The crude product was purified by column chromatography on silica gel using a gradient of 0-15% MeOH in DCM then washed with water and triturated with diethyl ether to afford the title compound as an amorphous beige solid (63 mg, 50%). ^{1}H-NMR (500 MHz, DMSO-*d$_6$*): δ 10.50 (s, 1H), 10.29 (s, 1H), 8.69 (d, *J* = 1.5 Hz, 1H), 8.50 (d, *J* = 8.4 Hz, 1H), 8.27 (dd, *J* = 8.4, 1.5 Hz, 1H), 8.13 (dd, *J* = 7.3, 2.4 Hz, 1H), 8.09 (d, *J* = 8.5 Hz, 1H), 7.74 (d, *J* = 8.4 Hz, 1H), 7.61 – 7.66 (m, 1H), 7.57 (d, *J* = 2.2 Hz, 1H), 7.55 (d, *J* = 8.5, 2.2 Hz, 1H), 7.28 (app t, *J* = 9.8 Hz, 1H), 6.98 (d, *J* = 8.5 Hz, 1H), 4.35 – 4.27 (m, 4H), 3.85 (s, 2H), 3.09 – 2.54 (m, 8H), 1.20 (br s, 9H). ^{13}C NMR (126 MHz, DMSO-*d$_6$*) δ 165.48, 164.96, 161.49, 152.55 (d, *J* = 247.7 Hz) 148.73, 146.92, 143.41, 138.04, 135.94 (d, *J* = 2.3 Hz), 131.78, 129.21, 129.00, 128.50, 127.91, 126.66, 125.74 (d, *J* = 12.4 Hz), 122.33, 121.73, 119.63, 119.42 (d, *J* = 7.1 Hz), 117.35, 117.23, 116.02 (d, *J* = 20.7 Hz), 64.87, 64.43, 56.26, 53.11, 45.83, 25.59. HRMS (ESI$^+$): calcd for $C_{34}H_{37}FN_5O_4$ $(M + H)^+$, 598.2824; found 598.2809.

(R)-N-(5-(2,3-Dihydrobenzo[*b*][1,4]dioxine-6-carboxamido)-2-fluorophenyl)-2-((4-ethyl-2-methylpiperazin-1-yl)methyl)quinoline-6-carboxamide **S55**

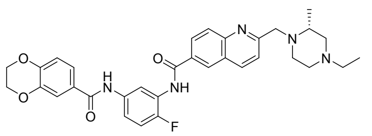

A solution of *N*-(5-(2,3-dihydrobenzo[*b*][1,4]dioxine-6-carboxamido)-2-fluorophenyl)-2-formylquinoline-6-carboxamide (0.200 g, 0.424 mmol) and *(R)-tert*-butyl 3-methylpiperazine-1-carboxylate (255 mg, 1.27 mmol) in anhydrous DCM (2.00 mL) was allowed to stir at 20 °C for 12 h, after which sodium triacetoxyborohydride (0.270 g, 1.27 mmol) was added in one portion and the resulting mixture was allowed to stir at 20 °C for 2 h. The reaction was quenched with $NaHCO_3$ aqueous saturated solution (5 mL) and extracted with a mixture DCM/MeOH 9/1 (3 x 5 mL). The organic layers were dried over Na_2SO_4 and concentrated under reduced pressure to afford the crude product *(R)-tert*-butyl 4-((6-((5-(2,3-dihydrobenzo[b][1,4]dioxine-6-carboxamido)-2-fluorophenyl)carbamoyl)quinolin-2-yl)methyl)-3-methylpiperazine-1-carboxylate (278 mg), which was dissolved in anhydrous DCM (2.5 mL) and treated with TFA (0.162 mL, 2.12 mmol). The resulting mixture was allowed to stir for 18 h, after which it was concentrated under vacuum to afford the crude product *(R)-N*-(5-(2,3-dihydrobenzo[*b*][1,4]dioxine-6-carboxamido)-2-fluorophenyl)-2-((2-methylpiperazin-1-yl)methyl)quinoline-6-carboxamide, which was taken onto the next step without purification (185 mg). To a solution of *(R)-N*-(5-(2,3-dihydrobenzo[*b*][1,4]dioxine-6-carboxamido)-2-fluorophenyl)-2-((2-methylpiperazin-1-yl)methyl)quinoline-6-carboxamide (0.185 g, 0.333 mmol) in anhydrous MeOH (3 mL) at 0 °C, sodium cyanoborohydride (23.02 mg, 0.366 mmol) was added in one portion, followed by the dropwise addition of acetaldehyde (0.013 mL, 0.233 mmol) and the resulting solution was allowed to warm to 20 °C and stir under argon for 18 h. The solvent was removed under reduced pressure and the crude was re-dissolved in DCM and washed with NaOH aqueous solution (1M). Purification by column chromatography on silica gel using a gradient of 0-20% MeOH in DCM, then washed with water and triturated with diethyl ether afforded the title compound as an amorphous white solid (27 mg, 14%). ^{1}H-NMR (500 MHz, DMSO-d_6): δ 10.38 (s, 1H), 10.18 (s, 1H), 8.64 (d, *J* = 2.2 Hz, 1H), 8.48 (d, *J* = 8.8 Hz, 1H), 8.24 (dd, *J* = 8.8, 2.2 Hz, 1H), 8.14 (dd, *J* = 6.6, 2.2 Hz, 1H), 8.07 (d, *J* = 8.8 Hz, 1H), 7.74 (d, *J* = 8.8 Hz, 1H), 7.68 – 7.63 (m, 1H), 7.54 (d, *J* = 2.2 Hz, 1H), 7.52 (d, *J* = 8.8, 2.2 Hz, 1H), 7.29 (app t, *J* = 10.3 Hz, 1H), 6.99 (d, *J* = 8.8 Hz, 1H), 4.35 – 4.28 (m, 4H), 4.23 (d, *J* = 14.2 Hz, 1H), 3.53 (d, *J* = 14.1 Hz, 1H), 2.74 – 2.57 (m, 3H), 2.34 – 2.21 (m, 4H), 2.12 – 1.99 (m, 1H), 1.95 – 1.82 (m, 1H), 1.09 (d, *J* = 6.5 Hz, 3H), 0.98 (t, *J* = 7.0 Hz, 3H). ^{13}C NMR (126 MHz, DMSO-d_6) δ 165.49, 164.95, 163.37, 153.31, 152.41 (d, *J* = 241.12 Hz), 146.91, 143.41, 137.80, 135.91 (d, *J* = 2.70 Hz), 131.65, 129.15, 128.99, 128.44, 127.92, 126.58, 125.81 (d, *J* = 13.49 Hz), 122.32, 121.75, 119.36, 119.21 (d, *J* = 6.28 Hz), 117.34, 117.19, 116.02 (d, *J* = 20.16 Hz), 64.87, 64.49, 60.70, 55.68, 53.09, 52.34, 52.02, 29.47, 17.61, 12.44. HRMS (ESI$^+$): calcd for $C_{33}H_{35}FN_5O_4$ (M + H)$^+$, 584.2668; found 584.2665.

N-(5-(2,3-Dihydrobenzo[*b*][1,4]dioxine-6-carboxamido)-2-fluorophenyl)-2-((4-ethyl-1,4-diazepan-1-yl)methyl)quinoline-6-carboxamide **S56**

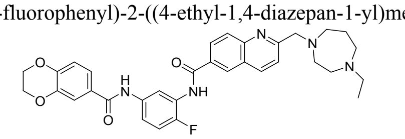

A solution of *N*-(5-(2,3-dihydrobenzo[*b*][1,4]dioxine-6-carboxamido)-2-fluorophenyl)-2-formylquinoline-6-carboxamide (250 mg, 0.530 mmol) and 1-ethyl-1,4-diazepane (204 mg, 1.59 mmol) in anhydrous DCM (5 mL) was allowed to stir at 20 °C for 18 h, after which sodium triacetoxyborohydride (337 mg, 1.59mmol) was added in one portion and the resulting mixture was allowed to stir at 20 °C for 3 h. The reaction was quenched with $NaHCO_3$ saturated aqueous solution (5 mL) and extracted with a mixture DCM/MeOH 9/1 (3 x 5 mL). Purification by column chromatography on silica gel using a gradient of 0-10% MeOH in DCM, followed by elution through an Isolute SCX-II cartridge using MeOH/NH_3, and trituration with MeOH, afforded the title compound as an amorphous pale yellow solid (40 mg, 13%).. ^{1}H NMR (500 MHz, DMSO-d_6) δ 10.43 (s, 1H), 10.23 (s, 1H), 8.68 (d, *J* = 1.7 Hz, 1H), 8.51 (d, *J* = 8.5 Hz, 1H), 8.27 (dd, *J* = 8.8, 1.9 Hz, 1H), 8.14 (dd, *J* = 7.0, 2.4 Hz, 1H), 8.08 (d, *J* = 8.8 Hz, 1H), 7.80 (d, *J* = 8.5 Hz, 1H), 7.66 (ddd, *J* = 8.7, 4.1, 2.7 Hz, 1H), 7.58 – 7.50 (m, 2H), 7.29 (t, *J* = 9.5 Hz, 1H), 6.99 (d, *J* = 8.4 Hz, 1H), 4.31 (q, *J* = 5.0 Hz, 5H), 4.00 (s, 2H), 3.07 (s, 4H), 2.94 (s, 2H), 2.82 – 2.73 (m, 2H), 1.97 (s, 2H), 1.20 (s, 4H). ^{13}C NMR (126 MHz, DMSO-d_6) δ 165.47, 164.96, 162.47, 152.56 (d, *J* = 242.7 Hz), 148.71, 146.92, 143.42, 138.11, 134.86 (d, *J* = 2.5 Hz), 131.79, 129.22, 129.04, 128.53, 127.92, 126.70, 125.81 (d, *J* = 13.9 Hz), 122.28, 121.75, 119.41, 119.28 (d, *J* = 8.8 Hz), 117.34, 117.20, 116.01 (d, *J* = 20.2 Hz), 64.88, 64.49, 64.17, 54.26, 53.74, 52.32, 51.78, 49.58, 26.29, 10.79. HRMS (ESI$^+$): calcd for $C_{33}H_{35}FN_5O_4$ (M + H)$^+$, 584.2673; found 584.2697.

2-((4-Cyclopropylpiperazin-1-yl)methyl)-*N*-(5-(2,3-dihydrobenzo[*b*][1,4]dioxine-6-carboxamido)-2-fluorophenyl)quinoline-6-carboxamide **S57**

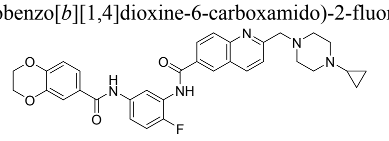

A solution of *N*-(5-(2,3-dihydrobenzo[*b*][1,4]dioxine-6-carboxamido)-2-fluorophenyl)-2-formylquinoline-6-carboxamide (100 mg, 0.212 mmol) and 1-cyclopropylpiperazine (0.077 mL, 0.636 mmol) in anhydrous DCM (2 mL) was allowed to stir at 20 °C for 6 h, after which sodium triacetoxyborohydride (135 mg, 0.636 mmol) was added in one portion and the resulting mixture was allowed to stir at 20 °C for 2 h. The reaction was quenched with $NaHCO_3$ aqueous saturated solution (5 mL) and extracted with DCM/MeOH 9/1 mixture (3 x 5 mL). Purification by column chromatography on silica gel using a gradient of 0-10% MeOH in DCM, then triturated with diethyl ether afforded the desired product as a pale yellow amorphous solid (47 mg, 38.1%). ^{1}H-NMR (500 MHz, DMSO-d_6) δ 10.38 (s, 1H), 10.18 (s, 1H), 8.65 (d, *J* = 1.7 Hz, 1H), 8.49 (d, *J* = 8.5 Hz, 1H), 8.25 (dd, *J* = 9.1, 2.6 Hz, 1H), 8.14 (dd, *J* 6.8, 2.6 Hz, 1H), 8.08 (d, *J* = 9.15 Hz, 1H), 7.73 (d, *J* = 8.5 Hz, 1H), 7.68 – 7.63 (m, 1H), 7.54 (d, *J* = 1.7 Hz, 1H), 7.52 (d, *J* = 7.7, 1.7 Hz, 1H), 7.30 (app t, *J* = 9.9 Hz, 1H), 6.99 (d, *J* = 8.5 Hz, 1H), 4.34 – 4.28 (m, 4H), 3.78 (s, 2H), 2.57 (br s, 4H), 2.44 (br s, 4H), 1.61 (app heptet, *J* = 3.3 Hz, 1H), 0.42 – 0.37 (m, 2H), 0.29 – 0.25 (m, 2H). ^{13}C NMR (126 MHz, DMSO-d_6) δ 165.48, 164.95, 162.19, 152.33 (d, *J* = 243.7 Hz), 148.73, 146.92, 143.42, 138.00, 135.91 (d, *J* = 2.4 Hz), 131.74, 129.22, 128.98, 128.45, 127.93, 126.66, 125.81 (d, *J* = 12.2 Hz), 122.28, 121.72, 119.27, 119.18 (d, *J* = 7.4 Hz), 117.36, 117.16, 116.05 (d, *J* = 21.2 Hz), 64.88, 64.49, 53.47, 53.30, 53.26, 38.48, 6.10. HRMS (ESI$^+$): calcd for $C_{33}H_{33}FN_5O_4$ (M + H)$^+$, 582.2511; found 582.2487.

N-(2-Bromo-5-nitrophenyl)-2-methylquinoline-6-carboxamide **S58**

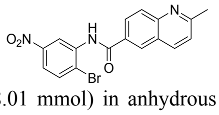

To a suspension of 2-methylquinoline-6-carboxylic acid (1.50 g, 8.01 mmol) in anhydrous DCM (20 mL), DMF (1.11 µl, 0.014 mmol), followed by oxalyl chloride (0.588 mL, 6.95 mmol) were added dropwise and the resulting green solution was allowed to stir at 20 °C for 3 h, after which it was concentrated under vacuum to afford a dry pale green solid. The solid was dissolved in pyridine (20mL) and 2-bromo-5-nitroaniline (1.26 g, 5.79 mmol) was added in one portion. The resulting dark yellow suspension was allowed to stir for 2 h, after which it was poured into water. The resulting yellow precipitate was filtered and washed several times with water, diethyl ether and finally with a minimum amount of DCM to afford the crude product as an amorphous yellow solid, which was used without further purification n (2.47 g). ^{1}H-NMR (500 MHz, DMSO-d_6): δ 10.56 (s, 1H), 8.65 (d, *J* = 1.9 Hz, 1H), 8.52 (app t, *J* = 1.8 Hz, 1H), 8.44 (d, *J* = 8.3 Hz, 1H), 8.26 (dd, *J* = 8.9, 1.88 Hz, 1H), 8.09 – 8.05 (m, 3H), 7.55 (d, *J* = 8.3 Hz, 1H), 2.71 (s, 3H). HRMS (ESI$^+$): calcd for $C_{17}H_{13}{}^{79}BrN_3O_3$ (M + H)$^+$, 386.0135; found 386.0129.

N-(5-Amino-2-bromophenyl)-2-methylquinoline-6-carboxamide **S59**

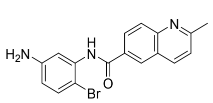

To a solution of *N*-(2-bromo-5-nitrophenyl)-2-methylquinoline-6-carboxamide (2.00 g, 5.18 mmol) in water (7 mL) and EtOH (21 mL), ammonium chloride (1.939 g, 36.3 mmol) and iron powder (2.03 g, 36.3 mmol) were added and the resulting suspension was allowed to stir at 90 °C for 1 h. The reaction mixture was allowed to cool to room temperature, diluted with MeOH and DCM and filtered through a pad of Celite®. The resulting filtrate was concentrated under vacuum to afford a light brown solid as crude product, which was taken onto the next step without purification (1.80 g, 98%). ^{1}H-NMR (500 MHz, DMSO-d_6): δ 9.94 (s, 1H), 8.59 (d, J = 1.8 Hz, 1H), 8.40 (d, J = 8.8 Hz, 1H), 8.22 (dd, J = 8.8, 1.8 Hz, 1H), 8.02 (d, J = 8.8 Hz, 1H), 7.52 (d, J = 8.8 Hz, 1H), 7.28 (d, J = 8.8 Hz, 1H), 6.86 (d, J = 1.8 Hz, 1H), 6.45 (dd, J = 8.8, 1.8 Hz, 1H), 5.40 (br s, 2H), 2.70 (s, 3H). ^{13}C NMR (126 MHz, DMSO-d_6) δ 165.22, 161.30, 149.30, 148.96, 137.63, 136.84, 132.78, 131.74, 128.87, 128.64, 128.23, 125.84, 123.47, 114.07, 105.01, 49.06, 25.51. HRMS (ESI$^+$): calcd for $C_{17}H_{15}{}^{79}BrN_3O$ (M + H)$^+$, 358.0393; found 358.0386.

N-(2-Bromo-5-(2,3-dihydrobenzo[*b*][1,4]dioxine-6-carboxamido)phenyl)-2-methylquinoline-6-carboxamide **S60**

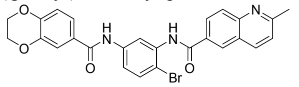

To a suspension of 2,3-dihydrobenzo[*b*][1,4]dioxine-6-carboxylic acid (1.00 g, 5.56 mmol) in anhydrous DCM (20 mL), DMF (0.972 µl, 0.013 mmol) and oxalyl chloride (0.513 mL, 6.06 mmol) were added dropwise and the resulting green solution was allowed to stir at 20 °C for 3 h, after which it was concentrated under vacuum to afford a dry pale green solid. The solid was dissolved in pyridine (20 mL) and *N*-(5-amino-2-bromophenyl)-2-methylquinoline-6-carboxamide (1.80 g, 5.05 mmol) was added in one portion. The resulting dark yellow suspension was allowed to stir for 72 h, after which it was poured into water. The resulting yellow precipitate was filtered and washed several times with water, diethyl ether and finally with a minimum amount of DCM to afford the crude product as a pale yellow amorphous solid, which was used without further purification (2.11 g, 81%). ^{1}H-NMR (500 MHz, DMSO-d_6): δ 10.29 (s, 1H), 10.27 (s, 1H), 8.63 (s, 1H), 8.42 (d, J = 9.1 Hz, 1H), 8.25 (d, J = 7.3 Hz, 1H), 8.10 (s, 1H), 8.05 (d, J = 8.2 Hz, 1H), 7.69 (br s, 2H), 7.58 – 7.49 (m, 3H), 6.99 (d, J = 9.1 Hz, 1H), 4.37 – 4.27 (m, 4H), 2.71 (s, 3H). ^{13}C NMR (126 MHz, DMSO-d_6) δ 165.49, 165.13, 161.43, 149.03, 147.04, 143.43, 139.67, 137.68, 136.89, 132.90, 131.43, 128.95, 128.81, 128.25, 127.76, 125.86, 123.54, 121.79, 120.72, 120.18, 117.39, 117.21, 114.50, 64.88, 64.49, 25.51. HRMS (ESI$^+$): calcd for $C_{26}H_{21}{}^{79}BrN_3NaO_4$ (M + Na)$^+$, 540.0529; found 520.0542.

N-(2-Bromo-5-(2,3-dihydrobenzo[*b*][1,4]dioxine-6-carboxamido)phenyl)-2-formylquinoline-6-carboxamide **S61**

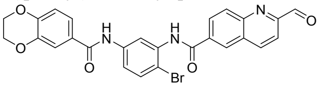

A solution of *N*-(2-bromo-5-(2,3-dihydrobenzo[*b*][1,4]dioxine-6-carboxamido)phenyl)-2-methylquinoline-6-carboxamide (1.00 g, 1.93 mmol) and selenium dioxide (0.235 g, 2.12 mmol) in anhydrous DMF (4 mL) and anhydrous 1,4-dioxane (12 mL) was heated at 150 °C for 1 h. The reaction mixture was allowed to cool to room temperature and it was diluted with DCM and filtered through a pad of Celite®. The filtrate was concentrated under vacuum to afford the crude product as a yellow amorphous solid, which was used without purification (1.00 g, 97%). ^{1}H-NMR (500 MHz, DMSO-d_6): δ 10.47 (s, 1H), 10.28 (s, 1H), 10.17 (d, J = 0.5 Hz, 1H), 8.81 – 8.77 (m, 2H), 8.42 – 8.36 (m, 2H), 8.13 – 8.11 (m, 1H), 8.09 (d, J = 8.4 Hz, 1H), 7.70 (d, J = 1.1 Hz, 2H), 7.55 (d, J = 2.2 Hz, 1H), 7.52 (dd, J = 8.6, 2.2 Hz, 1H), 7.00 (d, J = 8.6 Hz, 1H), 4.36 – 4.23 (m, 4H). HRMS (ESI$^+$): calcd for $C_{26}H_{19}{}^{79}BrN_3O_5$ (M + H)$^+$ 532.0503; found 532.0513.

2-(Azetidin-1-ylmethyl)-*N*-(2-bromo-5-(2,3-dihydrobenzo[*b*][1,4]dioxine-6-carboxamido)phenyl)quinoline-6-carboxamide **S62**

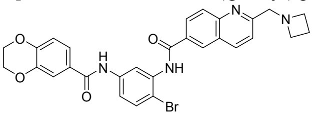

A solution of *N*-(2-bromo-5-(2,3-dihydrobenzo[*b*][1,4]dioxine-6-carboxamido)phenyl)-2-formylquinoline-6-carboxamide (0.500 g, 0.939 mmol) and azetidine (161 mg, 2.82 mmol) in anhydrous DCM (8 mL) was allowed to stir at 20 °C for 12 h, after which sodium triacetoxyborohydride (0.597 g, 2.82 mmol) was added in one portion and the resulting mixture was allowed to stir at 20 °C for 1 h. The reaction was quenched with $NaHCO_3$ aqueous saturated solution (10 mL) and extracted with DCM/MeOH 9/1 mixture (3 x 10 mL). Purification by column chromatography on silica gel using a gradient of 0-20% MeOH in DCM, followed by washing with water, trituration with diethyl ether and elution through an Isolute SCX-II cartridge using MeOH/NH_3, afforded the title compound as a pale beige amorphous solid (54 mg, 10%). ^{1}H-NMR (500 MHz, DMSO-d_6): δ 10.33 (s, 1H), 10.27 (s, 1H), 8.67 (d, J = 1.5 Hz, 1H), 8.53 (d, J = 8.0 Hz, 1H), 8.29 (dd, J = 8.8, 1.5 Hz, 1H), 8.13 – 8.09 (m, 2H), 7.69 (d, J = 1.5 Hz, 1H), 7.65 (d, J = 8.8 Hz, 2H), 7.55 (d, J = 2.4 Hz, 1H), 7.52 (dd, J = 8.0, 1.5 Hz, 1H), 7.00 (d, J = 8.0 Hz, 1H), 4.35 – 4.28 (m, 4H) 4.19 (br s, 2H), 3.59 (br s, 4H), 2.18 (br s, 2H). ^{13}C NMR (126 MHz, DMSO-d_6) δ 165.31, 165.13, 157.8, 148.47, 147.05, 143.43, 140.01, 139.71, 138.62, 136.81, 132.90, 132.34, 129.29, 128.99, 128.82, 127.74, 126.77, 121.81, 120.80, 120.29, 117.38, 117.23, 114.57, 64.89, 64.48, 61.17, 55.23, 17.44. HRMS (ESI$^+$): calcd for $C_{29}H_{26}{}^{81}BrN_4O_4$ (M + H)$^+$, 575.1116; found575.1088.

N-(2-Bromo-5-(2,3-dihydrobenzo[*b*][1,4]dioxine-6-carboxamido)phenyl)-2-((4-ethylpiperazin-1-yl)methyl)quinoline-6-carboxamide **S63**

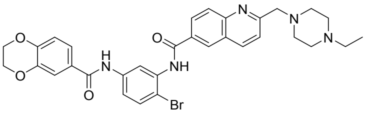

A solution of *N*-(2-bromo-5-(2,3-dihydrobenzo[*b*][1,4]dioxine-6-carboxamido)phenyl)-2-formylquinoline-6-carboxamide (0.500 g, 0.939 mmol) and 1-ethylpiperazine (322 mg, 2.82 mmol) in anhydrous DCM (8 mL) was allowed to stir at 20 °C for 12 h, after which sodium triacetoxyborohydride (0.597 g, 2.82 mmol) was added in one portion and the resulting mixture was allowed to stir at 20 °C for 1 h. The reaction was quenched with $NaHCO_3$ aqueous saturated solution (10 mL) and extracted with DCM/MeOH 9/1 mixture (3 x 10 mL). Purification by column chromatography on silica gel using a gradient of 0-20% MeOH in DCM, followed by elution through an Isolute SCX-II cartridge using MeOH/NH_3, afforded the title compound as a bright yellow amorphous solid (110 mg, 18%). ^{1}H-NMR (500 MHz, DMSO-d_6): δ 10.31 (s, 1H), 10.27 (s, 1H), 8.66 (d, J = 2.18 Hz, 1H), 8.50 (d, J = 8.70 Hz, 1H), 8.27 (dd, J = 8.70, 2.18 Hz, 1H), 8.13 – 8.08 (m, 2H), 7.73 (d, J = 8.70 Hz, 1H), 7.70 – 7.68 (m, 2H), 7.55 (d, J = 2.18 Hz, 1H), 7.52 (dd, J = 8.70, 2.18 Hz, 1H), 7.00 (d, J = 8.70 Hz, 1H), 4.35 – 4.28 (m, 4H), 3.81 (br s, 2H), 2.51 (br s, 10 H), 1.02 (br t, J = 6.68 Hz, 3H. ^{13}C NMR (126 MHz, DMSO-d_6) δ 165.39, 165.12, 162.02, 148.74, 147.05, 143.44, 139.69, 138.01, 136.85, 132.90, 131.89, 129.30, 128.83, 128.32, 127.75, 126.71, 122.32, 121.79, 120.73, 120.20, 117.39, 117.21, 114.49, 64.88, 64.68, 64.49, 53.13, 52.61, 51.94, 12.18. HRMS (ESI$^+$): calcd for $C_{32}H_{33}{}^{79}BrN_5O_4$ (M + H)$^+$, 630.1710; found 632.1694.

Synthesis of the preclinical development candidate CCT361814

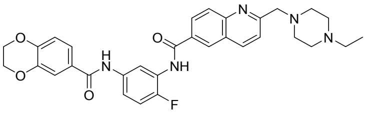

N-(5-(2,3-Dihydrobenzo[*b*][1,4]dioxine-6-carboxamido)-2-fluorophenyl)-2-((4-ethylpiperazin-1-yl)methyl)quinoline-6-carboxamide **22**.

Step 1. N-(2-Fluoro-5-nitrophenyl)-2-methylquinoline-6-carboxamide. Oxalyl chloride (3.25 mL, 38.4 mmol) was added dropwise to a solution of 2-methylquinoline-6-carboxylic acid (6.59 g, 35.2 mmol) and DMF (0.0062 mL, 0.080 mmol) in anhydrous DCM (80 mL). The reaction mixture was stirred at room temperature for 3 h and then concentrated under reduced pressure. The residue was dissolved in DCM (30 mL) and concentrated again under reduced pressure.

The resulting dry residue was dissolved in pyridine (80 mL) and 2-fluoro-5-nitroaniline (5.00 g, 32.0 mmol) was added in one portion. The reaction mixture was stirred at room temperature for 18 h and then poured onto water (100 mL). The green precipitate was filtered and washed with water (3 x 20 mL), diethyl ether (3 x 20 mL) and DCM (10 mL), to afford the title compound as a light green solid, which was carried onto the next step without further purification (10.4 g). ^{1}H NMR (500 MHz, DMSO-d$_6$) δ 10.70 (s, 1H), 8.72 (dd, *J* = 6.45, 2.93 Hz, 1H), 8.63 (d, *J* = 2.02 Hz, 1H), 8.43 (d, *J* = 8.46 Hz, 1H), 8.23 (dd, *J* = 8.48, 2.02 Hz, 1H), 8.21 – 8.16 (m, 1H), 8.05 (d, *J* = 8.86 Hz, 1H), 7.65 (app t, *J* = 9.25 Hz, 1H), 7.54 (d, *J* = 8.46 Hz, 1H), 2.71 (s, 3H). ^{13}C NMR (126 MHz, DMSO-*d*$_6$) δ 165.53, 161.22, 158.67 (d, *J* = 258.2 Hz), 148.65, 143.72, 137.32, 130.36, 128.88, 128.48, 128.00, 127.08 (d, *J* = 13.9 Hz), 125.33, 123.18, 122.14 (d, *J* = 9.6 Hz), 121.25 (d, *J* = 3.8 Hz), 117.19 (d, *J* = 22.8 Hz), 25.07. ^{19}F NMR (470 MHz, DMSO-d$_6$) δ -110.20. HRMS (ESI$^+$): calcd for $C_{17}H_{13}FN_3O_3$ (M + H)$^+$, 326.0935; found 326.0931.

Step 2. N-(5-Amino-2-fluorophenyl)-2-methylquinoline-6-carboxamide. To a solution of *N*-(2-fluoro-5-nitrophenyl)-2-methylquinoline-6-carboxamide (10.4 g, 32.0 mmol) in ethanol (120 mL) and water (40 mL), ammonium chloride (12.0 g, 224 mmol) and iron powder (12.5 g, 224 mmol) were added in one portion and the resulting suspension was allowed to stir at 90 °C for 1 h. The reaction mixture was allowed to cool to room temperature, diluted with MeOH (20 mL) and DCM (20 mL) and filtered through a pad of Celite. The resulting filtrate was concentrated under vacuum to afford a light brown solid which was re-dissolved in a mixture of DCM:MeOH (9:1, 150 mL) and washed with saturated aqueous $NaHCO_3$ (150 mL). The organic phase was dried over Na_2SO_4, filtered and concentrated under reduced pressure to afford a yellow solid as crude product, which was taken directly onto the next step without further purification (9.46 g). ^{1}H NMR (500 MHz, DMSO-d$_6$) δ 10.05 (s, 1H), 8.57 (d, *J* = 1.67 Hz, 1H), 8.39 (d, *J* = 8.74 Hz, 1H), 8.19 (dd, *J* = 8.74, 1.67 Hz, 1H), 8.01 (d, *J* = 8.74 Hz, 1H), 7.52 (d, *J* = 8.33 Hz, 1H), 6.94 (dd, *J* = 9.78, 8.28 Hz, 1H), 6.89 (dd, *J* = 6.58, 2.74 Hz, 1H), 6.46 – 6.39 (m, 1H), 5.05 (br s, 2H), 2.70 (s, 3H). ^{13}C NMR (126 MHz, DMSO-*d*$_6$) δ 164.93, 160.84, 148.49, 147.72 (d, *J* = 233.9 Hz), 145.08 (d, *J* = 1.9 Hz), 137.19, 131.19, 128.44, 128.33, 127.95, 125.50 (d, *J* = 13.1 Hz), 125.34, 122.99, 115.54 (d, *J* = 20.6 Hz), 111.52, 111.39 (d, *J* = 6.6 Hz), 25.05. ^{19}F NMR (470 MHz, DMSO-d$_6$) δ -138.12. HRMS (ESI$^+$): calcd for $C_{17}H_{15}FN_3O$ (M + H)$^+$, 296.1194; found 296.1191.

Step. 3. N-(5-(2,3-Dihydrobenzo[b][1,4]dioxine-6-carboxamido)-2-fluorophenyl)-2-methylquinoline-6-carboxamide. To a suspension of 2,3-dihydrobenzo[*b*][1,4]dioxine-6-carboxylic acid (12.7 g, 70.5 mmol) in anhydrous DCM (100 mL) under inert atmosphere, was dropwise added a catalytic amount of anhydrous DMF (6.16 µl, 0.080 mmol) and oxalyl chloride (6.51 mL, 77.0 mmol) and the resulting green solution was allowed to stir at room temperature for 3 h. After which time, the reaction mixture was concentrated under vacuum to afford a dry pale green solid. The solid was dissolved in pyridine (100 mL) and *N*-(5-amino-2-fluorophenyl)-2-methylquinoline-6-carboxamide (9.46 g, 32.0 mmol) was added in one portion. The resulting dark yellow suspension was allowed to stir for 2 h and was then poured onto water (100 mL). The yellow precipitate was filtered and washed with water (3 x 20 mL), diethyl ether (3 x 20 mL) and DCM (10 mL), to afford the crude product as a pale yellow solid, which was taken directly onto the next step without further purification (12.5 g). ^{1}H NMR (500 MHz, DMSO-d$_6$): δ 10.37 (s, 1H), 10.18 (s, 1H), 8.62 (d, *J* = 1.65 Hz, 1H), 8.41 (d, *J* = 8.77 Hz, 1H), 8.23 (dd, *J* = 8.77, 2.19 Hz, 1H), 8.13 (dd, *J* = 7.01, 2.63 Hz, 1H), 8.03 (d, *J* = 8.51 Hz, 1H), 7.68 – 7.63 (m, 1H), 7.55 – 7.53 (m, 2H), 7.52 (dd, *J* = 8.51, 2.09 Hz, 1H), 7.29 (dd, *J* = 9.98, 8.69 Hz, 1H), 6.99 (d, *J* = 8.51 Hz, 1H), 4.34 – 4.28 (m, 4H), 2.71 (s, 3H). ^{13}C NMR (126 MHz, DMSO-*d*$_6$) δ 165.09, 164.48, 160.95, 151.83 (d, *J* = 243.4 Hz), 148.57, 146.45, 142.95, 137.22, 135.42 (d, *J* = 2.0 Hz), 130.87, 128.50, 128.41, 127.94, 127.47, 125.39 (d, *J* = 9.5 Hz), 125.35, 123.05, 121.25, 118.81, 118.75 (d, *J* = 13.0 Hz), 116.89, 116.69, 115.56 (d, *J* = 21.2 Hz), 64.41, 64.03, 25.06. ^{19}F NMR (470 MHz, DMSO-d$_6$) δ -126.65. HRMS (ESI+): calcd for $C_{26}H_{21}FN_3O_4$ (M + H)$^+$, 458.1511; found 458.1499.

Step 4. N-(5-(2,3-Dihydrobenzo[b][1,4]dioxine-6-carboxamido)-2-fluorophenyl)-2-formylquinoline-6-carboxamide. A solution of *N*-(5-(2,3-dihydrobenzo[*b*][1,4]dioxine-6-carboxamido)-2-fluorophenyl)-2-methylquinoline-6-carboxamide (5.00 g, 10.9 mmol) and selenium dioxide (1.33 g, 12.0 mmol) in anhydrous DMF (40 mL) and 1,4-dioxane (120 mL) was heated at reflux for 1 h under an inert atmosphere. After which time, the reaction mixture was allowed to cool to room temperature, diluted with DCM (20 mL) and filtered through a pad of Celite. The filtrate was concentrated under vacuum (using a heptane/EtOAc azeotrope to remove DMF) to afford the crude product as a yellow solid, which was carried onto the next step without further purification (5.15 g). ^{1}H NMR (500 MHz, DMSO-d$_6$): δ 10.54 (s, 1H), 10.19 (s, 1H), 10.17 (s, 1H), 8.81 – 8.77 (m, 2H), 8.39 (dd, *J* = 8.73, 1.95 Hz, 1H), 8.36 (d, *J* = 8.73 Hz, 1H), 8.17 (dd, *J* = 6.93, 2.57 Hz, 1H), 8.09 (d, *J* = 9.26 Hz, 1H), 7.69 – 7.64 (m, 1H), 7.55 (d, *J* = 1.99 Hz, 1H), 7.52 (dd, *J* = 8.30, 1.99 Hz, 1H), 7.31 (app t, *J* = 9.97 Hz, 1H), 6.99 (d, *J* = 8.30 Hz, 1H), 4.36 – 4.27 (m, 4H). HRMS (ESI+): calcd for $C_{26}H_{19}FN_3O_5$ (M + H)$^+$, 472.1303; found 472.1286.

*Step 5. N-(5-(2,3-Dihydrobenzo[b][1,4]dioxine-6-carboxamido)-2-fluorophenyl)-2-((4-ethylpiperazin-1-yl)methyl)quinoline-6-carboxamide (**22**).* A solution of *N*-(5-(2,3-dihydrobenzo[*b*][1,4]dioxine-6-carboxamido)-2-fluorophenyl)-2-formylquinoline-6-carboxamide (1.19 g, 2.52 mmol) and 1-ethylpiperazine (7.57 mmol, 0.87 g, 0.96 mL) in anhydrous DCM (20 mL) was allowed to stir at 20 °C for 6 h. After which time, sodium triacetoxyborohydride (1.61 g, 7.57 mmol) was added in one portion and the resulting mixture was allowed to stir at 20 °C for 2 h. The reaction was quenched with saturated aqueous $NaHCO_3$ (20 mL) and extracted with a mixture DCM:MeOH (9:1, 3 x 20 mL). Purification by column chromatography on silica gel in gradient with DCM:MeOH (0-10%) afforded a yellow solid, which was re-dissolved in DCM:MeOH (9:1, 100 mL) and washed with water (100 mL). The organic phase was dried over Na_2SO_4, filtered and concentrated under reduced pressure. Final trituration of the resulting residue with diethyl ether (20 mL) afforded the title compound as a white solid (0.95 g, 66%). ^{1}H NMR (500 MHz, DMSO-d$_6$) δ 10.39 (s, 1H), 10.18 (s, 1H), 8.65 (d, *J* = 1.9 Hz, 1H), 8.49 (d, *J* = 8.4 Hz, 1H), 8.25 (dd, *J* = 8.8, 2.0 Hz, 1H), 8.14 (dd, *J* = 7.1, 2.6 Hz, 1H), 8.08 (d, *J* = 8.8 Hz, 1H), 7.73 (d, *J* = 8.5 Hz, 1H), 7.67–7.64 (m, 1H), 7.55 (d, *J* = 1.8 Hz, 1H), 7.53 (dd, *J* = 8.5, 1.8 Hz, 1H),7.29 (app t, *J* = 9.2 Hz, 1H), 6.99 (d, *J* = 8.4 Hz, 1H), 4.39 – 4.18 (m, 4H), 3.79 (s, 2H), 2.48–2.41 (m, 8H), 2.32 (q, *J* = 7.2 Hz, 2H), 0.98 (t, *J* = 7.2 Hz, 3H). ^{13}C NMR (126 MHz, DMSO) δ 165.02, 164.50, 161.69, 151.85 (d, *J* = 242.7 Hz), 148.28, 146.46, 142.96, 137.53, 135.44 (d, *J* = 2.4 Hz), 131.29, 128.76, 128.53, 128.00, 127.47, 126.20, 125.35 (d, *J* = 12.9 Hz), 121.81, 121.27, 118.83, 118.72 (d, *J* = 7.3 Hz), 116.89, 116.71, 115.57 (d, *J* = 21.0 Hz), 64.42, 64.38, 64.04, 52.93, 52.34, 51.60, 11.91. . ^{19}F NMR (470 MHz, DMSO-d$_6$) δ -126.63. HRMS (ESI+): calcd for $C_{32}H_{33}FN_5O_4$ (M + H)$^+$, 570.2511; found 570.2532.

NMR-Spectra of Key Compounds

^{1}H NMR (DMSO-d_6, 500 MHz) and ^{13}C NMR (DMSO-d_6, 126 MHz) of the chemical probe CCT251236 (**1**)

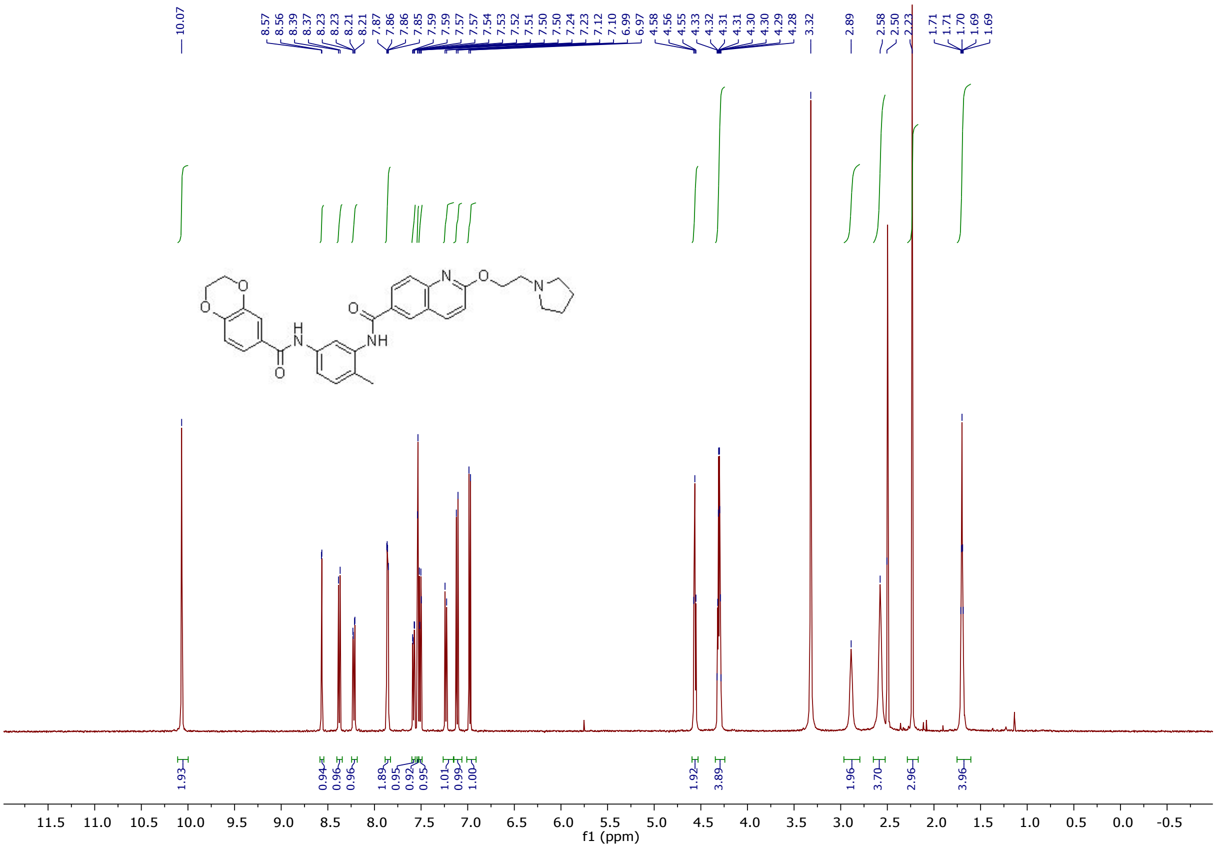

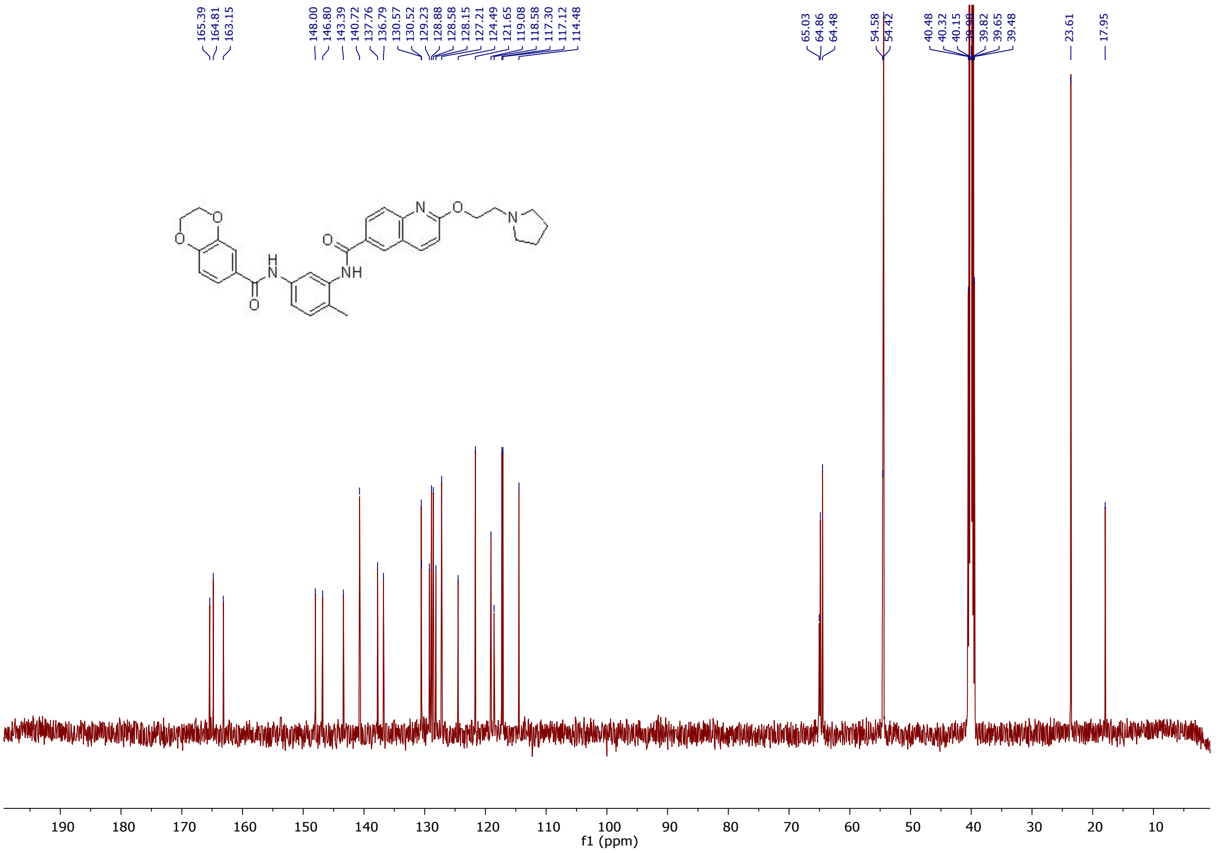

^{1}H NMR (DMSO-d_6, 500 MHz) and ^{13}C NMR (DMSO-d_6, 126 MHz) of compound **11**

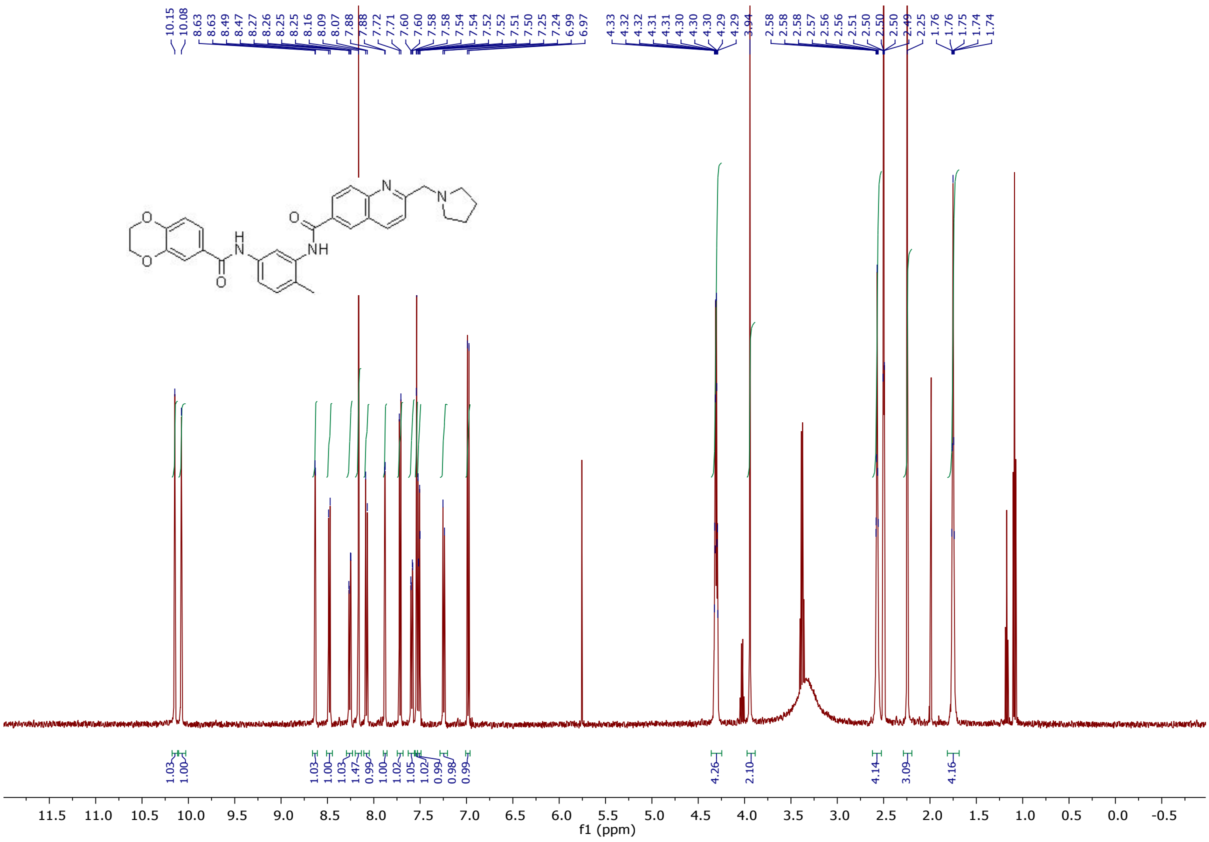

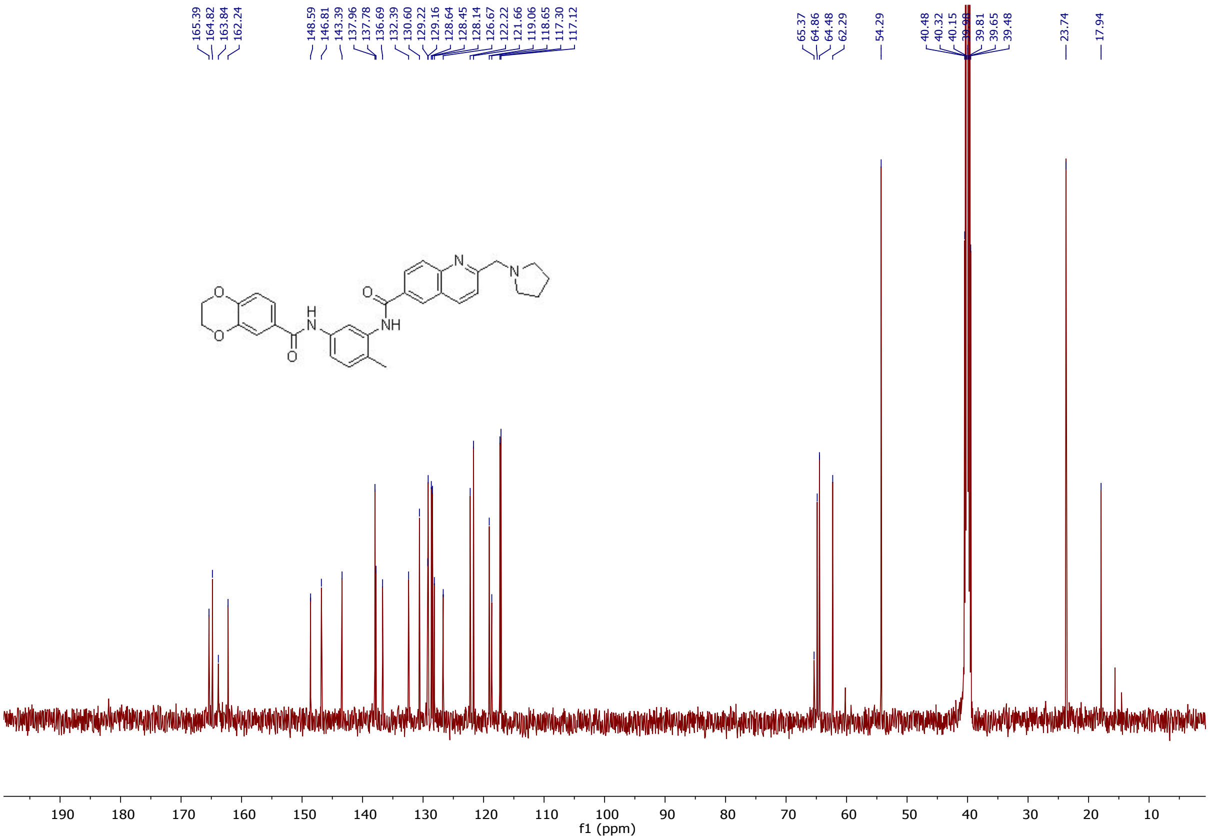

^{1}H NMR (DMSO-d_6, 500 MHz) and ^{13}C NMR (DMSO-d_6, 126 MHz) of compound **12**

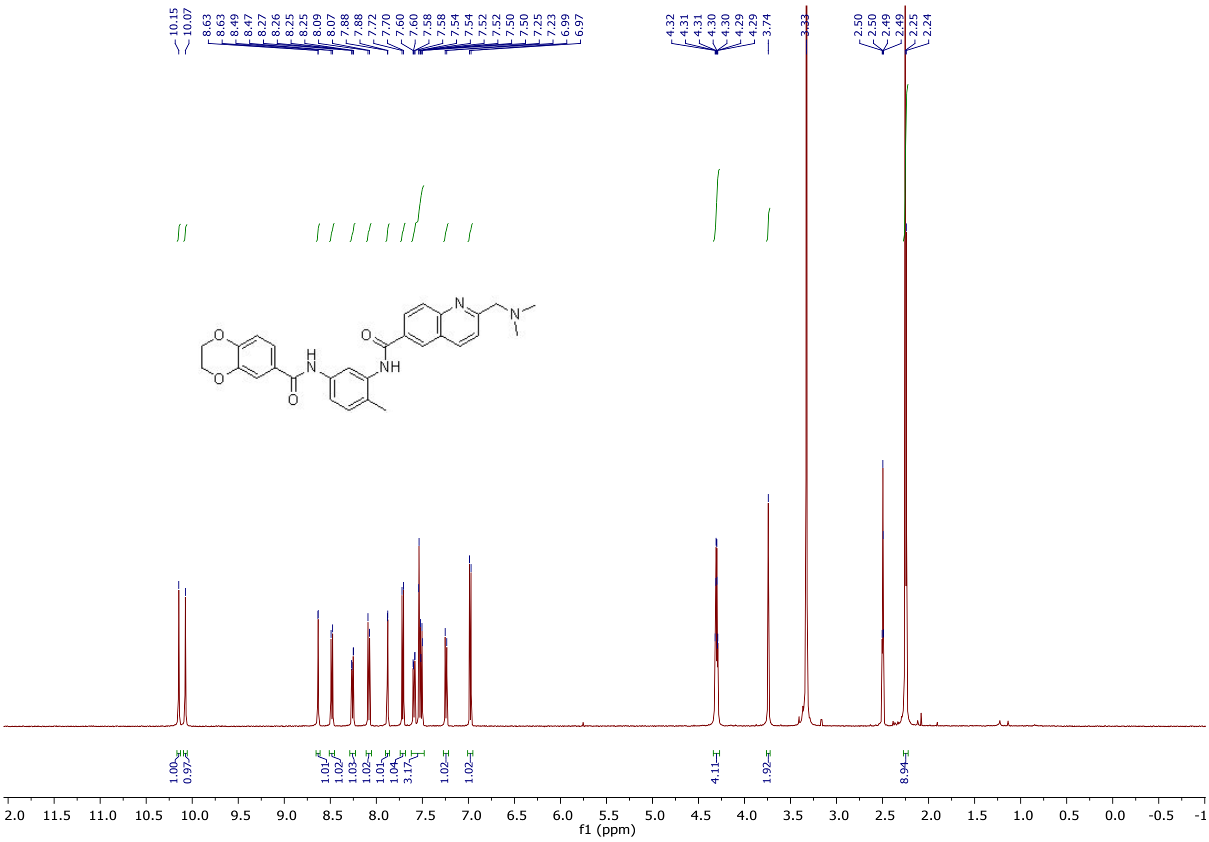

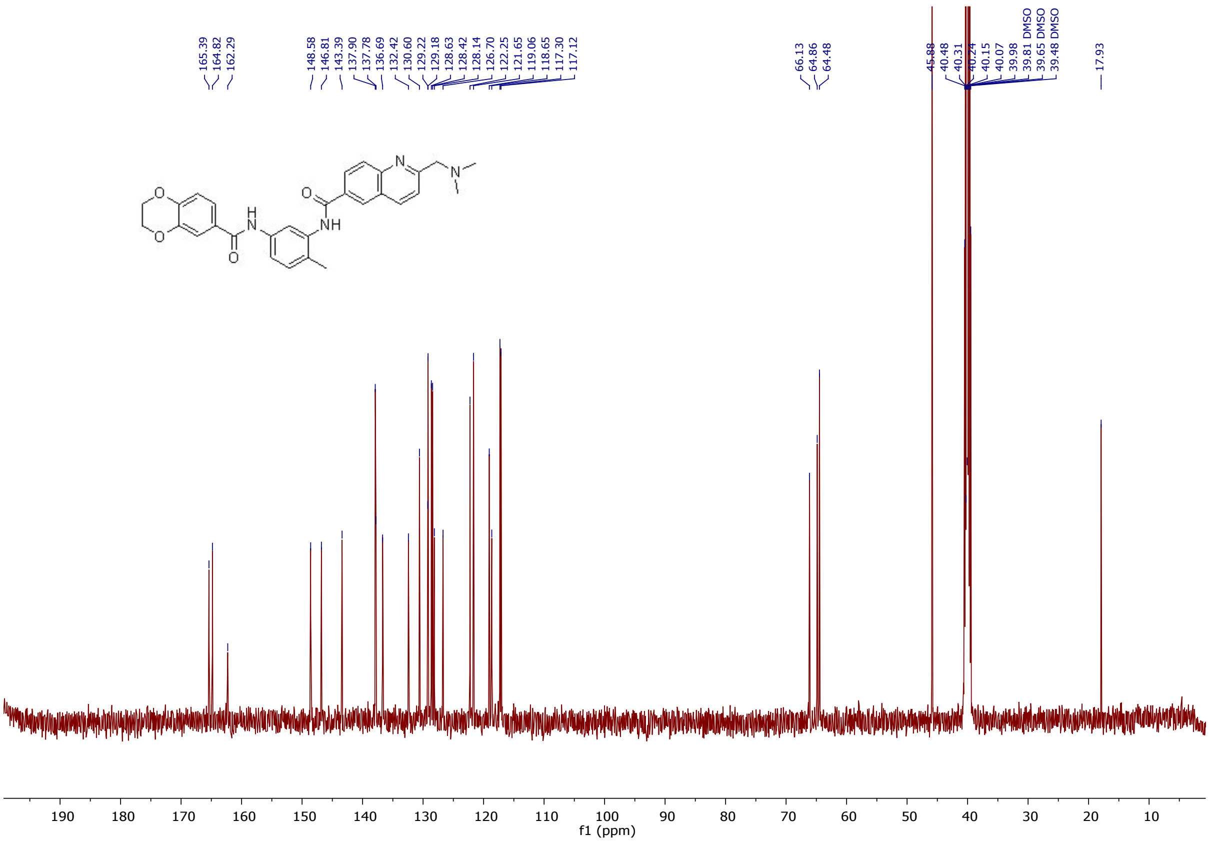

^{1}H NMR (MeOD, 500 MHz) and ^{13}C NMR (DMSO-d_6, 126 MHz) of compound **13**

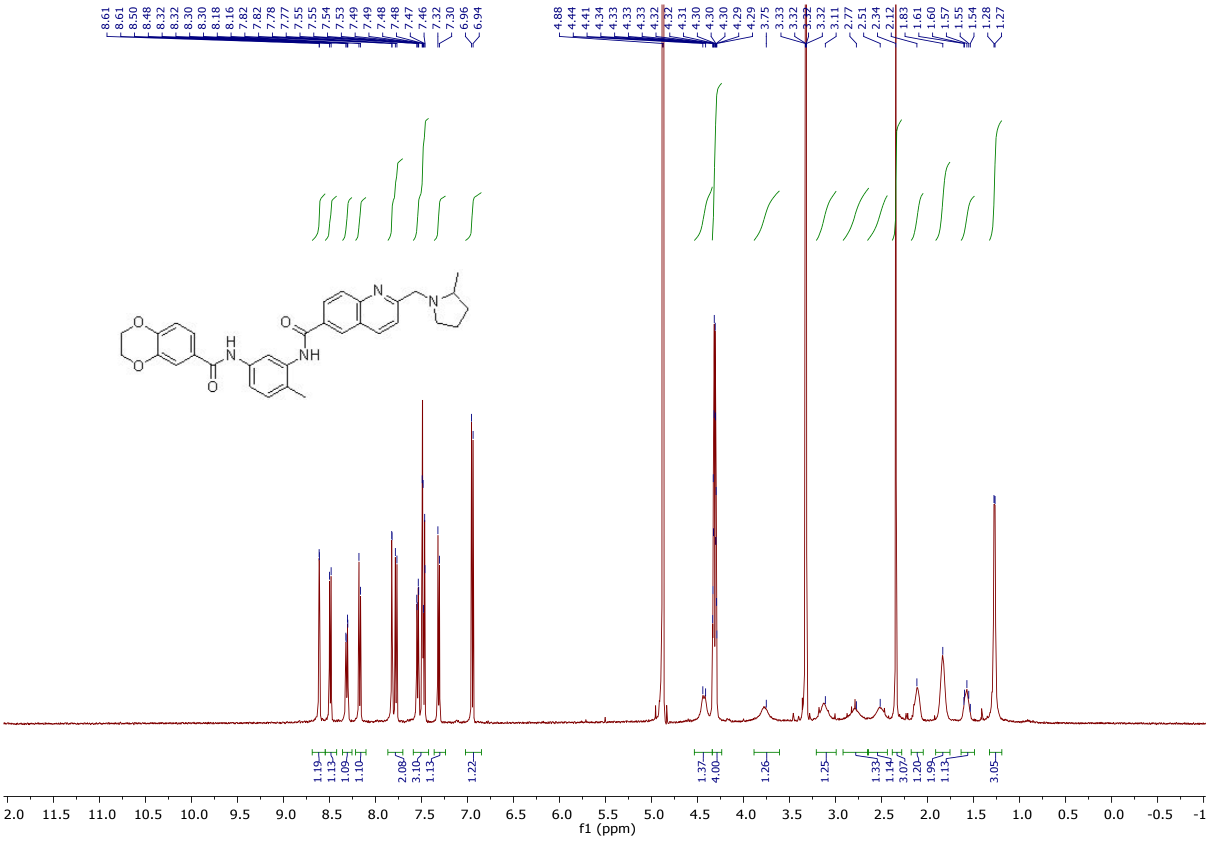

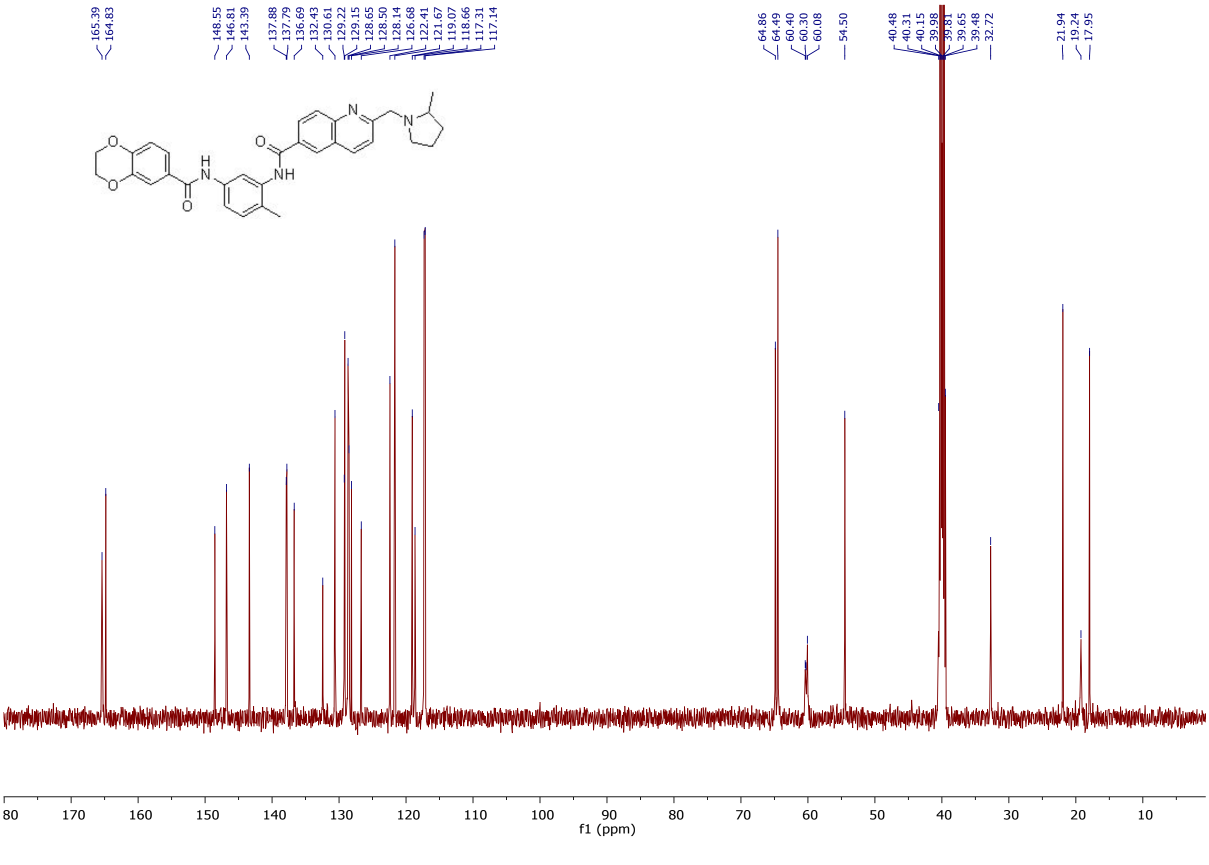

^{1}H NMR (DMSO-d$_6$, 500 MHz) and ^{13}C NMR (DMSO-d$_6$, 126 MHz) of compound **14**

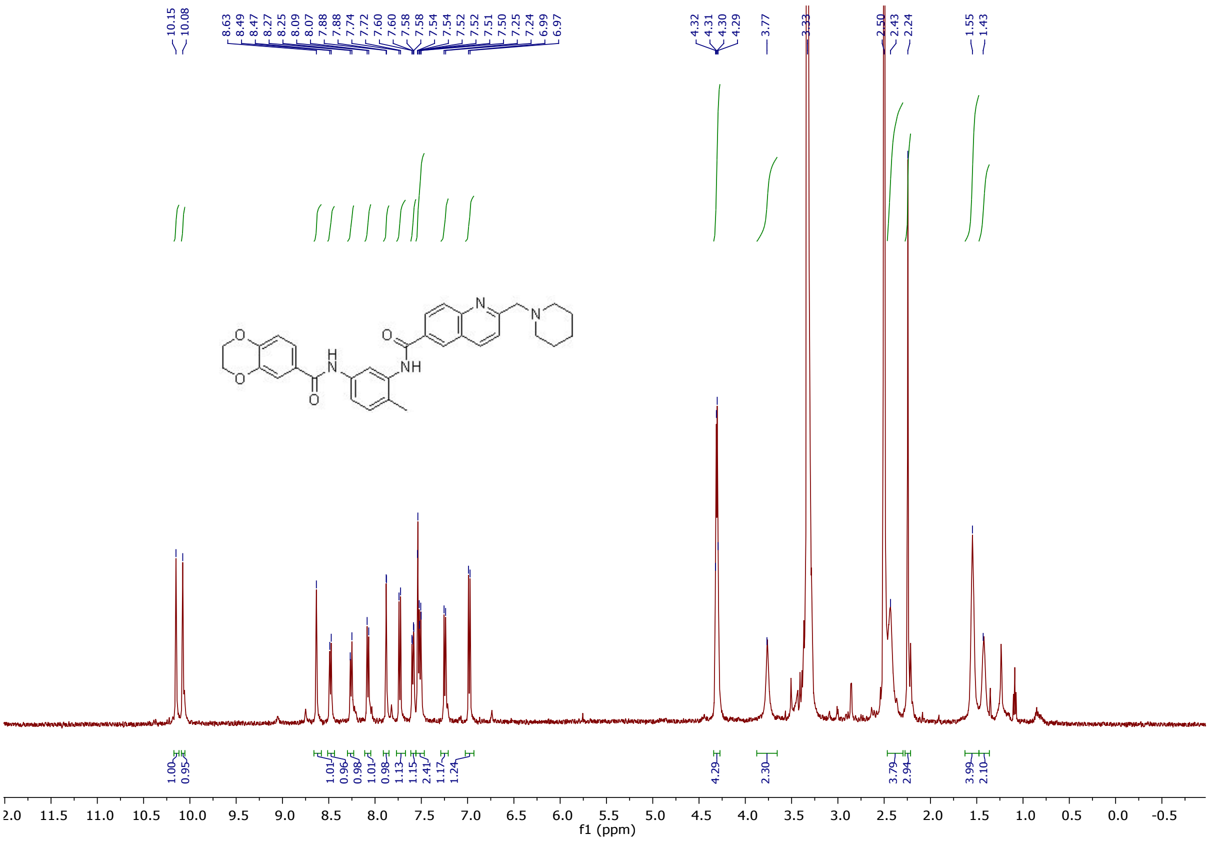

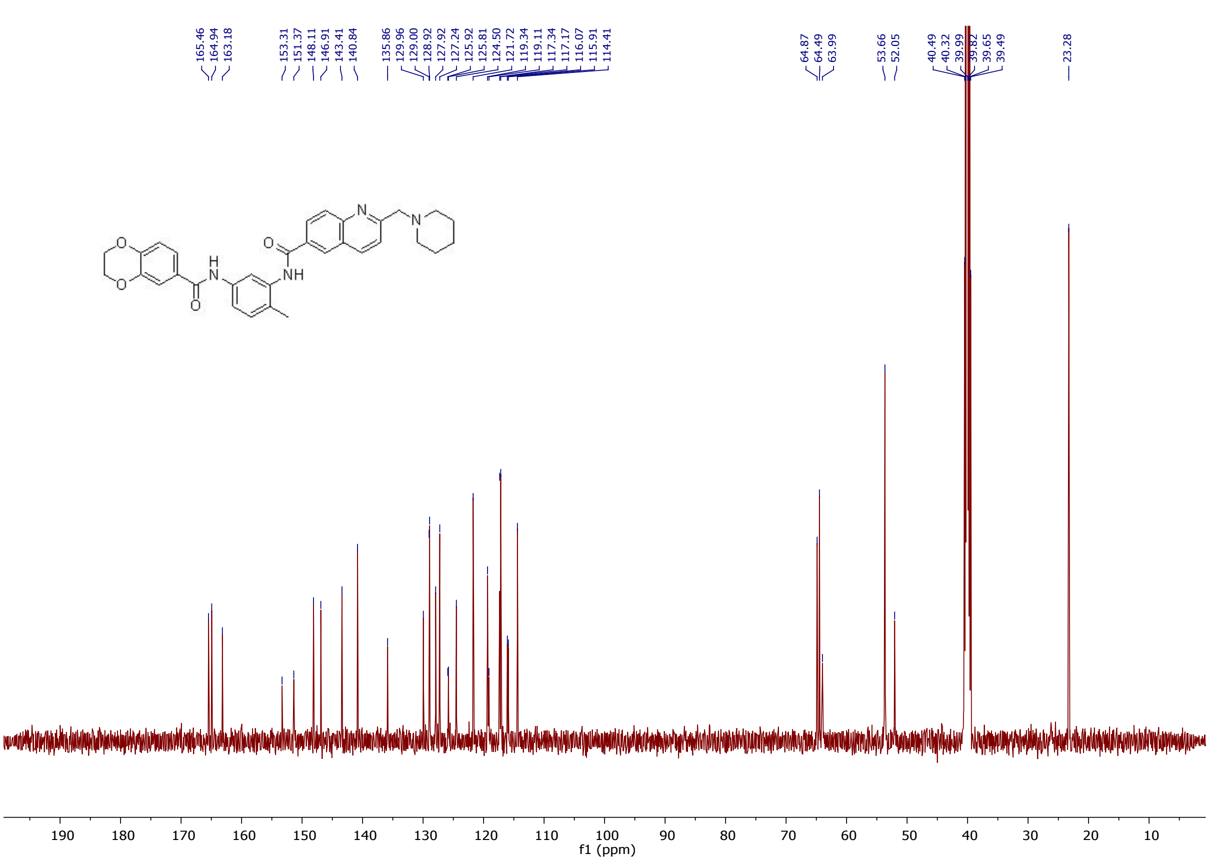

^{1}H NMR (DMSO-d_6, 500 MHz) and ^{13}C NMR (DMSO-d_6, 126 MHz) of compound **15**

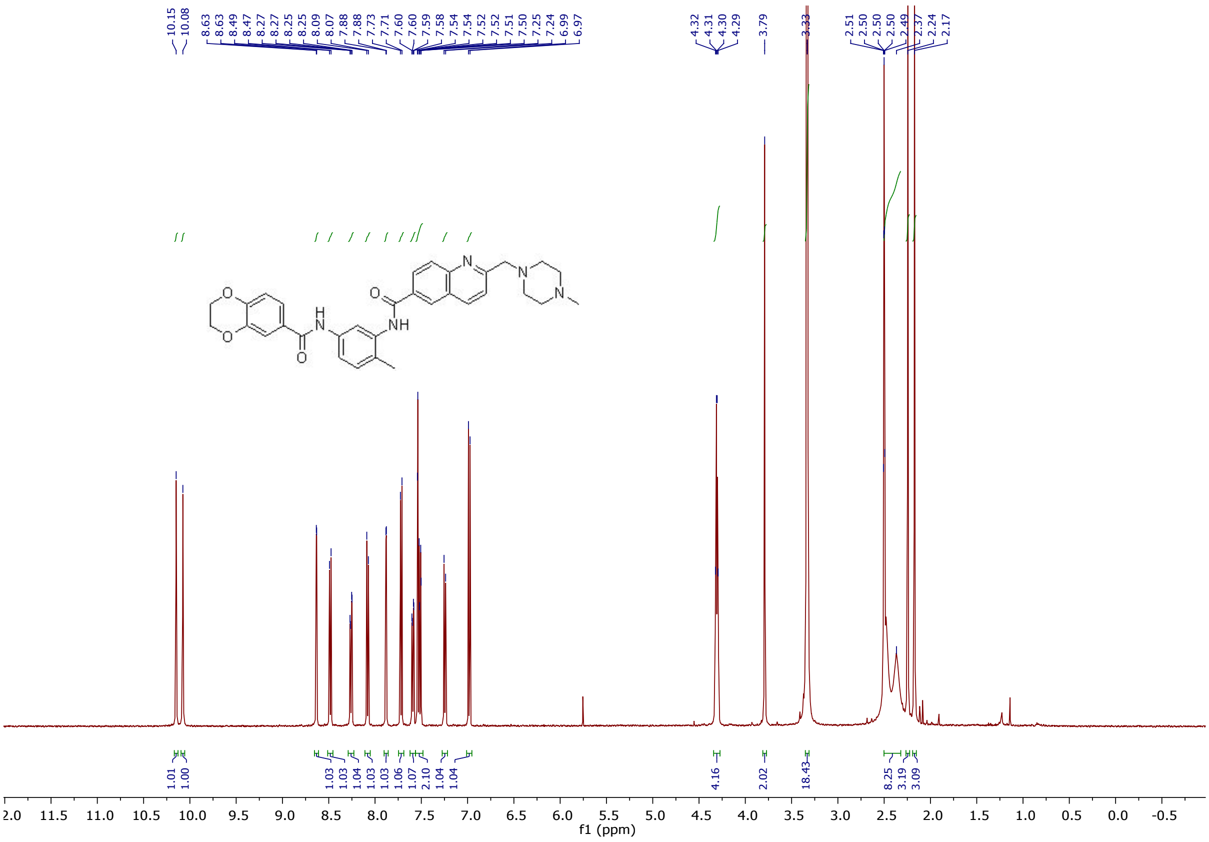

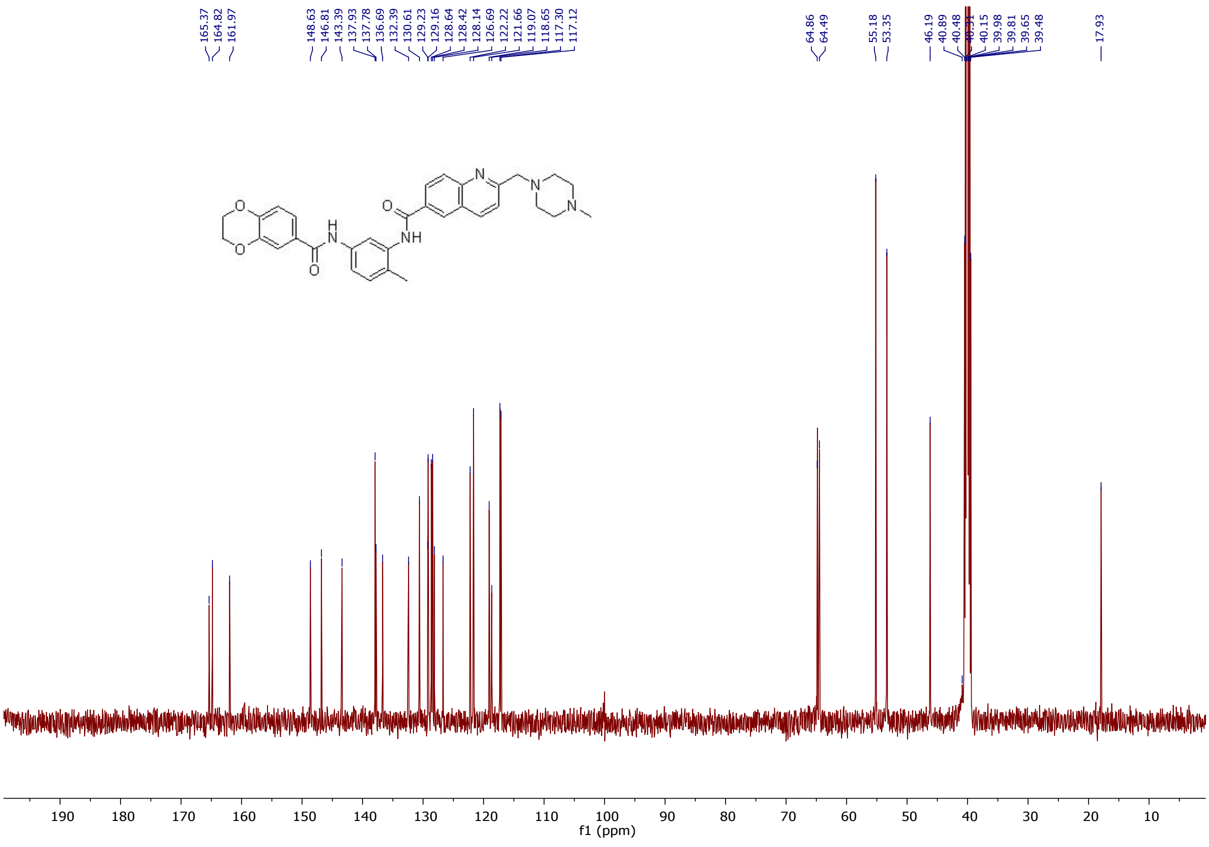

^{1}H NMR (DMSO-d_6, 500 MHz) and ^{13}C NMR (DMSO-d_6, 126 MHz) of compound **16**

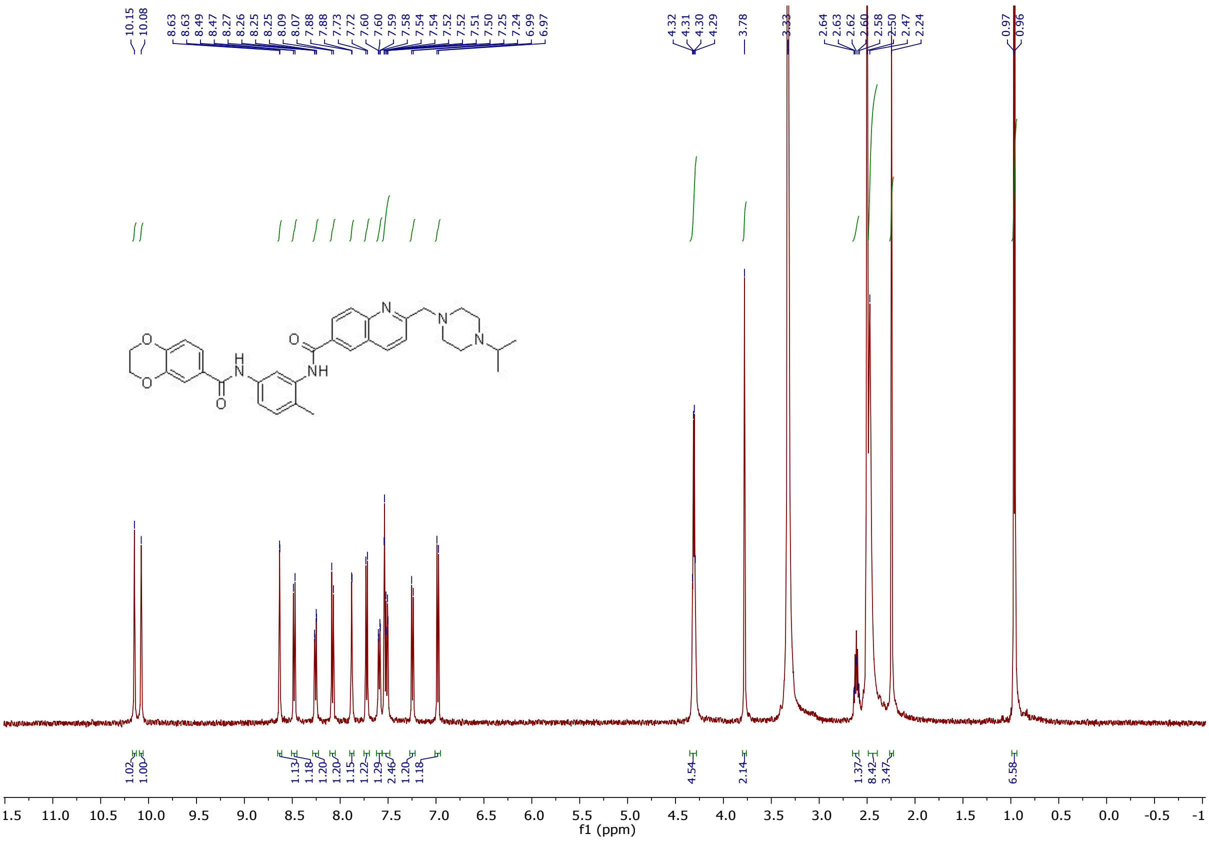

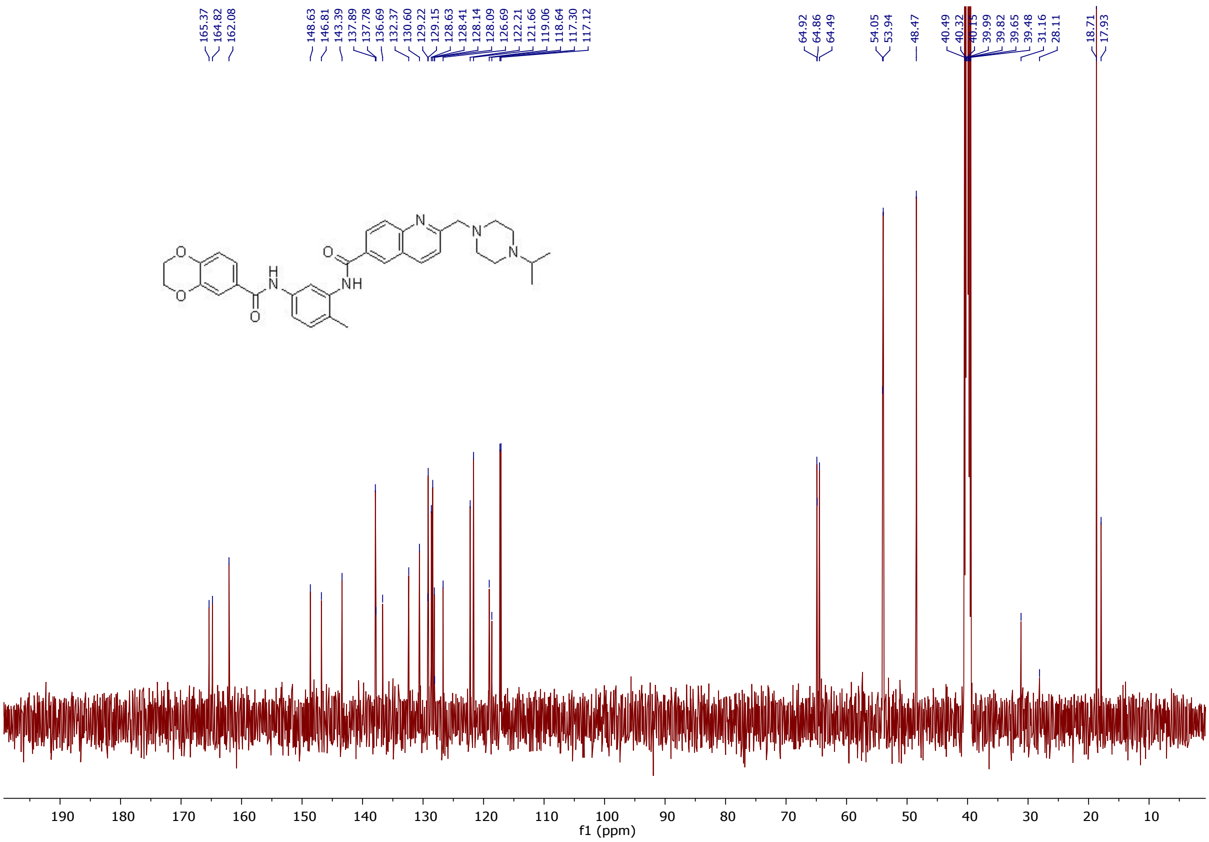

^{1}H NMR (DMSO-d_6, 500 MHz) and ^{13}C NMR (DMSO-d_6, 126 MHz) of compound **17**

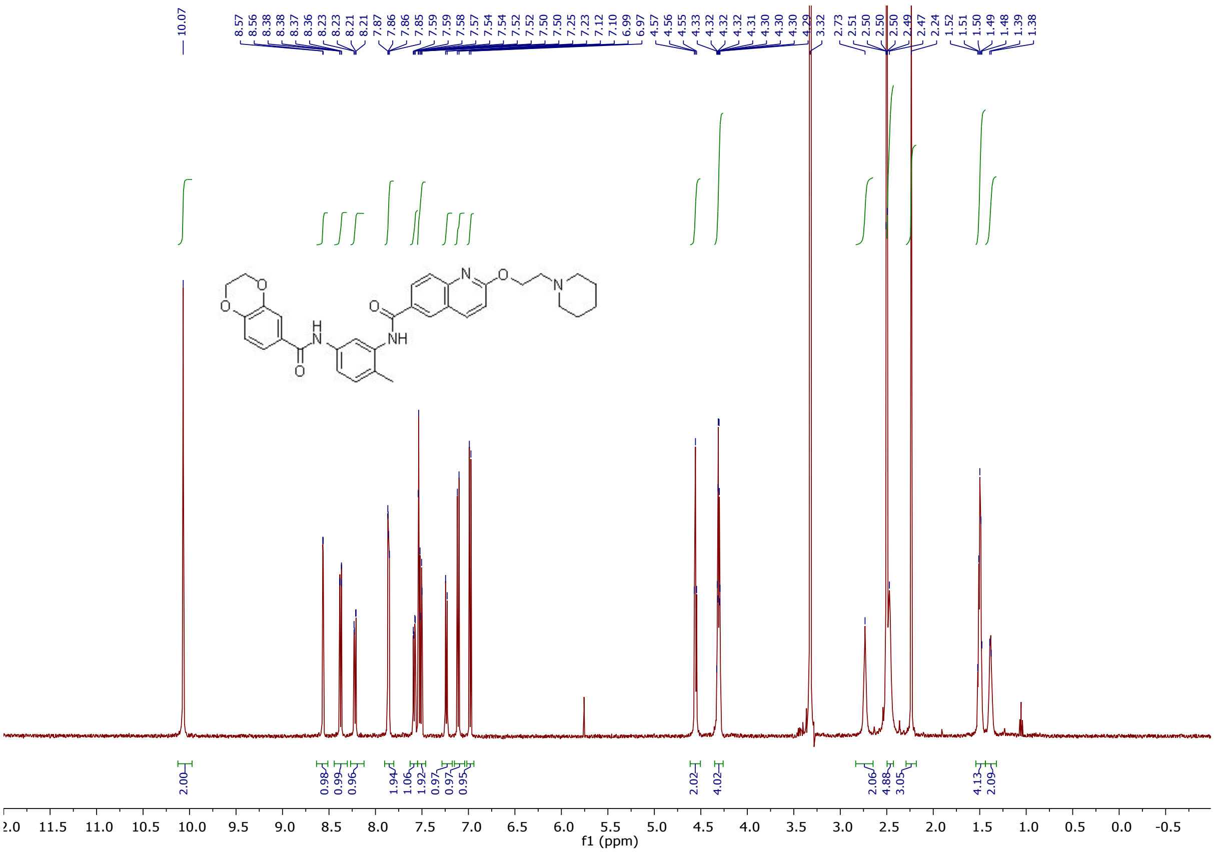

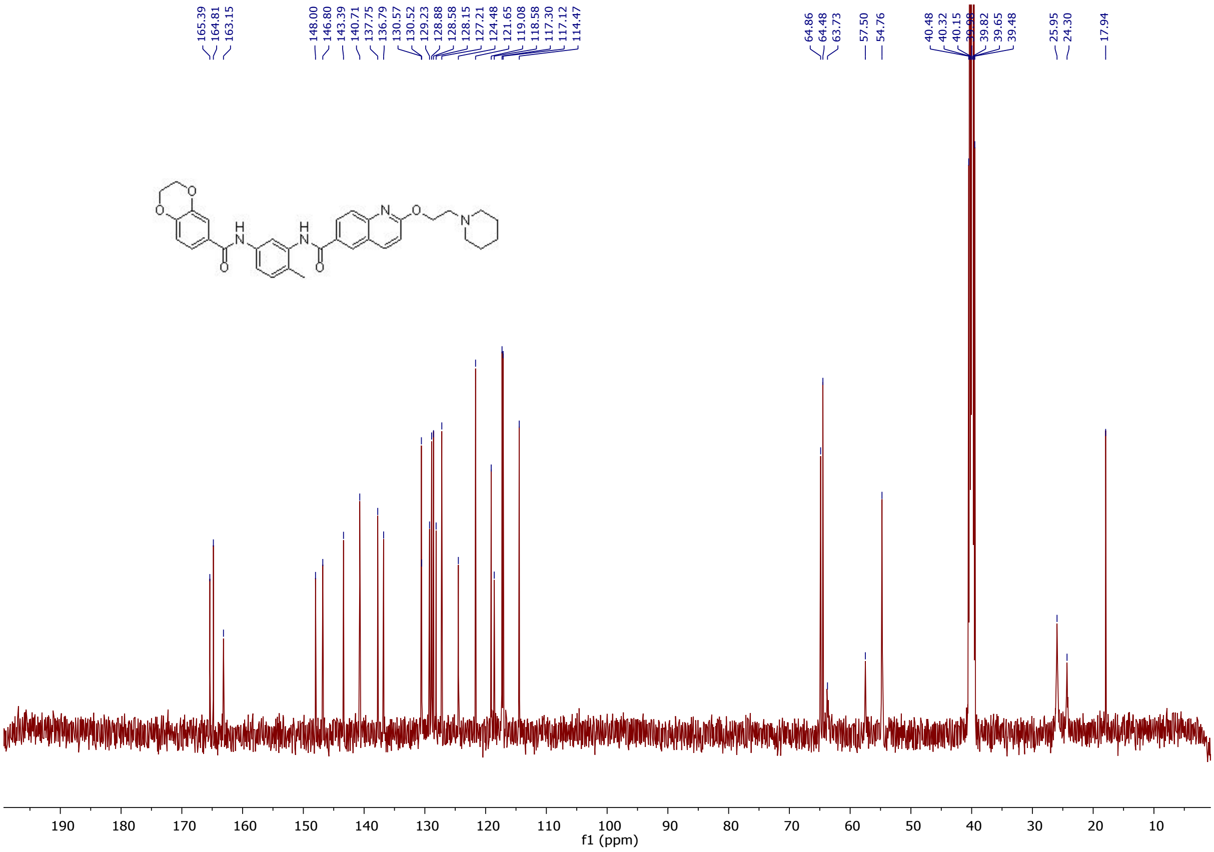

^{1}H NMR (DMSO-d_6, 500 MHz) and ^{13}C NMR (DMSO-d_6, 126 MHz) of compound **18**

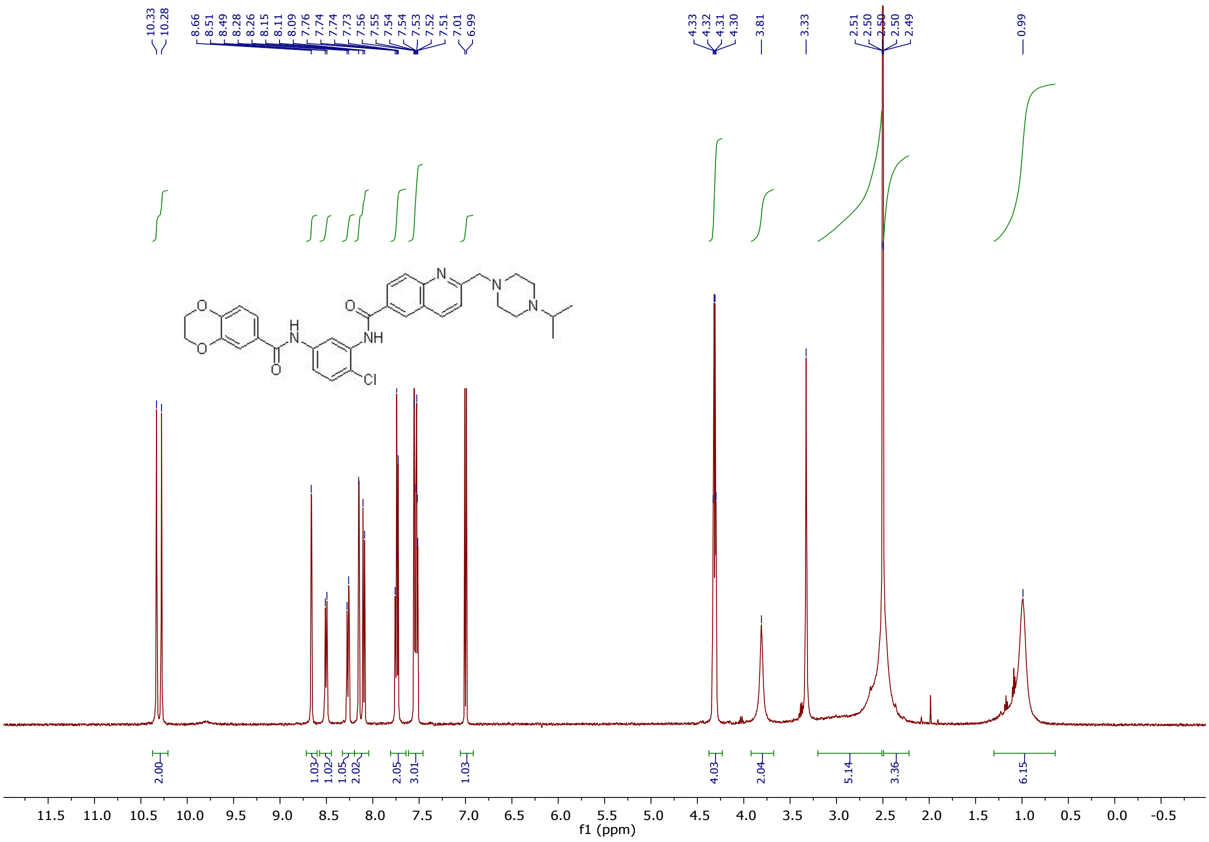

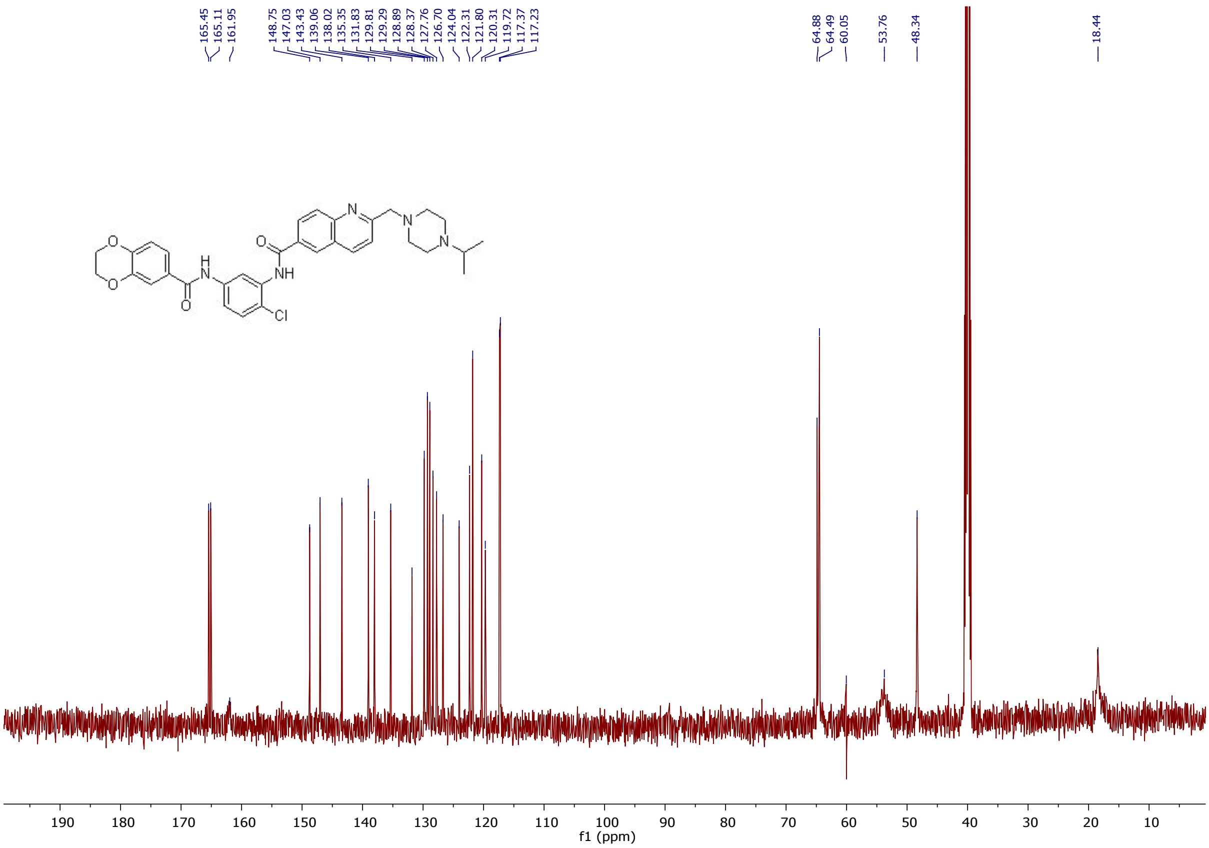

^{1}H NMR (DMSO-d$_6$, 500 MHz) and ^{13}C NMR (DMSO-d$_6$, 126 MHz) of compound **19**

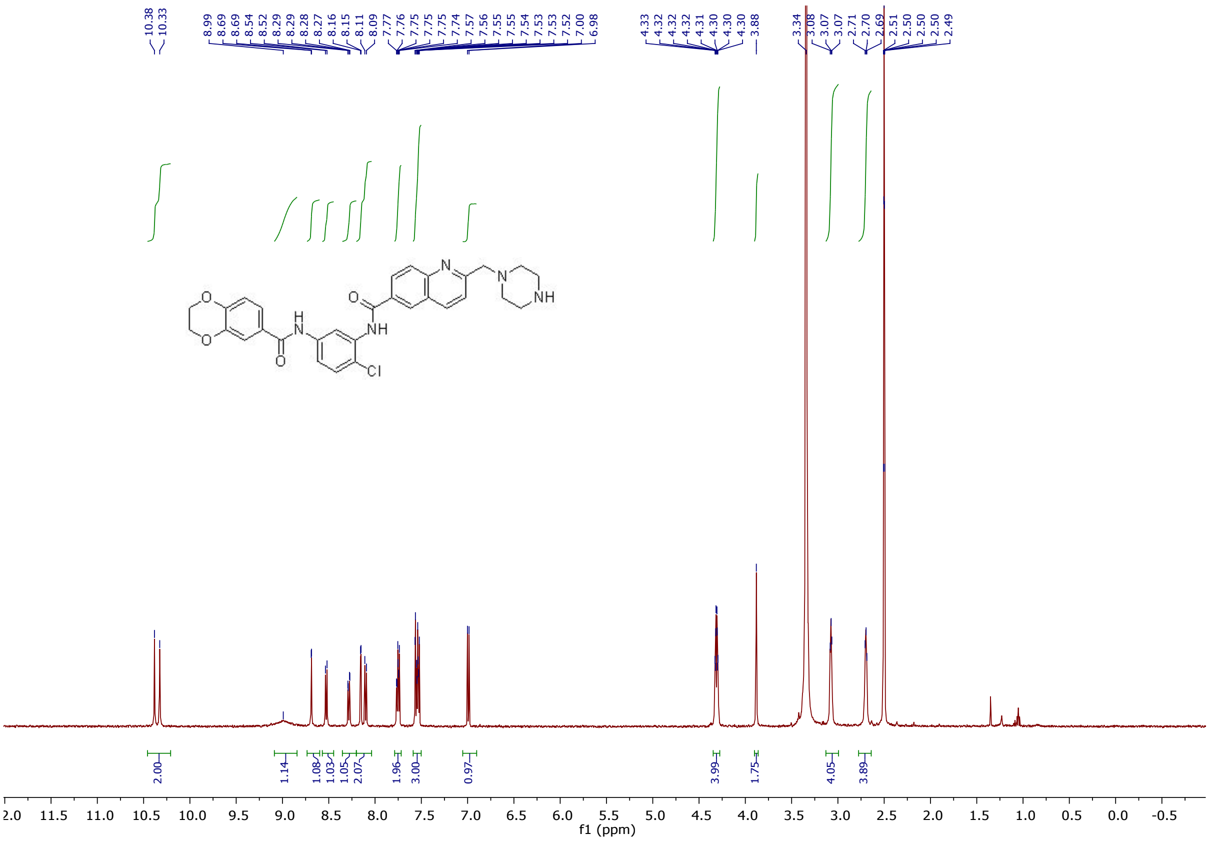

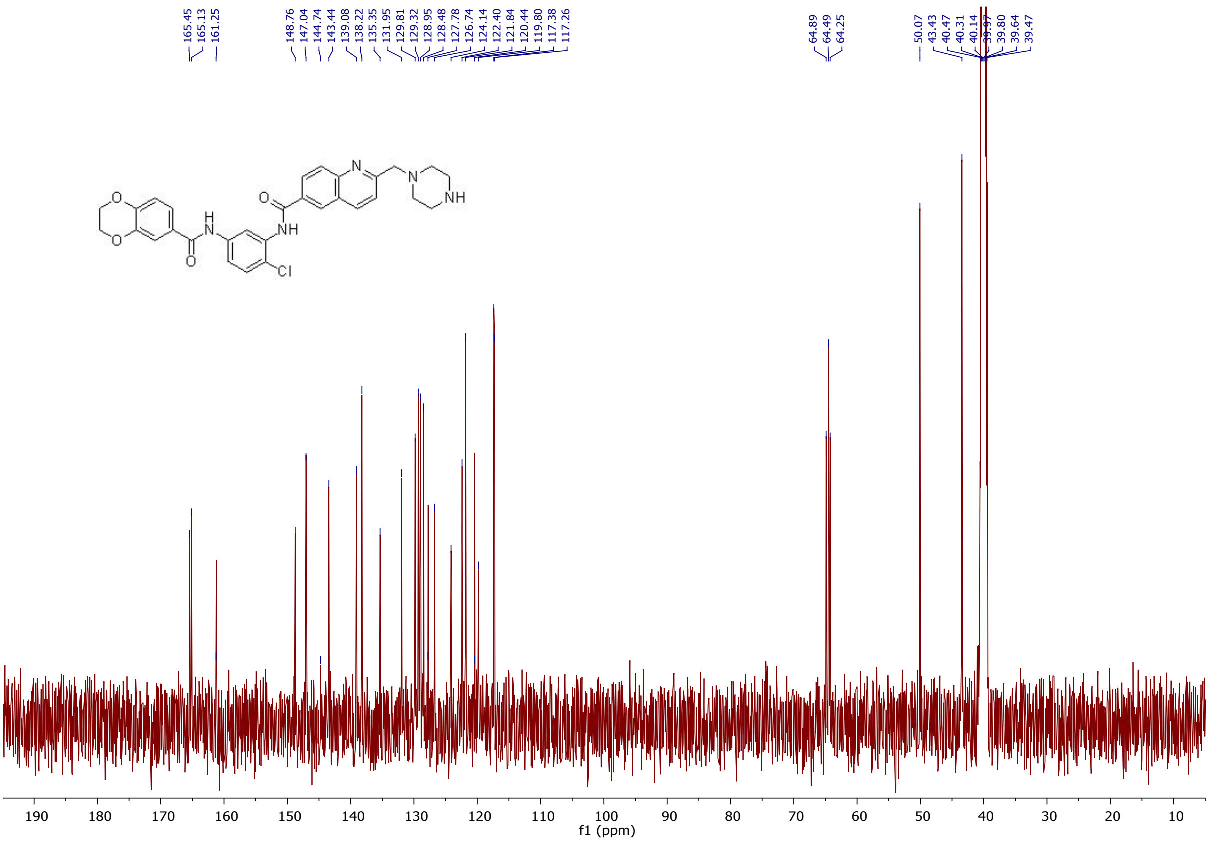

^{1}H NMR (DMSO-d_6, 500 MHz) and ^{13}C NMR (DMSO-d_6, 126 MHz) of compound **20**

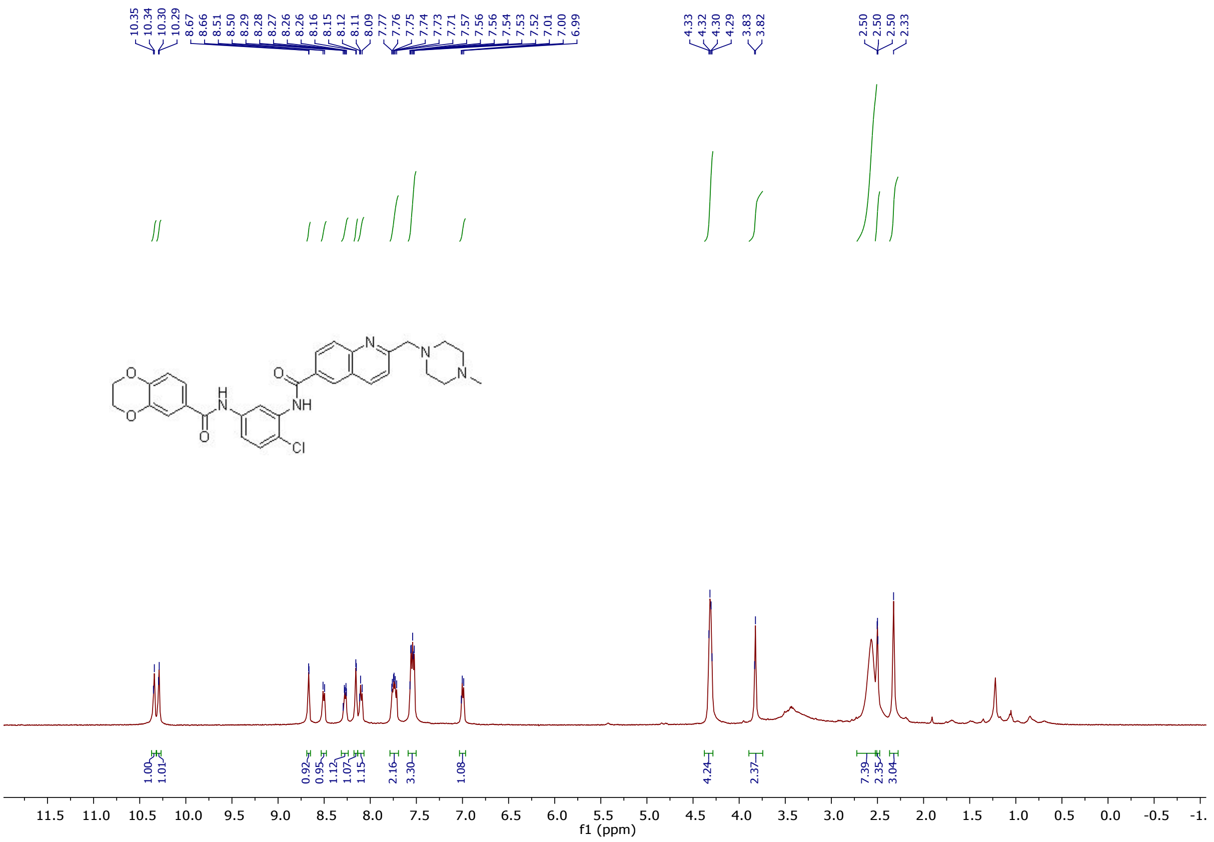

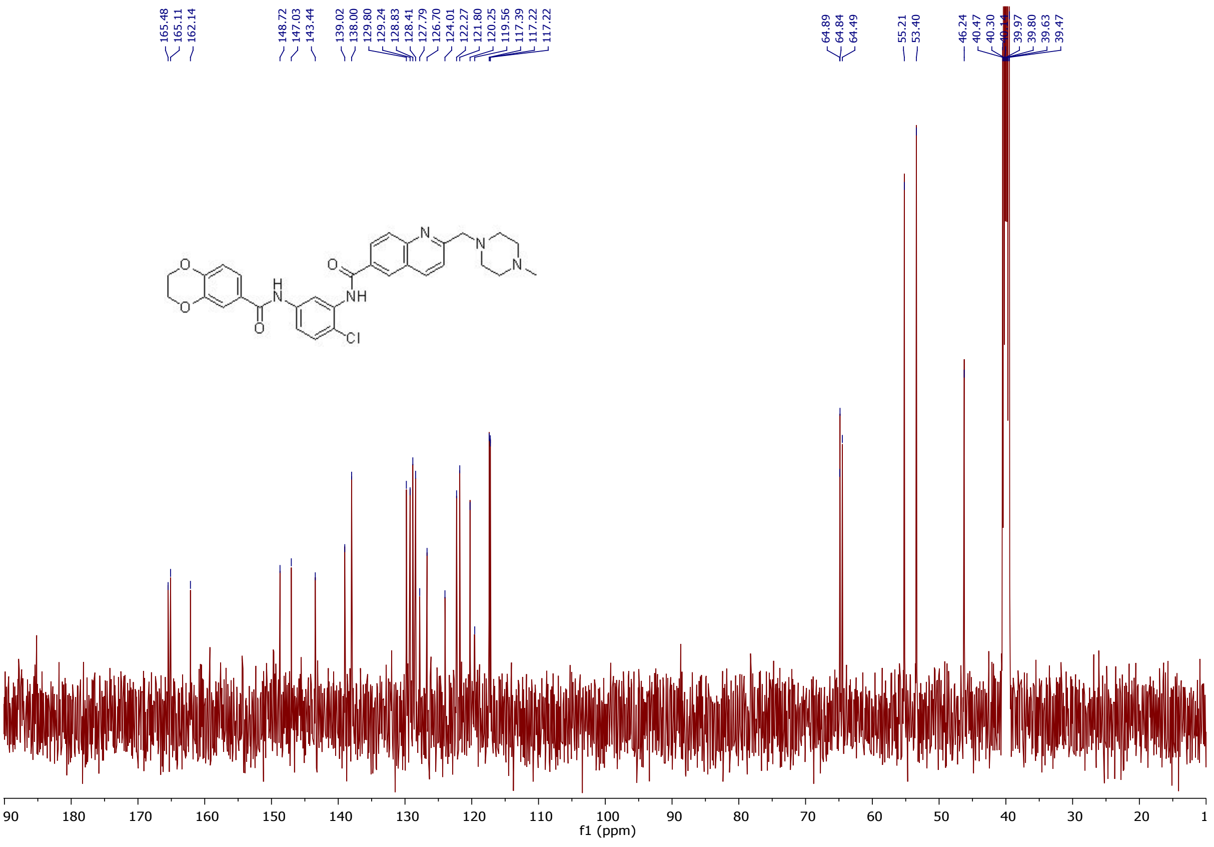

^{1}H NMR (DMSO-d_6, 500 MHz) and ^{13}C NMR (DMSO-d_6, 126 MHz) of compound **21**

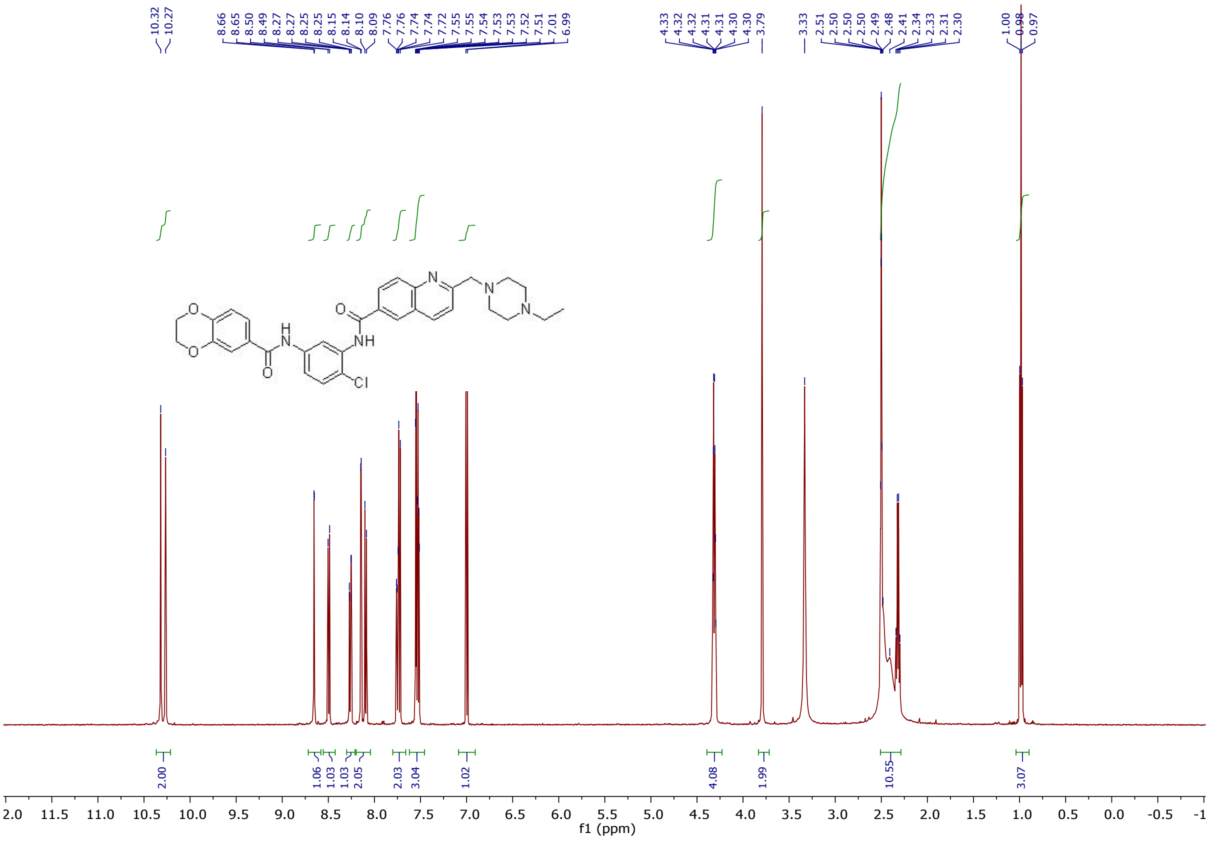

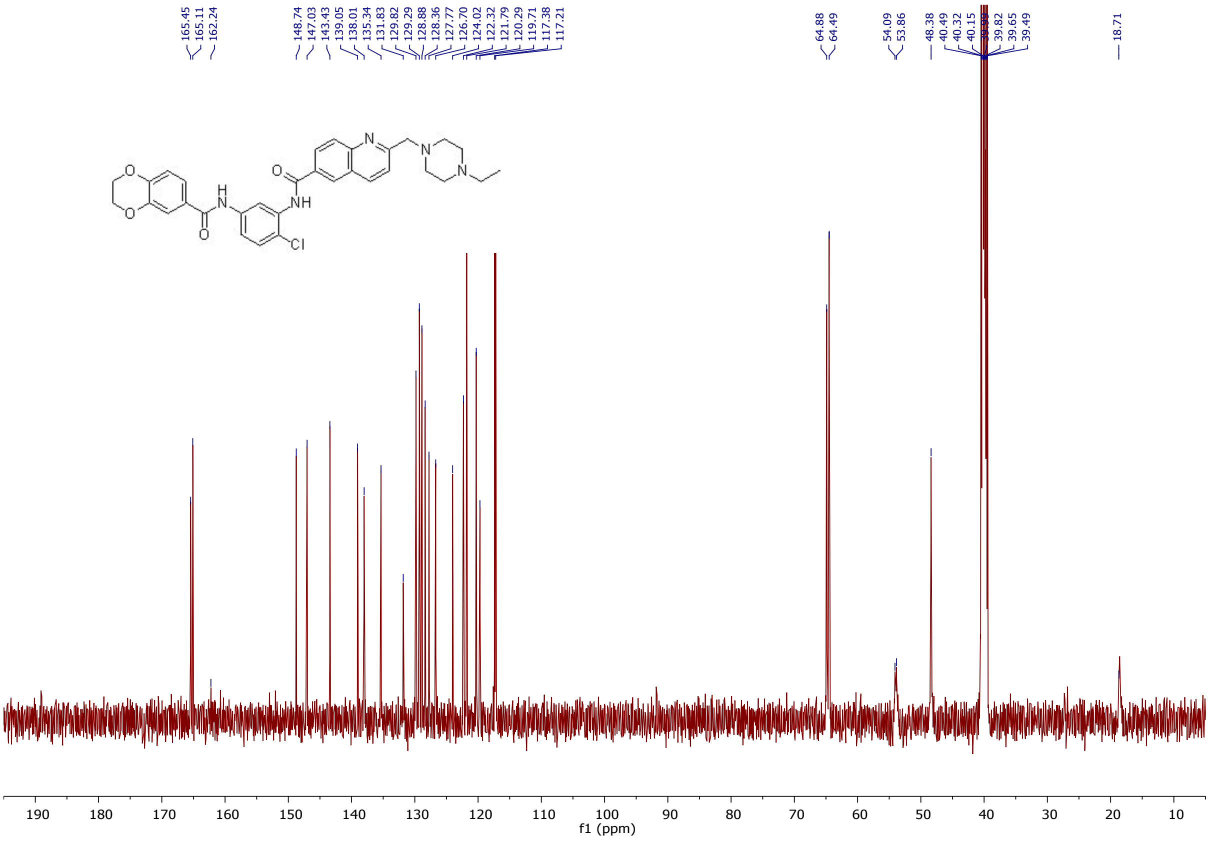

^{1}H NMR (DMSO-d_6, 500 MHz) and ^{13}C NMR (DMSO-d_6, 126 MHz) of CCT361814 (**22**)

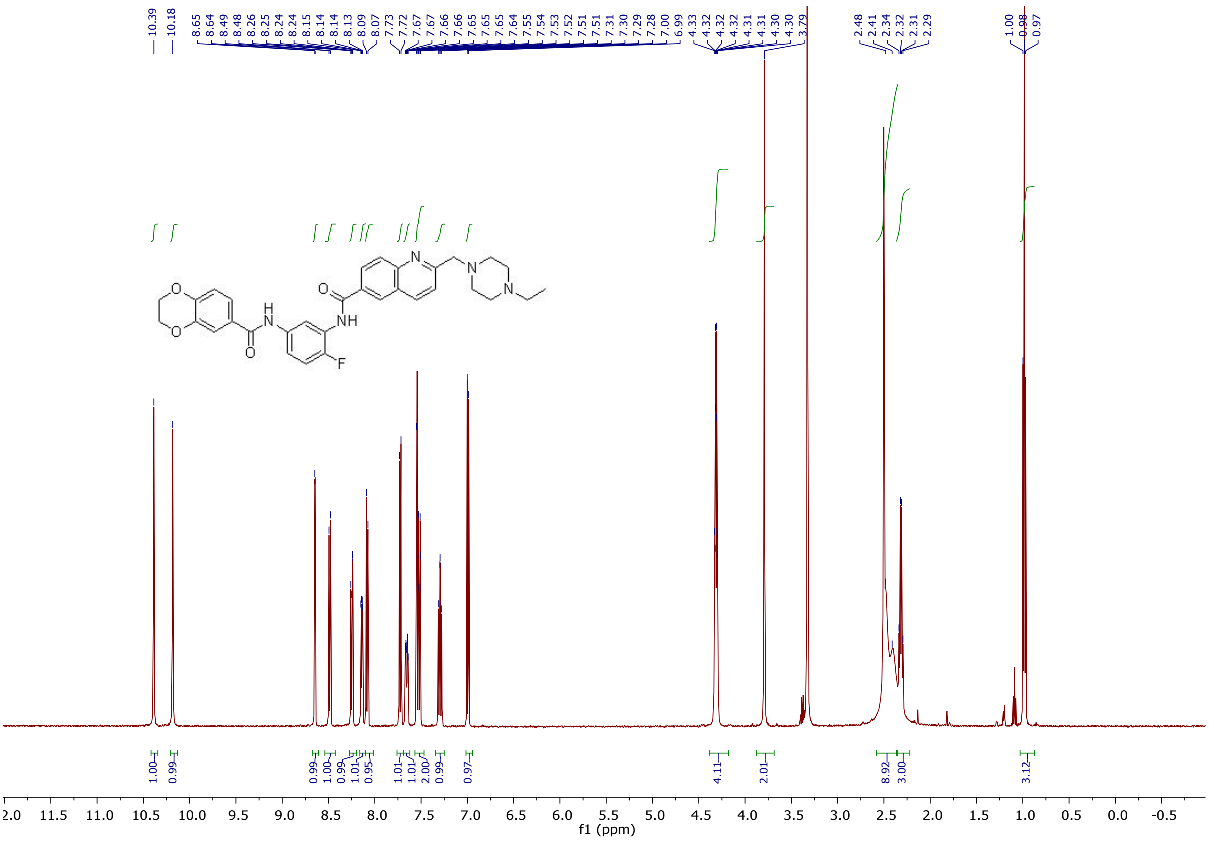

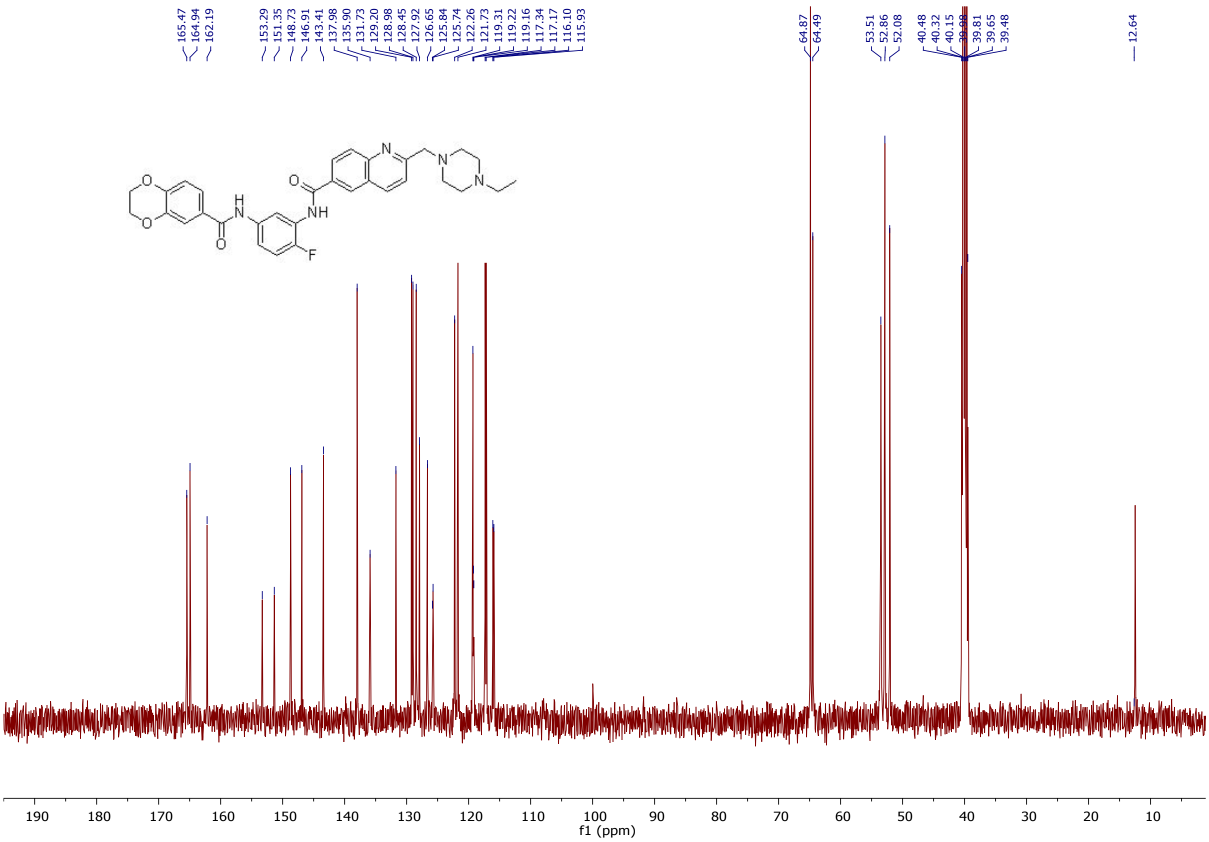

UV-Traces of in vivo Compounds

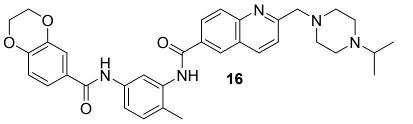

16

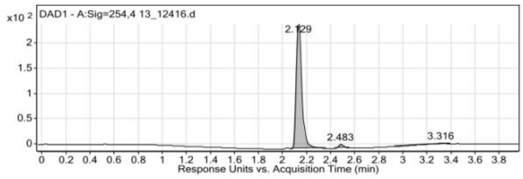

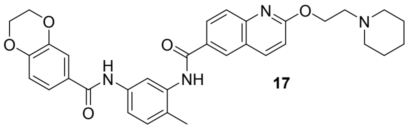

17

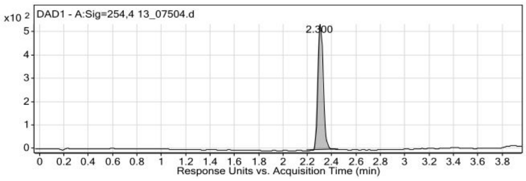

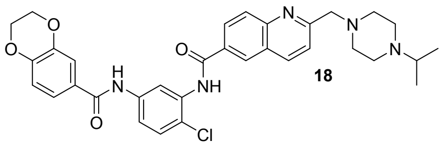

18

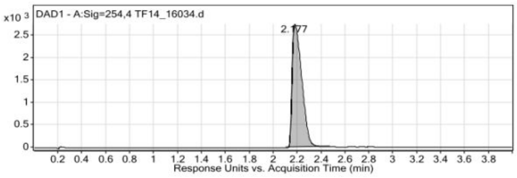

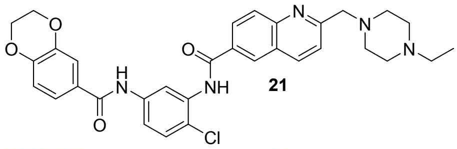

21

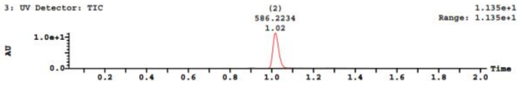

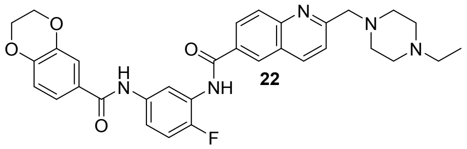

22

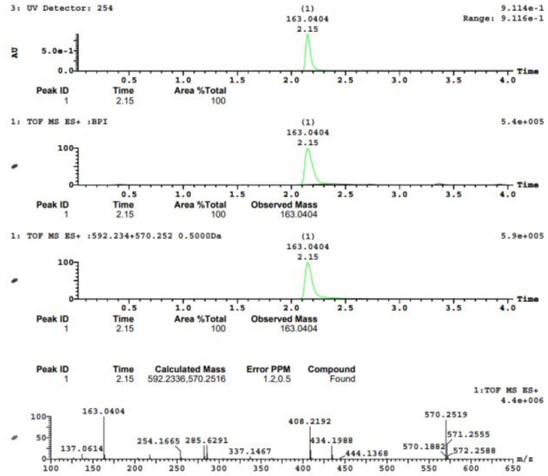

Physicochemical Properties Experimental

Reagents: HPLC grade solvents, formic acid, or alternative eluent modifiers were purchased from Sigma Aldrich (Poole, UK) unless otherwise stated.

$LogD_{7.4}$ Determination

Log $D_{7.4}$ values were determined via an in-house RP-HPLC method based on previous work by Kerns and Di.[6] Calibration was achieved by comparing the retention time of eight commercially available drugs with a range of Log $D_{7.4}$ values between -1.38 and 5.5, and correlating these retention times against literature Log $D_{7.4}$ values of the compounds. The calibration was validated by comparing HPLC-determined Log $D_{7.4}$ values of two other commercially-available drugs with literature Log $D_{7.4}$ values. The HPLC Log $D_{7.4}$ values of in-house compounds were determined by substituting the compounds' retention times into the equation obtained from the linear part of the calibration curve. Calibration, validation and in-house compounds were prepared at 1 mM in 10% DMSO/90% Trizma solution (100 mM Trizma in 75/25 methanol/water).
Two sets of 3 µL standard injections (with needle wash) of all calibration, validation and in-house samples were made onto a Phenomenex Luna C8 column (3 µm, 100 mm x 4.6 mm, 100A, Phenomenex, Torrence, USA). Chromatographic separation at 30 °C was carried out using a 1200 Series HPLC (Agilent, Santa Clara, USA) over a 5 minute gradient elution from 95:5 to 0:100 aqueous (20 mM Trizma in octanol-saturated water) and organic (acetonitrile + 0.25% v/v octanol) at a flow rate of 2 mL/min. The gradient was held at 0:100 water:organic for 0.8 minutes, then returned to the starting conditions of 95:5 water:organic for 0.2 minutes. The column was re-equilibrated for 5 minutes at the starting conditions prior to the next injection. UV-Vis spectra were acquired at 254 nm, 280 nm and 220 nm on a 1200 Series diode array detector (Agilent, Santa Clara, USA). Raw data was processed using Agilent Chemstation Rev.C.01.04.

Kinetic Solubility Determination

Kinetic Solubility values were determined via an in-house RP-HPLC method. Calibration standards were prepared at a concentration of 100 µM in 100% DMSO. Calibration was achieved by injecting 0.5, 2.5 and 5 µL of the 100 µM standard. Kinetic solubility samples were prepared at a concentration of 100 µM in 1% DMSO/99% PBS (pH 7.4). The samples were shaken for 120 min at room temperature and then centrifuged for 15 min at 14,000 rpm. The supernatant was removed and a small volume of DMSO was added to ensure the compound remained in solution. Kinetic solubility values were determined by substituting the AUC of the kinetic solubility samples into the equation obtained from the linear part of the calibration curve.
Injections (with needle wash) of the calibration and kinetic solubility samples were made onto a Phenomenex Kinetex C18 column (5 µm, 50 mm x 4.6 mm, 100A, Phenomenex, Torrence, USA). Chromatographic separation at 30 °C was carried out using a 1260 Series HPLC (Agilent, Santa Clara, USA) over a 5 minute gradient elution from 90:10 to 10:90 water:methanol (both modified with 0.1% formic acid) at a flow rate of 1.5 mL/min. UV-Vis spectra were acquired at 254 nm on a 1260 Series diode array detector (Agilent, Santa Clara, USA). Raw data was processed using Agilent Chemstation Rev.C.01.05.8. The data was then reprocessed on ChemStation C.01.04 to generate the calibration curve and solubility value.

Thermodynamic Solubility Determination for compound CCT361814 (22)

Fasted State Simulated Gastric Fluid (FaSSGF, Pharmidex)
http://www.pharmidex.com/service/physiocochemical-properties, February 2017
Method:
Aqueous thermodynamic solubility was measured at a concentration of 1 mg/mL. Compounds were equilibrated in Fasted State Simulated Gastric Fluid (FaSSGF), pH 1.6.
Aqueous thermodynamic solubility was measured at 37 °C. The test solutions are allowed to equilibrate for 4 h. Centrifugation at 15,000 g for 10 min. A 100 µL sample of the supernatant is carefully collected and the sample quantified using the analytical method. The standard curve for each compound was prepared in 100% acetonitrile. Soluble amount is determined by LC-MS/MS. The results spreadsheet allows calculation of solubility from each of the aqueous dilutions. Test samples read off calibration curve. Provision of solubility in mg/ml. Results are reported as low (less than 0.1 mg/mL), medium (0.1 – 0.5 mg/mL) and high (>0.5 mg/mL).

Table S16. FaSSGF thermodynamic solubility

Compd	mg/mL	Concentration (mg/mL)			Mean	SD	SEM	n	CV	CV(%)
		1	2	3						
CCT361814	1	0.975	0.854	0.930	**0.920**	0.061	0.035	3	0.07	6.66
Pimozide	1	0.087	0.092	0.090	**0.090**	0.003	0.001	3	0.03	2.80
Propranolol	1	1.246	1.094	1.002	**1.114**	0.123	0.071	3	0.11	11.06

Fasted State Simulated Intestinal Fluid (FaSSIF, Pharmidex)
http://www.pharmidex.com/service/physiocochemical-properties, February 2017
Method:
Aqueous thermodynamic solubility was measured at a concentration of 1 mg/mL. Compounds were equilibrated in Fasted State Simulated Intestinal Fluid (FaSSIF), pH 6.5. Aqueous thermodynamic solubility was measured at 37 °C. The test solutions are allowed to equilibrate for 4 h. Centrifugation at 15,000 g for 10 min. A 100 µL sample of the supernatant is carefully collected and the sample quantified using the analytical method. The standard curve for each compound was prepared in 100% acetonitrile. Soluble amount is determined by LC-MS/MS. The results spreadsheet allows calculation of solubility from each of the aqueous dilutions. Test samples read off calibration curve. Provision of solubility in mg/mL. Results are reported as low (less than 0.1 mg/mL), medium (0.1 – 0.5 mg/mL) and high (>0.5 mg/mL).

Table S17. FaSSIF thermodynamic solubility

Compd	mg/mL	Concentration (mg/mL)			Mean	SD	SEM	n	CV	CV(%)
		1	2	3						
CCT361814	1	0.029	0.025	0.026	**0.027**	0.002	0.001	3	0.09	8.73
Pimozide	1	0.004	0.003	0.003	**0.004**	0.001	0.000	3	0.21	21.03
Propranolol	1	0.853	1.047	0.931	**0.944**	0.098	0.056	3	0.10	10.36

Fed State Simulated Intestinal Fluid (FeSSIF, Pharmidex)
http://www.pharmidex.com/service/physiocochemical-properties, February 2017
Method:
Aqueous thermodynamic solubility was measured at a concentration of 1 mg/mL. Compounds were equilibrated in Fed State Simulated Intestinal Fluid (FeSSIF), pH 6.5. Aqueous thermodynamic solubility was measured at 37 °C. The test solutions are allowed to equilibrate for 4 h. Centrifugation at 15,000 g for 10 min. A 100 µL sample of the supernatant is carefully collected and the sample quantified using the analytical method. The standard curve for each compound was prepared in 100% acetonitrile. Soluble amount is determined by LC-MS/MS. The results spreadsheet allows calculation of solubility from each of the aqueous dilutions. Test samples read off calibration curve. Provision of solubility in mg/mL. Results are reported as low (less than 0.1 mg/mL), medium (0.1 – 0.5 mg/mL) and high (>0.5 mg/mL).

Table S18. FeSSIF thermodynamic solubility

Compd	mg/mL	Concentration (mg/mL)			Mean	SD	SEM	n	CV	CV(%)
		1	2	3						
CCT361814	1	0.035	0.039	0.029	**0.034**	0.002	0.005	3	0.14	14.30
Pimozide	1	0.018	0.008	0.007	**0.004**	0.011	0.006	3	0.59	59.30
Propranolol	1	1.669	1.812	1.465	**0.944**	1.649	0.174	3	0.11	10.58

Biology Experimental Procedures

Origin of the human ovarian carcinoma cell lines used in this study: SK-OV-3 was obtained from an ICR collaborator in the 1990s. It was passaged in vivo in athymic mice to increase its tumorigenicity and reliability as a xenograft, and used within 10 passages of banked stocks. Prior to use, the cells were analyzed by short tandem repeat (STR) profiling. Polymorphic STR loci were amplified using a polymerase chain reaction (PCR) primer set. The PCR product (each locus being labelled with a different fluorophore) was analyzed simultaneously with size standards using automated fluorescent detection. The number of repeats at 10 different loci (as recommended by the American Type Culture Collection, ATCC) was used to define the STR profile and this was cross-referenced with online databases to confirm authenticity. Using this method, the in vivo subline showed an acceptable 85.71 % identity with the ATCC reference line (LGC Promochem, UK). The cells were free of mycoplasma contamination as determined by a sensitive nested PCR protocol (Venor GeM kit, Minerva Biolabs, Germany). Cells were grown in DMEM/10 % FCS, 2 mM glutamine and nonessential amino acids in 5% CO_2. $CH1^{wt}$ was established in-house from an ascites sample (Hills *et al. Br. J. Cancer*, **1989**, *59*, 527-534). $CH1^{doxR}$ was established following the procedure from Sharp *et al. Br. J. Cancer*, **1994**, *70*, 409-414. IGROV1 was obtained from NCI cell bank in Frederick, USA. OVCAR3, CaOV3 and PA1were obtained from ATCC. OVCAR5 andOVCAR8 were obtained from the National Cancer Institute, New York.RMG1 was obtained from Health Science Research Resources Bank.

In vitro cell viability assay
The CellTiter-Blue viability (Promega) assay provides a homogenous, fluorometric method for estimating the number of viable cells. It uses the dark blue indicator dye resazurin to measure the metabolic capacity of cells which is an indicator of cell viability. Viable cells are able to reduce resazurin into resorufin (pink), which is highly fluorescent. Briefly, cells (~6 x 10^3 cells/mL) were seeded into 384-well plates and were incubated for 24 h. Compounds (at a range of concentrations) were added using the ECHO 550 liquid handler (Labcyte, USA) and then left at 37 °C for 96 h. Titer-Blue reagent was added to each well and left at 37 °C for 3-4 h. Fluorescence was measured using the Envision machine (Perkin Elmer, UK). The 50% growth inhibitory concentration (GI_{50}) was determined by fitting the data to a dose-response curve without limits using non-linear regression. Each concentration was tested twice.

$CH1^{doxR}$/$CH1^{wt}$ MDR Assay
The antiproliferative activity of compounds was assessed in $CH1^{wt}$ and $CH1^{doxR}$ cells using the same method described previously using the CellTiter-Blue viability assay. The rescue of the antiproliferative activity in the $CH1^{doxR}$ cell line was confirmed by treating the cells with 2 µM (*R*)-(+)-verapamil monohydrochloride hydrate (http://www.sigmaaldrich.com/catalog/product/sigma/v106?lang=en&region=GB, February 2017) and the bisamide analogue. The geometric mean pGI_{50} values (pGI_{50}=-log GI_{50} (M)) of at least n=3 biological repeats in the $CH1^{wt}$ and $CH1^{doxR}$ cells for each were then compared using a Student's t-test with Welch's correction; when $p<0.05$ the compound considered to be an MDR substrate (GraphPad Prism 7.01). The ratio of geometric mean GI_{50}s in $CH1^{doxR}$ and $CH1^{wt}$ was defined as the MDR ratio.

In vivo Studies
Experimental work was done in accordance with UK Home Office regulations under the Animals (Scientific Procedures) Act 1986, ICR ethical review processes and according to the UK National Cancer Research CRI Guidelines with local Ethical Committee approval.[7]
Compound **22** was dissolved in 10% DMSO and diluted in 90% sterile solvent (25% w/v hydroxypropyl β-cyclodextrin in 50 mM sodium citrate buffer pH 5) such that mice received the dose required in 0.1 mL of final solution per 10 g body weight. Controls received an equal volume of vehicle only. For multi-dose tolerability studies, NCr athymic mice (n=2 per cohort) were administered 50 mg/kg or 100 mg/kg of compound **22** orally every day for five days. Mice were monitored for signs of distress and body weights were measured daily until full recovery was observed. Dosing at 100 mg/kg of compound **22** was not tolerated and, therefore, was terminated at day 4.
For efficacy studies, SK-OV-3 cells (5 million per site) were injected s.c. in the flanks of 6- to 8-week-old female NCr athymic mice (n=20). Dosing commenced when tumors were well established (~5-6 mm diameter). Tumor volumes were determined as previously described. On study termination, blood samples were taken, and plasma was separated and stored at -80 °C.

CHAC1 Western Blot and MSD Assays
Tumors were snap frozen in liquid nitrogen and stored at -80 °C until processed. Tumors were lysed in 50 mM Tris-HCl (pH 7.4), 1 mM NaCl, 1 mM EDTA, 1% Triton X-100, 1 mM NaF, 1 mM sodium vanadate (activated), 10 µg/mL N_α-tosyl-L-lysine chloromethyl ketone hydrochloride, 5 µM fenvalerate, 5 µM bpVphen, 1 mM phenylmethanesulfonyl fluoride, 1:100 protease cocktail (Sigma P8340) and 1:50 of phosphatases inhibitor 2 and 3 (Sigma P2850). Protein concentration was determined by Direct Detect® Infrared Spectrometer (MerckMillipore).
Each lysate was separated by SDS-PAGE, electrotransferred onto PVDF membranes, blocked with 5% milk (Marvel) and probed with specific primary antibody CHAC1 (Santa-Cruz 133320 at 1:100 dilution) and horseradish peroxidase-conjugated secondary (1:1000) antibody (Cell Signalling Cambridge). Signal was detected with enhanced chemiluminescence reagent (Amersham Biosciences). Glyceraldehyde-3-phosphate dehydrogenase (Abcam 1:20000 dilution) was used as the loading control.
All reagents were purchased from Sigma-Aldrich, except bpVphen from MerckMillipore.
Initially 14 antibodies (Ab184982, ab155533, ab180380: purchased from Abcam; HPA043505: purchased from Sigma; SC382544: purchased from Santa-Cruz; TA507010, TA349800, TA329470, TA507032, TA507033, TA507048, TA507049, TA507052, TA507009: purchased from Origene) were tested in all combinations at two concentrations for each antibody on an *ex vivo* sample previously shown to be positive on a western blot at protein concentrations ranging from 80 µg to 1.25 µg per well. The "best" combinations were then tested with a "positive" and "negative" ex vivo sample. This produced the following assay.
CHAC1 antibody (ab184982, Abcam) was diluted to 2 µg/mL in PBS and 25 µL per well was added per well of Meso Scale Discovery's multi-array 96-well standard bind plates and incubated overnight at 4 °C. Plates were blocked with Blocker A for 1 h, washed with wash buffer, 30 µg sample added per well and incubated overnight at 4 °C. Plate was washed incubated with 1 µg/mL ab180380 (Abcam) for 1 h, washed, incubated with 2 µg/mL sulpho-tag goat anti-mouse detection antibody (MSD®) for 1 h, washed and 2x read buffer added and read on MESO SQ QuickPlex 120. Ab180380 and the sulpho-tag antibodies were diluted in 1% BSA in wash buffer.

Pharmacokinetics Experimental Procedures

Mouse Pharmacokinetics
Female BALB/c mice were obtained from Charles River (Margate, UK). CF1 wild type and mdr1a P-gp deficient CF1 mice (CF1-Abcb1a^{mds}) were obtained from Charles River (Wilmington, MA, USA). Animals were adapted to laboratory conditions for at least 1 week prior to dosing and were allowed food and water ad libitum. Compounds were administered iv or po (0.1 mL/10g) in 10 % DMSO in 25 % w/v hydroxypropyl beta cyclodextrin in 50 mM sodium citrate buffer or 10% DMSO, 5% Tween 20, 85% Saline. Blood samples were collected from the tail vein (20 µL) at 8 time points over 24 h post dosing and spotted onto Whatman FTA-DMPK B cards (VWR) together with a calibration curve and quality controls spiked in control blood. Cards were allowed to dry at room temperature for at least 2 h. 6 mm discs were punched from the cards and extracted with 200 µL methanol containing 500 nM olomoucine as an internal standard. Following centrifugation, extracts were analyzed by multiple reaction monitoring of precursor and product ions by LC-ESI-MS/MS on a QTRAP 4000 (Sciex, Warrington, UK) using a short gradient consisting of 0.1 % formic acid and methanol on a Phenomenex (Macclesfield, UK) Kinetex™ C18, 5 cm x 2.6 µm, 2.1 mm i.d UHPLC column. Pharmacokinetic parameters were derived from non-compartmental analysis using Phoenix (model 200 and 201) Pharsight WinNonlin® version 6.1/6.3.

Rat Pharmacokinetics
Female CD rats were obtained from Charles River (Margate, UK). Animals were adapted to laboratory conditions for at least 5 days prior to dosing and were allowed food and water ad libitum. Compounds were administered iv or po (1 mL/200 g) in 10% DMSO in 25% w/v hydroxypropyl beta cyclodextrin in 50 mM sodium citrate buffer or . Blood samples were collected from the tail vein (20 µL) at 8 time points over 24 h post dosing and spotted onto Whatman FTA-DMPK B cards (VWR) together with a calibration curve and quality controls spiked in control blood. Cards were allowed to dry at room temperature for at least 2 h. 6 mm discs were punched from the cards and extracted with 200 µL methanol containing internal standard. Following centrifugation, extracts were analyzed by multiple reaction monitoring of precursor and product ions by LC-ESI-MS/MS on a QTRAP 4000 (Sciex, Warrington, UK) using a short gradient consisting of 0.1% formic acid and methanol on a Phenomenex (Macclesfield, UK) Kinetex™ C18, 5 cm x 2.6 µm, 2.1 mm i.d. UHPLC column. Pharmacokinetic parameters were derived from non-compartmental analysis using Phoenix (model 200 and 201) Pharsight WinNonlin® version 6.1/6.3.

Dog Pharmacokinetics
The dog PK live phase, sponsored by the CRT Pioneer Fund LP, was carried out at Charles River Laboratories (Spencerville, OH, USA). Animals were adapted to laboratory conditions for at least 7 days prior to dosing and were allowed food and water ad libitum. On day 1, compound was administered twice to 2 male and 2 female dogs by iv bolus (1 ml/kg) for a final dose of 0.5 mg/kg/dose in 10% DMSO in 25% w/v hydroxypropyl beta cyclodextrin in 50 mM sodium citrate buffer, pH 5. Following a washout period, compound was administered twice in one day to 2 male and 2 female dogs by oral gavage (5 ml/kg) for a final dose of 2.5 mg/kg/dose in 10% DMSO in 25% w/v hydroxypropyl beta cyclodextrin in 50 mM sodium citrate buffer, pH 5. Blood was collected at 11 timepoints (including predose) by venipuncture of the jugular vein, centrifuged for plasma and tranported to ICR, Sutton, UK on dry ice for analysis. Plasma samples were then thawed and compound was measured against a matrix-matched calibration curve and quality controls by LC-MS-MS (see Rat Pharmacokinetics) post protein precipitation with 3 volumes of methanol containing internal standard. Pharmacokinetic parameters were derived from non-compartmental analysis using Phoenix (model 200 and 201) Pharsight WinNonlin® version 6.1/6.3.

Plasma Protein Binding
Protein binding was measured using Rapid Equilibrium Dialysis (RED, Thermo Fisher Scientific, Loughborough, UK). Plasma was obtained from either female Balb/C mice or female CD (SD) rats (Charles River, Margate, UK) and stored at -20 °C. Cell culture media was DMEM (Sigma Aldrich, Dorset, UK) supplemented with 10 % FCS (Invitrogen), 2 mmol/L of L-glutamine, and 1 x non-essential amino acids. The RED plate, buffer, plasma and media solutions were heated to 37 °C before dialysis. Test compound in DMSO was spiked into either diluted plasma (10-fold dilution in 100 mM phosphate buffer) or cell culture media as appropriate resulting in a concentration of 5 µM for dialysis, containing 1% DMSO. 300 µL of spiked diluted plasma or media was added to the donor side of the RED plate and 500 µL of 100 mM phosphate buffer was added to the receiver well. The plate was sealed with a gas-permeable lid and dialysis was performed by shaking for 4 h at 37 °C. After dialysis, samples were transferred from the RED plate and donor and receiver samples were matrix matched followed by protein precipitation with methanol containing internal standard. Samples were mixed, centrifuged and supernatant was taken for analysis by ESI-LCMS/MS on a QTRAP 4000 (Sciex, Warrington, UK) using a short gradient consisting of 0.1 % formic acid and methanol on a Phenomenex (Macclesfield, UK) Kinetex™ C18, 5 cm x 2.6 µm, 2.1 mm i.d. UHPLC column. The fraction unbound (f_u) was calculated as follows:

$f_u' = \frac{\text{PAR receiver}}{\text{PAR donor}}$ where PAR = Peak Area Ratio of Analyte/Internal Standard.

$f_u = 1/(1 + \left(\frac{1}{fu'} - 1\right) * \text{dilution factor})$ where dilution factor = 10 for plasma, 1 for media.

Blood to Plasma Ratio
Fresh blood was obtained from either female Balb/C mice or female CD (SD) rats (Charles River, Margate, UK) and an aliquot centrifuged to obtain plasma. Blood and plasma was pre-warmed to 37 °C. Test compound was spiked into blood and plasma to a final concentration of 1 µM containing 1 % of methanol:water and ≤0.1 % DMSO. Spiked samples were incubated for 30 minutes at 37 °C. Blood was then centrifuged to obtain plasma. Equal volumes of plasma from centrifuged blood and from original spiked plasma samples were protein precipitated with 10-fold methanol containing internal standard, mixed and centrifuged. Supernatant was taken for analysis by ESI-LCMS/MS on a QTRAP 4000 (Sciex, Warrington, UK) using a short gradient consisting of 0.1 % formic acid and methanol on a Phenomenex (Macclesfield, UK) Kinetex™ C18, 5 cm x 2.6 µm, 2.1 mm i.d. UHPLC column. The blood to plasma ratio was calculated as follows:

$\frac{\text{PAR in Spiked Plasma}}{\text{PAR in Plasma from Spiked Blood}}$ where PAR = Peak Area Ratio of Analyte/Internal Standard.

Caco-2 Assay Protocol
P_{app} (apparent permeability) was determined in the Caco-2 human colon carcinoma cell line. Cells were maintained (DMEM with 10% fetal bovine serum, penicillin, and streptomycin) in a humidified atmosphere with 5% CO_2/95% air for 10 days. Cells were plated out onto a cell culture assembly plate (Millipore, UK), and monolayer confluency was checked using a TEER electrode prior to the assay. Media was washed off and replaced in the appropriate apical and basal wells with HBSS buffer (pH 7.4) containing compound (10 µM, 1% DMSO). The Caco-2 plate was incubated for 2 h at 37 °C, and Lucifer Yellow was used to confirm membrane integrity after the assay. Samples from the apical and basolateral chambers were analyzed using a Waters TQ-S LC-MS/MS system.

CYP Inhibition Protocol
The potential for inhibiting eight different Cytochrome P450 enzymes, notably 1A2, 2A6, 2B6, 2C8, 2C9, 2C19, 2D6, 3A4, was determined using a cocktail of CYP450 - specific substrates (1A2: phenacetin; 2A6: coumarin; 2B6: bupropion; 2C8: paclitaxel; 2C9: tolbutamide; 2C19: mephenytoin; 2D6: bufuralol; 3A4: midazolam and testosterone) in human liver microsomes (Tebu-bio, Peterborough, UK). Test compound was incubated at 1, 10 and 50 µM (final DMSO concentration ≤ 0.5%) in 10 mM PBS containing 0.5 mg/mL human liver microsomes, 2 mM NADPH and 2 mM NADH. Control samples were incubated in equivalent conditions containing sample solvent only. Samples were incubated for 10 minutes at 37 °C post addition of the mixture of substrates then reactions quenched with 2 parts methanol containing internal standard. Marker metabolites were measured by LC-MS on a Waters TQ-S triple quadrupole mass spectrometer coupled to an H-class acquity LC system. % inhibition was determined by comparison of metabolite response with controls.

In Vitro Microsomal Stability Assay (Cyprotex)
For the screening protocol and assay conditions see: http://www.cyprotex.com/admepk/in-vitro-metabolism/microsomal-stability (February 2017).
Test compound (1 µM) is incubated with pooled liver microsomes. Test compound is incubated at 5 time points over the course of a 45 min experiment and the test compound is analyzed by LC MS/MS.

Experimental Procedure:
Pooled human liver microsomes (male and female), dog liver microsomes (male Beagle), minipig liver microsomes (male Gottingen), rat liver microsomes (male Sprague Dawley rats) and mouse liver microsomes (male CD1 mice) are purchased from a reputable commercial supplier. Microsomes are stored at -80 °C prior to use. Microsomes (final protein concentration 0.5 mg/mL), 0.1 M phosphate buffer pH 7.4 and test compound (final substrate concentration 1 µM; final DMSO concentration 0.25 %) are pre incubated at 37 °C prior to the addition of NADPH (final concentration 1 mM) to initiate the reaction. The final incubation volume is 50 µL. A control incubation is included for each compound tested where 0.1 M phosphate buffer pH 7.4 is added instead of NADPH (minus NADPH). Two control compounds are included with each species. All incubations are performed singularly for each test compound.
Each compound is incubated for 0, 5, 15, 30 and 45 min. The control (minus NADPH) is incubated for 45 min only. The reactions are stopped by transferring 25 µL of incubate to 50 µL methanol at the appropriate time points. The termination plates are centrifuged at 2,500 rpm for 20 min at 4 °C to precipitate the protein.
Quantitative Analysis:
Following protein precipitation, the sample supernatants are combined in cassettes of up to 4 compounds, internal standard is added and samples analyzed using Cyprotex generic LC MS/MS conditions.
Data Analysis:
From a plot of ln peak area ratio (compound peak area/internal standard peak area) against time, the gradient of the line is determined. Subsequently, half-life and intrinsic clearance are calculated. Two control compounds are included in the assay and if the values for these compounds are not within the specified limits the results are rejected and the experiment repeated.
Table S19. Microsome data

BISAMIDE **22** LIVER MICROSOME METABOLIC STABILITY							
Entry	Species	Compound Remaining (% of 0 min)					
		0 min	5 min	15 min	30 min	45 min	control
1	mouse	100*	107	96.1	80.4	76.2	107
2	rat	100	89.7	88.6	78.3	70.2	104
3	minipig	100	17.2	0.909	0.0289	0.0179	1.04
4	dog (f)	100	80.7*	81.3	69.2	61.2	97.0
5	dog (m)	100	93.4	86.4	69.5	64.6	105
6	human	100	87.7	79.8	69.8	71.2*	99.2

*data point discarded

$$ln[S]_t = -k * t + Ln[S]_0$$
$$V = \frac{volume\ of\ incubation}{protein\ in\ the\ incubation}$$
$$In\ vitro\ Intrinsic\ Clearance\ (Cl_{int}) = V * k$$

Figure S16. CCT361814 (22) Metabolism data

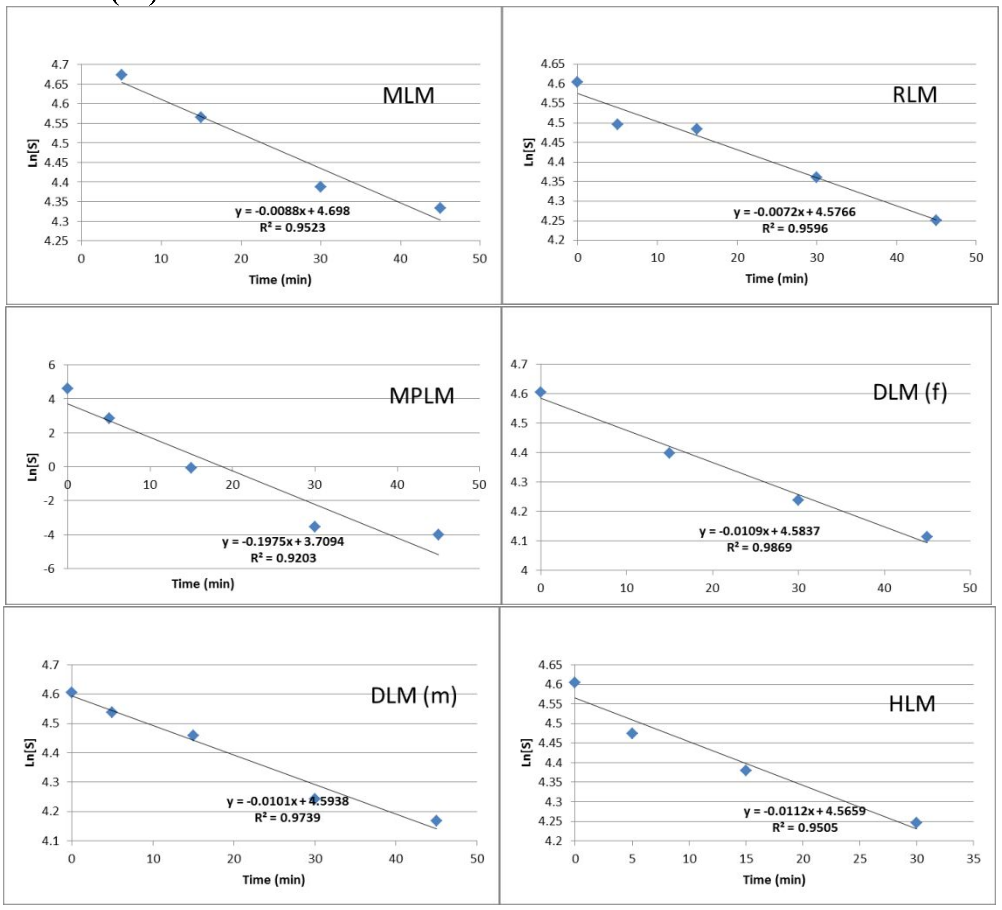

In Vitro Hepatocyte Stability Assay (Cyprotex)
For the screening protocol and assay conditions see: http://www.cyprotex.com/admepk/in-vitro-metabolism/hepatocyte-stability (February 2017).
Test compound (1 µM) is incubated with cryopreserved hepatocytes in suspension. Samples are removed at 6 time points over the course of a 60 min experiment and test compound is analyzed by LC-MS/MS. An intrinsic clearance value (CL_{int}) with standard error and half-life ($t_{½}$) is delivered.

Experimental Procedure:
Cryopreserved pooled hepatocytes are purchased from a reputable commercial supplier. Cryopreserved hepatocytes are stored in liquid nitrogen prior to use.

Williams E media supplemented with 2 mM L-glutamine and 25 mM HEPES and test compound (final substrate concentration 1 µM, final DMSO concentration 0.25%) are pre-incubated at 37 °C prior to the addition of a suspension of cryopreserved hepatocytes (final cell density 0.5 x 10^6 viable cells/mL in Williams E media supplemented with 2 mM L-glutamine and 25 mM HEPES) to initiate the reaction. The final incubation volume is 500 µL. Two control compounds are included with each species, alongside appropriate vehicle control. The reactions are stopped by transferring 50 µL of incubate to 100 µL methanol containing internal standard at the appropriate time points. The termination plates are centrifuged at 2500 rpm at 4 °C for 30 min to precipitate the protein.

Quantitative Analysis:
Following protein precipitation, the sample supernatants are combined in cassettes of up to 4 compounds and analyzed using Cyprotex generic LC-MS/MS conditions.

Data Analysis:
From a plot of ln peak area ratio (compound peak area/internal standard peak area) against time, the gradient of the line is determined. Subsequently, half-life ($t_{½}$) and intrinsic clearance (CL_{int}) are calculated using the equations below:

Elimination rate constant (k) = (- gradient)

Half-life ($t_{½}$)(min) = $\frac{0.693}{k}$

Intrinsic clearance (CL_{int})(µL/min/million cells) = $\frac{V \text{ x } 0.693}{t_{½}}$

where V = Incubation volume (µL)/Number of cells

Two control compounds for each species are included in the assay and if the values for these compounds are not within the specified limits the results are rejected and the experiment repeated.

$$ln[S]_t = -k * t + Ln[S]_0$$

$$V = \frac{volume\ of\ incubation}{protein\ in\ the\ incubation}$$

$$In\ vitro\ Intrinsic\ Clearance\ (Cl_{int}) = V * k$$

Table S20. Hepatocyte data

BISAMIDE **22** HEPATOCYTE METABOLIC STABILITY							
Entry	Species	Compound Remaining (% of 0 min)					
		0 min	10 min	20 min	40 min	60 min	control
1	mouse	100*	87.8	78	38.8	16.8	144
2	rat	100*	93.3	91.5	56.9	35.6	126
3	minipig	100*	1.34	0.0735	0.0119	0.00123	50.0
4	dog (m)	100*	98.4	90.1	64.1	46.9	107
5	dog (f)	100*	96.5	78	49.4	26.3	N/D
6	human	100*	110	104	95.8	90.6	139

*data point discarded.

Figure S17. Hepatocyte graphs

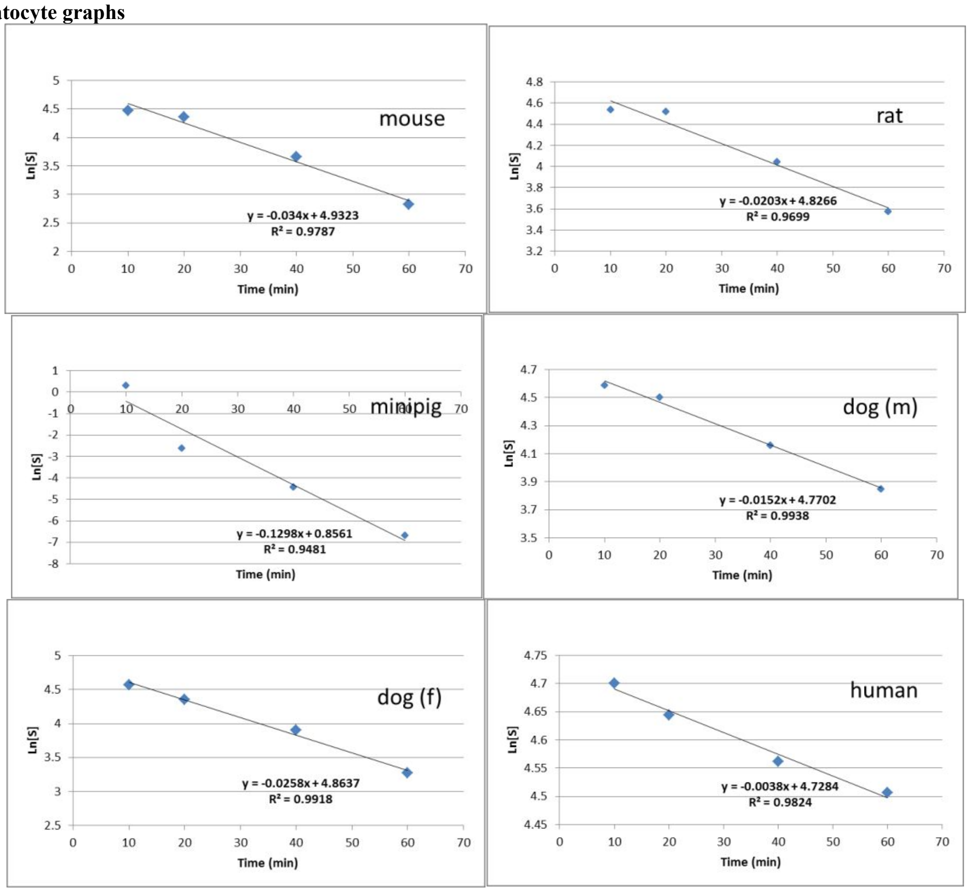

REFERENCES

[1] (a) Hosea, N. A.; Collard, W. T.; Cole, S.; Maurer, T. S.; Fang, R. X.; Jones, H.; Kakar, S. M.; Nakai, Y.; Smith, B. J.; Webster, R.; Beaumont, K. *J. Clin. Pharmacol.* **2009**, *49*, 513-533. (b) Sohlenius-Sternbeck, A-K *Toxicol. In Vitro* **2006**, 20, 1582-1586.

[2] (a) Obach, R. S.; Baxter, J. G.; Liston, T. E.; Silber, B. M.; Jones, B. C.; MacIntyre, F.; Rance, D. J.; Wastall, P. *J. Pharmacol. Exp. Ther.* **1997**, *283*, 46-58. (b) Di, L.; Feng, B.; Goosen, T. C.; Lai, Y.; Steyn, S. J.; Varma, M. V.; Obach, R. S. *Drug Metab. Dispos.* **2013**, *41*, 1975-1993.

[3] Bowes, J; Brown, A.J.; Hamon, J.; Jarolimek, W.; Sridhar, A.; Waldron, G.; Whitebread, S. *Nat. Rev. Drug Discov.* **2012**, *11*, 909-922.

[4] In-house PAINS filter protocol: Biovia Pipeline Pilot Version 9.5.

[5] Cheeseman, M. D.; Chessum, N. E. A.; Rye, C. S.; Pasqua, A. E.; Tucker, M. J.; Wilding, B.; Evans, L. E.; Lepri, S.; Richards, M.; Sharp, S. Y.; Ali, S.; Rowlands, M.; O'Fee, L.; Miah, A.; Hayes, A.; Henley, A. T.; Powers, M.; Poele, R.; De Billy, E.; Pellegrino, L.; Raynaud, F.; Burke, R.; van Montfort, R. L. M.; Eccles, S. A.; Workman, P.; Jones, K. Discovery of a Chemical Probe Bisamide (CCT251236): An Orally Bioavailable Efficacious Pirin Ligand from an HSF1 Phenotypic Screen, *J. Med. Chem.* **2017**, *60*, 180-201.

[6] Kerns, E. H.; Li Di, L.; Petuskya, S.; Kleintop, T.; Huryn, D.; McConnell, O.; Carter, G. Pharmaceutical profiling method for lipophilicity and integrity using liquid chromatography-mass spectrometry. *J. Chromatogr. B: Anal. Technol. Biomed. Life Sci.* **2003**, *791*, 371-388..

[7] Workman, P.; Aboagye, E. O.; Balkwill, F.; Balmain, A.; Bruder, G.; Chaplin, D. J.; Double, J. A.; Everitt, J.; Farningham, D.; Glennie, M. J.; Kelland, L. R.; Robinson, V.; Stratford, I. J.; Tozer, G. M.; Watson, S.; Wedge, S. R.; Eccles, S. A. Guidelines for the welfare and use of animals in cancer research. *Br. J. Cancer* **2010**, *102*, 1555-1577.
